# Prion‐Like Protein LENG8‐Mediated Nucleation Drives Stress Granule Assembly

**DOI:** 10.1002/advs.77622

**Published:** 2026-09-08

**Authors:** Mingxing Zhang, Xin Wang, Yilan Teng, Zhicheng Wu, Jing Fan, Hongwen Zhu, Peng Dai

**Affiliations:** ^1^ Shanghai Key Laboratory of Maternal and Fetal Medicine, Clinical and Translational Research Center of Shanghai First Maternity and Infant Hospital, Frontier Science Center for Stem Cell Research, School of Life Sciences and Technology Tongji University Shanghai China; ^2^ The Key Laboratory of Developmental Genes and Human Disease, School of Life Science and Technology Southeast University Nanjing China; ^3^ Precise Genome Engineering Center School of Life Sciences Guangzhou University Guangzhou China

**Keywords:** LENG8, liquid‐liquid phase separation, nucleator, prion‐like domain, stress granule

## Abstract

Stress granules (SGs) are highly dynamic and reversible cytoplasmic biomolecular condensates formed via liquid‐liquid phase separation (LLPS) under various stresses. As inherently heterogeneous assemblies, SGs possess distinct stable cores (initial nucleation seeds), substructures, or microphases. However, the mechanisms governing the formation and heterogeneity of SG nucleation seeds, and their dynamic integration, remain largely unclear. Here, we demonstrate that LENG8 is recruited to SGs under multiple stress conditions and is indispensable for SG assembly. Upon stress exposure, nuclear LENG8 granules disassemble, enabling LENG8 to translocate into the cytoplasm and undergo LLPS to form independent initial nucleation foci distinct from canonical G3BP1/TIA1‐dependent seeds. Subsequently, these LENG8‐initiated foci merge into growing SGs through a direct interaction between the prion‐like domain of LENG8 and TIA1, facilitating SG expansion and maturation. Depletion of LENG8 or disruption of the LENG8‐TIA1 interaction markedly impairs SG formation. Using conditional *Leng8* knockout mice, we further establish that LENG8 deficiency attenuates stress‐induced SG assembly and increases cellular apoptosis in germ cells. Collectively, our study identifies LENG8 as a previously unrecognized SG nucleator, revealing the hierarchical assembly and integration mechanism of distinct nucleation modules during early SG biogenesis.

## Graphical abstract

LENG8 is a newly identified stress granule (SG) nucleator required for SG assembly. Following stress, nuclear LENG8 granules disassemble, allowing LENG8 to translocate into the cytoplasm and form independent nucleation foci. These foci fuse with canonical early G3BP1/TIA1 seeds via LENG8‐TIA1 binding to drive SG maturation. Loss of LENG8 hinders SG formation and promotes germ cell apoptosis in mice.

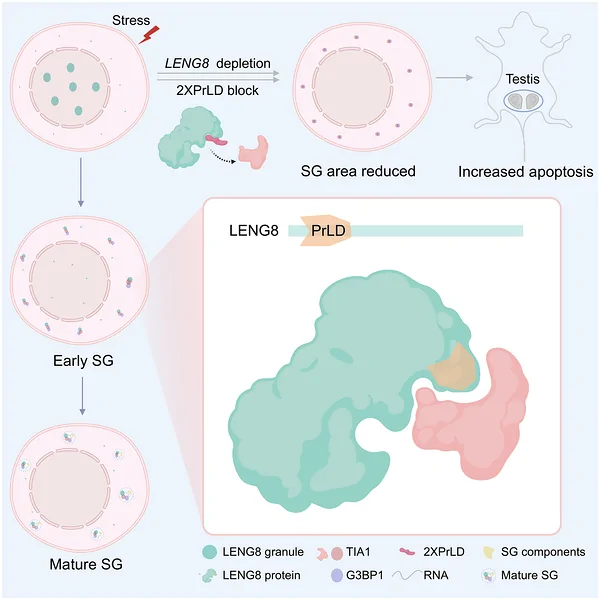

## 1 Introduction

Stress granules (SGs) are highly dynamic and reversible biomolecular condensates that form in the cytoplasm via liquid‐liquid phase separation (LLPS) in response to diverse intracellular or extracellular stress conditions, driven by weak, multivalent RNA‐RNA, protein‐protein, and protein‐RNA interactions [1]. Under normal conditions, ribosome‐coupled mRNAs and associated RNA‐binding proteins (RBPs) are distributed throughout the cytoplasm, with no microscopic evidence of SG assembly [2, 3]. Nevertheless, cumulative evidence from proximity labeling analyses has demonstrated that a pre‐existing protein interaction network among SG core proteins is present in the absence of stress [4, 5, 6, 7, 8]. These pre‐existing contacts among SG proteins may facilitate the rapid assembly of SGs in response to stress.

When exposed to various forms of stress, cells activate the integrated stress response (ISR) pathway, in which kinases including GCN2, HRI, PERK and PKR mediate the phosphorylation of eIF2α (p‐eIF2α) at serine 51 (S51) [9]. p‐eIF2α inhibits translation initiation, accompanied by the runoff of elongating ribosomes from mRNAs [10, 11, 12]. Consequently, untranslated messenger ribonucleoprotein (mRNP) complexes accumulate in the cytoplasm and subsequently recruit SG nucleators to drive the assembly of initial SGs or SG seeds [13, 14, 15, 16, 17]. SG nucleators are primarily constituted by RBPs that harbor key domains required for driving LLPS, including RNA‐binding domain (RBD), intrinsically disordered region (IDR), low‐complexity region (LCR), or prion‐like domain (PrLD) [18]. Recent studies have identified a 36‐protein core network, with G3BP1 serving as the central node [14, 19, 20]. G3BP1 contains an NTF2/2L domain that mediates its homodimerization and interactions with various SG nucleators, as well as an RNA‐recognition motif (RRM) and an adjacent arginine‐glycine (RG)‐rich IDR, all of which are essential for the LLPS of G3BP1 and the formation of SGs [14, 19, 20]. These SG core proteins that interact with G3BP1 act as bridges or nodes, providing additional and critical protein‐protein and protein‐RNA interfaces [20]. Moreover, as elevated concentrations of free cytosolic RNA disrupt the auto‐inhibited state of G3BP1, G3BP1 then acts as the central node and molecular switch to trigger RNA‐dependent LLPS and thereby initiates SG assembly [14, 19, 20]. After nucleation, “seed” SGs immediately recruit more proteins and RNAs, and gradually mature through growth and mutual fusion, in which several RNPs are also incorporated into G3BP1‐positive “seed” SGs in the form of microscopically observable independent foci or functional modules [15, 21, 22, 23].

Consistent with the observations made during SG maturation, electron microscopy and super‐resolution fluorescence microscopy analyses have revealed that SGs are inherently heterogeneous assemblies that possess distinct stable cores, substructures or microphases [20, 21, 24, 25, 26, 27]. A proposed simplified model, the “cores‑first” model, is used to explain the heterogeneity and layered architecture of SGs. It posits that during the early stages of stress, various types of RNA‐RNA, protein‐protein, and protein‐RNA interactions give rise to multiple distinct cores. As SGs undergo fusion and maturation, these initially dispersed cores gradually coalesce and recruit low‑concentration SG components to form a dynamic outer shell, ultimately assembling into large, microscopically visible SG granules [13, 20, 24, 25, 28, 29]. However, our understanding of how SG nucleation seeds form and acquire heterogeneity, and how different substructures mix and integrate remains extremely limited.

LENG8 (leukocyte receptor cluster member 8) is a poorly studied protein that contains an atypical PCI domain at its C‐terminus and an IDR together with a PrLD at its N‐terminus [30, 31, 32]. Recent studies demonstrate that LENG8 assembles with PCID2 and DSS1 into a TREX‐2‐like complex involved in mRNA export [31, 32]. Adipose‐specific conditional deletion of *Leng8* in mice lowers body weight and promotes adipose thermogenesis [32]. Furthermore, LENG8 also acts as a negative regulator of TREX‐2 to mediate nuclear retention and degradation of RNAs [30, 33]. We recently found that LENG8 forms a novel nuclear body through LLPS, termed the LENG8 granule, which functions as an active hub for alternative polyadenylation (APA) regulation [34]. However, whether LENG8 is also involved in regulating SG formation and how nuclear LENG8 granules are functionally linked to cytoplasmic stress granules remain unknown.

In this study, we show that LENG8 displays typical characteristics of a core protein and is essential for SG assembly. Under stress conditions, nuclear LENG8 granules undergo disassembly, and LENG8 subsequently translocates from the nucleus to the cytoplasm, where it forms abundant small foci through LLPS. These LENG8 foci differ from early G3BP1/TIA1 nucleation seeds and assemble independently of them. During SG maturation and fusion, LENG8 foci are targeted to G3BP1‐positive SGs through a direct interaction between the PrLD domain of LENG8 and TIA1. Recruitment of LENG8 foci promotes the growth and maturation of SGs. Intriguingly, LENG8 exhibits a punctate, non‐uniform distribution pattern inside mature stress granules. Either knockdown of LENG8 expression or blocking the direct interaction between LENG8 and TIA1 by PrLD‐derived peptides impairs SG formation, resulting in smaller SGs. Using conditional *Leng8* knockout mice, we found that LENG8 depletion impairs SG formation upon heat stress and in turn leads to increased apoptosis of germ cells. In summary, our study identifies LENG8 as a new SG nucleator that acts independently of the G3BP1/TIA1 seed formation and reveals the recruitment mechanisms of distinct SG seeds during the early stages of SG assembly.

## 2 Results

### 2.1 Stress‐Induced Translocation of LENG8 Protein into SGs

**FIGURE 1:**
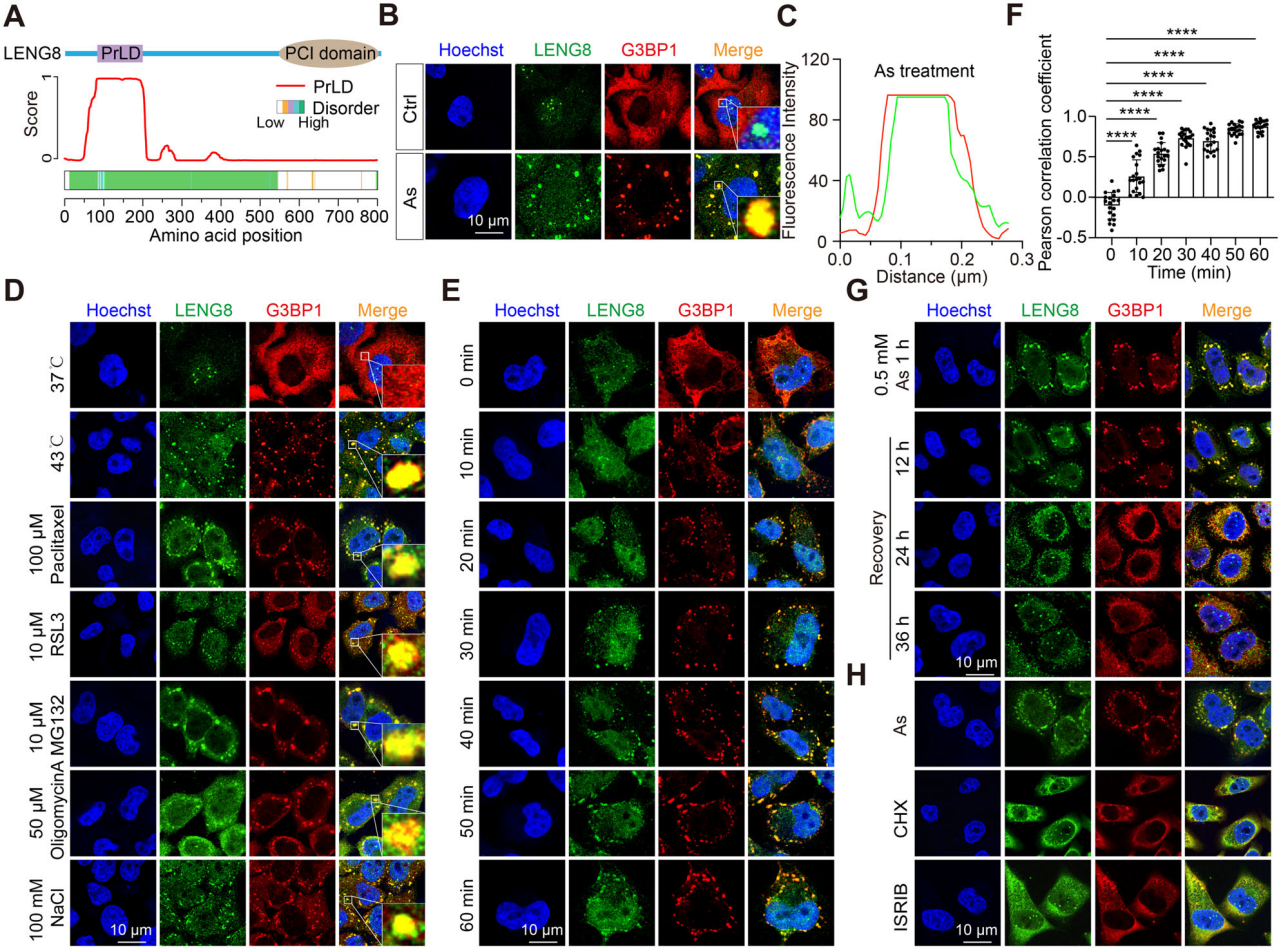
LENG8 granule disassembly and LENG8 relocalization to SGs under stress conditions. (A) Schematic diagram of domain analysis of LENG8. The PrLD (76‐96 aa) and IDR sequences are located at its N‐terminus, while the C‐terminus contains the PCI domain. The middle panel shows PrLD predictions of LENG8. Different colors in the bottom indicate high and low disorder prediction agreement. (B) Representative fluorescence images showing that arsenite (As) treatment induces LENG8 localization to SGs in HeLa cells. (C) Line scans display the related fluorescence intensity profiles of LENG8 (green) and G3BP1 (red). (D) Representative fluorescence images revealing LENG8 localization to SGs in HeLa cells under diverse stress conditions. (E) Representative fluorescence images showing the localization of LENG8 relative to SGs at the indicated time points upon As treatment in HeLa cells. (F) Pearson correlation coefficients quantifying LENG8‐G3BP1 colocalization at the indicated time points shown in (E). Data are presented as mean ± SD, *n* = 20, ****p <0.0001 by one‐way ANOVA. (G) Representative immunofluorescence images showing the localization of LENG8 relative to SGs during As treatment and recovery in HeLa cells. (H) Impact of inhibiting SG formation on LENG8 granule disassembly and LENG8 targeting to SGs in HeLa cells. CHX: cycloheximide; ISRIB: integrated stress response inhibitor.

Our previous study identified that the LENG8 protein N‐terminus encompasses an extended intrinsically disordered region (IDR) as well as a Q/Y rich prion‐like domain (PrLD) [34] (Figure 1A). Given the prevalence of IDRs and PrLDs in proteins that localize to SGs, we sought to investigate whether LENG8 translocates into stress granules under stress conditions. We treated HeLa cells with arsenite and subsequently performed immunofluorescence (IF) assays by co‐staining of G3BP1, TIAR, TIA1 or HUR, all well‐characterized marker of SGs. IF results revealed that LENG8 protein is specifically recruited into SGs upon arsenite‐induced oxidative stress. By contrast, we detected no LENG8 enrichment in membraneless P‐bodies (marked by DCP1A) or membrane‐bound organelles such as mitochondria (TOM20) and lysosomes (LAMP2) (Figure 1B,C and Figure S1A,B). Furthermore, at the same treatment duration, a higher concentration of arsenite resulted in a more pronounced effect on the recruitment of LENG8 into SGs (Figure S1C). We further examined the response of LENG8 protein to other stress treatments and found that additional stressors such as heat stress (43°C) could also induce the recruitment of LENG8 into SGs (Figure 1D and Figure S1D).

Ectopic expression of core SG nucleators G3BP1, TIA1, or the S51D phosphomimetic of eIF2α is sufficient to trigger SG formation even in the absence of stress [10, 15, 23, 35]. We found that LENG8 protein could also localize to SGs induced by overexpression of these proteins. In contrast, the non‐phosphorylatable eIF2α‐S51A mutant failed to trigger SG formation, and nuclear LENG8 granules remained stable under this condition (Figure S1E). Time‐course analyses revealed that under stress conditions, SGs marked by G3BP1 initially formed as small foci. Concomitantly, nuclear LENG8 granules underwent gradual disassembly and translocation to the cytoplasm, where LENG8 protein accumulated into small foci (Figure 1E,F). As these nascent G3BP1‐positive SGs matured and grew in size, LENG8 became progressively enriched within the SG structures (Figure 1E,F). After oxidative stress withdrawal, LENG8 was released from disassembling SGs, first appeared as discrete small foci in the cytoplasm, and eventually reconstituted nuclear LENG8 granules (Figure 1G). This process was markedly correlated with oxidative stress intensity, such that higher arsenite induction concentrations led to a longer duration for the disassembly of SGs and the recovery of LENG8 subcellular localization (Figure 1G and Figure S1F,G). Collectively, these results establish tight spatiotemporal coupling between LENG8 subcellular localization dynamics and SG biogenesis, suggesting that LENG8 may serve as a key participant in the stepwise assembly of SGs under stress conditions.

To further probe the functional linkage between nuclear LENG8 granules and cytoplasmic SG assembly, we utilized cycloheximide (CHX, a translation elongation inhibitor) and ISRIB (an inhibitor of the ISR) to block SG formation during oxidative stress [11, 36]. When SG biogenesis was suppressed by CHX or ISRIB treatment, stress‐triggered disassembly of nuclear LENG8 granules was markedly blocked. This observation demonstrates that the dissolution of LENG8 condensates is tightly coupled to SG formation (Figure 1H). We further confirmed that stress‐induced translocation of LENG8 into SGs is conserved across multiple cell lines (Figure S1H). Together, our findings identify LENG8 as a newly identified SG constituent that forms discrete foci independent of G3BP1‐positive seeds during the early stages of SG assembly, subsequently localizing to mature SGs. Our data also reveal that disassembly of nuclear LENG8 granules is coupled to SG formation.

### 2.2 LENG8 Exhibits Liquid‐Like Properties within SGs and Regulates SG Assembly

**FIGURE 2:**
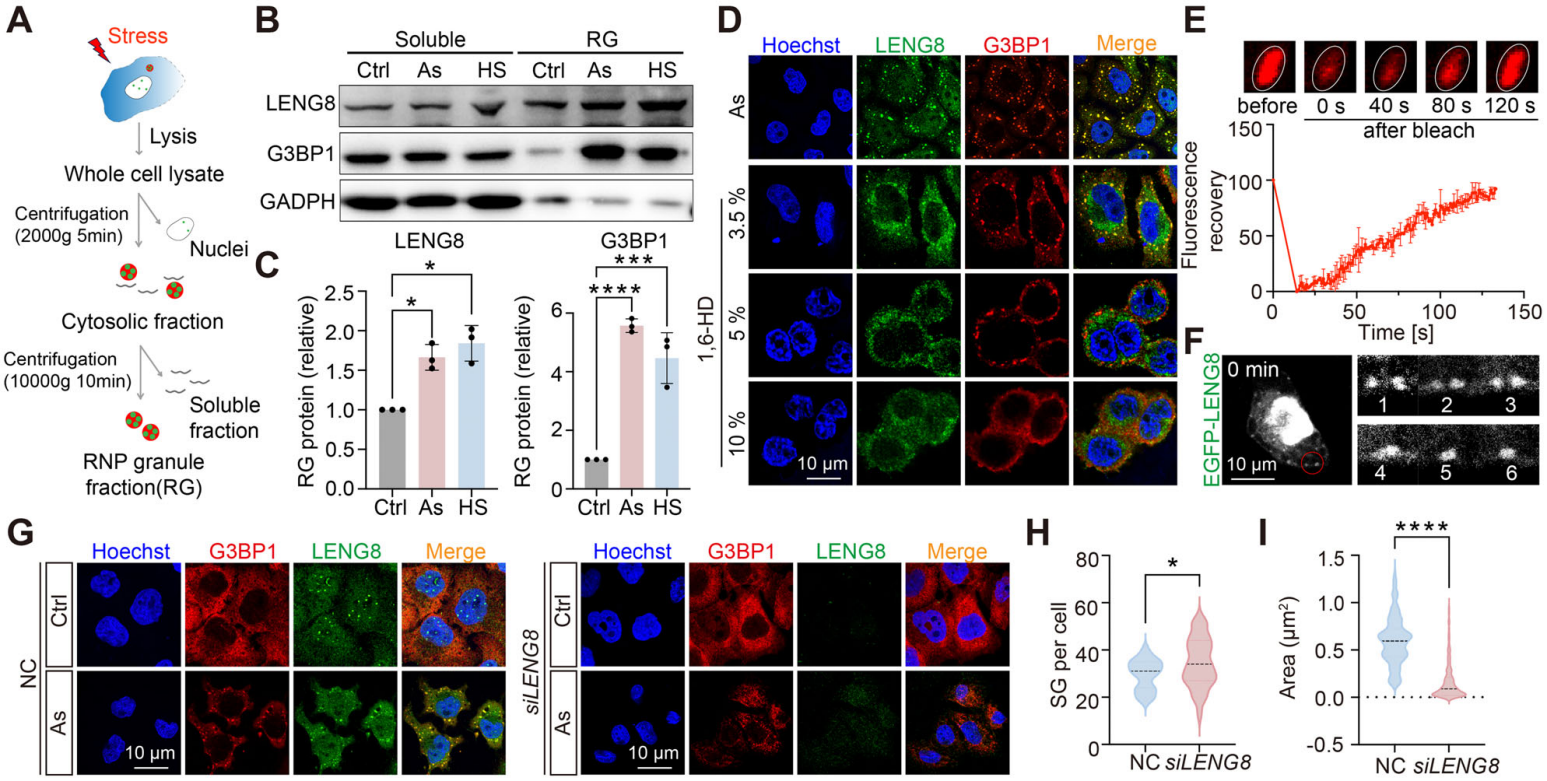
LENG8 possesses liquid‐like properties and regulates SG assembly. (A) Schematic diagram of fractionation of cell lysates by differential centrifugation. (B) Western blot analysis of LENG8 and G3BP1 distribution in soluble fractions versus RNP granules (RG) in HeLa cells under As treatment and heat stress. (C) Quantitative analysis of LENG8 and G3BP1 distribution in RG under As treatment and heat stress. Data are presented as mean ± SD, *n* = 3. *p <0.05, ***p <0.001, ****p <0.0001 by one‐way ANOVA. (D) Effect of treatment with different concentrations of 1,6‐hexanediol (1,6‐HD) on LENG8 localization within SGs in HeLa cells. (E) FRAP assays showed that fluorescence recovery of mCherry‐LENG8 foci after photobleaching in HeLa cells. The ellipse indicates the region being tested (top). Bottom: Quantification of fluorescence recovery of the mCherry‐LENG8 foci. The error bars denote the standard deviation calculated from three independent measurements at each time point (mean ± SD, *n* = 3). (F) Representative images showing the fusion of two EGFP‐LENG8 foci upon contact in HeLa cells. (G) Effect of LENG8 knockdown on G3BP1‐labeled SGs in HeLa cells. Left: negative control (NC) group; right: *LENG8* siRNA (si*LENG8*) group. (H, I) Quantitative analysis of the effect of LENG8 knockdown on the number (H) and area (I) of G3BP1‐labeled SGs. *n* = 35 (H) and *n* = 377 (I). The horizontal line inside each box indicates the median. *p <0.05, ****p <0.0001 by Student's *t*‐test.

To characterize LENG8 protein's properties in SGs, we first performed differential centrifugation to fractionate cytoplasmic lysates into soluble fractions and insoluble ribonucleoprotein (RNP) granule fractions containing SGs [37], and then analyzed the distribution of LENG8 (Figure 2A). Western blot (WB) assays revealed that, similar to G3BP1, LENG8 was enriched in RNP granule fractions under both arsenite‐induced oxidative stress and heat stress conditions (Figure 2B,C). As established in our previous study, LENG8 protein exhibits liquid‐liquid phase separation (LLPS) activity [34], and it forms small foci during SG biogenesis (Figure 1E,F). To test whether LLPS sustains LENG8 localization to SGs, we treated cells with 1,6‐hexanediol (1,6‐HD), a reagent that disrupts weak hydrophobic interactions supporting LLPS. 1,6‐HD treatment triggered dissociation of LENG8 from SGs and concurrently reduced SG size (Figure 2D). At 10% concentration, both SGs and LENG8 foci were almost completely disassembled, demonstrating that the formation of LENG8 foci as well as their enrichment within SGs are both mediated by LLPS driven by weak hydrophobic interactions (Figure 2D). Considering that LENG8 exists as small foci throughout SG formation, disassembly and recovery processes, these observations imply that LENG8 likely resides in SGs in the form of discrete substructures (Figure 1E–G and Figure 2D; Figure S1F,G). Consistently, high‐resolution imaging analyses validated these observations (Figure 1B,D and Figure S1D).

We next transfected exogenous mCherry‐tagged LENG8 into HeLa cells and employed fluorescence recovery after photobleaching (FRAP) assays to assess the mobility of LENG8 protein within SGs. FRAP measurements revealed rapid fluorescence recovery of mCherry‐LENG8, indicative of liquid‐like molecular mobility (Figure 2E). To further explore the nature of EGFP‐LENG8 foci, we tracked their dynamic behavior during the initial phase of SG formation. These protein foci rapidly fused into larger droplets, a phenomenon that further confirms their liquid‐like properties (Figure 2F). Given that SG assembly is RNA‐dependent, we tested the impact of RNA on the LLPS of LENG8 within SGs [14, 19]. Treatment with RNase A disassembled SGs in cells, causing LENG8 to dissociate from SGs and instead form small foci (Figure S2A,B). In line with the observations in Figure 1, LENG8 foci assembly through LLPS occurs in an RNA‐independent manner, while the recruitment /localization of LENG8 into SGs strictly relies on the maintenance of SG structural integrity.

These findings led us to hypothesize that small LENG8 foci generated under stress conditions are required for the subsequent maturation and size expansion of SGs. To verify this assumption, we knocked down *LENG8* via siRNA, which resulted in an increased number of SGs and a reduction in their size (Figure 2G–I and Figure S2C–F). The reduction in SG size caused by *LENG8* knockdown was not attributable to changes in the expression levels of core SG components (including G3BP1 and TIA1) or the phosphorylation level of eIF2α (Figure S2D). We further asked whether LENG8 overexpression alone, analogous to known SG nucleators G3BP1 and TIA1, could initiate SG formation under unstressed conditions. Ectopic expression of Myc‐LENG8 only drove formation of nuclear LENG8 condensates, consistent with our previous report [34], yet it did not trigger SG assembly in the cytoplasm (Figure S2G,H). To further address this, we transfected cells with EGFP‐LENG8 plasmids fused to a nuclear export signal (NES) to selectively elevate cytoplasmic LENG8 levels. Notably, increased cytoplasmic levels of EGFP‐LENG8 not only induced the formation of discrete small LENG8 foci but also triggered *de novo* SG assembly under unstressed conditions (Figure S2I).

In summary, our findings demonstrate that increased LENG8 abundance in the cytoplasm, regardless of whether it is achieved through ectopic overexpression or stress‐elicited elevation, can induce the formation of liquid‐like foci mediated by LLPS. As a novel SG nucleator, LENG8 plays a critical role in promoting the maturation and growth of SGs under stress conditions.

### 2.3 PrLD‐Mediated Recruitment of LENG8 into SGs

**FIGURE 3:**
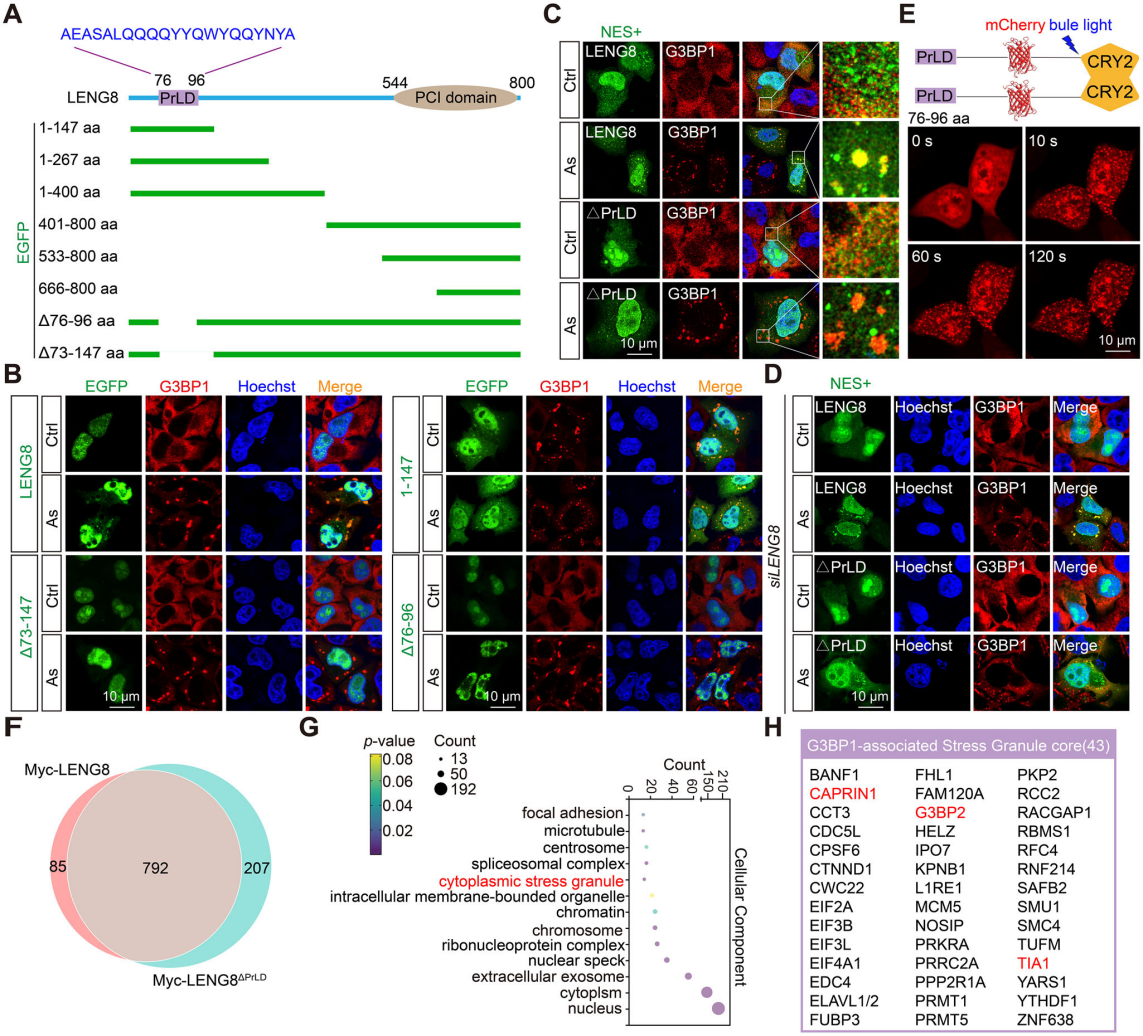
PrLD‐mediated phase separation of LENG8 and its targeting to SGs. (A) Schematic diagram of the construction of EGFP‐LENG8 mutants. (B) Representative immunofluorescence images showing the localization of different EGFP‐LENG8 mutants to SGs in HeLa cells under As treatment. (C) Deletion of PrLD (ΔPrLD) impairs the targeting of LENG8 to SGs in HeLa cells. (D) Effect of ΔPrLD on LENG8 localization and SG assembly under endogenous LENG8 knockdown in HeLa cells. (E) Optogenetic system for validating the phase separation capacity of PrLD (76‐96 aa) in HEK293T cells. (F) Intersection proteins of the interactomes between full LENG8 and the ΔPrLD mutant in HEK293T cells. (G) GO analysis of proteins with decreased interaction with the ΔPrLD mutant compared with full LENG8. (H) ΔPrLD impairs the interaction between LENG8 and multiple G3BP1‐associated SG proteins.

In order to map the domains responsible for LENG8 localization to SGs, we generated a series of EGFP‐tagged LENG8 constructs (Figure 3A). Consistent with the observation that elevating the cytoplasmic concentration of full‐length LENG8 is sufficient to induce the spontaneous formation of SGs under non‐stress conditions (Figure S2I), overexpression of the variants 1–147 aa and 1–267 aa also triggered *de novo* assembly of SGs (Figure 3B and Figure S3A). Under stress conditions, EGFP‐LENG8 variants harboring the N‐terminal PrLD (1‐147 aa, 1–267 aa, and 1–400 aa) successfully localized to SGs, whereas deletion of the PrLD (Δ73‐147 aa, Δ76‐96 aa) abolished this translocation (Figure 3B and Figure S3A–C). The C‐terminal fragments (533‐800 aa, 666–800 aa) formed cytoplasmic foci but did not colocalize with SGs (Figure S3A). Furthermore, both mCherry and Myc tags do not affect the recruitment of LENG8 into SGs under stress conditions (Figure S3D).

Notably, PrLD deletion caused prominent nuclear retention of LENG8, which complicated phenotypic interpretation. To eliminate this confounding factor, we appended an NES sequence to EGFP‐tagged ΔPrLD LENG8 to raise its cytoplasmic abundance. Although NES‐fused ΔPrLD LENG8 formed small cytoplasmic puncta, its recruitment into SGs was substantially diminished compared with NES‐tagged full‐length LENG8 (Figure 3C and Figure S3E). In rescue experiments, full‐length LENG8 was capable of restoring the smaller SG phenotype induced by LENG8 deficiency, whereas the ΔPrLD LENG8 mutant lacked this rescuing activity due to its inability to be recruited into SGs (Figure 3D). These data demonstrate that the PrLD is essential for LENG8 targeting to SGs and the subsequent size expansion under stress conditions.

PrLDs are widely recognized as potent drivers of phase separation. We therefore adopted an optogenetic CRY2 system to test whether the LENG8 PrLD is sufficient to trigger condensate assembly in the absence of stress [38, 39, 40, 41]. Under blue light stimulation, proteins with PrLD‐containing sequences (1‐147 aa, 76–96 aa, and 73–147 aa) inserted at the N‐terminus of the optogenetic module mCherry‐CRY2 rapidly formed multiple small foci via CRY2‐dependent oligomerization within 1 min, which gradually increased in size following 3 min of light activation (Figure 3E and Figure S3F). These results reveal that the PrLD acts as a driver of LENG8 protein LLPS events. Nevertheless, we found that the C‐terminal regions (533‐800 aa, 666–800 aa) of LENG8 protein also assemble into droplet‐like foci within cells (Figure S3A). Furthermore, loss of the PrLD does not completely impair the phase separation ability of LENG8 in the cytoplasm (Figure 3C,D). This suggests the PrLD acts cooperatively with the C‐terminal region to support full LLPS capacity of LENG8. To gain mechanistic insights into the role of the PrLD in mediating LENG8 translocation to SGs, we employed immunoprecipitation‐mass spectrometry (IP‐MS) assays to characterize alterations in the protein interaction profiles of full‐length LENG8 versus its ΔPrLD variant under stress conditions. Data analysis revealed that the mutation indeed altered the interaction profile of LENG8 protein, including attenuated interactions with key SG components, indicating that this region may mediate LENG8 recruitment to SGs by facilitating interactions with SG proteins (Figure 3F–H and Table S1).

Taken together, this species‐conserved PrLD enriched in Q/Y residues not only facilitates LENG8 translocation to SGs but also cooperates with the C‐terminal region to drive the LLPS process (Figure S3G,H).

### 2.4 PrLD Bridges LENG8‐TIA1 Interaction to Direct LENG8 Targeting to SGs

**FIGURE 4:**
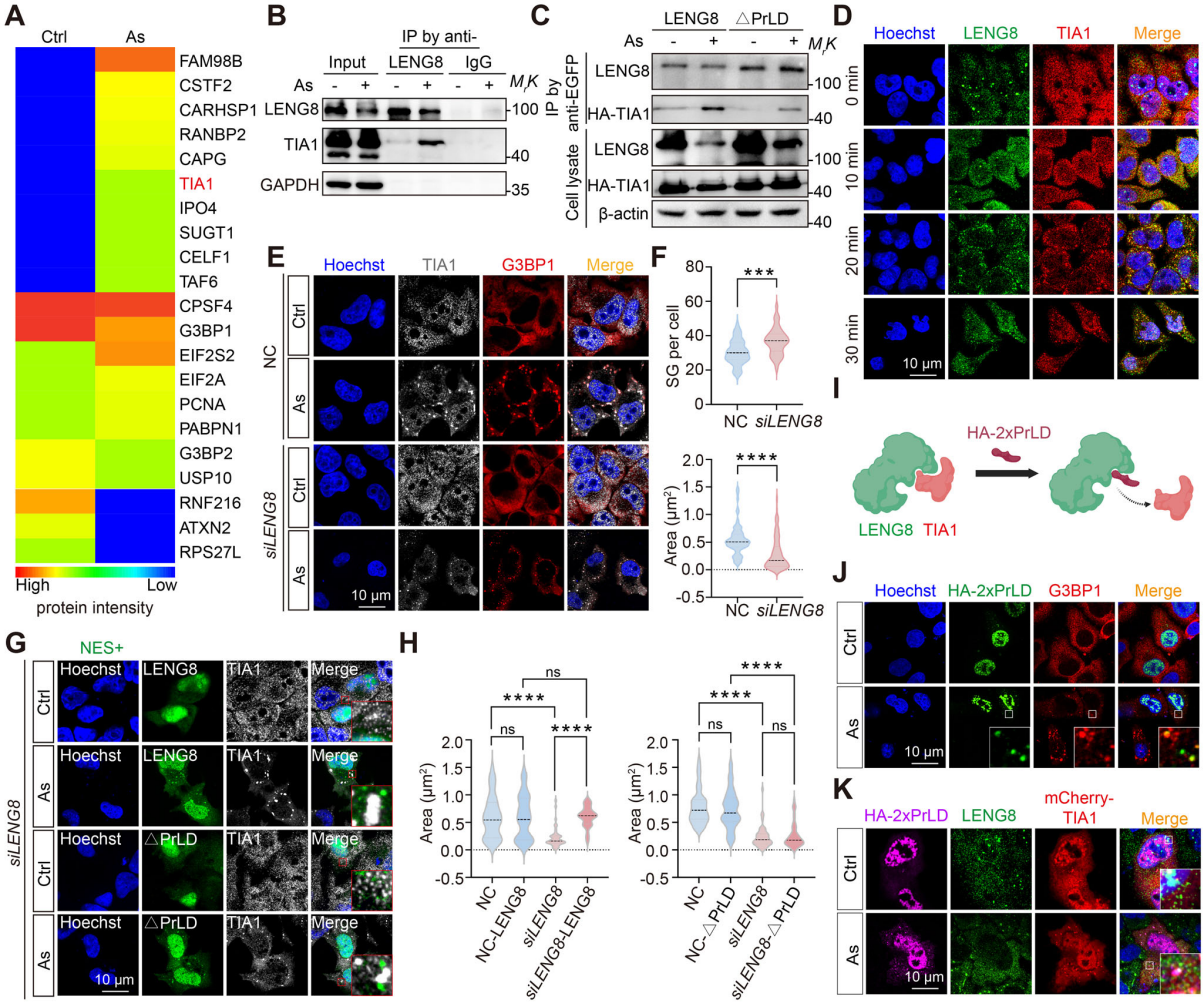
PrLD‐mediated LENG8‐TIA1 interaction regulates LENG8 targeting to SGs. (A) Mass spectrometry data and heat map analysis of changes in the LENG8‐interacting protein profile before and after As treatment in HeLa cells. (B) The interaction between endogenous LENG8 and TIA1 is enhanced under As treatment in HeLa cells. (C) Co‐IP assay of the interaction between EGFP‐LENG8 and HA‐TIA1 co‐transfected in HEK293T cells. ΔPrLD markedly weakens the interaction between LENG8 and TIA1 under As treatment. (D) Representative fluorescence images illustrating the localization relationship between LENG8 and TIA1 at the indicated time points following As treatment in HeLa cells. (E) Knockdown of LENG8 expression alters the area and number of TIA1/G3BP1‐positive SGs in HeLa cells. (F) Quantitative analysis of the effect of LENG8 knockdown on the number (top) and area (bottom) of TIA1/G3BP1‐positive SGs. The horizontal line inside each box indicates the median, *n* = 41 (top) and *n* = 171 (bottom). ***p <0.001, ****p <0.0001 by Student's *t*‐test. (G, H) Representative fluorescence images (G) and quantitative analysis (H) show that ΔPrLD LENG8 fails to rescue the alterations in the area and number of TIA1‐positive SGs in HeLa cells, compared with full‐length LENG8. The horizontal line inside each box indicates the median, *n* = 36 (left) and *n* = 44 (right). ns, not significant, ****p<0.0001 by two‐way ANOVA. (I) Schematic diagram of tandem PrLDs blocking the LENG8‐TIA1 interaction. The diagram was created with BioRender.com. (J) Representative fluorescence images illustrating that tandem PrLDs localize to SGs under As treatment in HeLa cells. (K) Representative fluorescence images showing that tandem PrLDs inhibit the formation of TIA1‐positive SGs in HeLa cells.

To elucidate the molecular mechanism underlying PrLD‐mediated LENG8 targeting to SGs, we performed IP‐MS to analyze changes in the LENG8‐interacting protein profile before and after stress induction. Combined analysis of heatmap and GO term enrichment demonstrated that LENG8 exhibited enhanced interactions with key SG components following stress induction (Figure 4A; Figure S4A and Table S2). These key SG proteins were consistently identified as LENG8 interactors in both HEK293T and HeLa cells, suggesting that LENG8 may rely on similar interacting partners to target SGs across distinct cell types. (Figure S4B and Table S3). Based on these findings, we prioritized TIA1 to dissect the molecular basis of PrLD‐mediated LENG8 translocation to SGs. Co‐immunoprecipitation (Co‐IP) analysis confirmed that the interaction between LENG8 and TIA1 was significantly strengthened under stress conditions (Figure 4B). To investigate the role of the PrLD in mediating the LENG8‐TIA1 interaction, we co‐transfected cells with EGFP‐tagged full‐length LENG8 or ΔPrLD LENG8, together with HA‐tagged TIA1. Subsequent Co‐IP assays revealed that deletion of the PrLD dramatically diminished the binding between LENG8 and TIA1 under stress conditions (Figure 4C). Time‐course analyses revealed that under stress conditions, TIA1‐marked SGs also initially formed as small foci and colocalized with G3BP1 at the 20 min time point (Figure S4C,D). Interestingly, LENG8 first assembled into discrete foci that gradually increased in size, and its fusion with TIA1‐labeled SGs became more evident by 30 min (Figure 4D and Figure S4C). Concurrently, we also observed the presence of independent LENG8 foci (Figure 4D). This dynamic fusion pattern recapitulates the behavior observed for LENG8 puncta and G3BP1‐labeled SG precursors (Figure 1E and Figure S4C,E). Additionally, the ΔPrLD LENG8 variant did not promote its translocation into TIA1‐positive SGs, as opposed to the full‐length LENG8 (Figure S4F).

To further elucidate the functional implications of the PrLD‐driven LENG8‐TIA1 interplay, we first performed *LENG8* knockdown assays and assessed its impacts on TIA1 localization and SG assembly. Likewise, *LENG8* knockdown induced significant changes in both the size and number of TIA1‐positive SGs (Figure 4E,F). In *LENG8*‐knockdown cells, ectopic expression of full‐length LENG8 effectively restored the reduced SG size phenotype, whereas the ΔPrLD LENG8 mutant failed to exert such a rescuing effect (Figure 4G,H and Figure S4F). These results align with the inability of the ΔPrLD LENG8 mutant to restore G3BP1‐labeled SGs (Figure 3D), further confirming the synergistic role of TIA1 and G3BP1 during early SG assembly, as well as the recruitment of LENG8 foci into maturing SGs via PrLD‐mediated binding to TIA1. To visualize the LENG8‐TIA1 interaction directly in live cells, we employed the split‐GFP complementation assay, a powerful tool for detecting proximal protein‐protein interactions [42]. We exploited this system by fusing LENG8 to the GFP11 fragment and TIA1 to the GFP1‐10 fragment, followed by co‐transfecting these two constructs into HeLa cells and monitoring GFP fluorescence signals (Figure S4G). Functional GFP fluorescence is dependent on the interaction between LENG8 and TIA1, which drives the spatial approximation and subsequent complementation of the two GFP fragments. Crucially, the emergence of green fluorescence in SGs provides direct visual evidence for the physical association between LENG8 and TIA1 during SG assembly (Figure S4G). To further validate the functional importance of the PrLD in this complex, we constructed an HA‐tagged expression vector encoding two tandem repeats of the PrLD sequence (Figure 4I). Intriguingly, we observed that HA‐tagged tandem PrLDs alone were sufficient to translocate into SGs (Figure 4J,K). Moreover, their incorporation effectively blocked the LENG8‐TIA1 interaction, thereby preventing LENG8 from being recruited into TIA1‐positive SGs and leading to the formation of smaller SGs under stress conditions (Figure 4K and Figure S4H). At this stage, LENG8 still formed distinct foci in the cytoplasm that were independent of SGs (Figure 4K). We additionally tested the dependence of LENG8‐SG colocalization on TIA1. *TIA1* depletion impaired the formation of SGs (Figure S4I), a phenotype consistent with observations in previous studies [14, 43]. Strikingly, nuclear LENG8 granules still underwent disassembly and translocated to the cytoplasm to form small LENG8 foci. However, these foci did not colocalize with the small G3BP1‐positive SGs under TIA1‐depleted conditions (Figure S4I). In conclusion, these results demonstrate that PrLD drives the LENG8‐TIA1 interaction, which facilitate the fusion of small, independent LENG8 foci with TIA1/G3BP1‐positive SG precursors, ultimately leading to the formation of large, mature SGs.

### 2.5 PrLD‐Driven Phase Separation and Droplet Fusion with TIA1

**FIGURE 5:**
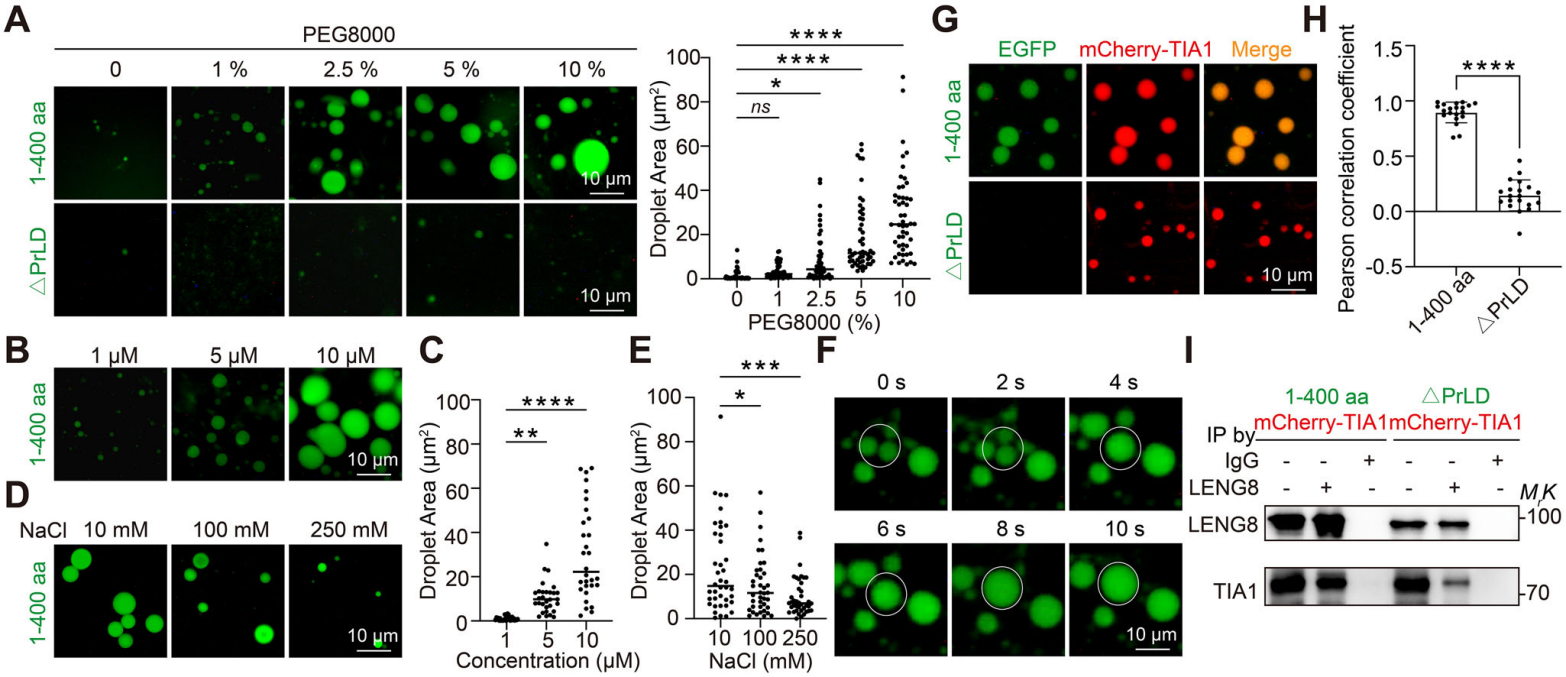
PrLD drives phase separation of LENG8 in vitro and promotes fusion with TIA1 droplets. (A) Effects of PEG8000 on droplet formation by 1 µM recombinant EGFP‐LENG8 (1–400 aa) and ΔPrLD mutant proteins in vitro. The right panel shows quantification of LENG8(1‐400 aa) droplet area at varying PEG8000 concentrations. The horizontal line indicates the median, *n* = 50, ns, not significant, *p<0.05, ****p < 0.0001 by one‐way ANOVA. (B) Droplet formation of recombinant EGFP‐LENG8 (1–400 aa) in vitro with varying protein concentrations (1 µM, 5 µM, 10 µM). (C) Quantification of droplet area of LENG8 (1–400 aa) proteins at varying concentrations shown in (B). The horizontal line indicates the median, *n* = 30, ** *p* < 0.01, ****p < 0.0001 by one‐way ANOVA. (D) Effects of varying NaCl concentrations on droplet formation by 5 µM recombinant EGFP‐LENG8 (1–400 aa) in vitro. (E) Quantification of droplet area of LENG8 (1‐400 aa) proteins as represented in (D). The horizontal line indicates the median, *n* = 40, *p<0.05, ***p<0.001 by one‐way ANOVA. (F) Fusion events of EGFP‐LENG8 (1–400 aa) droplets in vitro at 1 µM. (G) PrLD drives the fusion of LENG8 and TIA1 droplets in vitro at 1 µM. (H) Pearson correlation coefficient showing co‐localization patterns between LENG8 (1–400 aa) and mCherry‐TIA1 proteins in vitro as represented in (G). Data are shown as mean ± SD, *n* = 20, ****p <0.0001 by Student's *t*‐test. (I) ΔPrLD significantly impairs the interaction between LENG8 and TIA1 in vitro.

Several SG components contain PrLDs, and PrLD‐mediated LLPS has been implicated in SG formation [44]. Given that the PrLD collaborates with the C‐terminal region to drive the LLPS process of LENG8 under stress conditions, we utilized the N‐terminal LENG8 (1‐400 aa) to directly investigate the role of PrLD‐mediated LLPS in SG assembly. Similar to the full‐length LENG8 lacking the PrLD, the PrLD‐deleted 1–400 aa truncation was also unable to undergo recruitment to SGs (Figure S5A). In addition, PrLD deletion significantly reduced the interaction between LENG8 (1‐400 aa) and TIA1 (Figure S5B). We next performed in vitro reconstitution experiments to characterize the phase separation behaviour of purified recombinant proteins. EGFP‐tagged LENG8 (1‐400 aa) readily formed liquid droplets in a concentration‐dependent manner, and droplet formation was further promoted by the crowding agent PEG8000 (Figure 5A–C and Figure S5C). We additionally confirmed that the recombinant LENG8 (1‐400 aa) protein lacking the EGFP tag could still undergo LLPS in vitro, thus eliminating the potential interference of the EGFP tag on the phase separation behavior of LENG8 (Figure S5D–G). Increasing NaCl concentration impeded the phase separation of recombinant LENG8 (1‐400 aa) proteins (Figure 5D,E and Figure S5D,E). Furthermore, the LENG8 droplets displayed typical fusion and growth behaviors, indicative of their liquid‐like properties (Figure 5F). Conversely, deletion of the PrLD abolished the LLPS capability of LENG8, even at high PEG8000 concentrations (Figure 5A). These data demonstrate that the PrLD of LENG8 is sufficient to drive liquid droplet formation via LLPS in vitro.

We also purified the recombinant mCherry‐TIA1 protein. Consistent with previous studies [45, 46], TIA1 alone was capable of undergoing LLPS to form liquid‐like droplets in vitro (Figure S5H–K). Based on the established interaction between LENG8 and TIA1 mediated by the PrLD domain, we then asked whether this interaction could recruit LENG8 into TIA1 droplets. Our results showed that the EGFP‐LENG8 (1‐400 aa) was efficiently incorporated into TIA1 droplets (Figure 5G,H). Increasing concentrations of PEG8000 led to the formation of larger, coalesced droplets (Figure S5L). However, the ΔPrLD LENG8 variant (1‐400 aa) failed to localize to TIA1 droplets (Figure 5G,H). We further validated direct binding using an in vitro pull‐down assay. Recombinant LENG8 (1‐400 aa) interacted with TIA1, whereas PrLD deletion markedly reduced this complex formation (Figure 5I). These in vitro assays demonstrated that the PrLD of LENG8 not only facilitates the phase separation of LENG8 into liquid droplets, but also enables the fusion of two distinct droplets via subsequent PrLD‐mediated interaction with TIA1. This phenomenon is consistent with our intracellular observation that under stress conditions, LENG8 forms small foci in the cytoplasm and is then gradually recruited to TIA1/G3BP1‐positive early‐stage SGs.

Altogether, these findings indicate that PrLD‐driven phase separation and droplet fusion events recapitulate a key step of SG biogenesis in vitro, thereby highlighting the critical role of LENG8‐PrLD‐TIA1 interactions in orchestrating the assembly of early‐stage SGs.

### 2.6 LENG8 Mediates SG Assembly to Protect Thermosensitive Germ Cells

**FIGURE 6:**
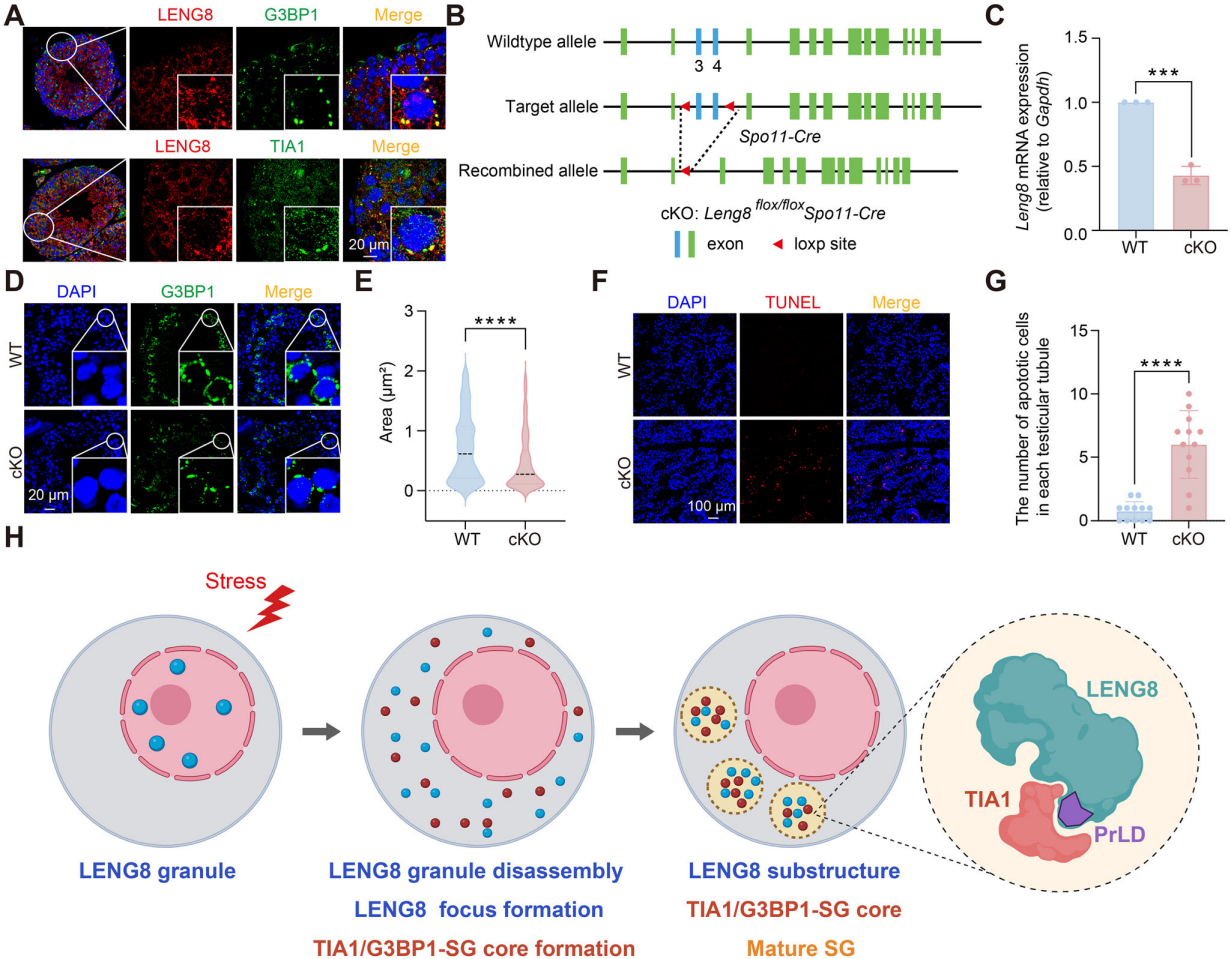
LENG8 promotes SG assembly to protect germ cells from heat stress. (A) Representative fluorescence images showing that LENG8 localizes to heat stress‐induced SGs in spermatocytes, with G3BP1 (top) and TIA1 (bottom) as SG markers, respectively. (B) Schematic diagram illustrating the construction of *Leng8* conditional knockout mice (cKO). Loxp sites were inserted between exons 3 and 4. Mice harboring the floxed allele were crossed with *Spo11‐Cre* transgenic mice to generate germ cell‐specific deletion of *Leng8*. (C) qPCR results show that *Leng8* cKO leads to a significant decrease in *Leng8* mRNA levels in the testes. Data are presented as mean ± SD, *n* = 3. ***p<0.001 by Student's *t*‐test. (D) Representative fluorescence images depicting the effects of *Leng8* cKO on the number and area of SGs in spermatocytes. (E) Quantitative analysis of the effect of *Leng8* cKO on the area of SGs in spermatocytes. The horizontal line inside each box indicates the median, *n* = 362, ****p<0.0001 by Student's *t*‐test. (F) TUNEL analysis of apoptotic germ cells in WT and *Leng8* cKO adult mouse testis sections. (G) Number of TUNEL‐positive cells per seminiferous tubule in WT and *Leng8* cKO adult mouse testes. Data are presented as mean ± SD, *n* = 12. ****p <0.0001 by Student's *t*‐test. (H) Model of LENG8‐mediated SG assembly. The model diagram was created with BioRender.com.

To sustain normal spermatogenesis, mammalian testes are maintained at a temperature 2–7°C lower than core body temperature and are thus exceptionally susceptible to heat stress [47]. Given this unique physiological feature, we explored whether LENG8 participates in the testicular stress response upon heat exposure. IF assays revealed that LENG8 localizes to the TIA1/G3BP1‐positive SGs formed in spermatocytes of wild‐type (WT) mouse testes following a 30 min heat treatment in a 43°C water bath (Figure 6A). In our previous study, we demonstrated that LENG8 is essential for spermatogenesis in mice [34]. Using conditional knockout (cKO) mice generated with the spermatogonia‐specific *Stra8‐Cre*, LENG8 deficiency caused a drastic loss of spermatocytes in male mice [34]. Since SG formation in testicular tissue in response to stress occurs primarily in spermatocytes, we generated *Leng8* cKO mice using *Spo11*‐*Cre* mice, where *Cre* recombinase is expressed in preleptotene/leptotene stage spermatocytes [48]. RT‑qPCR analyses revealed that *Leng8* mRNA levels were markedly downregulated in *Leng8* cKO male mice mediated by *Spo11‐Cre* (Figure 6B,C). Following heat stress, LENG8 ablation resulted in smaller SGs in testicular spermatocytes, recapitulating the phenotype observed in cultured somatic cells (Figure 6D,E). Accumulating evidence indicates that heat‐triggered SG assembly protects both germ cells and somatic cell lines from apoptotic cell death [49, 50]. Consistently, we found that defective SG formation upon LENG8 loss correlates with elevated apoptosis among spermatocytes (Figure 6F,G). Overall, these data suggest that LENG8 plays a conserved role in mediating SG assembly in mouse testes, thereby conferring protection to male germ cells against heat stress‐mediated damage by promoting SG formation.

## 3 Discussion

SGs are dynamic biomolecular condensates formed via LLPS in response to cellular stress, serving as critical regulators of mRNA metabolism and cellular stress adaptation. While the core SG nucleation network centered on G3BP1 has been well characterized, the mechanisms underlying the formation of SG nucleation seeds, the acquisition of SG heterogeneity, and the integration of distinct substructures during SG maturation remain poorly understood. In this study, we identified LENG8 as a novel SG nucleator that acts independently of the canonical G3BP1/TIA1 seed formation pathway, revealing a previously unrecognized layer of regulation in SG biogenesis. Our findings demonstrate that LENG8 undergoes stress‐induced nuclear‐cytoplasmic translocation, forms discrete liquid‐like foci via LLPS, and is recruited to maturing SGs through PrLD‐mediated interaction with TIA1, thereby promoting SG growth and maturation (Figure 6H). Furthermore, we validate the physiological significance of LENG8‐mediated SG assembly in protecting thermosensitive germ cells from heat stress‐induced apoptosis.

As we previously reported, LENG8 assembles into nuclear LLPS condensates (LENG8 granules) that mediate APA regulation, underscoring its involvement in nuclear RNA metabolism [34]. However, its potential involvement in cytoplasmic SG formation remained unknown. Our results show that LENG8 is specifically recruited to SGs under various stress conditions, including arsenite‐induced oxidative stress and heat stress, and this recruitment is tightly coupled to the disassembly of nuclear LENG8 granules. The spatiotemporal dynamics of LENG8, encompassing nuclear granule disassembly, cytoplasmic translocation, formation of discrete foci, and eventual incorporation into maturing SGs, closely mirror the stepwise assembly of SGs, suggesting that LENG8 is an integral participant in the SG biogenesis program. Notably, LENG8 translocation to SGs is conserved across cell types, indicating that its role in SG assembly is not cell‐type specific but rather a general cellular stress response mechanism. However, given the well‐established functions of LENG8 in nuclear RNA export, retention and RNA quality control, loss of LENG8 may disrupt mRNA subcellular localization and quality surveillance. Such defects would alter the pool of cytoplasmic untranslated mRNAs, thereby indirectly altering the assembly and size of stress granules. Accordingly, our current study cannot fully disentangle the distinct contributions of LENG8's direct function in regulating stress granule assembly and its broad roles in cellular RNA metabolism.

A key finding of this study is that LENG8 functions as a novel SG nucleator with distinct properties from canonical nucleators such as G3BP1 and TIA1. Unlike G3BP1, whose LLPS is triggered by the disruption of auto‐inhibition by free cytosolic RNA, LENG8 forms cytoplasmic foci via LLPS in an RNA‐independent manner, though its recruitment/localization to SGs requires intact SG structure. This independence from RNA for LLPS suggests that LENG8 may represent a unique class of SG nucleators that rely on protein‐protein interactions rather than RNA‐protein interactions to initiate condensate formation. Furthermore, while overexpression of G3BP1 or TIA1 is sufficient to induce SG formation under unstressed conditions, overexpression of full‐length LENG8 only forms nuclear granules without triggering cytoplasmic SGs. However, selective elevation of cytoplasmic LENG8 levels, either via NES‐tagged overexpression or stress‐induced translocation, induces both LENG8 foci formation and *de novo* SG assembly, confirming that cytoplasmic LENG8 is a functional SG nucleator. This distinction implies that the subcellular localization of LENG8 is a critical regulatory checkpoint for its SG‐nucleating activity, likely reflecting a mechanism to prevent aberrant SG formation under normal physiological conditions.

The N‐terminal PrLD of LENG8 emerges as a central mediator of its SG‐related functions. Our domain mapping experiments demonstrate that the PrLD is essential for LENG8 translocation to SGs, as deletion of this domain abolishes LENG8 recruitment to SGs and impairs its ability to rescue SG size defects in LENG8‐depleted cells. Optogenetic assays further confirm that the PrLD is sufficient to drive LLPS, consistent with the well‐established role of PrLDs in mediating phase separation of SG components [39]. Moreover, IP‐MS and Co‐IP analyses reveal that the PrLD mediates the stress‐enhanced interaction between LENG8 and TIA1, a core SG component critical for early SG assembly. Importantly, overexpression of tandem PrLD repeats disrupts the LENG8‐TIA1 interaction, blocks LENG8 recruitment to SGs, and leads to smaller SGs, mirroring the phenotype of LENG8 knockdown, confirming that the PrLD‐TIA1 interaction is indispensable for LENG8‐mediated SG maturation.

In vitro reconstitution experiments further delineate the molecular mechanisms underlying LENG8 function in SG assembly. Purified LENG8 (1‐400 aa) undergoes concentration‐dependent LLPS to form liquid‐like droplets, a process dependent on the PrLD and inhibited by high salt concentrations, consistent with the role of weak hydrophobic interactions in LLPS. Notably, LENG8 droplets can fuse with TIA1 droplets in vitro, recapitulating the in vivo observation of LENG8 foci merging with TIA1/G3BP1‐positive SG precursors. This fusion event is also PrLD‐dependent, as the ΔPrLD LENG8 variant fails to colocalize with TIA1 droplets. These results suggest a model in which LENG8 forms independent nucleation seeds via PrLD‐driven LLPS, and these seeds are then recruited to maturing SGs through PrLD‐TIA1 interactions, contributing to SG growth. This model aligns with the “cores‐first” model of SG assembly [13, 24, 25], where multiple distinct cores coalesce to form mature SGs, and identifies LENG8 foci as a previously unrecognized core type.

The physiological relevance of LENG8‐mediated SG assembly is underscored by our studies in *Leng8* cKO mice. Mammalian testes are highly sensitive to heat stress due to their lower optimal temperature, and SG formation is critical for protecting germ cells from stress‐induced apoptosis. We show that LENG8 localizes to SGs in spermatocytes of WT mice under heat stress, and depletion of LENG8 in spermatocytes impairs SG formation, leading to increased apoptotic germ cells. This finding extends our understanding of SG function in tissue‐specific stress responses and highlights LENG8 as a key protector of male fertility by mediating SG assembly in germ cells. Given our previous observation that LENG8 is essential for spermatogenesis, these results suggest that LENG8 may regulate germ cell survival through both nuclear APA regulation and cytoplasmic SG assembly, two distinct LLPS‐dependent mechanisms. Similarly, owing to LENG8's vital roles in RNA metabolism, the elevated spermatocyte apoptosis observed under heat stress upon LENG8 deficiency may also arise from disrupted RNA homeostasis. In future work, mutant mice that specifically disrupt the LENG8‐TIA1 interaction without impairing LENG8's RNA metabolic functions could be generated to dissect the independent role of LENG8 in stress granule assembly and spermatocyte survival under heat stress.

Our study also brings forward multiple outstanding research questions in SG biology. First, the mechanism underlying stress‐induced disassembly of nuclear LENG8 granules remains unclear. Since SG formation is coupled to the integrated stress response (ISR) pathway, and inhibition of ISR (via ISRIB) blocks LENG8 granule disassembly, it is possible that ISR‐related signals regulate the nuclear‐cytoplasmic shuttling of LENG8. Future studies will explore whether LENG8 is a direct target of ISR kinases or if its translocation is mediated by downstream ISR effectors or alternative post‐translational modifications. Second, immunofluorescence imaging reveals that LENG8 displays a punctate, heterogeneous distribution within mature stress granules. However, this observation alone cannot confirm that LENG8 assembles into discrete substructures inside SGs. Further experiments will therefore be necessary to rigorously validate whether these foci correspond to bona fide, physically segregated subcompartments and to characterize their relationship with other SG substructures (e.g., UBAP2L cores) [15, 21, 22, 23]. Third, the role of the C‐terminal region of LENG8 in LLPS and SG assembly remains incompletely understood. Our data suggest that the C‐terminal region cooperates with the PrLD to drive LENG8 LLPS, but the specific domains and interactions involved require further mapping. Fourth, our mass spectrometry interactome data reveal that LENG8 also interacts with other key node proteins within SGs, including FXR1, CAPRIN1 and UBAP2L. It remains to be further investigated whether, beyond mediating the fusion between early LENG8 foci and canonical G3BP1/TIA1 seeds via its interaction with TIA1, LENG8 also participates in building an interdependent regulatory network that coordinates the early assembly of stress granules.

In conclusion, our study identifies LENG8 as a novel SG nucleator that acts independently of the formation of the early G3BP1/TIA1 seeds, revealing a new layer of complexity in SG biogenesis. We demonstrate that LENG8's PrLD mediates both its LLPS and interaction with TIA1, facilitating the recruitment of LENG8 foci to maturing SGs and promoting SG growth. Furthermore, we establish a critical physiological role for LENG8 in protecting germ cells from heat stress via SG assembly. These findings not only advance our understanding of SG formation and heterogeneity but also highlight the multifunctional role of LENG8 in coordinating nuclear and cytoplasmic RNA metabolism through LLPS‐dependent condensates. Future studies will focus on elucidating the upstream regulators of LENG8 translocation and the molecular details of its interaction with TIA1, which may provide new therapeutic targets for diseases associated with aberrant SG formation or germ cell dysfunction.

## 4 Materials and Methods

### 4.1 Cell Culture

The HeLa (RRID: CVCL_0030), HEK293T (RRID: CVCL_0063), BEL 7402 (RRID: CVCL_5492), GC2 (RRID: CVCL_6633) and C2C12 (RRID: CVCL_0188) cells were cultured in DMEM medium (Thermo Fisher Scientific, C6126241) supplemented with 10% fetal bovine serum (TransGen Biotech, FS201) and 1% penicillin/streptomycin (Thermo Fisher Scientific, 15140122) at 37°C in a 5% CO_2_ humidified incubator. The culture medium was changed 24 h before stress conditions. The stressors included sodium arsenite (0.1, 0.5, or 1 mM), 0.1 mM puromycin, the mTOR pathway inhibitor Torin I (20 µM), the heat shock activator ML346 (0.1 M), the proteasome inhibitor MG132 (50 µM), the ferroptosis inducer RSL3 (50 µM), osmotic stress (0.2 M NaCl), the oxidative phosphorylation inhibitor oligomycin A (0.1 M), and the ER stress inducer thapsigargin (10 µM). Following treatment, cells were fixed with 4% paraformaldehyde (PFA, Sangon Biotech, E672002) for 15 min at room temperature for subsequent immunofluorescence analysis.

### 4.2 Animal Studies

Mice on a C57BL/6J (RRID: IMSR_JAX:000664) background were housed under specific pathogen‐free (SPF) conditions. Wild‐type male C57BL/6J mice aged 8 weeks with an average body weight of ∼24 g were purchased from the Shanghai Laboratory Animal Center (SLAC) and GemPharmatech Co., Ltd. (Nanjing, China). The C57BL/6J *Leng8*‐flox (*Leng8* ^flox/flox^) mouse strain (strain S‐CKO‐07581, Cyagen Biosciences) was constructed using CRISPR/Cas9 technology, with Loxp sites inserted into the introns flanking exons 3 and 4 of the *Leng8* gene. Germ cell‐specific deletion of *Leng8* was achieved by crossing *Leng8* ^flox/flox^ mice with the *Spo11‐Cre* transgenic line (Strain ID: 032646, Jackson laboratory, RRID: IMSR_JAX:032646). The *Cre* recombinase excises the floxed exons 3 and 4, causing a frameshift in the *Leng8* coding sequence and preventing the production of functional LENG8 protein. The genotypes of the mice were determined by PCR analysis. Male and female C57BL/6J mice utilized for mating and breeding were aged between 8 and 12 weeks.

### 4.3 Ethics Statement

The experimental procedures involving animals were approved by the Science and Technology Ethics Committee of Tongji University (TJAB06022103).

### 4.4 Plasmid Construction

Target gene sequences were cloned into the corresponding vectors (pCMV‐HA/Myc, pEGFP‐C1/N2, pCMV‐mCherry‐CRY2, pEGFP‐C1‐NES, pCold‐GST). The sequences of all primers are listed in Table S4. Gene‐specific amplification primers were designed with Primer software. Following amplification of the target fragments and linearization of the plasmid backbones, the products were ligated, transformed, and subsequently sent for commercial sequencing. Plasmid DNA was purified and verified by Sanger sequencing (Biosune).

### 4.5 Small Interfering RNA

The small interfering sequences were listed in the Table S4. All these siRNAs were obtained from Shanghai Generay Biotech Co, Ltd. siRNA and Lipofectamine 2000 (Thermo Fisher Scientific, 11668030) were separately diluted in Opti‐MEM medium (Thermo Fisher Scientific, 131985070). After 5 min incubation at room temperature, the solutions were combined and incubated for another 20 min to form complexes. The complexes were then added to HeLa cell cultures. At 48 h post‐transfection, cells were harvested for knockdown verification at both the protein and RNA levels by western blot and quantitative RT‐PCR, respectively.

### 4.6 Subcellular Fractionation

The fractionation of RNP granules was carried out essentially as previously described [37, 51]. Cells cultured in 10 cm dishes were placed on ice after sodium arsenite or heated treatment, washed twice with cold PBS and then subjected to lysis by adding 1 ml lysis buffer (50 mM Tris, pH7.0, 150 mM NaCl, 5 mM MgCl_2_, 1% Triton X‐100, 1×EDTA‐free protease inhibitor cocktail). After being lysed for 25 min at 4°C on a rotary mixer, whole cell lysates were centrifuged at 2000 g for 5 min to remove nuclei. The supernatants were collected for further RNP granule fractionation by centrifugation at 10 000 g for 10 min. The supernatants were collected as the soluble fraction, and the pellets were washed three times with lysis buffer for a total of 10 min before being harvested as the RG fraction. All the centrifugation procedures were conducted at 4°C. The subcellular fractions were then boiled in protein loading buffer for immunoblot analysis.

### 4.7 Immunoprecipitation Assays

Antibodies were conjugated to beads at 4°C. Following stress condition treatment, cells were placed on ice and lysed with lysis buffer for 20 min. The lysates were centrifuged at 8000 rpm for 10 min at 4°C. The resulting supernatants were added to the pre‐conjugated antibody‐bead complexes and incubated on a rotator for 4–6 h. Beads were then washed twice alternately with low‐salt buffer (50 mM Tris pH 7.0, 150 mM NaCl, 5 mM MgCl_2_, 0.1% Triton X‐100, 1× EDTA‐free protease inhibitor cocktail) and high‐salt buffer (50 mM Tris pH 7.0, 500 mM NaCl, 5 mM MgCl_2_, 0.1% Triton X‐100, 1× EDTA‐free protease inhibitor cocktail), followed by a final equilibration wash with lysis buffer. For downstream analyses, one portion of the beads was subjected to mass spectrometry, while the other portion was boiled in protein loading buffer for immunoblot analysis.

### 4.8 Western Blot

Protein samples were mixed with protein loading buffer and denatured by heating at 100°C for 10 min. Denatured samples were then loaded onto a 10% SDS‐polyacrylamide gel and electrophoresed at 100 V. Subsequently, proteins were transferred to PVDF membrane using wet transfer method at 4°C (100 V, 120 min). The membranes were blocked with blocking solution for 30 min (Beyotime, P0252), and incubated with diluted primary antibody (rabbit anti‐LENG8, Thermo Fisher, A304‐947A, 1:1000; rabbit anti‐LENG8, our lab, 1:1000; rabbit anti‐TIA1, Proteintech, 2133‐2‐AP, 1:1000, RRID: AB_2201427; rabbit anti‐G3BP1, Proteintech, 13057‐2‐AP, 1:1000, RRID: AB_2232034;; rabbit anti‐Myc, Abways, AB0001, 1:1000; rabbit anti‐GFP, Abways, AB0045, 1:1000; mouse anti‐HA, Proteintech, 66006‐2‐IG, 1:2000, RRID: AB_2881490; mouse anti‐GAPDH, Abclonal, AC054, 1:2000; anti‐β‐actin, Abways, AB2001, 1:5000; Beyotime, P0256) at 4°C overnight. After four washes with PBST buffer, membranes were incubated with corresponding secondary antibodies (HRP, Goat Anti‐Rabbit IgG, ABBKINE, A21020, 1:10000; HRP, Goat Anti‐Mouse IgG, ABBKINE, A21010, 1:10000; Beyotime, P0258). Following secondary antibody incubation, membranes were washed four times with PBST buffer. Protein bands were detected using ECL luminescent substrate (Tanon, 180–506) and visualized using a Tanon‑5200 Chemiluminescent Imaging System.

### 4.9 Immunofluorescence

Following different treatments, cells were fixed with 4% PFA for 15 min. Membranes were then blocked using a blocking solution for 30 min (Beyotime, P0260), followed by incubation with primary antibody (rabbit anti‐LENG8, Signalway, 39875, 1:200; rabbit anti‐LENG8, our lab, 1:100; mouse anti‐TIA1, Santa Cruz, sc‐166247, 1:100; mouse anti‐TIAR, Proteintech, 66907‐1‐Ig 1:200, RRID: AB_2882234; rabbit anti‐G3BP1, Proteintech, 13057‐2‐AP, 1:300; mouse anti‐G3BP1, Proteintech, 66486‐2‐AP, 1:500, RRID: AB_2232034; rabbit anti‐EIF2S1, Proteintech, 11170‐1‐AP, 1:300, RRID: AB_2096489; rabbit anti‐Phospho‐EIF2S1 (Ser51), Proteintech, 28740‐1‐AP, 1:300, RRID: AB_2881204; rabbit anti‐PABPC1, Proteintech, 10970‐1‐AP, 1:300, RRID: AB_10596918; mouse anti‐HUR, Invitrogen, 39–0600, 1:200, RRID: AB_2533394; mouse anti‐TOM20, Proteintech, 66777‐1‐Ig 1:200, RRID: AB_2882123; mouse anti‐LAMP2, Proteintech, 66301‐1‐Ig 1:200, RRID: AB_2881684; mouse anti‐DCP1A, Proteintech, 60494‐1‐Ig 1:200, RRID: AB_3670263; Beyotime, P0262) at 4°C overnight. The next day, samples were washed three times with PBS, followed by incubation with the corresponding secondary antibody (donkey anti‐rabbit‐594, Jackson, 711‐585‐152, 1:1000, RRID: AB_2340621; donkey anti‐mouse‐488, Jackson, 715‐545‐150, 1:1000, RRID: AB_2341099; donkey anti‐mouse‐red, Abbkine, A24411, 1:1000; Anti‐DAPI, Sigma, D9542, 1:2000; anti‐Hoechst3342, Beyotime, C1022, 1:2000; Beyotime, P0265), and subsequently washed three additional times. Apoptosis was detected using the One‐step TUNEL Apoptosis Detection Kit for Cells (Beyotime, C1090,) according to manufacturer's protocols. Finally, the samples were stained and mounted, and then analyzed using an Olympus SpinSR10 instrument under a 100× oil immersion objective.

### 4.10 Optogenetic System

We performed assays using the light‐inducible CRY2 system based on an established protocol, with specific adaptations for our experimental conditions [40]. Plasmids encoding the constructs for light‐induced protein aggregation (1–147 aa‐mCherry‐CRY2, 76–96 aa‐mCherry‐CRY2, and 73–147 aa‐mCherry‐CRY2) were first generated. These plasmids were then transfected into HeLa cells plated at 30% confluence on glass‐bottom dishes suitable for confocal microscopy. At 24 h post‐transfection, the dishes were transferred to an Olympus SpinSR10 super‐resolution confocal microscope system, which maintained a controlled environment for cell viability. Individual cells showing no spontaneous aggregation were selected for time‐lapse imaging. A 10 s blue‐light pulse was applied to induce aggregation, followed by image acquisition at 10 s intervals to monitor the dynamic process within the same cell over time.

### 4.11 qRT‐PCR

Total RNAs were extracted using the RNAiso Plus reagent (TaKaRa, # T9108) and subjected to reverse transcription for complementary DNA synthesis using the HiScript III RT SuperMix for qPCR (Vazyme, R323‐01), according to the manufacturer's instructions. mRNA abundance was determined using the UniPeak U+ One Step RT‐qPCR SYBR Green Kit (Vazyme, Q226‐01) on Light Cycler 96 System (Roche), with *Gapdh* or *β‐actin* used as the internal control for normalization.

### 4.12 Protein Expression and Purification

*LENG8^1‐400^* and *TIA1* were separately cloned into the pCold‐GST plasmid, and the recombinant plasmids were then transformed into *Escherichia coli* BL21 (DE3) cells (Tsingke, TSC‐E05). Protein expression was induced with 0.4 mM IPTG at 16°C for 12 h. Bacterial pellets were collected and lysed on ice at 4°C for 20 min, followed by ultrasonication (25% power, 2 s on/2 s off). The lysate was centrifuged at 12 000 rpm for 30 min at 4°C. The supernatant was incubated with GST beads for 2 h. The beads were washed five times with lysis buffer (50 mM Tris‐HCl, 500 mM NaCl, 1% Triton X‐100, 2 mM PMSF, 2 mM DTT, pH 8.0) for 30 min each. Recombinant proteins were eluted with elution buffer (50 mM Tris, 150 mM NaCl, 1% Triton X‐100, 30 mM GSH, 2 mM PMSF, 2 mM DTT, pH 8.0). Finally, the purified proteins were concentrated to a small volume for phase separation experiments.

### 4.13 GFP‐Splitting System

To investigate protein‐protein interactions, we employed a GFP complementation assay based on the GFP split‐system (GFP1‐10 and GFP11), as previously described with modifications [52]. Briefly, the cDNA sequences of GFP1‐10 and GFP11 were cloned into the pCMV‐HA‐TIA1 and pCMV‐Myc‐LENG8 vectors, respectively, resulting in the recombinant plasmids pCMV‐HA‐GFP1‐10‐TIA1 and pCMV‐Myc‐GFP11‐LENG8. HeLa cells were plated at 30% confluence and subsequently co‐transfected with the aforementioned recombinant plasmids. At 24 h post‐transfection, cells were treated with 0.5 mM sodium arsenite for 1 h to induce cellular stress. Following treatment, the culture medium was removed and the cells were washed once with PBS buffer. The cells were then fixed with 4% PFA for 15 min and subsequently washed three times with PBS buffer (5 min per wash). Finally, the samples were stained and mounted for observation using an Olympus SpinSR10 system under a 100× oil immersion objective.

### 4.14 Statistical Analysis

All statistical analyses were performed using GraphPad Prism (version 10.0). Data from at least three independent biological replicates are presented as mean ± SD, median. Each data point represents the mean of technical replicates from an individual biological experiment. Sample sizes (*n*) for each experiment are provided in the respective figure legends. For comparisons between two groups, a two‐tailed unpaired Student's *t*‐test was used. For multiple comparisons involving one control group, one‐way ANOVA was applied, while two‐way ANOVA was used for comparisons across multiple factors. Significance levels were defined as follows: *p* < 0.05 (*), *p* < 0.01 (**), *p* < 0.001 (***), and *p* <0.0001 (****); nonsignificant differences (*p* ≥ 0.05) are denoted as “ns”. Differentially expressed proteins (DEPs) between experimental and control groups (each with three biological replicates) were identified using the limma package [53], with a threshold of |fold change| > 1.4 and *p* < 0.05. Gene Ontology (GO) enrichment analysis of DEPs was conducted using the DAVID bioinformatics resource to annotate their associated biological processes and pathways [54, 55].

### 4.15 AI Disclosure

The authors acknowledge the use of the Doubao AI system for language proofreading and polishing of the manuscript text. All content generated or revised with assistance from this AI tool has been thoroughly reviewed, and verified by all authors, who assume full responsibility for the accuracy of the entire manuscript. No AI assisted data analysis, generation of scientific conclusions, or reference list compilation was performed in this work.

## Author Contributions

**M.X.Z**. established the model, designed and performed experiments, prepared figures, analyzed data and wrote the paper. **X.W**. and **Y.L.T**. analyzed data and provided resources for experiments. **Z.C.W**. performed mouse model construction. **J.F**. provided technical guidance for some experiments. **H.W.Z**. and **P.D**. supervised the project, acquired funding, and wrote, reviewed and edited the manuscript.

## Conflicts of Interest

The authors declare the following competing interests: A patent application covering the finding that tandem PrLD domains suppress stress granule assembly is currently pending (Application No. 202610917710.3). P.D. and M.‐X.Z. are named inventors on this application. The remaining authors declare no competing interests.

## Supporting information




**Supporting File 1**: advs77622‐sup‐0001‐SuppMat.docx.

**Supporting File 2**: advs77622‐sup‐0002‐TableS1.pdf.

**Supporting File 3**: advs77622‐sup‐0003‐TableS2.pdf.

**Supporting File 4**: advs77622‐sup‐0004‐TableS3.pdf.

**Supporting File 5**: advs77622‐sup‐0005‐TableS4.docx.

## Data Availability

All data are reported in the main text and supplementary materials, stored at Tongji University and available upon request.

## References

[1] N. Ripin and R. Parker , “Formation, Function, and Pathology of RNP Granules,” Cell 186 (2023): 4737–4756. 37890457 10.1016/j.cell.2023.09.006 PMC10617657

[2] J.‐Y. Youn , B. J. A. Dyakov , J. Zhang , et al., “Properties of Stress Granule and P‐Body Proteomes,” Molecular Cell 76 (2019): 286–294. 31626750 10.1016/j.molcel.2019.09.014

[3] C. L. Riggs , N. Kedersha , P. Ivanov , and P. Anderson , “Mammalian Stress Granules and P Bodies at a Glance,” Journal of Cell Science 133 (2020): 242487. 10.1242/jcs.242487 PMC10679417 32873715

[4] S. Markmiller , S. Soltanieh , K. L. Server , et al., “Context‐Dependent and Disease‐Specific Diversity in Protein Interactions within Stress Granules,” Cell 172 (2018): 590–604. 29373831 10.1016/j.cell.2017.12.032 PMC5969999

[5] J. Y. Youn , W. H. Dunham , S. J. Hong , et al., “High‐Density Proximity Mapping Reveals the Subcellular Organization of mRNA‐Associated Granules and Bodies,” Molecular Cell 69 (2018): 517–532. 29395067 10.1016/j.molcel.2017.12.020

[6] A. Padron , S. Iwasaki , and N. T. Ingolia , “Proximity RNA Labeling by APEX‐Seq Reveals the Organization of Translation Initiation Complexes and Repressive RNA Granules,” Molecular Cell 75 (2019): 875–887. 31442426 10.1016/j.molcel.2019.07.030 PMC6834362

[7] C. R. Pan , S. D. Knutson , S. W. Huth , and D. W. C. MacMillan , “µMap Proximity Labeling in Living Cells Reveals Stress Granule Disassembly Mechanisms,” Nature Chemical Biology 21 (2024): 490–500. 39215100 10.1038/s41589-024-01721-2 PMC11868469

[8] X. Sun , Y. Zhang , W. Lu , et al., “Precise and In Vivo‐Compatible Spatial Proteomics via Bioluminescence‐Triggered Photocatalytic Proximity Labeling,” ACS Central Science 11 (2025): 1611–1626. 41019118 10.1021/acscentsci.5c00520 PMC12464768

[9] M. Costa‐Mattioli and P. Walter , “The Integrated Stress Response: From Mechanism to Disease,” Science 368 (2020): aat5314. 10.1126/science.aat5314 PMC8997189 32327570

[10] N. L. Kedersha , M. Gupta , W. Li , I. Miller , and P. Anderson , “RNA‐Binding Proteins Tia‐1 and Tiar Link the Phosphorylation of Eif‐2α to the Assembly of Mammalian Stress Granules,” The Journal of Cell Biology 147 (1999): 1431–1442. 10613902 10.1083/jcb.147.7.1431 PMC2174242

[11] N. Kedersha , M. R. Cho , W. Li , et al., “Dynamic Shuttling of TIA‐1 Accompanies the Recruitment of mRNA to Mammalian Stress Granules,” The Journal of Cell Biology 151 (2000): 1257–1268. 11121440 10.1083/jcb.151.6.1257 PMC2190599

[12] H.‐S. Huang , L. Chen , J.‐X. Chi , et al., “Stress Granules and Cell Death: Crosstalk and Potential Therapeutic Strategies in Infectious Diseases,” Cell Death & Disease 16 (2025): 495. 40617807 10.1038/s41419-025-07800-z PMC12228806

[13] D. S. W. Protter and R. Parker , “Principles and Properties of Stress Granules,” Trends in Cell Biology 26 (2016): 668–679. 27289443 10.1016/j.tcb.2016.05.004 PMC4993645

[14] P. Yang , C. Mathieu , R.‐M. Kolaitis , et al., “G3BP1 Is a Tunable Switch that Triggers Phase Separation to Assemble Stress Granules,” Cell 181 (2020): 325–345. 32302571 10.1016/j.cell.2020.03.046 PMC7448383

[15] H. Glauninger and J. A. M. Bard , “Transcriptome‐Wide mRNP Condensation Precedes Stress Granule Formation and Excludes New mRNAs,” Molecular Cell 85 (2025): 4393–4409. 41289999 10.1016/j.molcel.2025.11.003 PMC12668231

[16] J. Guo , R. Huang , Y. Mei , et al., “Application of Stress Granule Core Element G3BP1 in Various Diseases: A Review,” International Journal of Biological Macromolecules 282 (2024): 137254. 39515684 10.1016/j.ijbiomac.2024.137254

[17] T. Wang , X. Tian , H. B. Kim , et al., “Intracellular Energy Controls Dynamics of Stress‐Induced Ribonucleoprotein Granules,” Nature Communications 13 (2022): 5584. 10.1038/s41467-022-33079-1 PMC9508253 36151083

[18] S. Hofmann , N. Kedersha , P. Anderson , and P. Ivanov , “Molecular Mechanisms of Stress Granule Assembly and Disassembly,” Biochimica et Biophysica Acta (BBA) ‐ Molecular Cell Research 1868 (2021): 118876. 33007331 10.1016/j.bbamcr.2020.118876 PMC7769147

[19] J. Guillén‐Boixet , A. Kopach , A. S. Holehouse , et al., “RNA‐Induced Conformational Switching and Clustering of G3BP Drive Stress Granule Assembly by Condensation,” Cell 181 (2020): 346–361. 32302572 10.1016/j.cell.2020.03.049 PMC7181197

[20] D. W. Sanders , N. Kedersha , D. S. W. Lee , et al., “Competing Protein‐RNA Interaction Networks Control Multiphase Intracellular Organization,” Cell 181 (Apr 16 2020): 306–324. 32302570 10.1016/j.cell.2020.03.050 PMC7816278

[21] L. Cirillo , A. Cieren , S. Barbieri , et al., “UBAP2L Forms Distinct Cores that Act in Nucleating Stress Granules Upstream of G3BP1,” Current Biology 30 (2020): 698–707. 31956030 10.1016/j.cub.2019.12.020

[22] S. Hu , Y. Zhang , Q. Yi , C. Yang , Y. Liu , and Y. Bai , “Time‐Resolved Proteomic Profiling Reveals Compositional and Functional Transitions Across the Stress Granule Life Cycle,” Nature Communications 14 (2023): 7782. 10.1038/s41467-023-43470-1 PMC10682001 38012130

[23] Y. Wu , X. Wang , L. Meng , et al., “Translation Landscape of Stress Granules,” Science Advances 11 (2025): ady6859. 10.1126/sciadv.ady6859 PMC12494010 41042876

[24] J. R. Wheeler , T. Matheny , S. Jain , R. Abrisch , and R. Parker , “Distinct Stages in Stress Granule Assembly and Disassembly,” Elife 5 (2016): 18413. 10.7554/eLife.18413 PMC5014549 27602576

[25] S. Jain , J. R. Wheeler , R. W. Walters , A. Agrawal , A. Barsic , and R. Parker , “ATPase‐Modulated Stress Granules Contain a Diverse Proteome and Substructure,” Cell 164 (2016): 487–498. 26777405 10.1016/j.cell.2015.12.038 PMC4733397

[26] T. Yoshizawa , R.‐S. Nozawa , T. Z. Jia , T. Saio , and E. Mori , “Biological Phase Separation: Cell Biology Meets Biophysics,” Biophysical Reviews 12 (2020): 519–539. 32189162 10.1007/s12551-020-00680-x PMC7242575

[27] N. A. Demeshkina and A. R. Ferré‐D'Amaré , “Large‐Scale Purifications Reveal Yeast and Human Stress Granule Cores are Heterogeneous Particles with Complex Transcriptomes and Proteomes,” Cell Reports 44 (2025): 115738. 40413746 10.1016/j.celrep.2025.115738 PMC12341040

[28] Q. Cui , Z. Liu , and G. Bai , “Friend or Foe: The Role of Stress Granule in Neurodegenerative Disease,” Neuron 112 (2024): 2464–2485. 38744273 10.1016/j.neuron.2024.04.025

[29] S. R. Millar , J. Q. Huang , K. J. Schreiber , et al., “A New Phase of Networking: The Molecular Composition and Regulatory Dynamics of Mammalian Stress Granules,” Chemical Reviews 123 (2023): 9036–9064. 36662637 10.1021/acs.chemrev.2c00608 PMC10375481

[30] L. Tian , L. Liu , Y. Yoon , et al., “LENG8 Mediates RNA Nuclear Retention and Degradation in Eukaryotes,” Molecular Cell 86 (2026): 1478–1494. 41861815 10.1016/j.molcel.2026.02.023 PMC13005043

[31] B. P. Clarke , S. Gao , M. Mei , et al., “Structural Mechanism of DDX39B Regulation by Human TREX‐2 and a Related Complex in mRNP Remodeling,” Nature Comminucations 16 (2025): 5471. 10.1038/s41467-025-60547-1 PMC12216326 40595470

[32] Y. Zhao , X. Wang , Y. Liu , et al., “LENG8 Regulation of mRNA Processing, is Responsible for the Control of Mitochondrial Activity,” bioRxiv, 10.1101/2021.07.17.452750.

[33] A. Bugai , U. Hohmann , A. Lorenzo , et al., “Molecular Basis of Polyadenylated RNA Fate Determination in the Nucleus,” Nature 655 (2026): 1070–1078. 42310446 10.1038/s41586-026-10650-0 PMC13391367

[34] Z.‐C. Wu , C. Du , X.‐Y. Huang , et al., “LENG8 Granule Regulates Alternative Polyadenylation in Mammals,” LangTaoSha Preprint Server (2026), 10.65215/LTSpreprints.2026.05.06.000176.

[35] H. Tourriere , K. Chebli , L. Zekri , et al., “The RasGAP‐Associated Endoribonuclease G3BP Mediates Stress Granule Assembly,” The Journal of Cell Biology 222 (2023): 20021212807. 10.1083/jcb.200212128072023new PMC10482220 37672657

[36] A. F. Zyryanova , K. Kashiwagi , C. Rato , et al., “ISRIB Blunts the Integrated Stress Response by Allosterically Antagonising the Inhibitory Effect of Phosphorylated eIF2 on eIF2B,” Molecular Cell 81 (2020): 88–103. 33220178 10.1016/j.molcel.2020.10.031 PMC7837216

[37] S. Liu , X. Zhang , X. Yao , et al., “Mammalian IRE1α Dynamically and Functionally Coalesces with Stress Granules,” Nature Cell Biology 26 (2024): 917–931. 38714852 10.1038/s41556-024-01418-7

[38] L. J. Bugaj , A. T. Choksi , C. K. Mesuda , R. S. Kane , and D. V. Schaffer , “Optogenetic Protein Clustering and Signaling Activation in Mammalian Cells,” Molecular & Cellular Oncology 10 (2013): 249–252. 10.1038/nmeth.2360 23377377

[39] T. Mittag and R. Parker , “Multiple Modes of Protein–Protein Interactions Promote RNP Granule Assembly,” Journal of Molecular Biology 430 (2018): 4636–4649. 30099026 10.1016/j.jmb.2018.08.005 PMC6204294

[40] Y. Shin , J. Berry , N. Pannucci , M. P. Haataja , J. E. Toettcher , and C. P. Brangwynne , “Spatiotemporal Control of Intracellular Phase Transitions Using Light‐Activated optoDroplets,” Cell 168 (2017): 159–171. 28041848 10.1016/j.cell.2016.11.054 PMC5562165

[41] P. Tan , L. He , Y. Huang , and Y. Zhou , “Optophysiology: Illuminating Cell Physiology with Optogenetics,” Physiological Reviews 102 (2022): 1263–1325. 35072525 10.1152/physrev.00021.2021 PMC8993538

[42] C. Bignon , A. Gruet , and S. Longhi , “Split‐GFP Reassembly Assay: Strengths and Caveats from a Multiparametric Analysis,” International Journal of Molecular Sciences 23 (2022): 13167. 36361946 10.3390/ijms232113167 PMC9658207

[43] N. Gilks , N. Kedersha , M. Ayodele , et al., “Stress Granule Assembly is Mediated by Prion‐Like Aggregation of TIA‐1,” Molecular Biology of the Cell 15 (2004): 5383–5398. 15371533 10.1091/mbc.E04-08-0715 PMC532018

[44] A. Fomicheva and E. D. Ross , “From Prions to Stress Granules: Defining the Compositional Features of Prion‐Like Domains That Promote Different Types of Assemblies,” International Journal of Molecular Sciences 22 (2021): 1251. 33513942 10.3390/ijms22031251 PMC7865556

[45] Y. Lin , D. S. Protter , M. K. Rosen , and R. Parker , “Formation and Maturation of Phase‐Separated Liquid Droplets by RNA‐Binding Proteins,” Molecular Cell 60 (2015): 208–219. 26412307 10.1016/j.molcel.2015.08.018 PMC4609299

[46] I. R. Mackenzie , A. M. Nicholson , M. Sarkar , et al., “TIA1 Mutations in Amyotrophic Lateral Sclerosis and Frontotemporal Dementia Promote Phase Separation and Alter Stress Granule Dynamics,” Neuron 95 (2017): 808–816. 28817800 10.1016/j.neuron.2017.07.025 PMC5576574

[47] R. A. Aldahhan and P. G. Stanton , “Heat Stress Response of Somatic Cells in the Testis,” Molecular and Cellular Endocrinology 527 (2021): 111216. 33639219 10.1016/j.mce.2021.111216

[48] S. R. Wellard , M. W. Skinner , X. Zhao , C. Shults , and P. W. Jordan , “PLK1 Depletion Alters Homologous Recombination and Synaptonemal Complex Disassembly Events during Mammalian Spermatogenesis,” Molecular Biology of the Cell 33 (2022): ar37. 35274968 10.1091/mbc.E21-03-0115 PMC9282006

[49] R.‐P. A. Chi , A. Gupta , Y. Li , et al., “The TENT5A‐ATXN2 Axis Modulates Germline and Somatic Cell Survival during Heat Stress,” Cell Death & Disease (2026), 10.1038/s41419-026-09086-1. PMC13646263 42443145

[50] F. Wang , L. Wang , S. Gan , et al., “SERBP1 Promotes Stress Granule Clearance by Regulating 26S Proteasome Activity and G3BP1 Ubiquitination and Protects Male Germ Cells from Thermostimuli Damage,” Research 6 (2023): 0091. 37223481 10.34133/research.0091 PMC10202183

[51] S. Namkoong , A. Ho , Y. M. Woo , H. Kwak , and J. H. Lee , “Systematic Characterization of Stress‐Induced RNA Granulation,” Molecular Cell 70 (2018): 175–187. 29576526 10.1016/j.molcel.2018.02.025 PMC6359928

[52] C. Lin , Y. Zhang , K. Zhang , et al., “Fever Promotes T Lymphocyte Trafficking via a Thermal Sensory Pathway Involving Heat Shock Protein 90 and α4 Integrins,” Immunity 50 (2019): 137–151. 30650373 10.1016/j.immuni.2018.11.013 PMC6432644

[53] M. E. Ritchie , B. Phipson , D. Wu , et al., “ *Limma* Powers Differential Expression Analyses for RNA‐Sequencing and Microarray Studies,” Nucleic Acids Research 43 (2015): 47. 10.1093/nar/gkv007 PMC4402510 25605792

[54] W. da Huang , B. T. Sherman , and R. A. Lempicki , “Systematic and Integrative Analysis of Large Gene Lists using DAVID Bioinformatics Resources,” Nature Protocols 4 (2009): 44–57. 19131956 10.1038/nprot.2008.211

[55] B. T. Sherman , M. Hao , J. Qiu , et al., “DAVID: A Web Server for Functional Enrichment Analysis and Functional Annotation of Gene Lists (2021 Update),” Nucleic Acids Research 50 (2022): W216–W221. 35325185 10.1093/nar/gkac194 PMC9252805

