# Supplementary material for: Prion‐Like Protein LENG8‐Mediated Nucleation Drives Stress Granule Assembly
Source: Adv Sci (Weinh). 2026 Sep 8:e77622. Online ahead of print. doi: 10.1002/advs.77622 (PMC13554387; doi:10.1002/advs.77622)
Supplement: Supplementary file 1 [file ADVS-9999-e77622-s001.pdf]

## Table S1. Differences in interacting proteins between Myc-LENG8 and Myc-LENG8$^{\Delta PrLD}$ mutant under arsenite condition.

**IP Intensity：Protein abundance was quantified using label-free protein intensity values obtained from MSFragger-based analysis.**

| Protein ID | Protein Names | Myc-LENG8$^{\Delta PrLD}$ (IP Intensity) | Myc-LENG8 (IP Intensity) | Description |
|---|---|---|---|---|
| O95831 | AIFM1 | 13948222 | 433524700 | Apoptosis-inducing factor 1, mitochondrial |
| P16402 | H1-3 | 1877414 | 51310096 | Histone H1.3 |
| Q86Y56 | DNAAF5 | 5855128 | 47192530 | Dynein axonemal assembly factor 5 |
| P42694 | HELZ | 3282280 | 24543480 | Probable helicase with zinc finger domain |
| P29558 | RBMS1 | 666445 | 4965324 | RNA-binding motif, single-stranded-interacting protein 1 |
| O43347 | MSI1 | 319131 | 2352891 | RNA-binding protein Musashi homolog 1 |
| Q9ULX3 | NOB1 | 24088204 | 171396860 | RNA-binding protein NOB1 |
| Q9UKJ3 | GPATCH8 | 5146717 | 31780028 | G patch domain-containing protein 8 |
| Q9UK61 | TASOR | 16368879 | 97618390 | Protein TASOR |
| Q9BYJ9 | YTHDF1 | 872723 | 4846406 | YTH domain-containing family protein 1 |
| P35251 | RFC1 | 2926125 | 16151629 | Replication factor C subunit 1 |
| Q12788 | TBL3 | 9671322 | 46226350 | Transducin beta-like protein 3 |
| Q6P2E9 | EDC4 | 5708619 | 27285414 | Enhancer of mRNA-decapping protein 4 |
| P46060 | RANGAP1 | 1615446 | 7605589 | Ran GTPase-activating protein 1 |
| Q5VWQ0 | RSBN1 | 3460212 | 15192310 | Lysine-specific demethylase 9 |
| Q92979 | EMG1 | 848401 | 3622882 | Ribosomal RNA small subunit methyltransferase NEP1 |
| Q96AG4 | LRRC59 | 4457975 | 18784266 | Leucine-rich repeat-containing protein 59 |
| O43298 | ZBTB43 | 2752857 | 11223286 | Zinc finger and BTB domain-containing protein 43 |
| P37108 | SRP14 | 1688075 | 6717116 | Signal recognition particle 14 kDa protein |
| P14618 | PKM | 915211 | 3426547 | Pyruvate kinase PKM |
| Q14498 | RBM39 | 48005972 | 171950340 | RNA-binding protein 39 |
| Q9NRW3 | APOBEC3C | 1368818 | 4758225 | DNA dC->dU-editing enzyme APOBEC-3C |
| Q13868 | EXOSC2 | 14070870 | 48346532 | Exosome complex component RRP4 |
| Q9UBB4 | ATXN10 | 10598076 | 32905304 | Ataxin-10 |
| P48634 | PRRC2A | 6856201 | 18616586 | Protein PRRC2A |
| Q5VU43 | PDE4DIP | 985878 | 2593372 | Myomegalin |
| P46778 | RPL21 | 91387250 | 234221070 | 60S ribosomal protein L21 |
| Q9BYG3 | NIFK | 1206896 | 3051987 | MKI67 FHA domain-interacting nucleolar phosphoprotein |
| Q01780 | EXOSC10 | 19823330 | 48876456 | Exosome component 10 |
| Q9NZL4 | HSPBP1 | 551501 | 1318749 | Hsp70-binding protein 1 |
| P52732 | KIF11 | 2918231 | 6908758 | Kinesin-like protein KIF11 |
| Q9BVI4 | NOC4L | 3164414 | 7373459 | Nucleolar complex protein 4 homolog |
| Q14137 | BOP1 | 1519947 | 3383071 | Ribosome biogenesis protein BOP1 |
| O95985 | TOP3B | 5346991 | 11804332 | DNA topoisomerase 3-beta-1 |
| Q9HCG8 | CWC22 | 7823173 | 17016896 | Pre-mRNA-splicing factor CWC22 homolog |
| Q9H0H5 | RACGAP1 | 1735478 | 3772485 | Rac GTPase-activating protein 1 |
| Q96T76 | MMS19 | 3485346 | 7551821 | MMS19 nucleotide excision repair protein homolog |
| P62277 | RPS13 | 27433332 | 59154824 | 40S ribosomal protein S13 |
| P19784 | CSNK2A2 | 6808568 | 14595916 | Casein kinase II subunit alpha' |
| Q9BY44 | EIF2A | 4861665 | 10154546 | Eukaryotic translation initiation factor 2A |
| P60842 | EIF4A1 | 13017777 | 27038606 | Eukaryotic initiation factor 4A-I |

| | | | | |
|---|---|---|---|---|
| P31483 | TIA1 | 9073610 | 18757830 | Nucleolysin TIAR |
| P19474 | TRIM21 | 8760442 | 16702351 | E3 ubiquitin-protein ligase TRIM21 |
| Q6NSI4 | RADX | 2970593 | 5644209 | RPA-related protein RADX |
| Q9UJX3 | ANAPC7 | 1324927 | 2487001 | Anaphase-promoting complex subunit 7 |
| O75400 | PRPF40A | 15791507 | 29546514 | Pre-mRNA-processing factor 40 homolog A |
| Q99575 | POP1 | 1410662 | 2637426 | Ribonucleases P/MRP protein subunit POP1 |
| Q5UIP0 | RIF1 | 13872747 | 25691808 | Telomere-associated protein RIF1 |
| Q3MHD2 | LSM12 | 3573358 | 6586201 | Protein LSM12 homolog |
| Q5QJE6 | DNTTIP2 | 1038919 | 1912141 | Deoxynucleotidyltransferase terminal-interacting protein 2 |
| P40938 | RFC3 | 6858332 | 12471593 | Replication factor C subunit 3 |
| Q9BQ70 | TCF25 | 4614937 | 8309029 | Transcription factor 25 |
| Q8IWA0 | WDR75 | 7583514 | 13343385 | WD repeat-containing protein 75 |
| Q5T8P6 | RBM26 | 1896864 | 3287796 | RNA-binding protein 26 |
| Q8TCG1 | CIP2A | 3609049 | 6179084 | Protein CIP2A |
| Q8IYB3 | SRRM1 | 48867800 | 82695736 | Serine/arginine repetitive matrix protein 1 |
| Q9GZR7 | DDX24 | 10122761 | 16762239 | ATP-dependent RNA helicase DDX24 |
| Q14839 | CHD4 | 14348583 | 23370192 | Chromodomain-helicase-DNA-binding protein 4 |
| P12235 | SLC25A4 | 3967231 | 6448546 | ADP/ATP translocase 1 |
| Q15388 | TOMM20 | 1961272 | 3160807 | Mitochondrial import receptor subunit TOM20 homolog |
| Q92688 | ANP32B | 6964615 | 11194188 | Acidic leucine-rich nuclear phosphoprotein 32 family member B |
| Q71RC2 | LARP4 | 5199975 | 8353373 | La-related protein 4 |
| P10515 | DLAT | 38698144 | 61877520 | Dihydrolipoyllysine-residue acetyltransferase component of pyruvate dehydrogenase complex, mitochondrial |
| Q13283 | G3BP1 | 3671738 | 5822783 | Ras GTPase-activating protein-binding protein 1 |
| Q9P2J5 | LARS1 | 10794734 | 16901998 | Leucine--tRNA ligase, cytoplasmic |
| Q9UKV3 | ACIN1 | 11188941 | 17474240 | Apoptotic chromatin condensation inducer in the nucleus |
| Q92552 | MRPS27 | 4866253 | 7547533 | 28S ribosomal protein S27, mitochondrial |
| P78346 | RPP30 | 2706449 | 4186757 | Ribonuclease P protein subunit p30 |
| Q9P0L0 | VAPA | 3858740 | 5962746 | Vesicle-associated membrane protein-associated protein A |
| Q6ZNB6 | NFXL1 | 6187499 | 9542210 | NF-X1-type zinc finger protein NFXL1 |
| Q9HAW4 | CLSPN | 26584432 | 40483670 | Claspin |
| O95639 | CPSF4 | 6233127 | 9405574 | Cleavage and polyadenylation specificity factor subunit 4 |
| P62851 | RPS25 | 165670600 | 249883940 | 40S ribosomal protein S25 |
| Q7Z739 | YTHDF3 | 3783896 | 5678239 | YTH domain-containing family protein 3 |
| Q96EV2 | RBM33 | 15965326 | 23918418 | RNA-binding protein 33 |
| Q99615 | DNAJC7 | 16596643 | 24831356 | DnaJ homolog subfamily C member 7 |
| O60762 | DPM1 | 1729169 | 2561521 | Dolichol-phosphate mannosyltransferase subunit 1 |
| P32969 | RPL9P9 | 76137210 | 112624160 | 60S ribosomal protein L9 |
| Q92733 | PRCC | 2922613 | 4297884 | Proline-rich protein PRCC |
| Q86VM9 | ZC3H18 | 13467140 | 19682964 | Zinc finger CCCH domain-containing protein 18 |
| Q9HCE1 | MOV10 | 23331160 | 33706028 | Helicase MOV-10 |

| | | | | |
|---|---|---|---|---|
| Q9UQE7 | SMC3 | 16314484 | 23426446 | Structural maintenance of chromosomes protein 3 |
| Q86V81 | ALYREF | 4461756 | 6303899 | THO complex subunit 4 |
| Q13595 | TRA2A | 10003491 | 14006352 | Transformer-2 protein homolog alpha |
| P13861 | PRKAR2A | 6985983 | 9711201 | cAMP-dependent protein kinase type II-alpha regulatory subunit |
| Q9UDY2 | TJP2 | 3199045 | 4398018 | Tight junction protein ZO-2 |
| P16989 | YBX3 | 34794664 | 47538896 | Y-box-binding protein 3 |
| Q9UI30 | TRMT112 | 14626267 | 19981986 | Multifunctional methyltransferase subunit TRM112-like protein |
| P06748 | NPM1 | 116628810 | 158286600 | Nucleophosmin |
| Q6PJT7 | ZC3H14 | 20861124 | 28018012 | Zinc finger CCCH domain-containing protein 14 |
| P33993 | MCM7 | 3064443 | 4093268 | DNA replication licensing factor MCM7 |
| Q29RF7 | PDS5A | 11126258 | 14776178 | Sister chromatid cohesion protein PDS5 homolog A |
| Q9Y2W1 | THRAP3 | 15399254 | 20318956 | Thyroid hormone receptor-associated protein 3 |
| Q7Z3K3 | POGZ | 5942312 | 7820364 | Pogo transposable element with ZNF domain |
| Q71U36 | TUBA1A | 101690000 | 133656350 | Tubulin alpha-1A chain |
| Q9NZI8 | IGF2BP1 | 52061224 | 67957544 | Insulin-like growth factor 2 mRNA-binding protein 1 |
| Q9Y5B9 | SUPT16H | 28199888 | 36663850 | FACT complex subunit SPT16 |
| P11387 | TOP1 | 26404282 | 34320444 | DNA topoisomerase 1 |
| Q9UPY3 | DICER1 | 2896050 | 3760053 | Endoribonuclease Dicer |
| Q9ULX6 | AKAP8L | 29084592 | 37748692 | A-kinase anchor protein 8-like |
| P02538 | KRT6A | 30081616 | 38992980 | Keratin, type II cytoskeletal 6A |
| P38646 | HSPA9 | 41047650 | 53177490 | Stress-70 protein, mitochondrial |
| Q8N9T8 | KRI1 | 15815854 | 20479236 | Protein KRI1 homolog |
| Q6DKI1 | RPL7L1 | 10195102 | 13154295 | 60S ribosomal protein L7-like 1 |
| P62424 | RPL7A | 239516300 | 308252800 | 60S ribosomal protein L7a |
| Q86U86 | PBRM1 | 1752885 | 2254479 | Protein polybromo-1 |
| Q8WWM7 | ATXN2L | 5035181 | 6463851 | Ataxin-2-like protein |
| Q9BXP5 | SRRT | 25651348 | 32739154 | Serrate RNA effector molecule homolog |
| P61326;Q96A72 | MAGOH;MAGOHB | 25984620 | 33154768 | Protein mago nashi homolog;Protein mago nashi homolog 2 |
| Q96KG9 | SCYL1 | 19130324 | 24375324 | N-terminal kinase-like protein |
| P62258 | YWHAE | 7207978 | 9160918 | 14-3-3 protein epsilon |
| Q14247 | CTTN | 78965550 | 99534776 | Src substrate cortactin |
| P62280 | RPS11 | 216555420 | 272876740 | 40S ribosomal protein S11 |
| Q14980 | NUMA1 | 4521989 | 5671723 | Nuclear mitotic apparatus protein 1 |
| Q02543 | RPL18A | 114676070 | 143083340 | 60S ribosomal protein L18a |
| P11940 | PABPC1 | 189261820 | 235439120 | Polyadenylate-binding protein 1 |
| O95163 | ELP1 | 2677689 | 3324241 | Elongator complex protein 1 |
| Q9BUF5 | TUBB6 | 6826955 | 8456829 | Tubulin beta-6 chain |
| Q14692 | BMS1 | 8233898 | 10178969 | Ribosome biogenesis protein BMS1 homolog |
| P82933 | MRPS9 | 512092 | 631811 | 28S ribosomal protein S9, mitochondrial |
| O43175 | PHGDH | 6137979 | 7534943 | D-3-phosphoglycerate dehydrogenase |
| Q15149 | PLEC | 9145213 | 11195419 | Plectin |
| P84098 | RPL19 | 271542100 | 332403040 | 60S ribosomal protein L19 |
| O75165 | DNAJC13 | 1102179 | 1339290 | DnaJ homolog subfamily C member 13 |
| Q00577 | PURA | 16572441 | 20133282 | Transcriptional activator protein Pur-alpha |
| Q15417 | CNN3 | 35880760 | 43583260 | Calponin-3 |
| Q12906 | ILF3 | 59597996 | 71724650 | Interleukin enhancer-binding factor 3 |

| | | | | |
|---|---|---|---|---|
| Q9NR30 | DDX21 | 134044584 | 160666880 | Nucleolar RNA helicase 2 |
| P46783 | RPS10 | 141002820 | 168523580 | 40S ribosomal protein S10 |
| Q9Y3X0 | CCDC9 | 2330064 | 2766814 | Coiled-coil domain-containing protein 9 |
| Q9Y5S9 | RBM8A | 41313116 | 48937730 | RNA-binding protein 8A |
| Q13427 | PPIG | 10339192 | 12194206 | Peptidyl-prolyl cis-trans isomerase G |
| Q15233 | NONO | 102976664 | 121225280 | Non-POU domain-containing octamer-binding protein |
| Q9Y6M1 | IGF2BP2 | 7932207 | 9337076 | Insulin-like growth factor 2 mRNA-binding protein 2 |
| P84090 | ERH | 65729572 | 77346600 | Enhancer of rudimentary homolog |
| Q12874 | SF3A3 | 11728202 | 13794329 | Splicing factor 3A subunit 3 |
| Q92900 | UPF1 | 19187994 | 22536300 | Regulator of nonsense transcripts 1 |
| Q14978 | NOLC1 | 56008530 | 65778260 | Nucleolar and coiled-body phosphoprotein 1 |
| P52756 | RBM5 | 13891473 | 16269056 | RNA-binding protein 5 |
| P01857;P01860 | IGHG1;IGHG3 | 1501747200 | 1753793000 | Immunoglobulin heavy constant gamma 1;Immunoglobulin heavy constant gamma 3 |
| P78332 | RBM6 | 7685721 | 8971771 | RNA-binding protein 6 |
| Q9NUD5 | ZCCHC3 | 17114368 | 19958542 | Zinc finger CCHC domain-containing protein 3 |
| O00425 | IGF2BP3 | 32747720 | 38124816 | Insulin-like growth factor 2 mRNA-binding protein 3 |
| O43852 | CALU | 11094695 | 12906296 | Calumenin |
| P27348 | YWHAQ | 1991475 | 2315528 | 14-3-3 protein theta |
| P42677 | RPS27 | 31292964 | 36223096 | 40S ribosomal protein S27 |
| Q9NUL3 | STAU2 | 16831688 | 19463338 | Double-stranded RNA-binding protein Staufen homolog 2 |
| Q14690 | PDCD11 | 5931085 | 6832681 | Protein RRP5 homolog |
| P78527 | PRKDC | 5365094 | 6154773 | DNA-dependent protein kinase catalytic subunit |
| P39023 | RPL3 | 236615360 | 271439500 | 60S ribosomal protein L3 |
| Q8IUE6 | H2AC21 | 3795305 | 4345454 | Histone H2A type 2-B |
| O43663 | PRC1 | 7576929 | 8669741 | Protein regulator of cytokinesis 1 |
| P68104;Q5VTE0 | EEF1A1;EEF1A1P5 | 39270864 | 44859028 | Elongation factor 1-alpha 1;Putative elongation factor 1-alpha-like 3 |
| O14646 | CHD1 | 9802480 | 11150846 | Chromodomain-helicase-DNA-binding protein 1 |
| Q15637 | SF1 | 3488109 | 3967263 | Splicing factor 1 |
| Q92841 | DDX17 | 28976334 | 32834392 | Probable ATP-dependent RNA helicase DDX17 |
| Q7Z2W4 | ZC3HAV1 | 10900730 | 12313193 | Zinc finger CCCH-type antiviral protein 1 |
| Q14566 | MCM6 | 12656755 | 14295583 | DNA replication licensing factor MCM6 |
| Q93009 | USP7 | 10786974 | 12150033 | Ubiquitin carboxyl-terminal hydrolase 7 |
| Q92499 | DDX1 | 50430200 | 56780764 | ATP-dependent RNA helicase DDX1 |
| Q9UJV9 | DDX41 | 26390846 | 29694538 | Probable ATP-dependent RNA helicase DDX41 |
| Q7Z417 | NUFIP2 | 5192607 | 5834981 | Nuclear fragile X mental retardation-interacting protein 2 |
| Q9H6S0 | YTHDC2 | 2784168 | 3127599 | 3'-5' RNA helicase YTHDC2 |
| Q8IU81 | IRF2BP1 | 1849314 | 2076915 | Interferon regulatory factor 2-binding protein 1 |
| Q9H7E2 | TDRD3 | 5291624 | 5932194 | Tudor domain-containing protein 3 |
| Q14683 | SMC1A | 43511560 | 48734756 | Structural maintenance of chromosomes protein 1A |

| | | | | |
|---|---|---|---|---|
| Q8IWR0 | ZC3H7A | 6935594 | 7754672 | Zinc finger CCCH domain-containing protein 7A |
| P62753 | RPS6 | 213701150 | 238926370 | 40S ribosomal protein S6 |
| Q5JTH9 | RRP12 | 5137224 | 5725880 | RRP12-like protein |
| Q9Y3F4 | STRAP | 20306608 | 22611028 | Serine-threonine kinase receptor-associated protein |
| P62249 | RPS16 | 122707900 | 136444290 | 40S ribosomal protein S16 |
| P55795 | HNRNPH2 | 16123414 | 17828426 | Heterogeneous nuclear ribonucleoprotein H2 |
| P08238 | HSP90AB1 | 28302140 | 31244788 | Heat shock protein HSP 90-beta |
| Q92973 | TNPO1 | 2574835 | 2835726 | Transportin-1 |
| P40429 | RPL13A | 120531550 | 132589820 | 60S ribosomal protein L13a |
| O00267 | SUPT5H | 16419192 | 18006312 | Transcription elongation factor SPT5 |
| Q9HAN9 | NMNAT1 | 7782672 | 8530722 | Nicotinamide/nicotinic acid mononucleotide adenylyltransferase 1 |
| P46779 | RPL28 | 148447090 | 162604270 | 60S ribosomal protein L28 |
| Q6UXN9 | WDR82 | 17200488 | 18703906 | WD repeat-containing protein 82 |
| Q99700 | ATXN2 | 3633800 | 3950410 | Ataxin-2 |
| Q9H307 | PNN | 29039024 | 31567180 | Pinin |
| P31942 | HNRNPH3 | 18921798 | 20494252 | Heterogeneous nuclear ribonucleoprotein H3 |
| Q9H6R4 | NOL6 | 1796990 | 1945844 | Nucleolar protein 6 |
| P31943 | HNRNPH1 | 129380250 | 140092160 | Heterogeneous nuclear ribonucleoprotein H |
| P62854 | RPS26 | 135743780 | 146692270 | 40S ribosomal protein S26 |
| Q9Y450 | HBS1L | 28253116 | 30432030 | HBS1-like protein |
| Q15459 | SF3A1 | 7357895 | 7918017 | Splicing factor 3A subunit 1 |
| O60814 | H2BC12 | 51610892 | 55475420 | Histone H2B type 1-K |
| P78347 | GTF2I | 64041690 | 68721840 | General transcription factor II-I |
| Q8NCA5 | FAM98A | 28371008 | 30289782 | Protein FAM98A |
| P62910 | RPL32 | 293154300 | 312634660 | 60S ribosomal protein L32 |
| Q9H583 | HEATR1 | 3354152 | 3570375 | HEAT repeat-containing protein 1 |
| Q92616 | GCN1 | 5353064 | 5692004 | eIF-2-alpha kinase activator GCN1 |
| Q6PKG0 | LARP1 | 64179290 | 68157840 | La-related protein 1 |
| P07437 | TUBB | 130881290 | 138990610 | Tubulin beta chain |
| Q9BV38 | WDR18 | 39696390 | 42151656 | WD repeat-containing protein 18 |
| P39019 | RPS19 | 141622350 | 150362180 | 40S ribosomal protein S19 |
| Q9Y5X1 | SNX9 | 148165500 | 157020580 | Sorting nexin-9 |
| O15234 | CASC3 | 22292116 | 23547416 | Protein CASC3 |
| Q13148 | TARDBP | 32312124 | 34076190 | TAR DNA-binding protein 43 |
| P61247 | RPS3A | 149678660 | 157525200 | 40S ribosomal protein S3a |
| Q8NC51 | SERBP1 | 130608550 | 137270050 | Plasminogen activator inhibitor 1 RNA-binding protein |
| P46777 | RPL5 | 170311780 | 178959800 | 60S ribosomal protein L5 |
| O95714 | HERC2 | 7084719 | 7434832 | E3 ubiquitin-protein ligase HERC2 |
| P62269 | RPS18 | 216829380 | 227384220 | 40S ribosomal protein S18 |
| P62263 | RPS14 | 122466770 | 128389830 | 40S ribosomal protein S14 |
| P52272 | HNRNPM | 185026430 | 193834510 | Heterogeneous nuclear ribonucleoprotein M |
| P04908 | H2AC8 | 409529100 | 428602600 | Histone H2A type 1-B/E |
| Q71UM5 | RPS27L | 83927336 | 87714904 | 40S ribosomal protein S27-like |
| Q9UNX3 | RPL26L1 | 221835820 | 231659330 | 60S ribosomal protein L26-like 1 |
| P46013 | MKI67 | 13418160 | 13975875 | Proliferation marker protein Ki-67 |
| P62917 | RPL8 | 172616820 | 179735940 | 60S ribosomal protein L8 |
| Q6P158 | DHX57 | 13931440 | 14500028 | Putative ATP-dependent RNA helicase DHX57 |

| | | | | |
|---|---|---|---|---|
| P49711 | CTCF | 8255113 | 8590774 | Transcriptional repressor CTCF |
| Q8WUA2 | PPIL4 | 63037700 | 65503828 | Peptidyl-prolyl cis-trans isomerase-like 4 |
| Q01804 | OTUD4 | 1820722 | 1886759 | OTU domain-containing protein 4 |
| Q04837 | SSBP1 | 745520830 | 772282900 | Single-stranded DNA-binding protein, mitochondrial |
| Q9Y2X3 | NOP58 | 10653611 | 11005034 | Nucleolar protein 58 |
| O00422 | SAP18 | 39007984 | 40277864 | Histone deacetylase complex subunit SAP18 |
| P35268 | RPL22 | 103483910 | 106799680 | 60S ribosomal protein L22 |
| P50402 | EMD | 52684770 | 54342040 | Emerin |
| Q9Y5A9 | YTHDF2 | 14075242 | 14506476 | YTH domain-containing family protein 2 |
| Q9BQ39 | DDX50 | 59632268 | 61421820 | ATP-dependent RNA helicase DDX50 |
| P42766 | RPL35 | 200519540 | 206482820 | 60S ribosomal protein L35 |
| Q00610 | CLTC | 4619173 | 4755314 | Clathrin heavy chain 1 |
| Q6P2Q9 | PRPF8 | 17590132 | 18076782 | Pre-mRNA-processing-splicing factor 8 |
| P62081 | RPS7 | 252057500 | 258815660 | 40S ribosomal protein S7 |
| P18077 | RPL35A | 206412530 | 211230750 | 60S ribosomal protein L35a |
| O15372 | EIF3H | 4824146 | 4935303 | Eukaryotic translation initiation factor 3 subunit H |
| P62857 | RPS28 | 42904690 | 43816056 | 40S ribosomal protein S28 |
| Q9UKF6 | CPSF3 | 13008485 | 13276400 | Cleavage and polyadenylation specificity factor subunit 3 |
| Q07021 | C1QBP | 2608042 | 2660386 | Complement component 1 Q subcomponent-binding protein, mitochondrial |
| Q92879 | CELF1 | 5718993 | 5833767 | CUGBP Elav-like family member 1 |
| Q9Y3U8 | RPL36 | 130114610 | 132585620 | 60S ribosomal protein L36 |
| O95793 | STAU1 | 31898388 | 32502544 | Double-stranded RNA-binding protein Staufen homolog 1 |
| Q8N5F7 | NKAP | 4886012 | 4957510 | NF-kappa-B-activating protein |
| O14893 | GEMIN2 | 3008069 | 3044382 | Gem-associated protein 2 |
| Q9P2I0 | CPSF2 | 18271118 | 18484738 | Cleavage and polyadenylation specificity factor subunit 2 |
| P67809 | YBX1 | 18369990 | 18536880 | Y-box-binding protein 1 |
| P52294 | KPNA1 | 7737510 | 7807769 | Importin subunit alpha-5 |
| A5YKK6 | CNOT1 | 3910374 | 3941652 | CCR4-NOT transcription complex subunit 1 |
| Q9NW13 | RBM28 | 35081956 | 35316524 | RNA-binding protein 28 |
| P61513 | RPL37A | 87469910 | 87971640 | 60S ribosomal protein L37a |
| P08708 | RPS17 | 56990396 | 57294436 | 40S ribosomal protein S17 |
| P36578 | RPL4 | 305530900 | 307014560 | 60S ribosomal protein L4 |
| P62701 | RPS4X | 325009800 | 326023070 | 40S ribosomal protein S4, X isoform |
| P62750 | RPL23A | 220573220 | 221173760 | 60S ribosomal protein L23a |
| Q15287 | RNPS1 | 52973576 | 53042644 | RNA-binding protein with serine-rich domain 1 |
| Q9UQ35 | SRRM2 | 26643260 | 26653836 | Serine/arginine repetitive matrix protein 2 |
| Q99569 | PKP4 | 2868359 | 2865405 | Plakophilin-4 |
| P78362 | SRPK2 | 25792848 | 25755178 | SRSF protein kinase 2 |
| Q9UNE7 | STUB1 | 29934228 | 29863196 | E3 ubiquitin-protein ligase CHIP |
| Q6P5R6 | RPL22L1 | 38063890 | 37945532 | 60S ribosomal protein L22-like 1 |
| P46087 | NOP2 | 36162012 | 35983732 | Probable 28S rRNA (cytosine(4447)-C(5))-methyltransferase |
| O00541 | PES1 | 11244344 | 11176302 | Pescadillo homolog |
| P63104 | YWHAZ | 14466103 | 14374716 | 14-3-3 protein zeta/delta |
| Q9P2E9 | RRBP1 | 9022867 | 8960068 | Ribosome-binding protein 1 |

| | | | | |
|---|---|---|---|---|
| O60832 | DKC1 | 20143128 | 19975344 | H/ACA ribonucleoprotein complex subunit DKC1 |
| Q9Y4X5 | ARIH1 | 8293417 | 8220945 | E3 ubiquitin-protein ligase ARIH1 |
| P09661 | SNRPA1 | 16725588 | 16576939 | U2 small nuclear ribonucleoprotein A' |
| P23246 | SFPQ | 17720786 | 17549182 | Splicing factor, proline- and glutamine-rich |
| Q13435 | SF3B2 | 17146766 | 16950890 | Splicing factor 3B subunit 2 |
| O14654 | IRS4 | 6782697 | 6701438 | Insulin receptor substrate 4 |
| P60709 | ACTB | 78076500 | 77119990 | Actin, cytoplasmic 1 |
| P17480 | UBTF | 22619598 | 22307278 | Nucleolar transcription factor 1 |
| Q09161 | NCBP1 | 4392780 | 4330299 | Nuclear cap-binding protein subunit 1 |
| Q14157 | UBAP2L | 14346801 | 14125398 | Ubiquitin-associated protein 2-like |
| Q07020 | RPL18 | 336153570 | 330465340 | 60S ribosomal protein L18 |
| Q14671 | PUM1 | 23933046 | 23527194 | Pumilio homolog 1 |
| Q96PK6 | RBM14 | 53610730 | 52694560 | RNA-binding protein 14 |
| P62993 | GRB2 | 13997733 | 13741021 | Growth factor receptor-bound protein 2 |
| P62714;P67775 | PPP2CB;PPP2CA | 4579874 | 4494094 | Serine/threonine-protein phosphatase 2A catalytic subunit beta isoform;Serine/threonine-protein phosphatase 2A catalytic subunit alpha isoform |
| Q96S55 | WRNIP1 | 89758470 | 88029850 | ATPase WRNIP1 |
| O60216 | RAD21 | 24625476 | 24137218 | Double-strand-break repair protein rad21 homolog |
| P16403 | H1-2 | 253511100 | 248436770 | Histone H1.2 |
| P67870 | CSNK2B | 10954488 | 10734587 | Casein kinase II subunit beta |
| Q15424 | SAFB | 19482496 | 19057096 | Scaffold attachment factor B1 |
| O60573 | EIF4E2 | 1395306 | 1363025 | Eukaryotic translation initiation factor 4E type 2 |
| O43719 | HTATSF1 | 82673020 | 80755940 | HIV Tat-specific factor 1 |
| Q8N3C0 | ASCC3 | 12023238 | 11737456 | Activating signal cointegrator 1 complex subunit 3 |
| Q1KMD3 | HNRNPUL2 | 26297258 | 25660474 | Heterogeneous nuclear ribonucleoprotein U-like protein 2 |
| Q9BPZ3 | PAIP2 | 3148181 | 3069408 | Polyadenylate-binding protein-interacting protein 2 |
| Q9BWF3 | RBM4 | 14792058 | 14386016 | RNA-binding protein 4 |
| P13645 | KRT10 | 86863624 | 84443290 | Keratin, type I cytoskeletal 10 |
| Q8TEX9 | IPO4 | 12373973 | 11961231 | Importin-4 |
| P62979 | RPS27A | 90859010 | 87817890 | Ubiquitin-40S ribosomal protein S27a |
| Q9HC44 | GPBP1L1 | 8177865 | 7882827 | Vasculin-like protein 1 |
| O75821 | EIF3G | 14871864 | 14304061 | Eukaryotic translation initiation factor 3 subunit G |
| Q969Q0 | RPL36AL | 119039830 | 114451380 | 60S ribosomal protein L36a-like |
| Q8N3U4 | STAG2 | 12555743 | 12059957 | Cohesin subunit SA-2 |
| O76021 | RSL1D1 | 60433804 | 58013588 | Ribosomal L1 domain-containing protein 1 |
| P07910 | HNRNPC | 260736100 | 249984780 | Heterogeneous nuclear ribonucleoproteins C1/C2 |
| Q10570 | CPSF1 | 12229358 | 11717128 | Cleavage and polyadenylation specificity factor subunit 1 |
| Q15185 | PTGES3 | 81416184 | 77987350 | Prostaglandin E synthase 3 |
| Q96GA3 | LTV1 | 12873165 | 12328712 | Protein LTV1 homolog |
| P38919 | EIF4A3 | 79176080 | 75648740 | Eukaryotic initiation factor 4A-III |
| P51970 | NDUFA8 | 2124652 | 2028998 | NADH dehydrogenase [ubiquinone] 1 alpha subcomplex subunit 8 |

| | | | | |
|---|---|---|---|---|
| Q7L014 | DDX46 | 3653351 | 3484615 | Probable ATP-dependent RNA helicase DDX46 |
| Q06830 | PRDX1 | 39790270 | 37836000 | Peroxiredoxin-1 |
| P63244 | RACK1 | 117039780 | 111091784 | Receptor of activated protein C kinase 1 |
| Q7KZF4 | SND1 | 3041579 | 2880750 | Staphylococcal nuclease domain-containing protein 1 |
| Q13547 | HDAC1 | 15118048 | 14290737 | Histone deacetylase 1 |
| P62241 | RPS8 | 166691760 | 157441260 | 40S ribosomal protein S8 |
| Q5SW79 | CEP170 | 5191251 | 4897551 | Centrosomal protein of 170 kDa |
| Q9NVP1 | DDX18 | 11910059 | 11234735 | ATP-dependent RNA helicase DDX18 |
| O60232 | ZNRD2 | 7409461 | 6974535 | Protein ZNRD2 |
| O00410 | IPO5 | 1849572 | 1740791 | Importin-5 |
| O00303 | EIF3F | 6509302 | 6118846 | Eukaryotic translation initiation factor 3 subunit F |
| O75175 | CNOT3 | 1643565 | 1544010 | CCR4-NOT transcription complex subunit 3 |
| P51114 | FXR1 | 18911666 | 17757542 | Fragile X mental retardation syndrome-related protein 1 |
| Q04637 | EIF4G1 | 8139476 | 7640886 | Eukaryotic translation initiation factor 4 gamma 1 |
| P22087 | FBL | 39270080 | 36849276 | rRNA 2'-O-methyltransferase fibrillarin |
| O00571 | DDX3X | 126728650 | 118546540 | ATP-dependent RNA helicase DDX3X |
| P43487 | RANBP1 | 15863507 | 14799359 | Ran-specific GTPase-activating protein |
| Q9BYN8 | MRPS26 | 8180867 | 7629909 | 28S ribosomal protein S26, mitochondrial |
| P05388 | RPLP0 | 156887710 | 146263760 | 60S acidic ribosomal protein P0 |
| Q9P2N5 | RBM27 | 2590137 | 2413162 | RNA-binding protein 27 |
| P15924 | DSP | 8618469 | 8027160 | Desmoplakin |
| P18124 | RPL7 | 350319900 | 326012770 | 60S ribosomal protein L7 |
| Q7L2E3 | DHX30 | 86069700 | 80037900 | ATP-dependent RNA helicase DHX30 |
| Q15029 | EFTUD2 | 39776284 | 36983490 | 116 kDa U5 small nuclear ribonucleoprotein component |
| O94776 | MTA2 | 9942741 | 9233837 | Metastasis-associated protein MTA2 |
| Q9UPT8 | ZC3H4 | 12273838 | 11396141 | Zinc finger CCCH domain-containing protein 4 |
| P49916 | LIG3 | 14186098 | 13170829 | DNA ligase 3 |
| Q02878 | RPL6 | 387398460 | 359316260 | 60S ribosomal protein L6 |
| P61313 | RPL15 | 354570600 | 328678080 | 60S ribosomal protein L15 |
| Q5T3I0 | GPATCH4 | 8231525 | 7605096 | G patch domain-containing protein 4 |
| Q96HS1 | PGAM5 | 189712640 | 175268940 | Serine/threonine-protein phosphatase PGAM5, mitochondrial |
| Q9Y3T9 | NOC2L | 4228869 | 3903368 | Nucleolar complex protein 2 homolog |
| P08670 | VIM | 452390750 | 417207230 | Vimentin |
| Q9Y3I0 | RTCB | 22281566 | 20541412 | RNA-splicing ligase RtcB homolog |
| P14866 | HNRNPL | 137396200 | 126343430 | Heterogeneous nuclear ribonucleoprotein L |
| Q13263 | TRIM28 | 60909536 | 55927196 | Transcription intermediary factor 1-beta |
| Q8NI27 | THOC2 | 13760475 | 12623809 | THO complex subunit 2 |
| Q6UN15 | FIP1L1 | 9241947 | 8477917 | Pre-mRNA 3'-end-processing factor FIP1 |
| Q02978 | SLC25A11 | 5017573 | 4599766 | Mitochondrial 2-oxoglutarate/malate carrier protein |
| P22061 | PCMT1 | 21450054 | 19631336 | Protein-L-isoaspartate(D-aspartate) O-methyltransferase |
| A0A0B4J2E5 | LOC102724159;PWP2 | 5065134 | 4625761 | Periodic tryptophan protein 2 homolog |
| P25398 | RPS12 | 173075090 | 158055230 | 40S ribosomal protein S12 |

| | | | | |
|---|---|---|---|---|
| P55769 | SNU13 | 14902271 | 13584057 | NHP2-like protein 1 |
| Q07157 | TJP1 | 16781758 | 15275513 | Tight junction protein ZO-1 |
| Q8N684 | CPSF7 | 20024246 | 18214350 | Cleavage and polyadenylation specificity factor subunit 7 |
| P61353 | RPL27 | 211079420 | 191643100 | 60S ribosomal protein L27 |
| O15381 | NVL | 34084980 | 30938540 | Nuclear valosin-containing protein-like |
| P63220 | RPS21 | 99305570 | 89966320 | 40S ribosomal protein S21 |
| P10599 | TXN | 37766824 | 34142028 | Thioredoxin |
| Q9NW75 | GPATCH2 | 8974479 | 8104752 | G patch domain-containing protein 2 |
| P42166 | TMPO | 24946882 | 22526588 | Lamina-associated polypeptide 2, isoform alpha |
| Q07666 | KHDRBS1 | 4507372500 | 4068161500 | KH domain-containing, RNA-binding, signal transduction-associated protein 1 |
| P54105 | CLNS1A | 3711504 | 3347866 | Methylosome subunit pICln |
| Q9NU22 | MDN1 | 6129957 | 5528594 | Midasin |
| Q9BUJ2 | HNRNPUL1 | 58795656 | 52952628 | Heterogeneous nuclear ribonucleoprotein U-like protein 1 |
| Q08945 | SSRP1 | 15326572 | 13790630 | FACT complex subunit SSRP1 |
| Q16629 | SRSF7 | 132863100 | 119204530 | Serine/arginine-rich splicing factor 7 |
| Q92522 | H1-10 | 27928266 | 25039740 | Histone H1.10 |
| P40939 | HADHA | 5610853 | 5028749 | Trifunctional enzyme subunit alpha, mitochondrial |
| Q02880 | TOP2B | 143182800 | 128271870 | DNA topoisomerase 2-beta |
| P68371 | TUBB4B | 424179680 | 379590080 | Tubulin beta-4B chain |
| P27635 | RPL10 | 237712000 | 212667230 | 60S ribosomal protein L10 |
| P26368 | U2AF2 | 22028844 | 19699818 | Splicing factor U2AF 65 kDa subunit |
| Q14257 | RCN2 | 106655440 | 95187416 | Reticulocalbin-2 |
| P51116 | FXR2 | 59334520 | 52901670 | Fragile X mental retardation syndrome-related protein 2 |
| P62273 | RPS29 | 96487600 | 86007770 | 40S ribosomal protein S29 |
| P05141 | SLC25A5 | 122802050 | 109445560 | ADP/ATP translocase 2 |
| P08865 | RPSA | 236495060 | 210403780 | 40S ribosomal protein SA |
| P42285 | MTREX | 77751860 | 69101496 | Exosome RNA helicase MTR4 |
| P18621 | RPL17 | 154362100 | 137171090 | 60S ribosomal protein L17 |
| Q96CT7 | CCDC124 | 11383519 | 10102336 | Coiled-coil domain-containing protein 124 |
| O00567 | NOP56 | 11176227 | 9901990 | Nucleolar protein 56 |
| O60264 | SMARCA5 | 17245014 | 15263936 | SWI/SNF-related matrix-associated actin-dependent regulator of chromatin subfamily A member 5 |
| P62888 | RPL30 | 99903990 | 88417910 | 60S ribosomal protein L30 |
| O75534 | CSDE1 | 28887878 | 25459488 | Cold shock domain-containing protein E1 |
| P05387 | RPLP2 | 177465460 | 156162080 | 60S acidic ribosomal protein P2 |
| P55265 | ADAR | 32225126 | 28338294 | Double-stranded RNA-specific adenosine deaminase |
| Q9Y2Z0 | SUGT1 | 9810262 | 8625469 | Protein SGT1 homolog |
| Q06787 | FMR1 | 34827730 | 30610958 | Synaptic functional regulator FMR1 |
| P43243 | MATR3 | 76854890 | 67388100 | Matrin-3 |
| O00148 | DDX39A | 16035877 | 13996049 | ATP-dependent RNA helicase DDX39A |
| P68431 | H3C12 | 256702600 | 223978560 | Histone H3.1 |
| O43390 | HNRNPR | 21867684 | 19072752 | Heterogeneous nuclear ribonucleoprotein R |
| P62913 | RPL11 | 344681020 | 300518340 | 60S ribosomal protein L11 |
| P46781 | RPS9 | 151774980 | 131928700 | 40S ribosomal protein S9 |
| P63241 | EIF5A | 26835420 | 23299060 | Eukaryotic translation initiation factor 5A-1 |

| | | | | |
|---|---|---|---|---|
| Q8IWX8 | CHERP | 62451972 | 54221492 | Calcium homeostasis endoplasmic reticulum protein |
| Q00059 | TFAM | 33470072 | 28973398 | Transcription factor A, mitochondrial |
| Q9BU76 | MMTAG2 | 16357877 | 14160087 | Multiple myeloma tumor-associated protein 2 |
| Q12905 | ILF2 | 63390550 | 54841250 | Interleukin enhancer-binding factor 2 |
| Q9NUQ6 | SPATS2L | 11969048 | 10347553 | SPATS2-like protein |
| Q9BRJ6 | C7orf50 | 5143580 | 4445504 | Uncharacterized protein C7orf50 |
| P62805 | H4-16 | 238630350 | 205924960 | Histone H4 |
| P15927 | RPA2 | 73490000 | 63198310 | Replication protein A 32 kDa subunit |
| Q5JSZ5 | PRRC2B | 17070996 | 14677961 | Protein PRRC2B |
| P05386 | RPLP1 | 32024922 | 27489890 | 60S acidic ribosomal protein P1 |
| A8MWD9 | SNRPGP15; SNRPG | 20372760 | 17487402 | Putative small nuclear ribonucleoprotein G-like protein 15;Small nuclear ribonucleoprotein G |
| Q14258 | TRIM25 | 4560078 | 3909370 | E3 ubiquitin/ISG15 ligase TRIM25 |
| Q9UMS4 | PRPF19 | 20916532 | 17905592 | Pre-mRNA-processing factor 19 |
| Q15365 | PCBP1 | 40339452 | 34498636 | Poly(rC)-binding protein 1 |
| Q7L0Y3 | TRMT10C | 6103067 | 5211182 | tRNA methyltransferase 10 homolog C |
| Q9H4L4 | SENP3 | 11042195 | 9398468 | Sentrin-specific protease 3 |
| O60684 | KPNA6 | 11590226 | 9860692 | Importin subunit alpha-7 |
| Q9NZ01 | TECR | 9391385 | 7981925 | Very-long-chain enoyl-CoA reductase |
| O94906 | PRPF6 | 7212617 | 6113574 | Pre-mRNA-processing factor 6 |
| P15880 | RPS2 | 349192260 | 295802800 | 40S ribosomal protein S2 |
| P17844 | DDX5 | 81215900 | 68778840 | Probable ATP-dependent RNA helicase DDX5 |
| P18583 | SON | 7986460 | 6751730 | Protein SON |
| O95218 | ZRANB2 | 136889760 | 115623150 | Zinc finger Ran-binding domain-containing protein 2 |
| O75643 | SNRNP200 | 21812114 | 18414836 | U5 small nuclear ribonucleoprotein 200 kDa helicase |
| Q14152 | EIF3A | 8751556 | 7386792 | Eukaryotic translation initiation factor 3 subunit A |
| Q15366 | PCBP2 | 94658650 | 79717830 | Poly(rC)-binding protein 2 |
| Q9C0J8 | WDR33 | 10267130 | 8633673 | pre-mRNA 3' end processing protein WDR33 |
| Q8IV63 | VRK3 | 6946317 | 5836629 | Inactive serine/threonine-protein kinase VRK3 |
| P55072 | VCP | 7192016 | 6023293 | Transitional endoplasmic reticulum ATPase |
| Q15393 | SF3B3 | 31299724 | 26211030 | Splicing factor 3B subunit 3 |
| P46782 | RPS5 | 163274830 | 136663840 | 40S ribosomal protein S5 |
| P84103 | SRSF3 | 118675480 | 99309840 | Serine/arginine-rich splicing factor 3 |
| Q9BY77 | POLDIP3 | 18489864 | 15450553 | Polymerase delta-interacting protein 3 |
| Q96RR1 | TWNK | 9856704 | 8228650 | Twinkle protein, mitochondrial |
| Q8TA86 | RP9 | 11247730 | 9386887 | Retinitis pigmentosa 9 protein |
| P62266 | RPS23 | 127129100 | 106085240 | 40S ribosomal protein S23 |
| O00273 | DFFA | 12442606 | 10376249 | DNA fragmentation factor subunit alpha |
| O15226 | NKRF | 18438744 | 15369616 | NF-kappa-B-repressing factor |
| O75533 | SF3B1 | 20673052 | 17226984 | Splicing factor 3B subunit 1 |
| Q68D10 | SPTY2D1 | 9182330 | 7641699 | Protein SPT2 homolog |
| P09429 | HMGB1 | 16538593 | 13754835 | High mobility group protein B1 |
| P09874 | PARP1 | 36677044 | 30470794 | Poly [ADP-ribose] polymerase 1 |
| P13639 | EEF2 | 95448640 | 79272600 | Elongation factor 2 |
| O95232 | LUC7L3 | 3959722 | 3287110 | Luc7-like protein 3 |
| Q99848 | EBNA1BP2 | 12347020 | 10231939 | Probable rRNA-processing protein EBP2 |

| | | | | |
|---|---|---|---|---|
| P0DMV8;P0DMV9 | HSPA1A;HSPA1B | 55614310 | 46058460 | Heat shock 70 kDa protein 1A;Heat shock 70 kDa protein 1B |
| Q5JVF3 | PCID2 | 30377660 | 25136246 | PCI domain-containing protein 2 |
| Q66PJ3 | ARL6IP4 | 10076192 | 8303334 | ADP-ribosylation factor-like protein 6-interacting protein 4 |
| Q07955 | SRSF1 | 78530940 | 64630720 | Serine/arginine-rich splicing factor 1 |
| P83111 | LACTB | 54429188 | 44788316 | Serine beta-lactamase-like protein LACTB, mitochondrial |
| Q8IX01 | SUGP2 | 21875196 | 17974340 | SURP and G-patch domain-containing protein 2 |
| P62829 | RPL23 | 204615310 | 167603890 | 60S ribosomal protein L23 |
| P17812 | CTPS1 | 2384824 | 1936322 | CTP synthase 1 |
| Q6UB35 | MTHFD1L | 1663823 | 1350383 | Monofunctional C1-tetrahydrofolate synthase, mitochondrial |
| Q5RKV6 | EXOSC6 | 8246019 | 6683924 | Exosome complex component MTR3 |
| Q9NYF8 | BCLAF1 | 41556400 | 33638000 | Bcl-2-associated transcription factor 1 |
| P28799 | GRN | 108766390 | 87884910 | Progranulin |
| P98175 | RBM10 | 44900390 | 36252496 | RNA-binding protein 10 |
| Q96SB4 | SRPK1 | 6418899 | 5177843 | SRSF protein kinase 1 |
| Q8TDD1 | DDX54 | 12930119 | 10424859 | ATP-dependent RNA helicase DDX54 |
| Q9BVP2 | GNL3 | 26351544 | 21239842 | Guanine nucleotide-binding protein-like 3 |
| P13010 | XRCC5 | 21918030 | 17625090 | X-ray repair cross-complementing protein 5 |
| P31689 | DNAJA1 | 8958002 | 7164119 | DnaJ homolog subfamily A member 1 |
| P42696 | RBM34 | 17866678 | 14283702 | RNA-binding protein 34 |
| P12956 | XRCC6 | 22850356 | 18221674 | X-ray repair cross-complementing protein 6 |
| P25440 | BRD2 | 31794772 | 25354260 | Bromodomain-containing protein 2 |
| Q8IY81 | FTSJ3 | 22356856 | 17805878 | pre-rRNA 2'-O-ribose RNA methyltransferase FTSJ3 |
| Q9NVI7 | ATAD3A | 14522807 | 11554753 | ATPase family AAA domain-containing protein 3A |
| P14678;P63162 | SNRPB;SNRPN | 18845402 | 14939403 | Small nuclear ribonucleoprotein-associated proteins B and B';Small nuclear ribonucleoprotein-associated protein N |
| Q13310 | PABPC4 | 163394110 | 129517470 | Polyadenylate-binding protein 4 |
| Q13523 | PRPF4B | 11472060 | 9087577 | Serine/threonine-protein kinase PRP4 homolog |
| P10809 | HSPD1 | 7727907 | 6118916 | 60 kDa heat shock protein, mitochondrial |
| Q7Z6E9 | RBBP6 | 23879992 | 18856108 | E3 ubiquitin-protein ligase RBBP6 |
| Q8WVV9 | HNRNPLL | 7244696 | 5708478 | Heterogeneous nuclear ribonucleoprotein L-like |
| Q13601 | KRR1 | 10202703 | 8037041 | KRR1 small subunit processome component homolog |
| Q9Y520 | PRRC2C | 20803596 | 16384930 | Protein PRRC2C |
| P23396 | RPS3 | 695706560 | 547667200 | 40S ribosomal protein S3 |
| P02768 | ALB | 414355300 | 325134300 | Albumin |
| Q9Y224 | RTRAF | 27608564 | 21646920 | RNA transcription, translation and transport factor protein |
| P36873 | PPP1CC | 65304108 | 51138464 | Serine/threonine-protein phosphatase PP1-gamma catalytic subunit |
| Q9Y4W2 | LAS1L | 17052842 | 13352660 | Ribosomal biogenesis protein LAS1L |
| P52701 | MSH6 | 7622915 | 5958425 | DNA mismatch repair protein Msh6 |
| Q9H7B2 | RPF2 | 10140718 | 7905324 | Ribosome production factor 2 homolog |

| | | | | |
|---|---|---|---|---|
| P38159 | RBMX | 55331532 | 43008068 | RNA-binding motif protein, X chromosome |
| P27694 | RPA1 | 111586200 | 86672820 | Replication protein A 70 kDa DNA-binding subunit |
| Q6Y7W6 | GIGYF2 | 11518665 | 8929730 | GRB10-interacting GYF protein 2 |
| Q9Y3B4 | SF3B6 | 9704915 | 7511978 | Splicing factor 3B subunit 6 |
| Q00839 | HNRNPU | 30345448 | 23482872 | Heterogeneous nuclear ribonucleoprotein U |
| P60228 | EIF3E | 14066919 | 10877672 | Eukaryotic translation initiation factor 3 subunit E |
| Q5SY16 | NOL9 | 6554294 | 5067655 | Polynucleotide 5'-hydroxyl-kinase NOL9 |
| O14802 | POLR3A | 39406150 | 30390444 | DNA-directed RNA polymerase III subunit RPC1 |
| P0DN76;Q01081 | U2AF1L5;U2AF1 | 29393436 | 22653554 | Splicing factor U2AF 35 kDa subunit-like protein;Splicing factor U2AF 35 kDa subunit |
| Q86WP2 | GPBP1 | 238749500 | 183893710 | Vasculin |
| P62244 | RPS15A | 214886030 | 165357360 | 40S ribosomal protein S15a |
| P04637 | TP53 | 38688544 | 29751298 | Cellular tumor antigen p53 |
| Q9H0D6 | XRN2 | 17415296 | 13384459 | 5'-3' exoribonuclease 2 |
| O15042 | U2SURP | 19874806 | 15262494 | U2 snRNP-associated SURP motif-containing protein |
| P52597 | HNRNPF | 128374824 | 98575530 | Heterogeneous nuclear ribonucleoprotein F |
| Q12789 | GTF3C1 | 9322425 | 7156455 | General transcription factor 3C polypeptide 1 |
| P57678 | GEMIN4 | 7974414 | 6116462 | Gem-associated protein 4 |
| Q07864 | POLE | 3405567 | 2610141 | DNA polymerase epsilon catalytic subunit A |
| Q8IZL8 | PELP1 | 36080820 | 27594856 | Proline-, glutamic acid- and leucine-rich protein 1 |
| P61978 | HNRNPK | 412620200 | 314651940 | Heterogeneous nuclear ribonucleoprotein K |
| P35908 | KRT2 | 96602880 | 73590460 | Keratin, type II cytoskeletal 2 epidermal |
| O15371 | EIF3D | 16695932 | 12695713 | Eukaryotic translation initiation factor 3 subunit D |
| P08621 | SNRNP70 | 18404946 | 13975100 | U1 small nuclear ribonucleoprotein 70 kDa |
| Q9BQG0 | MYBBP1A | 34470052 | 26112486 | Myb-binding protein 1A |
| P09651 | HNRNPA1 | 9597381 | 7264238 | Heterogeneous nuclear ribonucleoprotein A1 |
| Q08211 | DHX9 | 51302390 | 38719692 | ATP-dependent RNA helicase A |
| Q13347 | EIF3I | 8061730 | 6078889 | Eukaryotic translation initiation factor 3 subunit I |
| Q9Y3C1 | NOP16 | 2244466 | 1691895 | Nucleolar protein 16 |
| Q6PCB5 | RSBN1L | 4561114 | 3434163 | Lysine-specific demethylase RSBN1L |
| P61927 | RPL37 | 19222552 | 14468299 | 60S ribosomal protein L37 |
| P62841 | RPS15 | 202021870 | 151650300 | 40S ribosomal protein S15 |
| Q3KQU3 | MAP7D1 | 11853749 | 8878131 | MAP7 domain-containing protein 1 |
| Q9BTT0 | ANP32E | 9944620 | 7440549 | Acidic leucine-rich nuclear phosphoprotein 32 family member E |
| Q9UQ80 | PA2G4 | 6323653 | 4704934 | Proliferation-associated protein 2G4 |
| P52292 | KPNA2 | 4928604 | 3664865 | Importin subunit alpha-1 |
| Q5BKY9 | FAM133B | 15333084 | 11335007 | Protein FAM133B |
| P82930 | MRPS34 | 7348594 | 5430235 | 28S ribosomal protein S34, mitochondrial |

| | | | | |
|---|---|---|---|---|
| Q99496 | RNF2 | 10564094 | 7772404 | E3 ubiquitin-protein ligase RING2 |
| O75152 | ZC3H11A | 19762288 | 14538295 | Zinc finger CCCH domain-containing protein 11A |
| Q14684 | RRP1B | 22102856 | 16215120 | Ribosomal RNA processing protein 1 homolog B |
| P11388 | TOP2A | 384600000 | 281994980 | DNA topoisomerase 2-alpha |
| O60506 | SYNCRIP | 19987526 | 14650916 | Heterogeneous nuclear ribonucleoprotein Q |
| O43823 | AKAP8 | 37486996 | 27376532 | A-kinase anchor protein 8 |
| P13489 | RNH1 | 21625406 | 15792311 | Ribonuclease inhibitor |
| Q96MU7 | YTHDC1 | 18992768 | 13868964 | YTH domain-containing protein 1 |
| P82650 | MRPS22 | 18618858 | 13594200 | 28S ribosomal protein S22, mitochondrial |
| O75525 | KHDRBS3 | 174866880 | 127571660 | KH domain-containing, RNA-binding, signal transduction-associated protein 3 |
| O00139 | KIF2A | 8578536 | 6254691 | Kinesin-like protein KIF2A |
| Q96J01 | THOC3 | 14158538 | 10318083 | THO complex subunit 3 |
| Q15020 | SART3 | 21226606 | 15457686 | Squamous cell carcinoma antigen recognized by T-cells 3 |
| Q9Y5Q8 | GTF3C5 | 14993856 | 10915882 | General transcription factor 3C polypeptide 5 |
| P11142 | HSPA8 | 70982030 | 51643052 | Heat shock cognate 71 kDa protein |
| Q8IXT5 | RBM12B | 9833128 | 7144239 | RNA-binding protein 12B |
| P49750 | YLPM1 | 162932100 | 118141520 | YLP motif-containing protein 1 |
| Q02241 | KIF23 | 5961013 | 4300993 | Kinesin-like protein KIF23 |
| Q96I25 | RBM17 | 12741240 | 9161527 | Splicing factor 45 |
| P22626 | HNRNPA2B1 | 15012814 | 10781079 | Heterogeneous nuclear ribonucleoproteins A2/B1 |
| P60981 | DSTN | 41334736 | 29663068 | Destrin |
| Q8WUA4 | GTF3C2 | 15782029 | 11318717 | General transcription factor 3C polypeptide 2 |
| Q9BRX2 | PELO | 15874722 | 11383946 | Protein pelota homolog |
| Q12899 | TRIM26 | 2970710 | 2117622 | Tripartite motif-containing protein 26 |
| P06899;P23527;P33778;Q16778 | H2BC11;H2BC17;H2BC3;H2BC21 | 143292900 | 102072990 | Histone H2B type 1-J;Histone H2B type 1-O;Histone H2B type 1-B;Histone H2B type 2-E |
| Q14694 | USP10 | 4630086 | 3297743 | Ubiquitin carboxyl-terminal hydrolase 10 |
| Q96KR1 | ZFR | 12070245 | 8594972 | Zinc finger RNA-binding protein |
| P04264 | KRT1 | 119442024 | 85013416 | Keratin, type II cytoskeletal 1 |
| Q7KZ85 | SUPT6H | 8890645 | 6307455 | Transcription elongation factor SPT6 |
| P26373 | RPL13 | 452192500 | 320013980 | 60S ribosomal protein L13 |
| P23528 | CFL1 | 27569866 | 19365378 | Cofilin-1 |
| Q15181 | PPA1 | 4660793 | 3264760 | Inorganic pyrophosphatase |
| P35527 | KRT9 | 85759450 | 59939090 | Keratin, type I cytoskeletal 9 |
| P56537 | EIF6 | 11746568 | 8209205 | Eukaryotic translation initiation factor 6 |
| P35659 | DEK | 7874522 | 5465546 | Protein DEK |
| Q08J23 | NSUN2 | 19027590 | 13185442 | RNA cytosine C(5)-methyltransferase NSUN2 |
| P62995 | TRA2B | 48457816 | 33537026 | Transformer-2 protein homolog beta |
| O00411 | POLRMT | 12406451 | 8530812 | DNA-directed RNA polymerase, mitochondrial |
| Q9NXF1 | TEX10 | 34019740 | 23386242 | Testis-expressed protein 10 |
| Q96B26 | EXOSC8 | 11003639 | 7539103 | Exosome complex component RRP43 |
| Q9HCS7 | XAB2 | 15404188 | 10517564 | Pre-mRNA-splicing factor SYF1 |
| Q9H0A0 | NAT10 | 32114084 | 21903662 | RNA cytidine acetyltransferase |
| P02533 | KRT14 | 107181950 | 72939380 | Keratin, type I cytoskeletal 14 |

| | | | | |
|---|---|---|---|---|
| Q09028 | RBBP4 | 45075264 | 30629760 | Histone-binding protein RBBP4 |
| P51991 | HNRNPA3 | 34288080 | 23192730 | Heterogeneous nuclear ribonucleoprotein A3 |
| P26599 | PTBP1 | 33661816 | 22733336 | Polypyrimidine tract-binding protein 1 |
| Q7L2H7 | EIF3M | 5568896 | 3735355 | Eukaryotic translation initiation factor 3 subunit M |
| P63208 | SKP1 | 8370328 | 5522935 | S-phase kinase-associated protein 1 |
| Q5BKZ1 | ZNF326 | 50828020 | 33512858 | DBIRD complex subunit ZNF326 |
| O75531 | BANF1 | 94294310 | 62163210 | Barrier-to-autointegration factor |
| O75554 | WBP4 | 2427903 | 1599479 | WW domain-binding protein 4 |
| P49756 | RBM25 | 8565228 | 5642426 | RNA-binding protein 25 |
| Q9NXE8 | CWC25 | 3056709 | 2011830 | Pre-mRNA-splicing factor CWC25 homolog |
| Q92547 | TOPBP1 | 2498415 | 1644349 | DNA topoisomerase 2-binding protein 1 |
| O75494 | SRSF10 | 18957600 | 12460560 | Serine/arginine-rich splicing factor 10 |
| P63167 | DYNLL1 | 21584632 | 14165556 | Dynein light chain 1, cytoplasmic |
| O15027 | SEC16A | 30250172 | 19789726 | Protein transport protein Sec16A |
| P41223 | BUD31 | 3824572 | 2489979 | Protein BUD31 homolog |
| P07996 | THBS1 | 4578266 | 2979485 | Thrombospondin-1 |
| Q96FV9 | THOC1 | 4839652 | 3142712 | THO complex subunit 1 |
| Q9H5H4 | ZNF768 | 17790028 | 11552044 | Zinc finger protein 768 |
| P41091;Q2VIR3 | EIF2S3;EIF2S3B | 8706353 | 5653439 | Eukaryotic translation initiation factor 2 subunit 3;Eukaryotic translation initiation factor 2 subunit 3B |
| Q8TDN6 | BRIX1 | 7065389 | 4581070 | Ribosome biogenesis protein BRX1 homolog |
| Q9NYH9 | UTP6 | 4246899 | 2740546 | U3 small nucleolar RNA-associated protein 6 homolog |
| P62906 | RPL10A | 105050376 | 67752630 | 60S ribosomal protein L10a |
| Q9Y5Q9 | GTF3C3 | 10911923 | 7031570 | General transcription factor 3C polypeptide 3 |
| Q9P2D1 | CHD7 | 8573033 | 5508085 | Chromodomain-helicase-DNA-binding protein 7 |
| P05455 | SSB | 3857027 | 2477324 | Lupus La protein |
| P55884 | EIF3B | 13732301 | 8811750 | Eukaryotic translation initiation factor 3 subunit B |
| Q9Y3A4 | RRP7A | 1286620 | 824921 | Ribosomal RNA-processing protein 7 homolog A |
| Q9H1I8 | ASCC2 | 8212078 | 5248363 | Activating signal cointegrator 1 complex subunit 2 |
| O95429 | BAG4 | 10359488 | 6620037 | BAG family molecular chaperone regulator 4 |
| Q8ND24 | RNF214 | 9817666 | 6262802 | RING finger protein 214 |
| P35250 | RFC2 | 8379490 | 5342428 | Replication factor C subunit 2 |
| Q92615 | LARP4B | 15206595 | 9655002 | La-related protein 4B |
| Q5VZL5 | ZMYM4 | 9878208 | 6234005 | Zinc finger MYM-type protein 4 |
| Q13247 | SRSF6 | 126852610 | 80011750 | Serine/arginine-rich splicing factor 6 |
| Q9Y383 | LUC7L2 | 24634838 | 15530005 | Putative RNA-binding protein Luc7-like 2 |
| Q96T37 | RBM15 | 12250915 | 7721294 | RNA-binding protein 15 |
| O43143 | DHX15 | 17292636 | 10898462 | Pre-mRNA-splicing factor ATP-dependent RNA helicase DHX15 |
| P53999 | SUB1 | 53772780 | 33824610 | Activated RNA polymerase II transcriptional coactivator p15 |
| Q15717 | ELAVL1 | 35123864 | 22007190 | ELAV-like protein 1 |
| O00170 | AIP | 44749536 | 27928106 | AH receptor-interacting protein |

| | | | | |
|---|---|---|---|---|
| P49327 | FASN | 8091290 | 5045477 | Fatty acid synthase |
| Q14966 | ZNF638 | 11696957 | 7280055 | Zinc finger protein 638 |
| P62316 | SNRPD2 | 41537804 | 25813212 | Small nuclear ribonucleoprotein Sm D2 |
| Q9BTE3 | MCMBP | 1768449 | 1098913 | Mini-chromosome maintenance complex-binding protein |
| P68400 | CSNK2A1 | 25543734 | 15845822 | Casein kinase II subunit alpha |
| P49411 | TUFM | 11796423 | 7315067 | Elongation factor Tu, mitochondrial |
| P83731 | RPL24 | 159414820 | 98254890 | 60S ribosomal protein L24 |
| O60884 | DNAJA2 | 12985766 | 7988278 | DnaJ homolog subfamily A member 2 |
| P62140 | PPP1CB | 15144764 | 9303370 | Serine/threonine-protein phosphatase PP1-beta catalytic subunit |
| Q9GZT3 | SLIRP | 2406534 | 1474821 | SRA stem-loop-interacting RNA-binding protein, mitochondrial |
| Q9NY12 | GAR1 | 19840084 | 12139517 | H/ACA ribonucleoprotein complex subunit 1 |
| O60716 | CTNND1 | 12683231 | 7754465 | Catenin delta-1 |
| Q15050 | RRS1 | 7183024 | 4367501 | Ribosome biogenesis regulatory protein homolog |
| Q9NZB2 | FAM120A | 13284002 | 8070023 | Constitutive coactivator of PPAR-gamma-like protein 1 |
| Q9UN86 | G3BP2 | 23191818 | 14048893 | Ras GTPase-activating protein-binding protein 2 |
| O95816 | BAG2 | 6838149 | 4136086 | BAG family molecular chaperone regulator 2 |
| P04183 | TK1 | 1551996 | 931968 | Thymidine kinase, cytosolic |
| Q969X6 | UTP4 | 7010628 | 4190368 | U3 small nucleolar RNA-associated protein 4 homolog |
| Q14974 | KPNB1 | 9700324 | 5756838 | Importin subunit beta-1 |
| Q8NC56 | LEMD2 | 9771183 | 5788698 | LEM domain-containing protein 2 |
| P49207 | RPL34 | 228702370 | 135158320 | 60S ribosomal protein L34 |
| O00483 | NDUFA4 | 6857376 | 4047899 | Cytochrome c oxidase subunit NDUFA4 |
| Q96PV6 | LENG8 | 430678600 | 253648530 | Leukocyte receptor cluster member 8 |
| Q96GQ7 | DDX27 | 3961301 | 2332176 | Probable ATP-dependent RNA helicase DDX27 |
| Q9H0C8 | ILKAP | 3900556 | 2280403 | Integrin-linked kinase-associated serine/threonine phosphatase 2C |
| Q96D46 | NMD3 | 5177033 | 3025519 | 60S ribosomal export protein NMD3 |
| Q9Y265 | RUVBL1 | 3377186 | 1962388 | RuvB-like 1 |
| P19338 | NCL | 14259180 | 8243695 | Nucleolin |
| O43251;Q9NWB1 | RBFOX2;RBFOX1 | 7214619 | 4170104 | RNA binding protein fox-1 homolog 2;RNA binding protein fox-1 homolog 1 |
| Q12926 | ELAVL2 | 2452723 | 1410349 | ELAV-like protein 2 |
| Q9Y3Y2 | CHTOP | 13877054 | 7972591 | Chromatin target of PRMT1 protein |
| Q13243 | SRSF5 | 22918184 | 13076437 | Serine/arginine-rich splicing factor 5 |
| Q9H2P0 | ADNP | 6948162 | 3961647 | Activity-dependent neuroprotector homeobox protein |
| Q13642 | FHL1 | 14907378 | 8469029 | Four and a half LIM domains protein 1 |
| P41252 | IARS1 | 7071147 | 3989464 | Isoleucine--tRNA ligase, cytoplasmic |
| P35637 | FUS | 2201548 | 1241942 | RNA-binding protein FUS |
| Q14166 | TTLL12 | 24891880 | 14030859 | Tubulin--tyrosine ligase-like protein 12 |
| P50914 | RPL14 | 232337460 | 130610450 | 60S ribosomal protein L14 |
| O60293 | ZFC3H1 | 40277444 | 22638890 | Zinc finger C3H1 domain-containing protein |
| Q03701 | CEBPZ | 814113 | 454747 | CCAAT/enhancer-binding protein zeta |
| P11021 | HSPA5 | 11782395 | 6569875 | Endoplasmic reticulum chaperone BiP |
| P25205 | MCM3 | 18062704 | 9917306 | DNA replication licensing factor MCM3 |

| | | | | |
|---|---|---|---|---|
| B5ME19 | EIF3CL;EIF3C | 46503452 | 25461240 | Eukaryotic translation initiation factor 3 subunit C-like protein;Eukaryotic translation initiation factor 3 subunit C |
| Q15024 | EXOSC7 | 21361382 | 11692610 | Exosome complex component RRP42 |
| Q8IWS0 | PHF6 | 125276510 | 68451100 | PHD finger protein 6 |
| P12268 | IMPDH2 | 6529715 | 3567170 | Inosine-5'-monophosphate dehydrogenase 2 |
| P62847 | RPS24 | 53091170 | 28957068 | 40S ribosomal protein S24 |
| P24666 | ACP1 | 53104388 | 28960314 | Low molecular weight phosphotyrosine protein phosphatase |
| P30050 | RPL12 | 141872640 | 77324970 | 60S ribosomal protein L12 |
| P62873;P62879;Q9HAV0 | GNB1;GNB2;GNB4 | 4800507 | 2601603 | Guanine nucleotide-binding protein G(I)/G(S)/G(T) subunit beta-1;Guanine nucleotide-binding protein G(I)/G(S)/G(T) subunit beta-2;Guanine nucleotide-binding protein subunit beta-4 |
| Q6PK04 | CCDC137 | 7156623 | 3876111 | Coiled-coil domain-containing protein 137 |
| Q16576 | RBBP7 | 8543694 | 4582315 | Histone-binding protein RBBP7 |
| P60866 | RPS20 | 221806370 | 118703080 | 40S ribosomal protein S20 |
| Q9BZE4 | GTPBP4 | 17236242 | 9193605 | GTP-binding protein 4 |
| P11182 | DBT | 2289692 | 1220475 | Lipoamide acyltransferase component of branched-chain alpha-keto acid dehydrogenase complex, mitochondrial |
| P30153 | PPP2R1A | 5811729 | 3094406 | Serine/threonine-protein phosphatase 2A 65 kDa regulatory subunit A alpha isoform |
| P35244 | RPA3 | 31202402 | 16582203 | Replication protein A 14 kDa subunit |
| O15541 | RNF113A | 8992617 | 4778357 | E3 ubiquitin-protein ligase RNF113A |
| Q01130 | SRSF2 | 43581224 | 23078846 | Serine/arginine-rich splicing factor 2 |
| Q96JN8 | NEURL4 | 22400854 | 11761034 | Neuralized-like protein 4 |
| P49368 | CCT3 | 7431988 | 3900510 | T-complex protein 1 subunit gamma |
| Q9Y230 | RUVBL2 | 8622639 | 4523196 | RuvB-like 2 |
| Q00325 | SLC25A3 | 15419188 | 8032995 | Phosphate carrier protein, mitochondrial |
| P39687 | ANP32A | 14239340 | 7415298 | Acidic leucine-rich nuclear phosphoprotein 32 family member A |
| Q9UKM9 | RALY | 76107510 | 39244456 | RNA-binding protein Raly |
| O75569 | PRKRA | 4016528 | 2067525 | Interferon-inducible double-stranded RNA-dependent protein kinase activator A |
| Q13330 | MTA1 | 7980881 | 4067422 | Metastasis-associated protein MTA1 |
| Q9UKN8 | GTF3C4 | 14311753 | 7165303 | General transcription factor 3C polypeptide 4 |
| Q8WWK9 | CKAP2 | 14053146 | 7032275 | Cytoskeleton-associated protein 2 |
| Q99459 | CDC5L | 5997230 | 2996505 | Cell division cycle 5-like protein |
| Q9H2U1 | DHX36 | 3025901 | 1500429 | ATP-dependent DNA/RNA helicase DHX36 |
| Q7Z2T5 | TRMT1L | 9524330 | 4697765 | TRMT1-like protein |
| P61254 | RPL26 | 24099904 | 11879974 | 60S ribosomal protein L26 |
| P15170 | GSPT1 | 5703519 | 2804137 | Eukaryotic peptide chain release factor GTP-binding subunit ERF3A |
| Q9GZL7 | WDR12 | 5884766 | 2878715 | Ribosome biogenesis protein WDR12 |
| P35249 | RFC4 | 15370612 | 7510449 | Replication factor C subunit 4 |
| Q2TAY7 | SMU1 | 7036314 | 3416221 | WD40 repeat-containing protein SMU1 |
| Q92804 | TAF15 | 6175351 | 2987109 | TATA-binding protein-associated factor 2N |
| Q9BVA1 | TUBB2B | 67336440 | 32383518 | Tubulin beta-2B chain |

| | | | | |
|---|---|---|---|---|
| Q9NW08 | POLR3B | 8787440 | 4206934 | DNA-directed RNA polymerase III subunit RPC2 |
| P13647 | KRT5 | 21307914 | 10199529 | Keratin, type II cytoskeletal 5 |
| Q96RP9 | GFM1 | 2721015 | 1294622 | Elongation factor G, mitochondrial |
| Q9BUQ8 | DDX23 | 3106627 | 1477096 | Probable ATP-dependent RNA helicase DDX23 |
| Q8IXK0 | PHC2 | 3560774 | 1685479 | Polyhomeotic-like protein 2 |
| O75717 | WDHD1 | 10728235 | 5040459 | WD repeat and HMG-box DNA-binding protein 1 |
| Q06265 | EXOSC9 | 9730700 | 4549693 | Exosome complex component RRP45 |
| Q9H2H8 | PPIL3 | 6554916 | 3060005 | Peptidyl-prolyl cis-trans isomerase-like 3 |
| Q9Y2P8 | RCL1 | 3949696 | 1823150 | RNA 3'-terminal phosphate cyclase-like protein |
| Q13242 | SRSF9 | 38731640 | 17851712 | Serine/arginine-rich splicing factor 9 |
| Q01831 | XPC | 5474794 | 2523085 | DNA repair protein complementing XP-C cells |
| O15198 | SMAD9;SMAD3;SMAD2 | 14045899 | 6379745 | Mothers against decapentaplegic homolog 9;Mothers against decapentaplegic homolog 3;Mothers against decapentaplegic homolog 2 |
| Q96G61 | NUDT11 | 3967872 | 1800594 | Diphosphoinositol polyphosphate phosphohydrolase 3-beta |
| Q2TBE0 | CWF19L2 | 4786296 | 2146829 | CWF19-like protein 2 |
| Q53H12 | AGK | 4906810 | 2199905 | Acylglycerol kinase, mitochondrial |
| O14617 | AP3D1 | 4644552 | 2079414 | AP-3 complex subunit delta-1 |
| Q9UIG0 | BAZ1B | 7755260 | 3435934 | Tyrosine-protein kinase BAZ1B |
| Q86U42 | PABPN1 | 12143504 | 5325587 | Polyadenylate-binding protein 2 |
| O43432 | EIF4G3 | 1217214 | 531668 | Eukaryotic translation initiation factor 4 gamma 3 |
| P62937 | PPIA | 12369472 | 5383294 | Peptidyl-prolyl cis-trans isomerase A |
| Q9Y314 | NOSIP | 6701777 | 2910408 | Nitric oxide synthase-interacting protein |
| Q9Y262 | EIF3L | 8247548 | 3556336 | Eukaryotic translation initiation factor 3 subunit L |
| Q14151 | SAFB2 | 14551713 | 6260269 | Scaffold attachment factor B2 |
| Q8N9N2 | ASCC1 | 7847042 | 3330334 | Activating signal cointegrator 1 complex subunit 1 |
| O14979 | HNRNPDL | 9543999 | 4046240 | Heterogeneous nuclear ribonucleoprotein D-like |
| P62304 | SNRPE | 5242496 | 2212627 | Small nuclear ribonucleoprotein E |
| Q13151 | HNRNPA0 | 42382188 | 17704796 | Heterogeneous nuclear ribonucleoprotein A0 |
| Q13573 | SNW1 | 14885426 | 6193918 | SNW domain-containing protein 1 |
| Q9UHI6 | DDX20 | 7905575 | 3232527 | Probable ATP-dependent RNA helicase DDX20 |
| O95373 | IPO7 | 12121931 | 4902394 | Importin-7 |
| Q9UN81 | L1RE1 | 12625614 | 5084838 | LINE-1 retrotransposable element ORF1 protein |
| P18887 | XRCC1 | 3779807 | 1513048 | DNA repair protein XRCC1 |
| Q7Z478 | DHX29 | 3124136 | 1222778 | ATP-dependent RNA helicase DHX29 |
| Q9BSH4 | TACO1 | 11178554 | 4358767 | Translational activator of cytochrome c oxidase 1 |
| O43592 | XPOT | 2755442 | 1066668 | Exportin-T |
| Q9NQT5 | EXOSC3 | 11003068 | 4246090 | Exosome complex component RRP40 |
| Q969S3 | ZNF622 | 5786757 | 2212675 | Zinc finger protein 622 |
| P19388 | POLR2E | 11082043 | 4217671 | DNA-directed RNA polymerases I, II, and III subunit RPABC1 |

| | | | | |
|---|---|---|---|---|
| P40937 | RFC5 | 6629584 | 2514871 | Replication factor C subunit 5 |
| Q9P258 | RCC2 | 5984233 | 2266792 | Protein RCC2 |
| Q86W42 | THOC6 | 6470559 | 2445161 | THO complex subunit 6 homolog |
| P21964 | COMT | 4008978 | 1503758 | Catechol O-methyltransferase |
| P49458 | SRP9 | 6589991 | 2457286 | Signal recognition particle 9 kDa protein |
| P62633 | CNBP | 18885626 | 6870225 | Cellular nucleic acid-binding protein |
| Q9Y3B9 | RRP15 | 11405880 | 4071314 | RRP15-like protein |
| O43290 | SART1 | 6181903 | 2177178 | U4/U6.U5 tri-snRNP-associated protein 1 |
| Q96DT7 | ZBTB10 | 18011288 | 6308495 | Zinc finger and BTB domain-containing protein 10 |
| Q53F19 | NCBP3 | 5486064 | 1913838 | Nuclear cap-binding protein subunit 3 |
| Q9UGI8 | TES | 15008339 | 5220099 | Testin |
| P61962 | DCAF7 | 6464354 | 2242500 | DDB1- and CUL4-associated factor 7 |
| Q9H000 | MKRN2 | 16714300 | 5747747 | Probable E3 ubiquitin-protein ligase makorin-2 |
| Q16630 | CPSF6 | 12407234 | 4256746 | Cleavage and polyadenylation specificity factor subunit 6 |
| P26583 | HMGB2 | 4741671 | 1602868 | High mobility group protein B2 |
| O60306 | AQR | 5342518 | 1791320 | RNA helicase aquarius |
| Q9UQR0 | SCML2 | 6573710 | 2200145 | Sex comb on midleg-like protein 2 |
| P63173 | RPL38 | 57653784 | 19295236 | 60S ribosomal protein L38 |
| Q02218 | OGDH | 3600557 | 1203779 | 2-oxoglutarate dehydrogenase, mitochondrial |
| Q9UII4 | HERC5 | 806597 | 264590 | E3 ISG15--protein ligase HERC5 |
| P26641 | EEF1G | 6688076 | 2164607 | Elongation factor 1-gamma |
| Q9HAV4 | XPO5 | 12329371 | 3824245 | Exportin-5 |
| Q96T88 | UHRF1 | 22902704 | 7092278 | E3 ubiquitin-protein ligase UHRF1 |
| Q8NEY8 | PPHLN1 | 10937310 | 3375895 | Periphilin-1 |
| Q9UNX4 | WDR3 | 5116111 | 1570936 | WD repeat-containing protein 3 |
| P33992 | MCM5 | 4506238 | 1383168 | DNA replication licensing factor MCM5 |
| Q14444 | CAPRIN1 | 200541180 | 61198784 | Caprin-1 |
| Q8IY67 | RAVER1 | 5320877 | 1567053 | Ribonucleoprotein PTB-binding 1 |
| P62899 | RPL31 | 178396770 | 51720524 | 60S ribosomal protein L31 |
| Q9UPT9 | USP22 | 7795205 | 2222595 | Ubiquitin carboxyl-terminal hydrolase 22 |
| Q13823 | GNL2 | 3796954 | 1064658 | Nucleolar GTP-binding protein 2 |
| Q96ME7 | ZNF512 | 5393265 | 1490385 | Zinc finger protein 512 |
| Q9UIF9 | BAZ2A | 28057598 | 7600130 | Bromodomain adjacent to zinc finger domain protein 2A |
| P09234 | SNRPC | 4956256 | 1340692 | U1 small nuclear ribonucleoprotein C |
| Q9NTJ3 | SMC4 | 14159150 | 3783541 | Structural maintenance of chromosomes protein 4 |
| Q9ULW0 | TPX2 | 9231180 | 2443600 | Targeting protein for Xklp2 |
| Q96DI7 | SNRNP40 | 6838037 | 1807787 | U5 small nuclear ribonucleoprotein 40 kDa protein |
| Q9NVI1 | FANCI | 4857450 | 1258356 | Fanconi anemia group I protein |
| O00505 | KPNA3 | 4800361 | 1235717 | Importin subunit alpha-4 |
| Q9HCK8 | CHD8 | 19211420 | 4934577 | Chromodomain-helicase-DNA-binding protein 8 |
| Q99873 | PRMT1 | 35642196 | 9134286 | Protein arginine N-methyltransferase 1 |
| Q9NQ55 | PPAN | 10229558 | 2618900 | Suppressor of SWI4 1 homolog |
| Q9Y388 | RBMX2 | 5730248 | 1453467 | RNA-binding motif protein, X-linked 2 |
| O95391 | SLU7 | 9852895 | 2476437 | Pre-mRNA-splicing factor SLU7 |
| Q96I24 | FUBP3 | 56610576 | 13968358 | Far upstream element-binding protein 3 |
| P54920 | NAPA | 4528548 | 1099123 | Alpha-soluble NSF attachment protein |
| Q9Y2L1 | DIS3 | 35315230 | 7858201 | Exosome complex exonuclease RRP44 |
| Q13769 | THOC5 | 12677660 | 2808645 | THO complex subunit 5 homolog |

| | | | | |
|---|---|---|---|---|
| Q92945 | KHSRP | 7833930 | 1730036 | Far upstream element-binding protein 2 |
| P23258 | TUBG1 | 28591088 | 6124647 | Tubulin gamma-1 chain |
| O95625 | ZBTB11 | 6164283 | 1236019 | Zinc finger and BTB domain-containing protein 11 |
| P61964 | WDR5 | 49147692 | 9782572 | WD repeat-containing protein 5 |
| P53007 | SLC25A1 | 32954300 | 6549765 | Tricarboxylate transport protein, mitochondrial |
| O95347 | SMC2 | 13697637 | 2669609 | Structural maintenance of chromosomes protein 2 |
| Q14202 | ZMYM3 | 1422094 | 270941 | Zinc finger MYM-type protein 3 |
| P36957 | DLST | 10824903 | 2044400 | Dihydrolipoyllysine-residue succinyltransferase component of 2-oxoglutarate dehydrogenase complex, mitochondrial |
| O14744 | PRMT5 | 38683490 | 7255256 | Protein arginine N-methyltransferase 5 |
| Q7Z5K2 | WAPL | 28410830 | 5223067 | Wings apart-like protein homolog |
| Q96A35 | MRPL24 | 2529994 | 427206 | 39S ribosomal protein L24, mitochondrial |
| P24468 | NR2F2 | 8674561 | 1348992 | COUP transcription factor 2 |
| O15160 | POLR1C | 12784797 | 1961939 | DNA-directed RNA polymerases I and III subunit RPAC1 |
| Q8NI36 | WDR36 | 27042290 | 3975021 | WD repeat-containing protein 36 |
| P42704 | LRPPRC | 763994800 | 110465570 | Leucine-rich PPR motif-containing protein, mitochondrial |
| Q9NPD3 | EXOSC4 | 16752797 | 2421013 | Exosome complex component RRP41 |
| O75419 | CDC45 | 7043346 | 983164 | Cell division control protein 45 homolog |
| P56270 | MAZ | 33807936 | 4489273 | Myc-associated zinc finger protein |
| Q92769 | HDAC2 | 27053248 | 3296990 | Histone deacetylase 2 |
| Q8N5C6 | SRBD1 | 6724078 | 793126 | S1 RNA-binding domain-containing protein 1 |
| Q9NQT4 | EXOSC5 | 26469228 | 3007525 | Exosome complex component RRP46 |
| Q96N67 | DOCK7 | 8151093 | 920798 | Dedicator of cytokinesis protein 7 |
| Q14181 | POLA2 | 8748157 | 943216 | DNA polymerase alpha subunit B |
| Q13838 | DDX39B | 40696640 | 4014242 | Spliceosome RNA helicase DDX39B |
| Q99959 | PKP2 | 69548440 | 6763178 | Plakophilin-2 |
| Q96RF0 | SNX18 | 4447191 | 410362 | Sorting nexin-18 |
| Q7Z4Q2 | HEATR3 | 17023776 | 1570044 | HEAT repeat-containing protein 3 |
| P28340 | POLD1 | 13743564 | 1253421 | DNA polymerase delta catalytic subunit |
| Q8IZH2 | XRN1 | 57762012 | 4943934 | 5'-3' exoribonuclease 1 |
| P27708 | CAD | 29647520 | 2427406 | CAD protein |
| Q9P035 | HACD3 | 20913350 | 1464848 | Very-long-chain (3R)-3-hydroxyacyl-CoA dehydratase 3 |
| P28331 | NDUFS1 | 12432145 | 791284 | NADH-ubiquinone oxidoreductase 75 kDa subunit, mitochondrial |
| Q9UBL3 | ASH2L | 40636430 | 1424064 | Set1/Ash2 histone methyltransferase complex subunit ASH2 |
| P82663 | MRPS25 | 36886224 | 1189121 | 28S ribosomal protein S25, mitochondrial |
| P55735 | SEC13 | 15954081 | 504380 | Protein SEC13 homolog |
| P54577 | YARS1 | 404472700 | 10653618 | Tyrosine--tRNA ligase, cytoplasmic |
| Q99547 | MPHOSPH6 | 11005367 | 187494 | M-phase phosphoprotein 6 |
| Q96RS0 | TGS1 | 15956449 | 244683 | Trimethylguanosine synthase |
| Q8IX18 | DHX40 | 37218388 | 498938 | Probable ATP-dependent RNA helicase DHX40 |
| P49643 | PRIM2 | 86659890 | 489262 | DNA primase large subunit |

| | | | | |
|---|---|---|---|---|
| Q16531 | DDB1 | 549587600 | 2799838 | DNA damage-binding protein 1 |
