# Supplementary material for: Prion‐Like Protein LENG8‐Mediated Nucleation Drives Stress Granule Assembly
Source: Adv Sci (Weinh). 2026 Sep 8:e77622. Online ahead of print. doi: 10.1002/advs.77622 (PMC13554387; doi:10.1002/advs.77622)
Supplement: Supplementary file 2 [file ADVS-9999-e77622-s002.pdf]

Table S3. Compare the intersecting proteins in the HeLa and HEK293T cell lines under arsenite condition.

IP Intensity: Protein abundance was quantified using label-free protein intensity values obtained from MSFragger-based analysis.

Protein ID	Description	HeLa-As_LENG8 (IP Intensity)	HEK293T-As_LENG8 (IP Intensity)
Q12899	Tripartite motif-containing protein 26	95986757	233381860
Q969Q0	Ribosomal protein eL42-like	3457	46773140
Q9H582	Zinc finger protein 644	3902	7069466
Q9UPT8	Zinc finger CCCH domain-containing protein 4	4439	126344340
Q9UJX4	Anaphase-promoting complex subunit 5	6252	74580720
Q5VT25	Serine/threonine-protein kinase MRCK alpha	6895	26657162
Q9H1I8	Activating signal cointegrator 1 complex subunit 2	7903	60639500
Q99569	Plakophilin-4	8035	12231198
Q8IWR0	Zinc finger CCCH domain-containing protein 7A	8099	9981982
Q13643	Four and a half LIM domains protein 3	8201	9393447
Q5JSZ5	Protein PRRC2B	8333	14856748
O00418	Eukaryotic elongation factor 2 kinase	8672	29521488
Q15650	Activating signal cointegrator 1	9085	28522332
Q8N5C6	S1 RNA-binding domain-containing protein 1	9105	74820130
Q8N1G0	Zinc finger protein 687	9177	7430025
Q9UJX2	Cell division cycle protein 23 homolog	10335	6414331
Q8NI35	InaD-like protein	10453	17742608
Q9BRD0	BUD13 homolog	10631	14892316
P46736	Lys-63-specific deubiquitinase BRCC36	11558	2882612
Q92791	Endoplasmic reticulum protein SC65	12166	11839263
Q9P031	Thyroid transcription factor 1-associated protein 26	12279	19840948
Q9NQ29	Putative RNA-binding protein Luc7-like 1	12348	23772956
O75569	Interferon-inducible double-stranded RNA-dependent protein kinase activator A	12454	22724842
Q9UN86	Ras GTPase-activating protein-binding protein 2	12560	146534620
Q07864	DNA polymerase epsilon catalytic subunit A	12675	17326248
O14647	Chromodomain-helicase-DNA-binding protein 2	12904	7502289
O94762	ATP-dependent DNA helicase Q5	13551	7062730
Q9HCK8	Chromodomain-helicase-DNA-binding protein 8	13988	16144553
Q9H1A4	Anaphase-promoting complex subunit 1	14119	5144370
Q96QE5	Transcription elongation factor, mitochondrial	14610	3135038
Q92547	DNA topoisomerase 2-binding protein 1	15231	18816258
P36954	DNA-directed RNA polymerase II subunit RPB9	15581	24571842
Q13136	Liprin-alpha-1	15688	145687980
Q96T58	Msx2-interacting protein	15704	26342842
Q96ME7	Zinc finger protein 512	15836	56833452
P52756	RNA-binding protein 5	15840	63215644
Q9BXW9	Fanconi anemia group D2 protein	16809	36681184
Q9H9B1	Histone-lysine N-methyltransferase EHMT1	17315	37834784
O95793	Double-stranded RNA-binding protein Staufen homolog 1	17361	59062584
Q6P158	Putative ATP-dependent RNA helicase DHX57	17570	25488636

Q9NUQ6	SPATS2-like protein	17601	13137648
Q12872	Splicing factor, suppressor of white-apricot homolog	18409	13945900
Q9NVI1	Fanconi anemia group I protein	18776	48977720
Q9UGR2	Zinc finger CCCH domain-containing protein 7B	18921	295802700
Q8IW35	Centrosomal protein of 97 kDa	18968	5444450
Q15633	RISC-loading complex subunit TARBP2	20739	12881940
O14802	DNA-directed RNA polymerase III subunit RPC1	20785	40206230
Q06787	Fragile X messenger ribonucleoprotein 1	22178	69010110
Q06481	Amyloid beta precursor like protein 2	22199	22915104
Q8NEY8	Periphilin-1	22485	42757824
O95239	Chromosome-associated kinesin KIF4A	22732	23714072
O94880	PHD finger protein 14	23278	35638800
Q9H967	WD repeat-containing protein 76	23310	36280740
Q86UK7	E3 ubiquitin-protein ligase ZNF598	23776	33760424
Q7L2J0	7SK snRNA methylphosphate capping enzyme	23831	11235375
P05109	Protein S100-A8	23866	19526390
P78362	SRSF protein kinase 2	24014	23699112
Q9GZR7	ATP-dependent RNA helicase DDX24	24512	75836984
P49750	YLP motif-containing protein 1	24572	188008430
Q9H0C8	Integrin-linked kinase-associated serine/threonine phosphatase 2C	24835	32251568
Q96PV6	Leukocyte receptor cluster member 8	24947	34358572
Q9NVV4	Poly(A) RNA polymerase, mitochondrial	25374	3444936
Q8N3C0	Activating signal cointegrator 1 complex subunit 3	25463	116436170
O95251	Histone acetyltransferase KAT7	26214	25518634
Q9BRU9	rRNA-processing protein UTP23 homolog	26385	15838386
Q68D10	Protein SPT2 homolog	26885	10191710
Q9Y2X7	ARF GTPase-activating protein GIT1	26943	66172572
Q9H814	Phosphorylated adapter RNA export protein	27346	13589312
Q15648	Mediator of RNA polymerase II transcription subunit 1	28332	10678173
Q9UL40	Zinc finger protein 346	28587	4612588
P49207	Large ribosomal subunit protein eL34	29393	116091130
O60244	Mediator of RNA polymerase II transcription subunit 14	29785	26575822
Q7RTV0	PHD finger-like domain-containing protein 5A	31168	10898726
Q16637	Survival motor neuron protein	31453	11951773
P06702	Protein S100-A9	31494	14802373
Q15007	Pre-mRNA-splicing regulator WTAP	31563	8017470
Q9UBL3	Set1/Ash2 histone methyltransferase complex subunit ASH2	31670	9545756
Q9UMY1	Nucleolar protein 7	31700	33715120
Q9NQZ2	Something about silencing protein 10	31986	45480210
O60293	Zinc finger C3H1 domain-containing protein	32430	2792706
Q9ULK4	Mediator of RNA polymerase II transcription subunit 23	32485	13624116
Q9Y4C8	Probable RNA-binding protein 19	32606	20674962
O00629	Importin subunit alpha-3	32644	14908653
O00566	U3 small nucleolar ribonucleoprotein protein MPP10	32677	78004950
Q9BXS6	Nucleolar and spindle-associated protein 1	33093	3320102
O15541	E3 ubiquitin-protein ligase RNF113A	33267	21261660

Q9UDY2	Tight junction protein ZO-2	33426	47847370
Q86U86	Protein polybromo-1	33619	17260622
Q9Y232	Chromodomain Y-like protein	33810	38674988
Q9UJX5	Anaphase-promoting complex subunit 4	33827	4038307
Q96DT7	Zinc finger and BTB domain-containing protein 10	34064	48795620
Q7Z6E9	E3 ubiquitin-protein ligase RBBP6	34786	167910320
Q9NUL7	Probable ATP-dependent RNA helicase DDX28	34786	7903185
Q9Y4X5	E3 ubiquitin-protein ligase ARIH1	35487	19612976
P55081	Microfibrillar-associated protein 1	35656	136835650
Q9UK61	Protein TASOR	35994	36020972
Q969S3	Cytoplasmic 60S subunit biogenesis factor ZNF622	36422	3448713
Q9H2U1	ATP-dependent DNA/RNA helicase DHX36	36718	12346756
Q8NHQ9	ATP-dependent RNA helicase DDX55	36792	17865790
P01024	Complement C3	37664	120503110
Q7Z2T5	TRMT1-like protein	37763	32520876
Q12873	Chromodomain-helicase-DNA-binding protein 3	38438	40483230
O00257	E3 SUMO-protein ligase CBX4	38698	7568246
O60884	DnaJ homolog subfamily A member 2	39022	16211772
Q5T3I0	G patch domain-containing protein 4	39370	49018176
Q03111	Protein ENL	39438	6443988
Q00577	Transcriptional activator protein Pur-alpha	39484	11353103
Q9Y4B6	DDB1- and CUL4-associated factor 1	40156	17419362
P07199	Major centromere autoantigen B	40369	46432868
P19474	E3 ubiquitin-protein ligase TRIM21	40447	63355544
Q8WVV9	Heterogeneous nuclear ribonucleoprotein L-like	41177	46422836
P62487	DNA-directed RNA polymerase II subunit RPB7	42259	22932434
Q9BU76	Multiple myeloma tumor-associated protein 2	42738	69738460
Q9GZS1	DNA-directed RNA polymerase I subunit RPA49	43239	3931121
Q9HC36	rRNA methyltransferase 3, mitochondrial	43277	12034147
Q96N67	Dedicator of cytokinesis protein 7	44217	13872459
Q8IZH2	5'-3' exoribonuclease 1	44507	53426388
Q9Y5L0	Transportin-3	44835	20944604
Q68E01	Integrator complex subunit 3	45580	19987206
Q9Y5Q8	General transcription factor 3C polypeptide 5	45627	62730110
Q9H089	Large subunit GTPase 1 homolog	45635	15674944
P22061	Protein-L-isoaspartate(D-aspartate) O-methyltransferase	45764	24265908
Q8TD26	Chromodomain-helicase-DNA-binding protein 6	46072	8870294
P11182	Lipoamide acyltransferase component of branched-chain alpha-keto acid dehydrogenase complex, mitochondrial	46332	8557788
P42766	Large ribosomal subunit protein uL29	46467	172950180
P19387	DNA-directed RNA polymerase II subunit RPB3	46629	24193404
P78346	Ribonuclease P protein subunit p30	46801	26104330
O75616	GTPase Era, mitochondrial	46929	3735996
Q9BTE3	Mini-chromosome maintenance complex-binding protein	46934	19754608
Q9BWJ5	Splicing factor 3B subunit 5	47234	29553642
Q9Y5Q9	General transcription factor 3C polypeptide 3	47274	20656854

O75934	Pre-mRNA-splicing factor SPF27	47585	23772224
P0DPB6	DNA-directed RNA polymerases I and III subunit RPAC2	48238	31142104
Q9H4L4	Sentrin-specific protease 3	48338	38142256
Q9GZR2	RNA exonuclease 4	48558	40319510
O75165	DnaJ homolog subfamily C member 13	49393	52624812
O60287	Nucleolar pre-ribosomal-associated protein 1	49471	36827656
Q7Z5L9	Interferon regulatory factor 2-binding protein 2	49861	36957150
Q9P2I0	Cleavage and polyadenylation specificity factor subunit 2	49866	22503812
Q7KZ85	Transcription elongation factor SPT6	50493	196302080
P30260	Cell division cycle protein 27 homolog	50522	18873896
O75683	Surfeit locus protein 6	50826	87602370
P35251	Replication factor C subunit 1	51180	21616106
Q5JVF3	PCI domain-containing protein 2	51273	69848120
Q1ED39	Lysine-rich nucleolar protein 1	51650	26536460
O43823	A-kinase anchor protein 8	52089	44940010
Q5T280	Putative methyltransferase C9orf114	52540	57223940
Q96SB4	SRSF protein kinase 1	52941	20563598
Q8NAV1	Pre-mRNA-splicing factor 38A	52964	68467224
Q9H2P0	Activity-dependent neuroprotector homeobox protein	53367	25660512
P19784	Casein kinase II subunit alpha'	53816	31980514
P23258	Tubulin gamma-1 chain	54003	78927544
O95391	Pre-mRNA-splicing factor SLU7	54007	25932008
Q92620	Pre-mRNA-splicing factor ATP-dependent RNA helicase PRP16	54905	2128758
Q14181	DNA polymerase alpha subunit B	55393	6529706
Q13895	Bystin	55900	75563880
P83111	Serine beta-lactamase-like protein LACTB, mitochondrial	56229	28114826
O95218	Zinc finger Ran-binding domain-containing protein 2	56776	64811790
Q8WUA2	Peptidyl-prolyl cis-trans isomerase-like 4	56845	59364930
Q01085	Nucleolysin TIAR	57022	27526280
O15145	Actin-related protein 2/3 complex subunit 3	57059	14587662
O15514	DNA-directed RNA polymerase II subunit RPB4	57556	58023548
Q9C0J8	pre-mRNA 3' end processing protein WDR33	57962	35252132
Q9H2H8	Peptidyl-prolyl cis-trans isomerase-like 3	58713	15885864
Q7Z417	FMR1-interacting protein NUFIP2	58951	12141357
Q9NU22	Midasin	59362	38471104
O15160	DNA-directed RNA polymerases I and III subunit RPAC1	59741	6840729
Q8TDD1	ATP-dependent RNA helicase DDX54	59763	69662680
Q15050	Ribosome biogenesis regulatory protein homolog	59794	102228670
Q8IZ69	tRNA (uracil-5-)-methyltransferase homolog A	60280	14442146
Q96QC0	Serine/threonine-protein phosphatase 1 regulatory subunit 10	60907	18550430
P61964	WD repeat-containing protein 5	60940	21295426
Q9Y4W2	Ribosomal biogenesis protein LAS1L	62232	68172720
Q96D46	60S ribosomal export protein NMD3	62685	23384808
Q6PJT7	Zinc finger CCCH domain-containing protein 14	62925	17551410
Q13451	Peptidyl-prolyl cis-trans isomerase FKBP5	62941	17444492

Q14257	Reticulocalbin-2	62984	44640950
Q9NZB2	Constitutive coactivator of PPAR-gamma-like protein 1	63020	29181232
Q9BWF3	RNA-binding protein 4	63986	54133160
O43660	Pleiotropic regulator 1	64337	93444090
Q96EV2	RNA-binding protein 33	64863	72829550
P27694	Replication protein A 70 kDa DNA-binding subunit	65631	73137590
O95400	CD2 antigen cytoplasmic tail-binding protein 2	66394	6831023
P21127	Cyclin-dependent kinase 11B	66729	27245892
Q9Y2P8	RNA 3'-terminal phosphate cyclase-like protein	67000	59327220
Q9BQ39	ATP-dependent RNA helicase DDX50	68087	65574570
Q6UXN9	WD repeat-containing protein 82	68101	55580068
Q9H6F5	Coiled-coil domain-containing protein 86	68188	73498970
Q9BYN8	Small ribosomal subunit protein mS26	68303	9232295
Q9Y3B4	Splicing factor 3B subunit 6	68650	74123464
Q86YP4	Transcriptional repressor p66-alpha	69002	8697901
O43159	Ribosomal RNA-processing protein 8	69008	32890070
O43719	HIV Tat-specific factor 1	69494	78438700
Q8WUA4	General transcription factor 3C polypeptide 2	69883	32421348
Q5RKV6	Exosome complex component MTR3	70406	11434055
O00267	Transcription elongation factor SPT5	71205	48379140
Q9BVI4	Nucleolar complex protein 4 homolog	71229	58668576
Q9UGI8	Testin	71466	26880220
Q9BTT0	Acidic leucine-rich nuclear phosphoprotein 32 family member E	71944	154402900
Q92879	CUGBP Elav-like family member 1	73044	17875296
Q99959	Plakophilin-2	73310	3328535
Q9NXF1	Testis-expressed protein 10	73431	44972560
Q9GZL7	Ribosome biogenesis protein WDR12	75445	118445090
O95816	BAG family molecular chaperone regulator 2	75744	22550290
Q14692	Ribosome biogenesis protein BMS1 homolog	76032	426201700
Q9Y263	Phospholipase A-2-activating protein	76278	10399988
Q9Y221	60S ribosome subunit biogenesis protein NIP7 homolog	76556	184917380
P62140	Serine/threonine-protein phosphatase PP1-beta catalytic subunit	77218	12941424
P38432	Coilin	77569	22310530
Q9Y2K7	Lysine-specific demethylase 2A	77597	42641740
O95071	E3 ubiquitin-protein ligase UBR5	77679	19271152
P08579	U2 small nuclear ribonucleoprotein B''	77759	19539868
Q9H8H0	Nucleolar protein 11	77974	88008090
Q9UNX4	WD repeat-containing protein 3	78100	119471010
O14646	Chromodomain-helicase-DNA-binding protein 1	78530	81325690
P05386	Large ribosomal subunit protein P1	78701	24853720
Q00403	Transcription initiation factor IIB	79232	15340413
Q8IY37	Probable ATP-dependent RNA helicase DHX37	79246	64537496
Q9BXP5	Serrate RNA effector molecule homolog	79519	82909070
Q13242	Serine/arginine-rich splicing factor 9	79586	247138050
Q9UKN8	General transcription factor 3C polypeptide 4	79874	29891830
Q9BV38	WD repeat-containing protein 18	81067	72308350
Q96S55	ATPase WRNIP1	81328	31872496
Q8WWM7	Ataxin-2-like protein	81348	49379744
Q9Y224	RNA transcription, translation and transport factor protein	81513	71456230

Q96KG9	N-terminal kinase-like protein	81950	69335224
P51116	RNA-binding protein FXR2	82534	53577590
O00505	Importin subunit alpha-4	82670	23923446
Q92769	Histone deacetylase 2	83420	45588516
Q9Y520	Protein PRRC2C	84613	146694430
Q9H0H5	Rac GTPase-activating protein 1	85598	20959200
O00425	Insulin-like growth factor 2 mRNA-binding protein 3	86225	67894870
Q6PKG0	La-related protein 1	86241	56892656
Q8IZL8	Proline-, glutamic acid- and leucine-rich protein 1	86447	110876100
O75691	Small subunit processome component 20 homolog	86599	78538820
Q13823	Nucleolar GTP-binding protein 2	86855	216511800
Q5SY16	Polynucleotide 5'-hydroxyl-kinase NOL9	86912	82909100
P49916	DNA ligase 3	87351	39071160
P40763	Signal transducer and activator of transcription 3	87443	3785358
Q9Y2X0	Mediator of RNA polymerase II transcription subunit 16	88129	1038483
Q9NTJ3	Structural maintenance of chromosomes protein 4	89482	18272184
Q8IWX8	Calcium homeostasis endoplasmic reticulum protein	90305	117750560
Q9UKV3	Apoptotic chromatin condensation inducer in the nucleus	91088	103622270
Q86Y56	Dynein axonemal assembly factor 5	91587	35824332
Q96T88	E3 ubiquitin-protein ligase UHRF1	92800	41240684
Q96A33	PAT complex subunit CCDC47	93307	17242708
Q9BQ67	Glutamate-rich WD repeat-containing protein 1	93489	28311812
P35249	Replication factor C subunit 4	93845	51373084
Q9Y2R9	Small ribosomal subunit protein uS7m	94189	10616778
P24928	DNA-directed RNA polymerase II subunit RPB1	95431	83086070
O60231	Pre-mRNA-splicing factor ATP-dependent RNA helicase DHX16	95886	19579844
Q9NWT1	p21-activated protein kinase-interacting protein 1	96110	59136340
Q9UBB4	Ataxin-10	96500	19155624
Q5T749	Keratinocyte proline-rich protein	97321	20315806
Q9NY93	Probable ATP-dependent RNA helicase DDX56	97869	71156170
P43246	DNA mismatch repair protein Msh2	98400	20483456
Q9ULX3	RNA-binding protein NOB1	98429	54971624
Q02878	Large ribosomal subunit protein eL6	98595	237895730
Q9H0S4	Probable ATP-dependent RNA helicase DDX47	98630	63885304
Q9H6R4	Nucleolar protein 6	98647	119089144
Q13243	Serine/arginine-rich splicing factor 5	98973	92391560
Q5BKZ1	DBIRD complex subunit ZNF326	99631	83431340
Q15061	WD repeat-containing protein 43	99713	152073570
Q9Y3C1	Nucleolar protein 16	99827	36248904
Q9NW13	RNA-binding protein 28	101211	116310776
Q9BVJ6	U3 small nucleolar RNA-associated protein 14 homolog A	102221	91516856
Q9NX58	Cell growth-regulating nucleolar protein	103549	72824100
Q8N684	Cleavage and polyadenylation specificity factor subunit 7	104485	34361050

Q10570	Cleavage and polyadenylation specificity factor subunit 1	104696	72820610
Q53GS9	Ubiquitin carboxyl-terminal hydrolase 39	104711	18118316
Q13595	Transformer-2 protein homolog alpha	105400	57819450
Q96T76	MMS19 nucleotide excision repair protein homolog	107843	38988656
Q9NW82	WD repeat-containing protein 70	108523	33464370
Q9H6R0	ATP-dependent RNA helicase DHX33	108524	20442570
P27348	14-3-3 protein theta	109066	6229650
Q14151	Scaffold attachment factor B2	109223	129682410
Q9C0C2	182 kDa tankyrase-1-binding protein	109346	15891519
Q96B26	Exosome complex component RRP43	109779	14961807
Q96CS3	FAS-associated factor 2	110127	15204428
O15042	U2 snRNP-associated SURP motif-containing protein	110430	78349590
Q15024	Exosome complex component RRP42	110787	15032475
Q14684	Ribosomal RNA processing protein 1 homolog B	110969	110560570
Q6P1J9	Parafibromin	112119	15712854
P49756	RNA-binding protein 25	113004	54791716
Q8NFW8	N-acylneuraminate cytidylyltransferase	113820	6130612
Q7L0Y3	tRNA methyltransferase 10 homolog C	114311	24617632
P40938	Replication factor C subunit 3	114527	36987710
Q96I25	Splicing factor 45	114713	40228412
P51398	Small ribosomal subunit protein mS29	114970	6232151
Q9UQR0	Sex comb on midleg-like protein 2	115087	52038816
Q9Y2L1	Exosome complex exonuclease RRP44	115253	193764420
Q96RE7	Nucleus accumbens-associated protein 1	115931	12680108
O15371	Eukaryotic translation initiation factor 3 subunit D	116375	27132322
Q12965	Unconventional myosin-Ie	116682	14574942
Q7Z4Q2	HEAT repeat-containing protein 3	116685	145534100
Q9Y4P3	Transducin beta-like protein 2	117057	245807
P67870	Casein kinase II subunit beta	117922	54473260
P61513	Large ribosomal subunit protein eL43	118625	63343612
P08779	Keratin, type I cytoskeletal 16	118730	167839310
Q9Y3A4	Ribosomal RNA-processing protein 7 homolog A	118931	54033936
Q04695	Keratin, type I cytoskeletal 17	119712	153820910
Q14192	Four and a half LIM domains protein 2	119830	10063281
Q13601	KRR1 small subunit processome component homolog	120071	121610390
Q5QJE6	Deoxynucleotidyltransferase terminal-interacting protein 2	121648	69375784
Q6UN15	Pre-mRNA 3'-end-processing factor FIP1	122087	22357518
P62917	Large ribosomal subunit protein uL2	122522	78338120
O43290	U4/U6.U5 tri-snRNP-associated protein 1	122893	36560070
O75717	WD repeat and HMG-box DNA-binding protein 1	123611	35677436
Q14146	Unhealthy ribosome biogenesis protein 2 homolog	125262	189415700
Q71RC2	La-related protein 4	125285	11937827
P17480	Nucleolar transcription factor 1	127282	49995836
Q9Y2W1	Thyroid hormone receptor-associated protein 3	127788	159287380
O15381	Nuclear valosin-containing protein-like	129150	81366930
Q8WXI9	Transcriptional repressor p66-beta	129260	6195799

Q29RF7	Sister chromatid cohesion protein PDS5 homolog A	129674	53585680
P42677	Small ribosomal subunit protein eS27	130729	29269946
Q15654	Thyroid receptor-interacting protein 6	131568	48515596
A5YKK6	CCR4-NOT transcription complex subunit 1	132187	33695630
P06493	Cyclin-dependent kinase 1	132279	38358676
Q14974	Importin subunit beta-1	132808	85729380
Q15785	Mitochondrial import receptor subunit TOM34	132962	29567408
P55735	Protein SEC13 homolog	133892	35492104
Q8TDN6	Ribosome biogenesis protein BRX1 homolog	134624	59481050
Q9BYG3	MKI67 FHA domain-interacting nucleolar phosphoprotein	135532	81297600
O95478	Ribosome biogenesis protein NSA2 homolog	136757	88421020
P68366	Tubulin alpha-4A chain	137685	64710520
Q96GM8	Target of EGR1 protein 1	138852	7793865
Q9UJV9	Probable ATP-dependent RNA helicase DDX41	140416	76071940
Q9BY44	Eukaryotic translation initiation factor 2A	142146	9596823
O60832	H/ACA ribonucleoprotein complex subunit DKC1	142321	101016070
Q14247	Src substrate cortactin	142451	65748424
Q96DI7	U5 small nuclear ribonucleoprotein 40 kDa protein	142568	77016400
Q8N163	Cell cycle and apoptosis regulator protein 2	143273	57651624
Q92917	G-patch domain and KOW motifs-containing protein	143619	13253295
O15027	Protein transport protein Sec16A	146619	46255404
Q93009	Ubiquitin carboxyl-terminal hydrolase 7	146719	16756686
Q8IY81	pre-rRNA 2'-O-ribose RNA methyltransferase FTSJ3	147917	157526140
Q9BQG0	Myb-binding protein 1A	149678	166160750
Q9NY12	H/ACA ribonucleoprotein complex subunit 1	149903	203260000
P61247	Small ribosomal subunit protein eS1	150796	217375980
P53621	Coatomer subunit alpha	151259	25681056
O60264	SWI/SNF-related matrix-associated actin-dependent regulator of chromatin subfamily A member 5	152138	107245570
P53618	Coatomer subunit beta	153042	4649000
P51532	Transcription activator BRG1	153705	20514788
Q7L2H7	Eukaryotic translation initiation factor 3 subunit M	154931	29016084
Q9Y2Z0	Protein SGT1 homolog	155638	15990583
P49959	Double-strand break repair protein MRE11	158517	6151952
P52292	Importin subunit alpha-1	159017	104885230
Q14966	Zinc finger protein 638	159437	26649568
P62280	Small ribosomal subunit protein uS17	159961	171851060
Q5JTH9	RRP12-like protein	163051	107862620
Q9NX24	H/ACA ribonucleoprotein complex subunit 2	163242	83764580
Q2NL82	Pre-rRNA-processing protein TSR1 homolog	164004	42492224
P42285	Exosome RNA helicase MTR4	164818	35970050
Q92522	Histone H1.10	166182	159913200
Q01780	Exosome complex component 10	166263	31472512
O95639	Cleavage and polyadenylation specificity factor subunit 4	166364	24791644
P62136	Serine/threonine-protein phosphatase PP1-alpha catalytic subunit	166792	38943580
Q9HCE1	Helicase MOV-10	167671	92790370

O75131	Copine-3	168743	103111690
Q15717	ELAV-like protein 1	169582	95487290
P54136	Arginine--tRNA ligase, cytoplasmic	170505	2768358
Q14690	Protein RRP5 homolog	171516	165330800
Q14683	Structural maintenance of chromosomes protein 1A	172740	76851550
Q14166	Tubulin--tyrosine ligase-like protein 12	172949	27752646
Q15459	Splicing factor 3A subunit 1	173379	43441120
P39023	Large ribosomal subunit protein uL3	173920	434513860
O75534	Cold shock domain-containing protein E1	176528	66740436
P09012	U1 small nuclear ribonucleoprotein A	176685	52040390
P46013	Proliferation marker protein Ki-67	177495	113029870
Q9Y5J1	U3 small nucleolar RNA-associated protein 18 homolog	177589	99952690
P31689	DnaJ homolog subfamily A member 1	177917	26225962
Q9NVP1	ATP-dependent RNA helicase DDX18	178308	189043950
Q9NV31	U3 small nucleolar ribonucleoprotein protein IMP3	178620	86835560
P54577	Tyrosine--tRNA ligase, cytoplasmic	179150	40223584
Q9Y383	Putative RNA-binding protein Luc7-like 2	179587	115978210
Q12788	Transducin beta-like protein 3	180469	98517820
Q9Y3F4	Serine-threonine kinase receptor-associated protein	180650	75706620
O60841	Eukaryotic translation initiation factor 5B	180794	5026467
Q09161	Nuclear cap-binding protein subunit 1	181020	31308668
Q5SSJ5	Heterochromatin protein 1-binding protein 3	181182	70709150
Q14C86	GTPase-activating protein and VPS9 domain-containing protein 1	181867	21840088
O94906	Pre-mRNA-processing factor 6	183277	68629656
Q9BY77	Polymerase delta-interacting protein 3	185386	28987330
P18077	Large ribosomal subunit protein eL33	186350	121712130
Q16531	DNA damage-binding protein 1	187778	66154990
Q969X6	U3 small nucleolar RNA-associated protein 4 homolog	189515	218919620
Q15427	Splicing factor 3B subunit 4	191777	16394093
Q9UMS4	Pre-mRNA-processing factor 19	192551	166510480
P18031	Tyrosine-protein phosphatase non-receptor type 1	196236	10224103
O95373	Importin-7	197060	37898336
P50402	Emerin	197161	94106824
Q13501	Sequestosome-1	197485	8372816
O15226	NF-kappa-B-repressing factor	199092	45182456
Q13185	Chromobox protein homolog 3	201098	89623656
Q96GQ7	Probable ATP-dependent RNA helicase DDX27	201677	113734536
Q9Y230	RuvB-like 2	202406	175710030
Q9UHB6	LIM domain and actin-binding protein 1	203468	6808216
Q14258	E3 ubiquitin/ISG15 ligase TRIM25	203862	101827460
P14923	Junction plakoglobin	204320	17986846
Q9NQT5	Exosome complex component RRP40	205947	24222330
Q15020	Squamous cell carcinoma antigen recognized by T-cells 3	209317	34761084
P62314	Small nuclear ribonucleoprotein Sm D1	211181	39962704
Q02880	DNA topoisomerase 2-beta	215460	246205700
Q07666	KH domain-containing, RNA-binding, signal transduction-associated protein 1	215869	934764700
Q2TAY7	WD40 repeat-containing protein SMU1	216497	116797580

P62266	Small ribosomal subunit protein uS12	216716	127512150
Q8N7H5	RNA polymerase II-associated factor 1 homolog	218514	3854852
Q13435	Splicing factor 3B subunit 2	218737	123315816
Q7Z2W4	Zinc finger CCCH-type antiviral protein 1	219137	31852212
P10599	Thioredoxin	220472	25635232
P36873	Serine/threonine-protein phosphatase PP1-gamma catalytic subunit	221801	154047790
P26368	Splicing factor U2AF 65 kDa subunit	222077	216455310
Q9UBQ5	Eukaryotic translation initiation factor 3 subunit K	224229	20113832
Q00341	Vigilin	226920	7608846
P07814	Bifunctional glutamate/proline--tRNA ligase	230246	9508109
Q7L2E3	ATP-dependent RNA helicase DHX30	230539	582197800
P52948	Nuclear pore complex protein Nup98-Nup96	231290	80959470
P11388	DNA topoisomerase 2-alpha	231694	199159710
P55795	Heterogeneous nuclear ribonucleoprotein H2	232238	43858990
O75694	Nuclear pore complex protein Nup155	233641	60555690
P02533	Keratin, type I cytoskeletal 14	233837	323284800
Q9Y5S9	RNA-binding protein 8A	235826	86695304
O75822	Eukaryotic translation initiation factor 3 subunit J	236087	24042886
P27708	CAD protein	236336	22537154
P61158	Actin-related protein 3	240831	18332340
Q6P2Q9	Pre-mRNA-processing-splicing factor 8	246133	120534530
O60216	Double-strand-break repair protein rad21 homolog	248407	37343080
P62316	Small nuclear ribonucleoprotein Sm D2	251853	109492620
Q9Y5B9	FACT complex subunit SPT16	252680	71982390
Q8NC51	SERPINE1 mRNA-binding protein 1	252906	66222150
Q9BUQ8	Probable ATP-dependent RNA helicase DDX23	255098	28931356
Q92616	Stalled ribosome sensor GCN1	256590	73993760
Q9NZI8	Insulin-like growth factor 2 mRNA-binding protein 1	256983	485286430
Q15181	Inorganic pyrophosphatase	257128	44552190
Q9BQA1	Methylosome protein WDR77	262697	23139746
P63173	Large ribosomal subunit protein eL38	263768	177409900
O94776	Metastasis-associated protein MTA2	265089	45485696
Q9BUF5	Tubulin beta-6 chain	265157	24158552
Q9Y3I0	RNA-splicing ligase RtcB homolog	266972	124976610
P62244	Small ribosomal subunit protein uS8	274314	170981280
Q9P258	Protein RCC2	275870	47127188
Q9NPD3	Exosome complex component RRP41	278397	27858764
P46060	Ran GTPase-activating protein 1	283782	71690180
Q13151	Heterogeneous nuclear ribonucleoprotein A0	287583	110367780
Q9NWH9	SAFB-like transcription modulator	292595	16755553
Q9P035	Very-long-chain (3R)-3-hydroxyacyl-CoA dehydratase 3	292636	8243315
Q99832	T-complex protein 1 subunit eta	297339	13405902
P58107	Epiplakin	298722	8293452
P11387	DNA topoisomerase 1	299822	344413630
Q8NI27	THO complex subunit 2	300879	24318824
P62081	Small ribosomal subunit protein eS7	304633	112424130
Q96HC4	PDZ and LIM domain protein 5	309471	137522180
Q03252	Lamin-B2	309618	161326910

O75643	U5 small nuclear ribonucleoprotein 200 kDa helicase	310481	215256000
P46783	Small ribosomal subunit protein eS10	314262	65745188
Q9H0A0	RNA cytidine acetyltransferase	318097	164125870
P17987	T-complex protein 1 subunit alpha	327435	25989900
P63244	Small ribosomal subunit protein RACK1	331824	199082900
P52597	Heterogeneous nuclear ribonucleoprotein F	337107	282725300
Q9NVI7	ATPase family AAA domain-containing protein 3A	348354	71863704
P63220	Small ribosomal subunit protein eS21	350675	19085836
P41091	Eukaryotic translation initiation factor 2 subunit 3	355857	27704168
P32969	Large ribosomal subunit protein uL6	362754	166831200
O76021	Ribosomal L1 domain-containing protein 1	375627	78008590
P25398	Small ribosomal subunit protein eS12	382655	173308740
O60684	Importin subunit alpha-7	388568	32264152
P33993	DNA replication licensing factor MCM7	391510	155904140
P08621	U1 small nuclear ribonucleoprotein 70 kDa	392599	96683750
Q9UNE7	E3 ubiquitin-protein ligase CHIP	392875	54515104
P35250	Replication factor C subunit 2	397129	39230550
P62888	Large ribosomal subunit protein eL30	398759	91623910
Q92945	Far upstream element-binding protein 2	399587	13403930
Q96EY1	DnaJ homolog subfamily A member 3, mitochondrial	411394	21080690
P38919	Eukaryotic initiation factor 4A-III	414132	152224210
Q09028	Histone-binding protein RBBP4	414872	139730720
P37108	Signal recognition particle 14 kDa protein	417701	35053016
Q16629	Serine/arginine-rich splicing factor 7	422076	462540580
O00159	Unconventional myosin-Ic	428486	34544650
P31942	Heterogeneous nuclear ribonucleoprotein H3	438059	88866056
Q9Y2X3	Nucleolar protein 58	442137	297893150
Q99848	Probable rRNA-processing protein EBP2	445227	87474640
P62906	Large ribosomal subunit protein uL1	445553	155518770
Q02978	Mitochondrial 2-oxoglutarate/malate carrier protein	454954	23142188
P49792	E3 SUMO-protein ligase RanBP2	457994	127542904
P56537	Eukaryotic translation initiation factor 6	463806	41968470
P56192	Methionine--tRNA ligase, cytoplasmic	480091	7854175
P53396	ATP-citrate synthase	482653	26527648
P62701	Small ribosomal subunit protein eS4, X isoform	493098	116780536
P59998	Actin-related protein 2/3 complex subunit 4	502956	6152125
Q13162	Peroxiredoxin-4	503461	10595198
Q13547	Histone deacetylase 1	506696	52211132
P38159	RNA-binding motif protein, X chromosome	510089	175619540
P50991	T-complex protein 1 subunit delta	510481	12431207
O43175	D-3-phosphoglycerate dehydrogenase	511039	39943896
P46781	Small ribosomal subunit protein uS4	521776	277274750
P15924	Desmoplakin	523888	31995040
Q96HS1	Serine/threonine-protein phosphatase PGAM5, mitochondrial	532457	123424040
P39687	Acidic leucine-rich nuclear phosphoprotein 32 family member A	534008	57829770
O75533	Splicing factor 3B subunit 1	535681	91157970
Q9BZE4	GTP-binding protein 4	542750	213748220
P20700	Lamin-B1	545062	509208580

O43143	ATP-dependent RNA helicase DHX15	550217	125668680
Q15366	Poly(rC)-binding protein 2	554145	236249630
Q9UKD2	mRNA turnover protein 4 homolog	557270	79501976
P49458	Signal recognition particle 9 kDa protein	560618	57592930
P60866	Small ribosomal subunit protein uS10	565080	95042390
P62304	Small nuclear ribonucleoprotein E	584261	46749936
Q08945	FACT complex subunit SSRP1	588190	102365064
Q9Y277	Voltage-dependent anion-selective channel protein 3	597923	25200926
P13647	Keratin, type II cytoskeletal 5	604626	179589540
P78371	T-complex protein 1 subunit beta	607922	5944519
P62829	Large ribosomal subunit protein uL14	608209	213682340
Q9Y314	Nitric oxide synthase-interacting protein	630794	51551040
P62857	Small ribosomal subunit protein eS28	644857	76730010
Q04837	Single-stranded DNA-binding protein, mitochondrial	645692	79081580
P63104	14-3-3 protein zeta/delta	651224	10932328
O75367	Core histone macro-H2A.1	652361	696525250
P12956	X-ray repair cross-complementing protein 6	683502	50592530
P62851	Small ribosomal subunit protein eS25	691486	140764820
P35580	Myosin-10	701043	17220980
P46087	Probable 28S rRNA (cytosine(4447)-C(5))-methyltransferase	703171	81776430
P13010	X-ray repair cross-complementing protein 5	708222	29528176
P05387	Large ribosomal subunit protein P2	709037	127759460
O43390	Heterogeneous nuclear ribonucleoprotein R	712117	140124750
Q12906	Interleukin enhancer-binding factor 3	718496	166352290
Q9Y3U8	Large ribosomal subunit protein eL36	721938	143368580
Q96PK6	RNA-binding protein 14	723031	79766660
P83731	Large ribosomal subunit protein eL24	723593	460748900
P39019	Small ribosomal subunit protein eS19	735950	142374340
P62249	Small ribosomal subunit protein uS9	758256	230038750
P60981	Destrin	770996	85027630
Q15393	Splicing factor 3B subunit 3	779190	175475950
O14979	Heterogeneous nuclear ribonucleoprotein D-like	798846	100911224
P36578	Large ribosomal subunit protein uL4	804355	589067650
P15880	Small ribosomal subunit protein uS5	821689	86660240
P55084	Trifunctional enzyme subunit beta, mitochondrial	829191	30056426
Q09666	Neuroblast differentiation-associated protein AHNAK	852963	15428133
P50914	Large ribosomal subunit protein eL14	863531	272596160
P09661	U2 small nuclear ribonucleoprotein A'	865083	42583684
Q08J23	RNA cytosine C(5)-methyltransferase NSUN2	868731	55143556
P67809	Y-box-binding protein 1	870717	46547616
P42166	Lamina-associated polypeptide 2, isoform alpha	878953	68902110
P51991	Heterogeneous nuclear ribonucleoprotein A3	886045	106258580
P13489	Ribonuclease inhibitor	887331	53775450
O95831	Apoptosis-inducing factor 1, mitochondrial	889215	209597600
P62277	Small ribosomal subunit protein uS15	909428	214020820
Q92804	TATA-binding protein-associated factor 2N	977591	22001870
P62318	Small nuclear ribonucleoprotein Sm D3	1026031	129640210
P62424	Large ribosomal subunit protein eL8	1138524	159208860
P05388	Large ribosomal subunit protein uL10	1207220	159417040
P84103	Serine/arginine-rich splicing factor 3	1210298	508740130
Q15149	Plectin	1291438	25990258

Q08211	ATP-dependent RNA helicase A	1317957	510906940
Q9NR30	Nucleolar RNA helicase 2	1320199	147585620
P30050	Large ribosomal subunit protein uL11	1327847	96498580
P62750	Large ribosomal subunit protein uL23	1339832	269617570
P78527	DNA-dependent protein kinase catalytic subunit	1355429	36120650
P12236	ADP/ATP translocase 3	1372374	248536750
Q96AG4	Leucine-rich repeat-containing protein 59	1494096	18756822
Q15365	Poly(rC)-binding protein 1	1509772	150977540
P62753	Small ribosomal subunit protein eS6	1544959	173497000
P08865	Small ribosomal subunit protein uS2	1579663	175824320
P18124	Large ribosomal subunit protein uL30	1663222	162823200
P49411	Elongation factor Tu, mitochondrial	1675321	11093842
P26373	Large ribosomal subunit protein eL13	1679703	367582080
P13639	Elongation factor 2	1726642	81562616
P49327	Fatty acid synthase	1847916	22205172
Q13885	Tubulin beta-2A chain	1951100	106437040
P35908	Keratin, type II cytoskeletal 2 epidermal	2009943	108251100
P21333	Filamin-A	2070630	60541024
P23396	Small ribosomal subunit protein uS3	2127764	324482850
P62241	Small ribosomal subunit protein eS8	2215151	126713224
P21796	Voltage-dependent anion-selective channel protein 1	2261704	11926175
P55072	Transitional endoplasmic reticulum ATPase	2438732	28738310
P62269	Small ribosomal subunit protein uS13	2461380	75344450
P31943	Heterogeneous nuclear ribonucleoprotein H	2534678	173830400
P31946	14-3-3 protein beta/alpha	2583265	42215988
P26641	Elongation factor 1-gamma	2812498	22770274
P11142	Heat shock cognate 71 kDa protein	2874122	257096200
P35527	Keratin, type I cytoskeletal 9	3000596	48182740
P19338	Nucleolin	3126774	145490370
Q00839	Heterogeneous nuclear ribonucleoprotein U	3340509	158741000
P62937	Peptidyl-prolyl cis-trans isomerase A	3383709	43231940
P02768	Albumin	3423357	608216100
P06733	Alpha-enolase	3782656	17200144
P52272	Heterogeneous nuclear ribonucleoprotein M	4216910	317270880
P07355	Annexin A2	4733508	27610750
P11021	Endoplasmic reticulum chaperone BiP	4890100	39058444
P25705	ATP synthase subunit alpha, mitochondrial	5156441	4889025
Q06830	Peroxiredoxin-1	5249662	96104856
P05141	ADP/ATP translocase 2	5618726	62734600
P08670	Vimentin	9765065	820425660
P62979	Ubiquitin-ribosomal protein eS31 fusion protein	12399681	910149440
P62805	Histone H4	61443292	4371634700
