# Supplementary material for: Prion‐Like Protein LENG8‐Mediated Nucleation Drives Stress Granule Assembly
Source: Adv Sci (Weinh). 2026 Sep 8:e77622. Online ahead of print. doi: 10.1002/advs.77622 (PMC13554387; doi:10.1002/advs.77622)
Supplement: Supplementary file 3 [file ADVS-9999-e77622-s005.pdf]

**Table S2 Differences in endogenous LENG8-interacting SG-associated proteins under basal and arsenite stress conditions.**

**IP Intensity: Protein abundance was quantified using label-free protein intensity values obtained from MSFragger-based analysis.**

| Protein Names | Ctrl_LENG8 (IP Intensity) | As_LENG8 (IP Intensity) |
|---|---|---|
| FAM98B | 0 | 68107240 |
| CSTF2 | 0 | 26210088 |
| CARHSP1 | 0 | 20597144 |
| RANBP2 | 0 | 14115852 |
| CAPG | 0 | 9522636 |
| TIA1 | 0 | 4246929 |
| IPO4 | 0 | 3449938 |
| SUGT1 | 0 | 3092149 |
| CELF1 | 0 | 1653292 |
| TAF6 | 0 | 1491175 |
| CPSF4 | 90843528 | 80374056 |
| EIF2S2 | 2001279 | 52961576 |
| G3BP1 | 86735496 | 46924976 |
| EIF2A | 5235655 | 11123147 |
| PCNA | 2985845 | 8794884 |
| PABPN1 | 1974262 | 5497353 |
| G3BP2 | 20409268 | 5402420 |
| USP10 | 20636968 | 1450273 |
| RNF216 | 37595760 | 0 |
| ATXN2 | 5877505 | 0 |
| RPS27L | 5190563 | 0 |

## Table S2 Differences in endogenous LENG8-interacting total proteins under basal and arsenite stress conditions.

**IP Intensity: Protein abundance was quantified using label-free protein intensity values obtained from MSFragger-based analysis.**

| Protein ID | Protein Names | Ctrl_LENG8 (IP Intensity) | As_LENG8 (IP Intensity) | Description |
|---|---|---|---|---|
| A0A075B6H7 | IGKV3-7 | 17232754 | 1920086 | Probable non-functional |
| A0A075B6S2 | IGKV2D-29 | 995611260 | 1866355580 | Immunoglobulin kappa variable 2D-29 |
| A0A075B6S5 | IGKV1-27 | 10762060 | 15312786 | Immunoglobulin kappa variable 1-27 |
| A0A0B4J2D9 | IGKV1D-13 | 10363777 | 9902258 | Immunoglobulin kappa variable 1D-13 |
| A0A0C4DH72 | IGKV1-6 | 17119750 | 0 | Immunoglobulin kappa variable 1-6 |
| A0A0G2JRQ6 | | 61129116 | 80625184 | Ig-like domain-containing protein (Fragment) |
| A0M8Q6 | IGLC7 | 4531593 | 5549122 | Immunoglobulin lambda constant 7 |
| A3KMH1 | VWA8 | 0 | 2296105 | von Willebrand factor A domain-containing protein 8 |
| A6NHG4 | DDTL | 9650086 | 3106443 | D-dopachrome decarboxylase-like protein |
| A8K2U0 | A2ML1 | 30300408 | 6470843 | Alpha-2-macroglobulin-like protein 1 |
| A8MV65 | VGLL3 | 32170076 | 0 | Transcription cofactor vestigial-like protein 3 |
| A8MWD9 | SNRPGP15 | 0 | 1676513 | Putative small nuclear ribonucleoprotein G-like protein 15 |
| B3KNS4 | ERVK3-1 | 5559521 | 0 | Endogenous retrovirus group K3 member 1 |
| B5ME19 | EIF3CL | 42211868 | 10389072 | Eukaryotic translation initiation factor 3 subunit C-like protein |
| B9A064 | IGLL5 | 111639664 | 29040500 | Immunoglobulin lambda-like polypeptide 5 |
| E9PAV3 | NACA | 2835295 | 3766514 | Nascent polypeptide-associated complex subunit alpha, muscle-specific form |
| E9PRG8 | C11orf98 | 7157170 | 8313442 | Uncharacterized protein C11orf98 |
| O00148 | DDX39A | 2666747 | 9420594 | ATP-dependent RNA helicase DDX39A |
| O00159 | MYO1C | 458173184 | 307084768 | Unconventional myosin-Ic |

| | | | | |
|---|---|---|---|---|
| O00170 | AIP | 0 | 1137712 | AH receptor-interacting protein |
| O00257 | CBX4 | 2577067 | 1242287 | E3 SUMO-protein ligase CBX4 |
| O00264 | PGRMC1 | 5675991 | 5565520 | Membrane-associated progesterone receptor component 1 |
| O00267 | SUPT5H | 3980592 | 913944 | Transcription elongation factor SPT5 |
| O00299 | CLIC1 | 6613242 | 5983639 | Chloride intracellular channel protein 1 |
| O00303 | EIF3F | 54247992 | 11744087 | Eukaryotic translation initiation factor 3 subunit F |
| O00330 | PDHX | 0 | 1310332 | Pyruvate dehydrogenase protein X component, mitochondrial |
| O00411 | POLRMT | 3858581 | 24673816 | DNA-directed RNA polymerase, mitochondrial |
| O00425 | IGF2BP3 | 59297352 | 265077072 | Insulin-like growth factor 2 mRNA-binding protein 3 |
| O00443 | PIK3C2A | 11840189 | 0 | Phosphatidylinositol 4-phosphate 3-kinase C2 domain-containing subunit alpha |
| O00541 | PES1 | 7768158 | 15580778 | Pescadillo homolog |
| O00560 | SDCBP | 16776258 | 17343480 | Syntenin-1 |
| O00567 | NOP56 | 10586454 | 17695194 | Nucleolar protein 56 |
| O00571 | DDX3X | 351083904 | 531561824 | ATP-dependent RNA helicase DDX3X |
| O00622 | CCN1 | 0 | 2147023 | CCN family member 1 |
| O00712 | NFIB | 10667706 | 11004296 | Nuclear factor 1 B-type |
| O00767 | SCD | 2721585 | 4606513 | Stearoyl-CoA desaturase |
| O14545 | TRAFD1 | 4731972 | 1766827 | TRAF-type zinc finger domain-containing protein 1 |
| O14641 | DVL2 | 1522322 | 0 | Segment polarity protein dishevelled homolog DVL-2 |
| O14732 | IMPA2 | 0 | 4924959 | Inositol monophosphatase 2 |
| O14744 | PRMT5 | 6037242 | 24511320 | Protein arginine N-methyltransferase 5 |
| O14818 | PSMA7 | 6192044 | 7107896 | Proteasome subunit alpha type-7 |
| O14880 | MGST3 | 0 | 1877982 | Glutathione S-transferase 3, mitochondrial |
| O14929 | HAT1 | 0 | 1393164 | Histone acetyltransferase type B catalytic subunit |

| | | | | |
|---|---|---|---|---|
| O14950 | MYL12B | 1550494 | 22451520 | Myosin regulatory light chain 12B |
| O14965 | AURKA | 0 | 4305684 | Aurora kinase A |
| O14979 | HNRNPDL | 6761596 | 178677104 | Heterogeneous nuclear ribonucleoprotein D-like |
| O14983 | ATP2A1 | 0 | 18278958 | Sarcoplasmic/endoplasmic reticulum calcium ATPase 1 |
| O15014 | ZNF609 | 17672986 | 1226058 | Zinc finger protein 609 |
| O15027 | SEC16A | 2226174460 | 506552416 | Protein transport protein Sec16A |
| O15042 | U2SURP | 145692656 | 96484760 | U2 snRNP-associated SURP motif-containing protein |
| O15078 | CEP290 | 1929267 | 0 | Centrosomal protein of 290 kDa |
| O15084 | ANKRD28 | 3417564 | 1220940 | Serine/threonine-protein phosphatase 6 regulatory ankyrin repeat subunit A |
| O15111 | CHUK | 0 | 836084 | Inhibitor of nuclear factor kappa-B kinase subunit alpha |
| O15143 | ARPC1B | 0 | 13403536 | Actin-related protein 2/3 complex subunit 1B |
| O15144 | ARPC2 | 675333 | 0 | Actin-related protein 2/3 complex subunit 2 |
| O15169 | AXIN1 | 4835251 | 2557934 | Axin-1 |
| O15173 | PGRMC2 | 1338074 | 0 | Membrane-associated progesterone receptor component 2 |
| O15226 | NKRF | 3213491 | 2633791 | NF-kappa-B-repressing factor |
| O15234 | CASC3 | 6962365 | 0 | Protein CASC3 |
| O15264 | MAPK13 | 449060 | 1029841 | Mitogen-activated protein kinase 13 |
| O15269 | SPTLC1 | 0 | 314666 | Serine palmitoyltransferase 1 |
| O15357 | INPPL1 | 5399698 | 2185755 | Phosphatidylinositol 3,4,5-trisphosphate 5-phosphatase 2 |
| O15371 | EIF3D | 34263344 | 21877814 | Eukaryotic translation initiation factor 3 subunit D |
| O15372 | EIF3H | 22948240 | 9846020 | Eukaryotic translation initiation factor 3 subunit H |
| O15427 | SLC16A3 | 0 | 2834097 | Monocarboxylate transporter 4 |
| O15541 | RNF113A | 6768349 | 6841983 | E3 ubiquitin-protein ligase RNF113A |
| O43143 | DHX15 | 103003376 | 97334376 | ATP-dependent RNA helicase DHX15 |

| | | | | |
|---|---|---|---|---|
| O43159 | RRP8 | 9338330 | 0 | Ribosomal RNA-processing protein 8 |
| O43172 | PRPF4 | 2002130 | 11037280 | U4/U6 small nuclear ribonucleoprotein Prp4 |
| O43175 | PHGDH | 15821798 | 41947400 | D-3-phosphoglycerate dehydrogenase |
| O43242 | PSMD3 | 0 | 6066123 | 26S proteasome non-ATPase regulatory subunit 3 |
| O43251 | RBFOX2 | 0 | 2133187 | RNA binding protein fox-1 homolog 2 |
| O43290 | SART1 | 1913808 | 10467679 | U4/U6.U5 tri-snRNP-associated protein 1 |
| O43298 | ZBTB43 | 6818052 | 690861 | Zinc finger and BTB domain-containing protein 43 |
| O43324 | EEF1E1 | 2424823 | 4583052 | Eukaryotic translation elongation factor 1 epsilon-1 |
| O43390 | HNRNPR | 280486784 | 1423404670 | Heterogeneous nuclear ribonucleoprotein R |
| O43395 | PRPF3 | 87193696 | 24545930 | U4/U6 small nuclear ribonucleoprotein Prp3 |
| O43396 | TXNL1 | 0 | 2646916 | Thioredoxin-like protein 1 |
| O43432 | EIF4G3 | 0 | 1562281 | Eukaryotic translation initiation factor 4 gamma 3 |
| O43474 | KLF4 | 2261340 | 0 | Krueppel-like factor 4 |
| O43513 | MED7 | 7692772 | 6081800 | Mediator of RNA polymerase II transcription subunit 7 |
| O43670 | ZNF207 | 0 | 1654796 | BUB3-interacting and GLEBS motif-containing protein ZNF207 |
| O43684 | BUB3 | 0 | 2447408 | Mitotic checkpoint protein BUB3 |
| O43707 | ACTN4 | 7197907 | 28661066 | Alpha-actinin-4 |
| O43719 | HTATSF1 | 0 | 4453752 | HIV Tat-specific factor 1 |
| O43760 | SYNGR2 | 2919265 | 4002893 | Synaptogyrin-2 |
| O43790 | KRT86 | 53729960 | 6135374 | Keratin, type II cuticular Hb6 |
| O43795 | MYO1B | 127499504 | 22980208 | Unconventional myosin-Ib |
| O43809 | NUDT21 | 996030400 | 1562663680 | Cleavage and polyadenylation specificity factor subunit 5 |
| O43813 | LANCL1 | 0 | 605769 | Glutathione S-transferase LANCL1 |
| O43823 | AKAP8 | 171282368 | 84691632 | A-kinase anchor protein 8 |
| O43852 | CALU | 392318016 | 543456900 | Calumenin |
| O43920 | NDUFS5 | 9932966 | 9901328 | NADH dehydrogenase [ubiquinone] iron-sulfur protein 5 |

| | | | | |
|---|---|---|---|---|
| O60232 | ZNRD2 | 2684482 | 0 | Protein ZNRD2 |
| O60244 | MED14 | 16829120 | 9992576 | Mediator of RNA polymerase II transcription subunit 14 |
| O60264 | SMARCA5 | 8593239 | 47039880 | SWI/SNF-related matrix-associated actin-dependent regulator of chromatin subfamily A member 5 |
| O60293 | ZFC3H1 | 94195464 | 22358788 | Zinc finger C3H1 domain-containing protein |
| O60341 | KDM1A | 5564109 | 0 | Lysine-specific histone demethylase 1A |
| O60506 | SYNCRIP | 106341984 | 200259008 | Heterogeneous nuclear ribonucleoprotein Q |
| O60563 | CCNT1 | 159363 | 899001 | Cyclin-T1 |
| O60573 | EIF4E2 | 8953410 | 0 | Eukaryotic translation initiation factor 4E type 2 |
| O60684 | KPNA6 | 0 | 3126404 | Importin subunit alpha-7 |
| O60716 | CTNND1 | 3793574 | 16257802 | Catenin delta-1 |
| O60762 | DPM1 | 5057088 | 5402214 | Dolichol-phosphate mannosyltransferase subunit 1 |
| O60763 | USO1 | 1259686 | 1324610 | General vesicular transport factor p115 |
| O60814 | H2BC12 | 158537568 | 676362820 | Histone H2B type 1-K |
| O60828 | PQBP1 | 0 | 2409320 | Polyglutamine-binding protein 1 |
| O60832 | DKC1 | 3871092 | 9937931 | H/ACA ribonucleoprotein complex subunit DKC1 |
| O60841 | EIF5B | 1574002 | 0 | Eukaryotic translation initiation factor 5B |
| O75083 | WDR1 | 1942367 | 3960216 | WD repeat-containing protein 1 |
| O75131 | CPNE3 | 0 | 1749892 | Copine-3 |
| O75145 | PPFIA3 | 7787442 | 751931 | Liprin-alpha-3 |
| O75152 | ZC3H11A | 3677234 | 20405956 | Zinc finger CCCH domain-containing protein 11A |
| O75165 | DNAJC13 | 10200532 | 97753832 | DnaJ homolog subfamily C member 13 |
| O75179 | ANKRD17 | 12720687 | 0 | Ankyrin repeat domain-containing protein 17 |
| O75223 | GGCT | 3782522 | 9804850 | Gamma-glutamylcyclotransferase |
| O75251 | NDUFS7 | 2096698 | 39535508 | NADH dehydrogenase [ubiquinone] iron-sulfur protein 7, mitochondrial |

| | | | | |
|---|---|---|---|---|
| O75323 | NIPSNAP2 | 0 | 6168261 | Protein NipSnap homolog 2 |
| O75340 | PDCD6 | 0 | 1330203 | Programmed cell death protein 6 |
| O75342 | ALOX12B | 15451977 | 14993970 | Arachidonate 12-lipoxygenase, 12R-type |
| O75367 | MACROH2A1 | 0 | 3105253 | Core histone macro-H2A.1 |
| O75369 | FLNB | 1240728 | 17203082 | Filamin-B |
| O75376 | NCOR1 | 1152564 | 4631136 | Nuclear receptor corepressor 1 |
| O75381 | PEX14 | 0 | 5740400 | Peroxisomal membrane protein PEX14 |
| O75390 | CS | 1043512 | 2283886 | Citrate synthase, mitochondrial |
| O75400 | PRPF40A | 0 | 9590853 | Pre-mRNA-processing factor 40 homolog A |
| O75420 | GIGYF1 | 2923964 | 0 | GRB10-interacting GYF protein 1 |
| O75436 | VPS26A | 0 | 4785130 | Vacuolar protein sorting-associated protein 26A |
| O75448 | MED24 | 4129986 | 1711193 | Mediator of RNA polymerase II transcription subunit 24 |
| O75477 | ERLIN1 | 0 | 2339733 | Erlin-1 |
| O75489 | NDUFS3 | 4089928 | 3900368 | NADH dehydrogenase [ubiquinone] iron-sulfur protein 3, mitochondrial |
| O75494 | SRSF10 | 0 | 30032766 | Serine/arginine-rich splicing factor 10 |
| O75531 | BANF1 | 36320824 | 26521772 | Barrier-to-autointegration factor |
| O75533 | SF3B1 | 5979085 | 124667848 | Splicing factor 3B subunit 1 |
| O75534 | CSDE1 | 12420570 | 387992288 | Cold shock domain-containing protein E1 |
| O75554 | WBP4 | 0 | 754657 | WW domain-binding protein 4 |
| O75569 | PRKRA | 1993310 | 0 | Interferon-inducible double-stranded RNA-dependent protein kinase activator A |
| O75586 | MED6 | 4168298 | 1061047 | Mediator of RNA polymerase II transcription subunit 6 |
| O75635 | SERPINB7 | 0 | 910015 | Serpin B7 |
| O75643 | SNRNP200 | 12459813 | 31154460 | U5 small nuclear ribonucleoprotein 200 kDa helicase |
| O75683 | SURF6 | 2016335 | 0 | Surfeit locus protein 6 |

| | | | | |
|---|---|---|---|---|
| O75694 | NUP155 | 4350921 | 5243918 | Nuclear pore complex protein Nup155 |
| O75715 | GPX5 | 21345868 | 22976224 | Epididymal secretory glutathione peroxidase |
| O75717 | WDHD1 | 0 | 7272113 | WD repeat and HMG-box DNA-binding protein 1 |
| O75821 | EIF3G | 15438999 | 5243670 | Eukaryotic translation initiation factor 3 subunit G |
| O75822 | EIF3J | 67828272 | 37224280 | Eukaryotic translation initiation factor 3 subunit J |
| O75874 | IDH1 | 2411021 | 3986393 | Isocitrate dehydrogenase [NADP] cytoplasmic |
| O75909 | CCNK | 1252745 | 1900861 | Cyclin-K |
| O75947 | ATP5PD | 0 | 198635 | ATP synthase subunit d, mitochondrial |
| O76021 | RSL1D1 | 43924300 | 21035656 | Ribosomal L1 domain-containing protein 1 |
| O76094 | SRP72 | 27748672 | 0 | Signal recognition particle subunit SRP72 |
| O82803 | SRPP | 19158726 | 26705466 | Small rubber particle protein |
| O94762 | RECQL5 | 4038126 | 0 | ATP-dependent DNA helicase Q5 |
| O94776 | MTA2 | 15335550 | 56890696 | Metastasis-associated protein MTA2 |
| O94842 | TOX4 | 162077280 | 81780432 | TOX high mobility group box family member 4 |
| O94875 | SORBS2 | 16318896 | 26189246 | Sorbin and SH3 domain-containing protein 2 |
| O94880 | PHF14 | 20529894 | 8628058 | PHD finger protein 14 |
| O94906 | PRPF6 | 0 | 2609936 | Pre-mRNA-processing factor 6 |
| O94913 | PCF11 | 2390697 | 966008 | Pre-mRNA cleavage complex 2 protein Pcf11 |
| O94986 | CEP152 | 3347270660 | 0 | Centrosomal protein of 152 kDa |
| O94992 | HEXIM1 | 1975106 | 2306539 | Protein HEXIM1 |
| O95049 | TJP3 | 1762320 | 0 | Tight junction protein ZO-3 |
| O95071 | UBR5 | 68634704 | 61652140 | E3 ubiquitin-protein ligase UBR5 |
| O95218 | ZRANB2 | 91519728 | 102691152 | Zinc finger Ran-binding domain-containing protein 2 |
| O95232 | LUC7L3 | 3047068 | 10405689 | Luc7-like protein 3 |
| O95239 | KIF4A | 67846136 | 44053384 | Chromosome-associated kinesin KIF4A |

| | | | | |
|---|---|---|---|---|
| O95292 | VAPB | 0 | 1759254 | Vesicle-associated membrane protein-associated protein B/C |
| O95299 | NDUFA10 | 1429112 | 5156095 | NADH dehydrogenase [ubiquinone] 1 alpha subcomplex subunit 10, mitochondrial |
| O95347 | SMC2 | 2423740 | 4694415 | Structural maintenance of chromosomes protein 2 |
| O95359 | TACC2 | 0 | 3289927 | Transforming acidic coiled-coil-containing protein 2 |
| O95373 | IPO7 | 937391 | 66830328 | Importin-7 |
| O95391 | SLU7 | 2477412 | 4738232 | Pre-mRNA-splicing factor SLU7 |
| O95402 | MED26 | 24197312 | 787124 | Mediator of RNA polymerase II transcription subunit 26 |
| O95429 | BAG4 | 14731078 | 1790499 | BAG family molecular chaperone regulator 4 |
| O95551 | TDP2 | 59268540 | 25923198 | Tyrosyl-DNA phosphodiesterase 2 |
| O95639 | CPSF4 | 90843528 | 80374056 | Cleavage and polyadenylation specificity factor subunit 4 |
| O95714 | HERC2 | 26115960 | 2291303 | E3 ubiquitin-protein ligase HERC2 |
| O95757 | HSPA4L | 0 | 778092 | Heat shock 70 kDa protein 4L |
| O95785 | WIZ | 18159340 | 10588406 | Protein Wiz |
| O95816 | BAG2 | 32536314 | 35798648 | BAG family molecular chaperone regulator 2 |
| O95817 | BAG3 | 0 | 2637988 | BAG family molecular chaperone regulator 3 |
| O95831 | AIFM1 | 28818154 | 91775024 | Apoptosis-inducing factor 1, mitochondrial |
| O96019 | ACTL6A | 1055469 | 6919451 | Actin-like protein 6A |
| P00338 | LDHA | 27612748 | 41605648 | L-lactate dehydrogenase A chain |
| P00366 | GLUD1 | 0 | 1390792 | Glutamate dehydrogenase 1, mitochondrial |
| P00387 | CYB5R3 | 0 | 847687 | NADH-cytochrome b5 reductase 3 |
| P00403 | MT-CO2 | 1872312 | 4090289 | Cytochrome c oxidase subunit 2 |
| P00441 | SOD1 | 0 | 4492498 | Superoxide dismutase [Cu-Zn] |
| P00450 | CP | 21722060 | 13495720 | Ceruloplasmin |

| | | | | |
|---|---|---|---|---|
| P00491 | PNP | 3211131 | 2043008 | Purine nucleoside phosphorylase |
| P00505 | GOT2 | 0 | 67327360 | Aspartate aminotransferase, mitochondrial |
| P00558 | PGK1 | 17166646 | 35240212 | Phosphoglycerate kinase 1 |
| P00736 | C1R | 64751076 | 33144264 | Complement C1r subcomponent |
| P00761 | | 20947249200 | 14678175700 | Trypsin |
| P00918 | CA2 | 2014378 | 2014379 | Carbonic anhydrase 2 |
| P00966 | ASS1 | 11042311 | 14950966 | Argininosuccinate synthase |
| P01008 | SERPINC1 | 24716810 | 29324648 | Antithrombin-III |
| P01023 | A2M | 1941111 | 4228845 | Alpha-2-macroglobulin |
| P01024 | C3 | 315667008 | 299050048 | Complement C3 |
| P01031 | C5 | 8945180 | 8149538 | Complement C5 |
| P01040 | CSTA | 53959624 | 61505872 | Cystatin-A |
| P01593 | IGKV1D-33 | 4310206 | 4823495 | Immunoglobulin kappa variable 1D-33 |
| P01817 | IGHV2-5 | 127557048 | 0 | Immunoglobulin heavy variable 2-5 |
| P01834 | IGKC | 1484788 | 1029153 | Immunoglobulin kappa constant |
| P01857 | IGHG1 | 17386045400 | 16166116400 | Immunoglobulin heavy constant gamma 1 |
| P01876 | IGHA1 | 4842648 | 5951679 | Immunoglobulin heavy constant alpha 1 |
| P02452 | COL1A1 | 0 | 4500670 | Collagen alpha-1(I) chain |
| P02461 | COL3A1 | 2281525 | 3631691 | Collagen alpha-1(III) chain |
| P02533 | KRT14 | 1676460290 | 1388529280 | Keratin, type I cytoskeletal 14 |
| P02538 | KRT6A | 1090918270 | 259650832 | Keratin, type II cytoskeletal 6A |
| P02545 | LMNA | 52512420 | 160162048 | Prelamin-A/C |
| P02656 | APOC3 | 14461936 | 16372048 | Apolipoprotein C-III |
| P02662 | CSN1S1 | 123001296 | 31244960 | Alpha-S1-casein |
| P02663 | CSN1S2 | 110526032 | 39458184 | Alpha-S2-casein |
| P02666 | CSN2 | 188343360 | 55799064 | Beta-casein |
| P02668 | CSN3 | 43383164 | 10583228 | Kappa-casein |
| P02751 | FN1 | 31303214 | 19736016 | Fibronectin |
| P02768 | ALB | 15415956 | 30115946 | Albumin |
| P02769 | ALB | 8533864400 | 10281219100 | Albumin |
| P02786 | TFRC | 0 | 7252958 | Transferrin receptor protein 1 |
| P02788 | LTF | 7132901 | 5745696 | Lactotransferrin |
| P02794 | FTH1 | 12723296 | 23185716 | Ferritin heavy chain |
| P04040 | CAT | 20325684 | 23527964 | Catalase |
| P04075 | ALDOA | 24255872 | 20117000 | Fructose-bisphosphate aldolase A |

| | | | | |
|---|---|---|---|---|
| P04080 | CSTB | 0 | 673838 | Cystatin-B |
| P04083 | ANXA1 | 12266408 | 5174455 | Annexin A1 |
| P04196 | HRG | 19823822 | 57032540 | Histidine-rich glycoprotein |
| P04259 | KRT6B | 141293696 | 24172372 | Keratin, type II cytoskeletal 6B |
| P04264 | KRT1 | 35504361000 | 37117661000 | Keratin, type II cytoskeletal 1 |
| P04406 | GAPDH | 68629720 | 83074216 | Glyceraldehyde-3-phosphate dehydrogenase |
| P04439 | HLA-A | 0 | 3107397 | HLA class I histocompatibility antigen, A alpha chain |
| P04792 | HSPB1 | 84002160 | 83546320 | Heat shock protein beta-1 |
| P04843 | RPN1 | 0 | 4034725 | Dolichyl-diphosphooligosaccharide--protein glycosyltransferase subunit 1 |
| P04844 | RPN2 | 0 | 313043 | Dolichyl-diphosphooligosaccharide--protein glycosyltransferase subunit 2 |
| P05023 | ATP1A1 | 2732193 | 6152908 | Sodium/potassium-transporting ATPase subunit alpha-1 |
| P05089 | ARG1 | 42815212 | 38075008 | Arginase-1 |
| P05109 | S100A8 | 168198672 | 24637972 | Protein S100-A8 |
| P05141 | SLC25A5 | 227492160 | 294825632 | ADP/ATP translocase 2 |
| P05156 | CFI | 2308721 | 2437767 | Complement factor I |
| P05198 | EIF2S1 | 850224 | 9675534 | Eukaryotic translation initiation factor 2 subunit 1 |
| P05386 | RPLP1 | 20487922 | 7794419 | Large ribosomal subunit protein P1 |
| P05387 | RPLP2 | 361103680 | 155154880 | Large ribosomal subunit protein P2 |
| P05388 | RPLP0 | 1756615940 | 222811328 | Large ribosomal subunit protein uL10 |
| P05455 | SSB | 0 | 976129 | Lupus La protein |
| P05549 | TFAP2A | 117187816 | 117556008 | Transcription factor AP-2-alpha |
| P05783 | KRT18 | 34226156 | 532192096 | Keratin, type I cytoskeletal 18 |
| P05787 | KRT8 | 102426336 | 676713540 | Keratin, type II cytoskeletal 8 |
| P06396 | GSN | 26148052 | 16857886 | Gelsolin |
| P06454 | PTMA | 5713626 | 28202672 | Prothymosin alpha |
| P06576 | ATP5F1B | 35728856 | 40345796 | ATP synthase subunit beta, mitochondrial |
| P06702 | S100A9 | 171717168 | 34618852 | Protein S100-A9 |
| P06703 | S100A6 | 14551623 | 13566804 | Protein S100-A6 |

| | | | | |
|---|---|---|---|---|
| P06732 | CKM | 7647428 | 2910127 | Creatine kinase M-type |
| P06733 | ENO1 | 119174328 | 65240092 | Alpha-enolase |
| P06744 | GPI | 8412280 | 1313037 | Glucose-6-phosphate isomerase |
| P06748 | NPM1 | 41979944 | 102476424 | Nucleophosmin |
| P06753 | TPM3 | 13110275 | 7840707 | Tropomyosin alpha-3 chain |
| P07195 | LDHB | 21507112 | 27520676 | L-lactate dehydrogenase B chain |
| P07237 | P4HB | 84598288 | 132639544 | Protein disulfide-isomerase |
| P07339 | CTSD | 30966520 | 18886104 | Cathepsin D |
| P07355 | ANXA2 | 76272136 | 144066768 | Annexin A2 |
| P07384 | CAPN1 | 1578022 | 3318839 | Calpain-1 catalytic subunit |
| P07437 | TUBB | 1529424000 | 544862780 | Tubulin beta chain |
| P07476 | IVL | 0 | 1114304 | Involucrin |
| P07477 | PRSS1 | 451681088 | 466044896 | Serine protease 1 |
| P07686 | HEXB | 3169882 | 1660223 | Beta-hexosaminidase subunit beta |
| P07737 | PFN1 | 10571704 | 20385610 | Profilin-1 |
| P07741 | APRT | 0 | 5475933 | Adenine phosphoribosyltransferase |
| P07814 | EPRS1 | 2448586 | 32346934 | Bifunctional glutamate/proline--tRNA ligase |
| P07900 | HSP90AA1 | 22711876 | 39263924 | Heat shock protein HSP 90-alpha |
| P07910 | HNRNPC | 60052456 | 103065856 | Heterogeneous nuclear ribonucleoproteins C1/C2 |
| P07996 | THBS1 | 15365077 | 15604177 | Thrombospondin-1 |
| P08123 | COL1A2 | 0 | 14722204 | Collagen alpha-2(I) chain |
| P08133 | ANXA6 | 0 | 1092425 | Annexin A6 |
| P08134 | RHOC | 1756939 | 1217153 | Rho-related GTP-binding protein RhoC |
| P08195 | SLC3A2 | 12787297 | 19842602 | Amino acid transporter heavy chain SLC3A2 |
| P08237 | PFKM | 3430785 | 0 | ATP-dependent 6-phosphofructokinase, muscle type |
| P08238 | HSP90AB1 | 143448256 | 483987840 | Heat shock protein HSP 90-beta |
| P08559 | PDHA1 | 1484609 | 3747430 | Pyruvate dehydrogenase E1 component subunit alpha, somatic form, mitochondrial |
| P08574 | CYC1 | 802583 | 2258363 | Cytochrome c1, heme protein, mitochondrial |
| P08621 | SNRNP70 | 18863734 | 17927468 | U1 small nuclear ribonucleoprotein 70 kDa |

| | | | | |
|---|---|---|---|---|
| P08651 | NFIC | 70914712 | 84113168 | Nuclear factor 1 C-type |
| P08670 | VIM | 84566928 | 1652985600 | Vimentin |
| P08708 | RPS17 | 67881080 | 16129905 | Small ribosomal subunit protein eS17 |
| P08729 | KRT7 | 9122077 | 48529312 | Keratin, type II cytoskeletal 7 |
| P08758 | ANXA5 | 2024859 | 1163988 | Annexin A5 |
| P08779 | KRT16 | 308114048 | 140997296 | Keratin, type I cytoskeletal 16 |
| P08842 | STS | 0 | 1072258 | Steryl-sulfatase |
| P08865 | RPSA | 440754784 | 166262944 | Small ribosomal subunit protein uS2 |
| P09012 | SNRPA | 5666316 | 5915866 | U1 small nuclear ribonucleoprotein A |
| P09211 | GSTP1 | 4664174 | 2539007 | Glutathione S-transferase P |
| P09382 | LGALS1 | 7980803 | 15980594 | Galectin-1 |
| P09496 | CLTA | 39145008 | 4714716 | Clathrin light chain A |
| P09497 | CLTB | 19167466 | 1003398 | Clathrin light chain B |
| P09651 | HNRNPA1 | 10416572 | 24824000 | Heterogeneous nuclear ribonucleoprotein A1 |
| P09669 | COX6C | 0 | 4095813 | Cytochrome c oxidase subunit 6C |
| P09874 | PARP1 | 0 | 6533834 | Poly [ADP-ribose] polymerase 1 |
| P09884 | POLA1 | 0 | 597609 | DNA polymerase alpha catalytic subunit |
| P09972 | ALDOC | 1157347 | 0 | Fructose-bisphosphate aldolase C |
| P0C0L4 | C4A | 25053964 | 28372842 | Complement C4-A |
| P0C0S5 | H2AZ1 | 3903288 | 19438984 | Histone H2A.Z |
| P0C0S8 | H2AC17 | 153012496 | 434945312 | Histone H2A type 1 |
| P0CW18 | PRSS56 | 0 | 6598009 | Serine protease 56 |
| P0DMV8 | HSPA1A | 168172736 | 189052944 | Heat shock 70 kDa protein 1A |
| P0DP23 | CALM1 | 25918126 | 21533432 | Calmodulin-1 |
| P0DTL6 | ZFTRAF1 | 0 | 103465904 | Zinc finger TRAF-type-containing protein 1 |
| P10124 | SRGN | 2537455 | 0 | Serglycin |
| P10412 | H1-4 | 217603 | 4190188 | Histone H1.4 |
| P10515 | DLAT | 4121878 | 40624972 | Dihydrolipoyllysine-residue acetyltransferase component of pyruvate dehydrogenase complex, mitochondrial |
| P10599 | TXN | 50249324 | 37160460 | Thioredoxin |
| P10619 | CTSA | 1174447 | 0 | Lysosomal protective protein |
| P10809 | HSPD1 | 398246432 | 481440800 | 60 kDa heat shock protein, mitochondrial |

| | | | | |
|---|---|---|---|---|
| P11021 | HSPA5 | 99851000 | 73503160 | Endoplasmic reticulum chaperone BiP |
| P11142 | HSPA8 | 378736320 | 539132740 | Heat shock cognate 71 kDa protein |
| P11171 | EPB41 | 1995054 | 5028210 | Protein 4.1 |
| P11182 | DBT | 0 | 58851816 | Lipoamide acyltransferase component of branched-chain alpha-keto acid dehydrogenase complex, mitochondrial |
| P11279 | LAMP1 | 0 | 1149407 | Lysosome-associated membrane glycoprotein 1 |
| P11387 | TOP1 | 17240702 | 39447960 | DNA topoisomerase 1 |
| P11388 | TOP2A | 31197576 | 93664904 | DNA topoisomerase 2-alpha |
| P11586 | MTHFD1 | 1492824 | 1610948 | C-1-tetrahydrofolate synthase, cytoplasmic |
| P11766 | ADH5 | 5286788 | 2009826 | Alcohol dehydrogenase class-3 |
| P11940 | PABPC1 | 639922940 | 74754296 | Polyadenylate-binding protein 1 |
| P12004 | PCNA | 2985845 | 8794884 | Proliferating cell nuclear antigen |
| P12035 | KRT3 | 7825866 | 8091930 | Keratin, type II cytoskeletal 3 |
| P12081 | HARS1 | 0 | 317886 | Histidine--tRNA ligase, cytoplasmic |
| P12235 | SLC25A4 | 42564436 | 73464200 | ADP/ATP translocase 1 |
| P12236 | SLC25A6 | 12891815 | 16769021 | ADP/ATP translocase 3 |
| P12268 | IMPDH2 | 10762506 | 31588410 | Inosine-5'-monophosphate dehydrogenase 2 |
| P12270 | TPR | 0 | 1857826 | Nucleoprotein TPR |
| P12273 | PIP | 6845065 | 3340631 | Prolactin-inducible protein |
| P12277 | CKB | 16180868 | 15410153 | Creatine kinase B-type |
| P12955 | PEPD | 20004320 | 56773880 | Xaa-Pro dipeptidase |
| P12956 | XRCC6 | 2633546 | 15948004 | X-ray repair cross-complementing protein 6 |
| P13010 | XRCC5 | 1361969 | 6069937 | X-ray repair cross-complementing protein 5 |
| P13489 | RNH1 | 9647977 | 66300068 | Ribonuclease inhibitor |
| P13639 | EEF2 | 35554680 | 83203856 | Elongation factor 2 |
| P13645 | KRT10 | 30717841400 | 47358894000 | Keratin, type I cytoskeletal 10 |
| P13646 | KRT13 | 20621732 | 0 | Keratin, type I cytoskeletal 13 |
| P13647 | KRT5 | 2481695230 | 2584726020 | Keratin, type II cytoskeletal 5 |
| P13674 | P4HA1 | 30363946 | 68800440 | Prolyl 4-hydroxylase subunit alpha-1 |

| | | | | |
|---|---|---|---|---|
| P13716 | ALAD | 0 | 789543 | Delta-aminolevulinic acid dehydratase |
| P13797 | PLS3 | 5038601 | 0 | Plastin-3 |
| P13804 | ETFA | 0 | 871850 | Electron transfer flavoprotein subunit alpha, mitochondrial |
| P13861 | PRKAR2A | 9500387 | 9784839 | cAMP-dependent protein kinase type II-alpha regulatory subunit |
| P13929 | ENO3 | 2431661 | 3605209 | Beta-enolase |
| P14136 | GFAP | 3238007 | 3816523 | Glial fibrillary acidic protein |
| P14174 | MIF | 2244880 | 1747024 | Macrophage migration inhibitory factor |
| P14314 | PRKCSH | 0 | 2230327 | Glucosidase 2 subunit beta |
| P14373 | TRIM27 | 0 | 6738474 | Zinc finger protein RFP |
| P14406 | COX7A2 | 2792648 | 0 | Cytochrome c oxidase subunit 7A2, mitochondrial |
| P14618 | PKM | 65392248 | 88832248 | Pyruvate kinase PKM |
| P14625 | HSP90B1 | 10365095 | 30117606 | Endoplasmin |
| P14678 | SNRPB | 11943252 | 23364322 | Small nuclear ribonucleoprotein-associated proteins B and B' |
| P14735 | IDE | 0 | 971292 | Insulin-degrading enzyme |
| P14866 | HNRNPL | 12794482 | 231443744 | Heterogeneous nuclear ribonucleoprotein L |
| P14868 | DARS1 | 5065582 | 18525548 | Aspartate--tRNA ligase, cytoplasmic |
| P14923 | JUP | 209704624 | 301539776 | Junction plakoglobin |
| P15121 | AKR1B1 | 14405848 | 2132993 | Aldo-keto reductase family 1 member B1 |
| P15153 | RAC2 | 0 | 2184442 | Ras-related C3 botulinum toxin substrate 2 |
| P15172 | MYOD1 | 0 | 2359702 | Myoblast determination protein 1 |
| P15252 | | 499378688 | 321000032 | Rubber elongation factor protein |
| P15259 | PGAM2 | 1923634 | 2682967 | Phosphoglycerate mutase 2 |
| P15309 | ACP3 | 0 | 460108 | Prostatic acid phosphatase |
| P15311 | EZR | 3476764 | 3203552 | Ezrin |
| P15531 | NME1 | 18680968 | 16912414 | Nucleoside diphosphate kinase A |
| P15880 | RPS2 | 336330688 | 74138216 | Small ribosomal subunit protein uS5 |
| P15924 | DSP | 632748480 | 1130048380 | Desmoplakin |
| P15927 | RPA2 | 6009743 | 1054980 | Replication protein A 32 kDa subunit |

| | | | | |
|---|---|---|---|---|
| P16152 | CBR1 | 0 | 238919 | Carbonyl reductase [NADPH] 1 |
| P16401 | H1-5 | 5158795 | 98666936 | Histone H1.5 |
| P16403 | H1-2 | 114490696 | 432152064 | Histone H1.2 |
| P16435 | POR | 0 | 1138633 | NADPH--cytochrome P450 reductase |
| P16989 | YBX3 | 26856262 | 0 | Y-box-binding protein 3 |
| P17096 | HMGA1 | 0 | 6024312 | High mobility group protein HMG-I/HMG-Y |
| P17174 | GOT1 | 1159619 | 0 | Aspartate aminotransferase, cytoplasmic |
| P17480 | UBTF | 0 | 8954677 | Nucleolar transcription factor 1 |
| P17612 | PRKACA | 1311452 | 1547608 | cAMP-dependent protein kinase catalytic subunit alpha |
| P17812 | CTPS1 | 0 | 4309139 | CTP synthase 1 |
| P17844 | DDX5 | 3999986180 | 4321243600 | Probable ATP-dependent RNA helicase DDX5 |
| P17900 | GM2A | 2757211 | 1321423 | Ganglioside GM2 activator |
| P17931 | LGALS3 | 0 | 2549013 | Galectin-3 |
| P17936 | IGFBP3 | 0 | 6488963 | Insulin-like growth factor-binding protein 3 |
| P17987 | TCP1 | 4436990 | 9142847 | T-complex protein 1 subunit alpha |
| P18031 | PTPN1 | 0 | 1597986 | Tyrosine-protein phosphatase non-receptor type 1 |
| P18077 | RPL35A | 360657472 | 185632416 | Large ribosomal subunit protein eL33 |
| P18085 | ARF4 | 849329 | 6768309 | ADP-ribosylation factor 4 |
| P18124 | RPL7 | 525522112 | 266927552 | Large ribosomal subunit protein uL30 |
| P18206 | VCL | 0 | 60820888 | Vinculin |
| P18621 | RPL17 | 190991696 | 111012488 | Large ribosomal subunit protein uL22 |
| P18754 | RCC1 | 0 | 1972852 | Regulator of chromosome condensation |
| P18850 | ATF6 | 1226142 | 501823 | Cyclic AMP-dependent transcription factor ATF-6 alpha |
| P19012 | KRT15 | 10312092 | 0 | Keratin, type I cytoskeletal 15 |
| P19338 | NCL | 24877462 | 38532180 | Nucleolin |
| P19387 | POLR2C | 6717836 | 0 | DNA-directed RNA polymerase II subunit RPB3 |
| P19474 | TRIM21 | 379256032 | 114078664 | E3 ubiquitin-protein ligase TRIM21 |

| | | | | |
|---|---|---|---|---|
| P19784 | CSNK2A2 | 7166041 | 2775639 | Casein kinase II subunit alpha' |
| P20042 | EIF2S2 | 2001279 | 52961576 | Eukaryotic translation initiation factor 2 subunit 2 |
| P20073 | ANXA7 | 0 | 420042 | Annexin A7 |
| P20226 | TBP | 0 | 533239 | TATA-box-binding protein |
| P20472 | PVALB | 35420096 | 21839224 | Parvalbumin alpha |
| P20618 | PSMB1 | 5462028 | 3264029 | Proteasome subunit beta type-1 |
| P20700 | LMNB1 | 22047442 | 43268176 | Lamin-B1 |
| P20930 | FLG | 15529395 | 18128958 | Filaggrin |
| P21127 | CDK11B | 297416 | 5165261 | Cyclin-dependent kinase 11B |
| P21333 | FLNA | 2093727 | 217496240 | Filamin-A |
| P21796 | VDAC1 | 4161742 | 2631885 | Voltage-dependent anion-selective channel protein 1 |
| P21964 | COMT | 0 | 13651946 | Catechol O-methyltransferase |
| P22087 | FBL | 2318388 | 7417480 | rRNA 2'-O-methyltransferase fibrillarin |
| P22234 | PAICS | 4015238 | 4709708 | Bifunctional phosphoribosylaminoimidazole carboxylase/phosphoribosylaminoimidazole succinocarboxamide synthetase |
| P22314 | UBA1 | 2334662 | 6731121 | Ubiquitin-like modifier-activating enzyme 1 |
| P22352 | GPX3 | 38724504 | 48511012 | Glutathione peroxidase 3 |
| P22532 | SPRR2D | 0 | 1346553 | Small proline-rich protein 2D |
| P22626 | HNRNPA2B1 | 14225334 | 74883560 | Heterogeneous nuclear ribonucleoproteins A2/B1 |
| P22695 | UQCRC2 | 0 | 1113147 | Cytochrome b-c1 complex subunit 2, mitochondrial |
| P22735 | TGM1 | 48681620 | 39722984 | Protein-glutamine gamma-glutamyltransferase K |
| P23142 | FBLN1 | 8187898 | 8696136 | Fibulin-1 |
| P23193 | TCEA1 | 0 | 1862956 | Transcription elongation factor A protein 1 |
| P23246 | SFPQ | 293643648 | 485148224 | Splicing factor, proline- and glutamine-rich |
| P23258 | TUBG1 | 20366144 | 11947505 | Tubulin gamma-1 chain |
| P23284 | PPIB | 24423118 | 28981690 | Peptidyl-prolyl cis-trans isomerase B |
| P23396 | RPS3 | 619784700 | 141837152 | Small ribosomal subunit protein uS3 |

| | | | | |
|---|---|---|---|---|
| P23490 | LORICRIN | 6962308 | 12620370 | Loricrin |
| P23526 | AHCY | 9278752 | 7813985 | Adenosylhomocysteinase |
| P23528 | CFL1 | 12576806 | 63022252 | Cofilin-1 |
| P23588 | EIF4B | 8024475 | 14803840 | Eukaryotic translation initiation factor 4B |
| P24534 | EEF1B2 | 708993 | 5234143 | Elongation factor 1-beta |
| P24666 | ACP1 | 1008469 | 19243404 | Low molecular weight phosphotyrosine protein phosphatase |
| P24863 | CCNC | 2041979 | 2133471 | Cyclin-C |
| P24928 | POLR2A | 30242888 | 17492006 | DNA-directed RNA polymerase II subunit RPB1 |
| P25054 | APC | 16588238 | 0 | Adenomatous polyposis coli protein |
| P25205 | MCM3 | 0 | 28290458 | DNA replication licensing factor MCM3 |
| P25311 | AZGP1 | 21725682 | 22762146 | Zinc-alpha-2-glycoprotein |
| P25398 | RPS12 | 33334154 | 15851309 | Small ribosomal subunit protein eS12 |
| P25490 | YY1 | 0 | 1929917 | Transcriptional repressor protein YY1 |
| P25705 | ATP5F1A | 38584188 | 59331504 | ATP synthase subunit alpha, mitochondrial |
| P25786 | PSMA1 | 3657368 | 9010911 | Proteasome subunit alpha type-1 |
| P25789 | PSMA4 | 5218913 | 4430961 | Proteasome subunit alpha type-4 |
| P25815 | S100P | 5185307 | 2261982 | Protein S100-P |
| P26038 | MSN | 13631706 | 6190388 | Moesin |
| P26368 | U2AF2 | 43562732 | 59202216 | Splicing factor U2AF 65 kDa subunit |
| P26373 | RPL13 | 607765760 | 329651328 | Large ribosomal subunit protein eL13 |
| P26447 | S100A4 | 13670079 | 48960016 | Protein S100-A4 |
| P26599 | PTBP1 | 1828582 | 53945340 | Polypyrimidine tract-binding protein 1 |
| P26639 | TARS1 | 2550975 | 10584931 | Threonine--tRNA ligase 1, cytoplasmic |
| P26641 | EEF1G | 5782843 | 15309188 | Elongation factor 1-gamma |
| P27144 | AK4 | 0 | 714914 | Adenylate kinase 4, mitochondrial |
| P27348 | YWHAQ | 4185820 | 6126254 | 14-3-3 protein theta |
| P27482 | CALML3 | 781941 | 0 | Calmodulin-like protein 3 |
| P27635 | RPL10 | 171247120 | 82142320 | Large ribosomal subunit protein uL16 |

| | | | | |
|---|---|---|---|---|
| P27694 | RPA1 | 87474192 | 7016949 | Replication protein A 70 kDa DNA-binding subunit |
| P27708 | CAD | 956682 | 6676171 | CAD protein |
| P27797 | CALR | 0 | 3350264 | Calreticulin |
| P27824 | CANX | 5773498 | 13020092 | Calnexin |
| P28066 | PSMA5 | 923035 | 6292683 | Proteasome subunit alpha type-5 |
| P28070 | PSMB4 | 0 | 1820533 | Proteasome subunit beta type-4 |
| P28072 | PSMB6 | 4467427 | 6992884 | Proteasome subunit beta type-6 |
| P28074 | PSMB5 | 5585553 | 6240177 | Proteasome subunit beta type-5 |
| P28288 | ABCD3 | 2398262 | 9612134 | ATP-binding cassette sub-family D member 3 |
| P28300 | LOX | 3047589 | 1354044 | Protein-lysine 6-oxidase |
| P28799 | GRN | 9041631 | 2447860 | Progranulin |
| P28838 | LAP3 | 279755 | 604609 | Cytosol aminopeptidase |
| P29034 | S100A2 | 3171600 | 0 | Protein S100-A2 |
| P29083 | GTF2E1 | 0 | 2036704 | General transcription factor IIE subunit 1 |
| P29372 | MPG | 2516278 | 2767654 | DNA-3-methyladenine glycosylase |
| P29401 | TKT | 8031386 | 15784534 | Transketolase |
| P29508 | SERPINB3 | 43194992 | 10163497 | Serpin B3 |
| P29692 | EEF1D | 6129354 | 62349156 | Elongation factor 1-delta |
| P30041 | PRDX6 | 2707077 | 8133861 | Peroxiredoxin-6 |
| P30044 | PRDX5 | 3337827 | 5530851 | Peroxiredoxin-5, mitochondrial |
| P30048 | PRDX3 | 15014971 | 16957366 | Thioredoxin-dependent peroxide reductase, mitochondrial |
| P30050 | RPL12 | 222087808 | 115723808 | Large ribosomal subunit protein uL11 |
| P30086 | PEBP1 | 0 | 4063902 | Phosphatidylethanolamine-binding protein 1 |
| P30101 | PDIA3 | 1682325 | 17358318 | Protein disulfide-isomerase A3 |
| P30153 | PPP2R1A | 28029130 | 8640197 | Serine/threonine-protein phosphatase 2A 65 kDa regulatory subunit A alpha isoform |
| P30260 | CDC27 | 1679789 | 1357457 | Cell division cycle protein 27 homolog |
| P30291 | WEE1 | 10997279 | 0 | Wee1-like protein kinase |

| | | | | |
|---|---|---|---|---|
| P30876 | POLR2B | 8455772 | 9754998 | DNA-directed RNA polymerase II subunit RPB2 |
| P31025 | LCN1 | 7079473 | 5111316 | Lipocalin-1 |
| P31040 | SDHA | 11818056 | 40301160 | Succinate dehydrogenase [ubiquinone] flavoprotein subunit, mitochondrial |
| P31151 | S100A7 | 25471624 | 7143637 | Protein S100-A7 |
| P31152 | MAPK4 | 853906 | 0 | Mitogen-activated protein kinase 4 |
| P31327 | CPS1 | 20909980 | 25929826 | Carbamoyl-phosphate synthase [ammonia], mitochondrial |
| P31483 | TIA1 | 0 | 4246929 | Cytotoxic granule associated RNA binding protein TIA1 |
| P31939 | ATIC | 536543 | 0 | Bifunctional purine biosynthesis protein ATIC |
| P31941 | APOBEC3A | 0 | 3030452 | DNA dC->dU-editing enzyme APOBEC-3A |
| P31942 | HNRNPH3 | 35540912 | 215537424 | Heterogeneous nuclear ribonucleoprotein H3 |
| P31943 | HNRNPH1 | 151934176 | 404175264 | Heterogeneous nuclear ribonucleoprotein H |
| P31944 | CASP14 | 63979472 | 63670152 | Caspase-14 |
| P31946 | YWHAB | 1931953 | 3857728 | 14-3-3 protein beta/alpha |
| P31947 | SFN | 14211951 | 18844046 | 14-3-3 protein sigma |
| P31948 | STIP1 | 0 | 3367525 | Stress-induced-phosphoprotein 1 |
| P31949 | S100A11 | 8507138 | 10627941 | Protein S100-A11 |
| P32119 | PRDX2 | 21077276 | 45867748 | Peroxiredoxin-2 |
| P32322 | PYCR1 | 0 | 2739379 | Pyrroline-5-carboxylate reductase 1, mitochondrial |
| P32969 | RPL9P9 | 81493680 | 27945542 | Large ribosomal subunit protein uL6 |
| P33240 | CSTF2 | 0 | 26210088 | Cleavage stimulation factor subunit 2 |
| P33897 | ABCD1 | 0 | 5988540 | ATP-binding cassette sub-family D member 1 |
| P33992 | MCM5 | 4386333 | 10877090 | DNA replication licensing factor MCM5 |
| P33993 | MCM7 | 576017 | 16466274 | DNA replication licensing factor MCM7 |
| P34897 | SHMT2 | 1450387 | 14439102 | Serine hydroxymethyltransferase, mitochondrial |
| P34932 | HSPA4 | 0 | 1275773 | Heat shock 70 kDa protein 4 |
| P35030 | PRSS3 | 17743504 | 13233729 | Trypsin-3 |

| | | | | |
|---|---|---|---|---|
| P35221 | CTNNA1 | 2009001 | 3267071 | Catenin alpha-1 |
| P35222 | CTNNB1 | 2229672 | 1131896 | Catenin beta-1 |
| P35232 | PHB1 | 4029862 | 38534008 | Prohibitin 1 |
| P35237 | SERPINB6 | 2567716 | 4275417 | Serpin B6 |
| P35244 | RPA3 | 10311399 | 1867429 | Replication protein A 14 kDa subunit |
| P35249 | RFC4 | 18666666 | 25288652 | Replication factor C subunit 4 |
| P35250 | RFC2 | 1993235 | 15813881 | Replication factor C subunit 2 |
| P35251 | RFC1 | 5820822 | 2109673 | Replication factor C subunit 1 |
| P35268 | RPL22 | 187126944 | 38591120 | Large ribosomal subunit protein eL22 |
| P35527 | KRT9 | 8510876700 | 9368805400 | Keratin, type I cytoskeletal 9 |
| P35579 | MYH9 | 23767886 | 132214336 | Myosin-9 |
| P35580 | MYH10 | 5031265 | 0 | Myosin-10 |
| P35606 | COPB2 | 1759963 | 635225 | Coatomer subunit beta' |
| P35613 | BSG | 0 | 1157154 | Basigin |
| P35625 | TIMP3 | 31000608 | 4114466 | Metalloproteinase inhibitor 3 |
| P35637 | FUS | 178066800 | 237795808 | RNA-binding protein FUS |
| P35659 | DEK | 2977452 | 22359928 | Protein DEK |
| P35754 | GLRX | 1485946 | 1743686 | Glutaredoxin-1 |
| P35908 | KRT2 | 13814041600 | 15936821200 | Keratin, type II cytoskeletal 2 epidermal |
| P35998 | PSMC2 | 0 | 4064370 | 26S proteasome regulatory subunit 7 |
| P36542 | ATP5F1C | 5947700 | 13060167 | ATP synthase subunit gamma, mitochondrial |
| P36578 | RPL4 | 785986180 | 338632896 | Large ribosomal subunit protein uL4 |
| P36776 | LONP1 | 0 | 1120650 | Lon protease homolog, mitochondrial |
| P36873 | PPP1CC | 19666220 | 21125744 | Serine/threonine-protein phosphatase PP1-gamma catalytic subunit |
| P36957 | DLST | 0 | 19528052 | Dihydrolipoyllysine-residue succinyltransferase component of 2-oxoglutarate dehydrogenase complex, mitochondrial |
| P37108 | SRP14 | 1911850 | 10210561 | Signal recognition particle 14 kDa protein |
| P37802 | TAGLN2 | 0 | 53008648 | Transgelin-2 |
| P37837 | TALDO1 | 0 | 2374269 | Transaldolase |
| P38159 | RBMX | 53897456 | 468228064 | RNA-binding motif protein, X chromosome |

| | | | | |
|---|---|---|---|---|
| P38606 | ATP6V1A | 2216101 | 0 | V-type proton ATPase catalytic subunit A |
| P38646 | HSPA9 | 106247816 | 168210016 | Stress-70 protein, mitochondrial |
| P38919 | EIF4A3 | 79176856 | 22146022 | Eukaryotic initiation factor 4A-III |
| P39019 | RPS19 | 154696592 | 17739518 | Small ribosomal subunit protein eS19 |
| P39023 | RPL3 | 547845570 | 223413952 | Large ribosomal subunit protein uL3 |
| P39656 | DDOST | 0 | 731808 | Dolichyl-diphosphooligosaccharide--protein glycosyltransferase 48 kDa subunit |
| P39687 | ANP32A | 785032 | 1196943 | Acidic leucine-rich nuclear phosphoprotein 32 family member A |
| P39748 | FEN1 | 1100169 | 9193588 | Flap endonuclease 1 |
| P40121 | CAPG | 0 | 9522636 | Macrophage-capping protein |
| P40227 | CCT6A | 62074668 | 9370192 | T-complex protein 1 subunit zeta |
| P40429 | RPL13A | 300079648 | 134954272 | Large ribosomal subunit protein uL13 |
| P40616 | ARL1 | 0 | 521948 | ADP-ribosylation factor-like protein 1 |
| P40925 | MDH1 | 2061845 | 0 | Malate dehydrogenase, cytoplasmic |
| P40926 | MDH2 | 14651205 | 9066546 | Malate dehydrogenase, mitochondrial |
| P40937 | RFC5 | 15443580 | 11651286 | Replication factor C subunit 5 |
| P40938 | RFC3 | 5343357 | 10180415 | Replication factor C subunit 3 |
| P40939 | HADHA | 6084389 | 12530385 | Trifunctional enzyme subunit alpha, mitochondrial |
| P41091 | EIF2S3 | 1603291 | 20441308 | Eukaryotic translation initiation factor 2 subunit 3 |
| P41212 | ETV6 | 16576764 | 5322828 | Transcription factor ETV6 |
| P41252 | IARS1 | 0 | 265969568 | Isoleucine--tRNA ligase, cytoplasmic |
| P42166 | TMPO | 107443080 | 34997872 | Lamina-associated polypeptide 2, isoform alpha |
| P42285 | MTREX | 72445880 | 25020730 | Exosome RNA helicase MTR4 |
| P42357 | HAL | 28041994 | 39705796 | Histidine ammonia-lyase |
| P42677 | RPS27 | 144695984 | 28209516 | Small ribosomal subunit protein eS27 |

| | | | | |
|---|---|---|---|---|
| P42704 | LRPPRC | 2014473 | 16956826 | Leucine-rich PPR motif-containing protein, mitochondrial |
| P42766 | RPL35 | 119887096 | 70165672 | Large ribosomal subunit protein uL29 |
| P42785 | PRCP | 0 | 645173 | Lysosomal Pro-X carboxypeptidase |
| P43243 | MATR3 | 56672144 | 180034784 | Matrin-3 |
| P43487 | RANBP1 | 4681238 | 4960313 | Ran-specific GTPase-activating protein |
| P45880 | VDAC2 | 4429684 | 9552122 | Voltage-dependent anion-selective channel protein 2 |
| P46013 | MKI67 | 9680665 | 7436440 | Proliferation marker protein Ki-67 |
| P46087 | NOP2 | 0 | 52302180 | Probable 28S rRNA (cytosine(4447)-C(5))-methyltransferase |
| P46776 | RPL27A | 227271200 | 98607376 | Large ribosomal subunit protein uL15 |
| P46777 | RPL5 | 249782128 | 119019936 | Large ribosomal subunit protein uL18 |
| P46778 | RPL21 | 227215968 | 118794912 | Large ribosomal subunit protein eL21 |
| P46779 | RPL28 | 140586672 | 71906584 | Large ribosomal subunit protein eL28 |
| P46781 | RPS9 | 4090932480 | 9352874000 | Small ribosomal subunit protein uS4 |
| P46782 | RPS5 | 108367656 | 20249590 | Small ribosomal subunit protein uS7 |
| P46783 | RPS10 | 96317944 | 9221082 | Small ribosomal subunit protein eS10 |
| P46940 | IQGAP1 | 1189310 | 10792304 | Ras GTPase-activating-like protein IQGAP1 |
| P47756 | CAPZB | 7103230 | 7962403 | F-actin-capping protein subunit beta |
| P47897 | QARS1 | 398691 | 1897363 | Glutamine--tRNA ligase |
| P47914 | RPL29 | 102894000 | 52265892 | Large ribosomal subunit protein eL29 |
| P47929 | LGALS7B | 78450424 | 43460904 | Galectin-7 |
| P48047 | ATP5PO | 0 | 3688786 | ATP synthase subunit O, mitochondrial |
| P48163 | ME1 | 0 | 1500847 | NADP-dependent malic enzyme |
| P48454 | PPP3CC | 2466509 | 853783 | Serine/threonine-protein phosphatase 2B catalytic subunit gamma isoform |

| | | | | |
|---|---|---|---|---|
| P48594 | SERPINB4 | 2484340 | 0 | Serpin B4 |
| P48634 | PRRC2A | 39761208 | 5362321 | Protein PRRC2A |
| P48637 | GSS | 0 | 612806 | Glutathione synthetase |
| P48643 | CCT5 | 0 | 6344112 | T-complex protein 1 subunit epsilon |
| P48651 | PTDSS1 | 2301429 | 0 | Phosphatidylserine synthase 1 |
| P48729 | CSNK1A1 | 9390154 | 0 | Casein kinase I isoform alpha |
| P48740 | MASP1 | 19276292 | 22123096 | Mannan-binding lectin serine protease 1 |
| P49189 | ALDH9A1 | 2716672 | 5609708 | 4-trimethylaminobutyraldehyde dehydrogenase |
| P49207 | RPL34 | 252671008 | 71977992 | Large ribosomal subunit protein eL34 |
| P49327 | FASN | 14307282 | 189543136 | Fatty acid synthase |
| P49368 | CCT3 | 6148841 | 12578932 | T-complex protein 1 subunit gamma |
| P49406 | MRPL19 | 0 | 1592820 | Large ribosomal subunit protein bL19m |
| P49411 | TUFM | 24701436 | 54486968 | Elongation factor Tu, mitochondrial |
| P49458 | SRP9 | 0 | 3161921 | Signal recognition particle 9 kDa protein |
| P49588 | AARS1 | 2239118 | 23951086 | Alanine--tRNA ligase, cytoplasmic |
| P49643 | PRIM2 | 0 | 4625425 | DNA primase large subunit |
| P49720 | PSMB3 | 4555915 | 4332266 | Proteasome subunit beta type-3 |
| P49721 | PSMB2 | 61793404 | 3971137 | Proteasome subunit beta type-2 |
| P49736 | MCM2 | 0 | 1791240 | DNA replication licensing factor MCM2 |
| P49750 | YLPM1 | 148833200 | 173706032 | YLP motif-containing protein 1 |
| P49755 | TMED10 | 0 | 426015 | Transmembrane emp24 domain-containing protein 10 |
| P49756 | RBM25 | 3557880 | 7481052 | RNA-binding protein 25 |
| P49790 | NUP153 | 0 | 1734210 | Nuclear pore complex protein Nup153 |
| P49792 | RANBP2 | 0 | 14115852 | E3 SUMO-protein ligase RanBP2 |
| P49848 | TAF6 | 0 | 1491175 | Transcription initiation factor TFIID subunit 6 |
| P49916 | LIG3 | 0 | 4264143 | DNA ligase 3 |

| | | | | |
|---|---|---|---|---|
| P49959 | MRE11 | 0 | 2652369 | Double-strand break repair protein MRE11 |
| P50395 | GDI2 | 2642846 | 5084814 | Rab GDP dissociation inhibitor beta |
| P50402 | EMD | 25303372 | 105407768 | Emerin |
| P50416 | CPT1A | 0 | 414817 | Carnitine O-palmitoyltransferase 1, liver isoform |
| P50452 | SERPINB8 | 0 | 2806147 | Serpin B8 |
| P50454 | SERPINH1 | 0 | 10712302 | Serpin H1 |
| P50502 | ST13 | 2093907 | 3706826 | Hsc70-interacting protein |
| P50750 | CDK9 | 4355403 | 3950832 | Cyclin-dependent kinase 9 |
| P50914 | RPL14 | 219202576 | 112808536 | Large ribosomal subunit protein eL14 |
| P50990 | CCT8 | 9638904 | 17605446 | T-complex protein 1 subunit theta |
| P50991 | CCT4 | 2504430 | 12113067 | T-complex protein 1 subunit delta |
| P51114 | FXR1 | 44343728 | 37420572 | RNA-binding protein FXR1 |
| P51116 | FXR2 | 20106562 | 1624106 | RNA-binding protein FXR2 |
| P51149 | RAB7A | 0 | 1708022 | Ras-related protein Rab-7a |
| P51398 | DAP3 | 0 | 3681023 | Small ribosomal subunit protein mS29 |
| P51532 | SMARCA4 | 0 | 6216879 | Transcription activator BRG1 |
| P51571 | SSR4 | 0 | 806314 | Translocon-associated protein subunit delta |
| P51659 | HSD17B4 | 6737624 | 0 | Peroxisomal multifunctional enzyme type 2 |
| P51991 | HNRNPA3 | 7716708 | 49652992 | Heterogeneous nuclear ribonucleoprotein A3 |
| P52272 | HNRNPM | 57092304 | 568397890 | Heterogeneous nuclear ribonucleoprotein M |
| P52292 | KPNA2 | 1443980 | 10197692 | Importin subunit alpha-1 |
| P52294 | KPNA1 | 0 | 3770553 | Importin subunit alpha-5 |
| P52434 | POLR2H | 1802687 | 2522267 | DNA-directed RNA polymerases I, II, and III subunit RPABC3 |
| P52597 | HNRNPF | 283680864 | 164617536 | Heterogeneous nuclear ribonucleoprotein F |
| P52701 | MSH6 | 1577372 | 5269992 | DNA mismatch repair protein Msh6 |
| P52756 | RBM5 | 1203898 | 29406598 | RNA-binding protein 5 |
| P52907 | CAPZA1 | 6665146 | 8027855 | F-actin-capping protein subunit alpha-1 |

| | | | | |
|---|---|---|---|---|
| P52948 | NUP98 | 0 | 19222104 | Nuclear pore complex protein Nup98-Nup96 |
| P53007 | SLC25A1 | 20806932 | 42571788 | Tricarboxylate transport protein, mitochondrial |
| P53396 | ACLY | 8114479 | 13230474 | ATP-citrate synthase |
| P53621 | COPA | 2500494 | 15123453 | Coatomer subunit alpha |
| P53803 | POLR2K | 3891761 | 2823006 | DNA-directed RNA polymerases I, II, and III subunit RPABC4 |
| P53992 | SEC24C | 0 | 4813095 | Protein transport protein Sec24C |
| P53999 | SUB1 | 78444672 | 202853840 | Activated RNA polymerase II transcriptional coactivator p15 |
| P54105 | CLNS1A | 2704900 | 3846616 | Methylosome subunit pICln |
| P54132 | BLM | 4508074 | 0 | RecQ-like DNA helicase BLM |
| P54136 | RARS1 | 4676947 | 13295517 | Arginine--tRNA ligase, cytoplasmic |
| P54259 | ATN1 | 5616952 | 8941452 | Atrophin-1 |
| P54577 | YARS1 | 1548071 | 15836250 | Tyrosine--tRNA ligase, cytoplasmic |
| P54886 | ALDH18A1 | 1869242 | 6815420 | Delta-1-pyrroline-5-carboxylate synthase |
| P55072 | VCP | 20813230 | 41457060 | Transitional endoplasmic reticulum ATPase |
| P55081 | MFAP1 | 12764260 | 40594488 | Microfibrillar-associated protein 1 |
| P55083 | MFAP4 | 28259540 | 35211940 | Microfibril-associated glycoprotein 4 |
| P55084 | HADHB | 4457423 | 10654230 | Trifunctional enzyme subunit beta, mitochondrial |
| P55199 | ELL | 0 | 2115458 | RNA polymerase II elongation factor ELL |
| P55209 | NAP1L1 | 10795278 | 4910842 | Nucleosome assembly protein 1-like 1 |
| P55265 | ADAR | 9713586 | 66657596 | Double-stranded RNA-specific adenosine deaminase |
| P55735 | SEC13 | 77214200 | 35330592 | Protein SEC13 homolog |
| P55786 | NPEPPS | 1700557 | 0 | Puromycin-sensitive aminopeptidase |
| P55795 | HNRNPH2 | 12375030 | 40122356 | Heterogeneous nuclear ribonucleoprotein H2 |
| P55809 | OXCT1 | 0 | 9549957 | Succinyl-CoA:3-ketoacid coenzyme A transferase 1, mitochondrial |

| | | | | |
|---|---|---|---|---|
| P55854 | SUMO3 | 3402229 | 11091626 | Small ubiquitin-related modifier 3 |
| P55884 | EIF3B | 39588368 | 19664246 | Eukaryotic translation initiation factor 3 subunit B |
| P56134 | ATP5MF | 869991 | 0 | ATP synthase subunit f, mitochondrial |
| P56182 | RRP1 | 3458551 | 3411819 | Ribosomal RNA processing protein 1 homolog A |
| P56192 | MARS1 | 1958444 | 8763663 | Methionine--tRNA ligase, cytoplasmic |
| P56537 | EIF6 | 7547216 | 5431115 | Eukaryotic translation initiation factor 6 |
| P56545 | CTBP2 | 0 | 8199781 | C-terminal-binding protein 2 |
| P56556 | NDUFA6 | 2735720 | 0 | NADH dehydrogenase [ubiquinone] 1 alpha subcomplex subunit 6 |
| P57081 | WDR4 | 0 | 1969265 | tRNA (guanine-N(7)-)-methyltransferase non-catalytic subunit WDR4 |
| P57088 | TMEM33 | 794998 | 1904385 | Transmembrane protein 33 |
| P57678 | GEMIN4 | 5847602 | 11031558 | Gem-associated protein 4 |
| P58107 | EPPK1 | 0 | 14941144 | Epiplakin |
| P59998 | ARPC4 | 8764458 | 2262804 | Actin-related protein 2/3 complex subunit 4 |
| P60174 | TPI1 | 10422832 | 11221120 | Triosephosphate isomerase |
| P60228 | EIF3E | 54070188 | 15145826 | Eukaryotic translation initiation factor 3 subunit E |
| P60468 | SEC61B | 2038177 | 0 | Protein transport protein Sec61 subunit beta |
| P60660 | MYL6 | 15158907 | 41240108 | Myosin light polypeptide 6 |
| P60709 | ACTB | 2162710530 | 2535534340 | Actin, cytoplasmic 1 |
| P60842 | EIF4A1 | 20173168 | 24142536 | Eukaryotic initiation factor 4A-I |
| P60866 | RPS20 | 236204224 | 39448888 | Small ribosomal subunit protein uS10 |
| P60900 | PSMA6 | 7374195 | 8740945 | Proteasome subunit alpha type-6 |
| P60903 | S100A10 | 0 | 2102665 | Protein S100-A10 |
| P60981 | DSTN | 0 | 28786210 | Destrin |
| P61019 | RAB2A | 0 | 481806 | Ras-related protein Rab-2A |
| P61020 | RAB5B | 0 | 2658727 | Ras-related protein Rab-5B |
| P61077 | UBE2D3 | 0 | 1677092 | Ubiquitin-conjugating enzyme E2 D3 |
| P61088 | UBE2N | 2742400 | 1147206 | Ubiquitin-conjugating enzyme E2 N |

| | | | | |
|---|---|---|---|---|
| P61106 | RAB14 | 4377505 | 2368793 | Ras-related protein Rab-14 |
| P61158 | ACTR3 | 2457658 | 4884862 | Actin-related protein 3 |
| P61160 | ACTR2 | 4410125 | 2314068 | Actin-related protein 2 |
| P61204 | ARF3 | 0 | 392981 | ADP-ribosylation factor 3 |
| P61224 | RAP1B | 0 | 5880901 | Ras-related protein Rap-1b |
| P61247 | RPS3A | 446746336 | 84437688 | Small ribosomal subunit protein eS1 |
| P61313 | RPL15 | 321828896 | 125880336 | Large ribosomal subunit protein eL15 |
| P61326 | MAGOH | 1062354 | 0 | Protein mago nashi homolog |
| P61353 | RPL27 | 283620352 | 100601896 | Large ribosomal subunit protein eL27 |
| P61513 | RPL37A | 43052540 | 25566306 | Large ribosomal subunit protein eL43 |
| P61626 | LYZ | 20694848 | 20873776 | Lysozyme C |
| P61764 | STXBP1 | 0 | 467224 | Syntaxin-binding protein 1 |
| P61962 | DCAF7 | 2772344 | 2042582 | DDB1- and CUL4-associated factor 7 |
| P61964 | WDR5 | 16458561 | 97248752 | WD repeat-containing protein 5 |
| P61978 | HNRNPK | 887141630 | 2699629310 | Heterogeneous nuclear ribonucleoprotein K |
| P61981 | YWHAG | 5803795 | 4323910 | 14-3-3 protein gamma |
| P62081 | RPS7 | 150789200 | 68920560 | Small ribosomal subunit protein eS7 |
| P62136 | PPP1CA | 108896752 | 116443096 | Serine/threonine-protein phosphatase PP1-alpha catalytic subunit |
| P62140 | PPP1CB | 43377268 | 49725772 | Serine/threonine-protein phosphatase PP1-beta catalytic subunit |
| P62241 | RPS8 | 284732096 | 38458492 | Small ribosomal subunit protein eS8 |
| P62244 | RPS15A | 204900144 | 24094038 | Small ribosomal subunit protein uS8 |
| P62249 | RPS16 | 411519232 | 51785224 | Small ribosomal subunit protein uS9 |
| P62258 | YWHAE | 42465028 | 54909996 | 14-3-3 protein epsilon |
| P62263 | RPS14 | 215626160 | 40083664 | Small ribosomal subunit protein uS11 |
| P62266 | RPS23 | 131106040 | 32877074 | Small ribosomal subunit protein uS12 |
| P62269 | RPS18 | 361980992 | 43886580 | Small ribosomal subunit protein uS13 |

| | | | | |
|---|---|---|---|---|
| P62273 | RPS29 | 38019420 | 6886732 | Small ribosomal subunit protein uS14 |
| P62277 | RPS13 | 309843488 | 66171804 | Small ribosomal subunit protein uS15 |
| P62280 | RPS11 | 390260416 | 69875056 | Small ribosomal subunit protein uS17 |
| P62304 | SNRPE | 4712027 | 5522418 | Small nuclear ribonucleoprotein E |
| P62306 | SNRPF | 0 | 5461807 | Small nuclear ribonucleoprotein F |
| P62310 | LSM3 | 1146331 | 717181 | U6 snRNA-associated Sm-like protein LSm3 |
| P62314 | SNRPD1 | 3618660 | 6920159 | Small nuclear ribonucleoprotein Sm D1 |
| P62316 | SNRPD2 | 7595616 | 12146784 | Small nuclear ribonucleoprotein Sm D2 |
| P62318 | SNRPD3 | 6781763 | 14556164 | Small nuclear ribonucleoprotein Sm D3 |
| P62328 | TMSB4X | 24828498 | 18385264 | Thymosin beta-4 |
| P62424 | RPL7A | 477083840 | 264546880 | Large ribosomal subunit protein eL8 |
| P62491 | RAB11A | 0 | 413401 | Ras-related protein Rab-11A |
| P62701 | RPS4X | 598827070 | 101398464 | Small ribosomal subunit protein eS4, X isoform |
| P62714 | PPP2CB | 5215531 | 4037334 | Serine/threonine-protein phosphatase 2A catalytic subunit beta isoform |
| P62736 | ACTA2 | 39214196 | 55971932 | Actin, aortic smooth muscle |
| P62750 | RPL23A | 331627360 | 118373152 | Large ribosomal subunit protein uL23 |
| P62753 | RPS6 | 238565744 | 68665648 | Small ribosomal subunit protein eS6 |
| P62805 | H4C16 | 204942464 | 756328510 | Histone H4 |
| P62820 | RAB1A | 2501819 | 8428137 | Ras-related protein Rab-1A |
| P62826 | RAN | 8073032 | 22012188 | GTP-binding nuclear protein Ran |
| P62829 | RPL23 | 249245232 | 138236176 | Large ribosomal subunit protein uL14 |
| P62841 | RPS15 | 50534904 | 7160120 | Small ribosomal subunit protein uS19 |
| P62847 | RPS24 | 85051824 | 24233202 | Small ribosomal subunit protein eS24 |
| P62851 | RPS25 | 317787680 | 44839820 | Small ribosomal subunit protein eS25 |

| | | | | |
|---|---|---|---|---|
| P62854 | RPS26 | 145707616 | 31298132 | Small ribosomal subunit protein eS26 |
| P62857 | RPS28 | 82093000 | 24806932 | Small ribosomal subunit protein eS28 |
| P62861 | FAU | 73628936 | 14198622 | Ubiquitin-like FUBI-ribosomal protein eS30 fusion protein |
| P62879 | GNB2 | 0 | 1780043 | Guanine nucleotide-binding protein G(I)/G(S)/G(T) subunit beta-2 |
| P62888 | RPL30 | 1140190210 | 283475616 | Large ribosomal subunit protein eL30 |
| P62891 | RPL39 | 2875045 | 0 | Large ribosomal subunit protein eL39 |
| P62899 | RPL31 | 283916064 | 77381496 | Large ribosomal subunit protein eL31 |
| P62906 | RPL10A | 348171072 | 212856720 | Large ribosomal subunit protein uL1 |
| P62910 | RPL32 | 97891112 | 52114672 | Large ribosomal subunit protein eL32 |
| P62913 | RPL11 | 234628544 | 179166992 | Large ribosomal subunit protein uL5 |
| P62917 | RPL8 | 414365120 | 274336960 | Large ribosomal subunit protein uL2 |
| P62937 | PPIA | 29258860 | 75136232 | Peptidyl-prolyl cis-trans isomerase A |
| P62979 | RPS27A | 222434976 | 592644480 | Ubiquitin-40S ribosomal protein S27a |
| P62995 | TRA2B | 16622562 | 121287976 | Transformer-2 protein homolog beta |
| P63010 | AP2B1 | 449537 | 0 | AP-2 complex subunit beta |
| P63104 | YWHAZ | 17475910 | 25974838 | 14-3-3 protein zeta/delta |
| P63167 | DYNLL1 | 5623234 | 39529944 | Dynein light chain 1, cytoplasmic |
| P63173 | RPL38 | 62033360 | 13213928 | Large ribosomal subunit protein eL38 |
| P63208 | SKP1 | 2550705 | 2382112 | S-phase kinase-associated protein 1 |
| P63220 | RPS21 | 94044944 | 10554791 | Small ribosomal subunit protein eS21 |
| P63241 | EIF5A | 11604817 | 51780864 | Eukaryotic translation initiation factor 5A-1 |
| P63244 | RACK1 | 299307136 | 82697232 | Small ribosomal subunit protein RACK1 |
| P63261 | ACTG1 | 0 | 2727165 | Actin, cytoplasmic 2 |

| | | | | |
|---|---|---|---|---|
| P63313 | TMSB10 | 2658638 | 0 | Thymosin beta-10 |
| P67809 | YBX1 | 183822480 | 5002410 | Y-box-binding protein 1 |
| P67870 | CSNK2B | 6340027 | 4272517 | Casein kinase II subunit beta |
| P68104 | EEF1A1 | 192352672 | 246734784 | Elongation factor 1-alpha 1 |
| P68366 | TUBA4A | 39000856 | 23043790 | Tubulin alpha-4A chain |
| P68371 | TUBB4B | 261740960 | 97040816 | Tubulin beta-4B chain |
| P68400 | CSNK2A1 | 3375821 | 9339973 | Casein kinase II subunit alpha |
| P68431 | H3C12 | 84947304 | 400018976 | Histone H3.1 |
| P68871 | HBB | 158818736 | 40385040 | Hemoglobin subunit beta |
| P69905 | HBA2 | 244903952 | 133206888 | Hemoglobin subunit alpha |
| P78316 | NOP14 | 1979777 | 8090228 | Nucleolar protein 14 |
| P78330 | PSPH | 0 | 733880 | Phosphoserine phosphatase |
| P78332 | RBM6 | 54049372 | 48694124 | RNA-binding protein 6 |
| P78337 | PITX1 | 1755185 | 0 | Pituitary homeobox 1 |
| P78344 | EIF4G2 | 2511507 | 10858164 | Eukaryotic translation initiation factor 4 gamma 2 |
| P78347 | GTF2I | 7248503 | 26025480 | General transcription factor II-I |
| P78358 | CTAG1B | 1883133 | 6599868 | Cancer/testis antigen 1 |
| P78362 | SRPK2 | 4037993 | 2605129 | SRSF protein kinase 2 |
| P78527 | PRKDC | 145802224 | 5343004200 | DNA-dependent protein kinase catalytic subunit |
| P81605 | DCD | 136915776 | 167546288 | Dermcidin |
| P82650 | MRPS22 | 1579732 | 0 | Small ribosomal subunit protein mS22 |
| P82675 | MRPS5 | 4752665 | 10729900 | Small ribosomal subunit protein uS5m |
| P82914 | MRPS15 | 0 | 4427774 | Small ribosomal subunit protein uS15m |
| P82930 | MRPS34 | 5752683 | 4559108 | Small ribosomal subunit protein mS34 |
| P83111 | LACTB | 8291090 | 16302874 | Serine beta-lactamase-like protein LACTB, mitochondrial |
| P83731 | RPL24 | 128743312 | 70087016 | Large ribosomal subunit protein eL24 |
| P84022 | SMAD3 | 0 | 2213019 | Mothers against decapentaplegic homolog 3 |
| P84090 | ERH | 9131878 | 23857712 | Enhancer of rudimentary homolog |
| P84098 | RPL19 | 192511904 | 76714928 | Large ribosomal subunit protein eL19 |
| P84103 | SRSF3 | 79444744 | 302981568 | Serine/arginine-rich splicing factor 3 |

| | | | | |
|---|---|---|---|---|
| P98170 | XIAP | 6284717 | 0 | E3 ubiquitin-protein ligase XIAP |
| P98175 | RBM10 | 22684178 | 1240583170 | RNA-binding protein 10 |
| P99999 | CYCS | 1827163 | 2032036 | Cytochrome c |
| Q00059 | TFAM | 11363638 | 23253626 | Transcription factor A, mitochondrial |
| Q00325 | SLC25A3 | 63607612 | 47558960 | Solute carrier family 25 member 3 |
| Q00577 | PURA | 15971068 | 1926670 | Transcriptional activator protein Pur-alpha |
| Q00587 | CDC42EP1 | 7767937 | 6276948 | Cdc42 effector protein 1 |
| Q00610 | CLTC | 542847680 | 92898496 | Clathrin heavy chain 1 |
| Q00653 | NFKB2 | 337115 | 20372526 | Nuclear factor NF-kappa-B p100 subunit |
| Q00839 | HNRNPU | 117331856 | 853199360 | Heterogeneous nuclear ribonucleoprotein U |
| Q01081 | U2AF1 | 5460300 | 10520710 | Splicing factor U2AF 35 kDa subunit |
| Q01082 | SPTBN1 | 2025161470 | 0 | Spectrin beta chain, non-erythrocytic 1 |
| Q01105 | SET | 23725032 | 14131393 | Protein SET |
| Q01130 | SRSF2 | 43772652 | 195450336 | Serine/arginine-rich splicing factor 2 |
| Q01469 | FABP5 | 22239888 | 21323764 | Fatty acid-binding protein 5 |
| Q01650 | SLC7A5 | 3891038 | 8472702 | Large neutral amino acids transporter small subunit 1 |
| Q01658 | DR1 | 0 | 513590 | Protein Dr1 |
| Q01813 | PFKP | 5042519 | 4939539 | ATP-dependent 6-phosphofructokinase, platelet type |
| Q01844 | EWSR1 | 186379840 | 120705408 | RNA-binding protein EWS |
| Q02218 | OGDH | 0 | 808874 | 2-oxoglutarate dehydrogenase complex component E1 |
| Q02241 | KIF23 | 0 | 2302113 | Kinesin-like protein KIF23 |
| Q02413 | DSG1 | 152322704 | 161648784 | Desmoglein-1 |
| Q02543 | RPL18A | 71187312 | 71456080 | Large ribosomal subunit protein eL20 |
| Q02809 | PLOD1 | 0 | 542980 | Procollagen-lysine,2-oxoglutarate 5-dioxygenase 1 |
| Q02878 | RPL6 | 654324800 | 445429664 | Large ribosomal subunit protein eL6 |
| Q02880 | TOP2B | 16183182 | 38439176 | DNA topoisomerase 2-beta |
| Q02978 | SLC25A11 | 16122230 | 7780369 | Mitochondrial 2-oxoglutarate/malate carrier protein |

| | | | | |
|---|---|---|---|---|
| Q03135 | CAV1 | 20689508 | 60753736 | Caveolin-1 |
| Q03468 | ERCC6 | 11737150 | 6333552 | DNA excision repair protein ERCC-6 |
| Q04206 | RELA | 0 | 1280127 | Transcription factor p65 |
| Q04637 | EIF4G1 | 0 | 4914831 | Eukaryotic translation initiation factor 4 gamma 1 |
| Q04695 | KRT17 | 294412032 | 52483532 | Keratin, type I cytoskeletal 17 |
| Q04760 | GLO1 | 4362337 | 2043667 | Lactoylglutathione lyase |
| Q04837 | SSBP1 | 369597248 | 217729536 | Single-stranded DNA-binding protein, mitochondrial |
| Q05048 | CSTF1 | 0 | 1732543 | Cleavage stimulation factor subunit 1 |
| Q05209 | PTPN12 | 0 | 639713 | Tyrosine-protein phosphatase non-receptor type 12 |
| Q05519 | SRSF11 | 8408346 | 23250948 | Serine/arginine-rich splicing factor 11 |
| Q05655 | PRKCD | 0 | 550620 | Protein kinase C delta type |
| Q06210 | GFPT1 | 0 | 1474798 | Glutamine--fructose-6-phosphate aminotransferase [isomerizing] 1 |
| Q06265 | EXOSC9 | 2279991 | 1492364 | Exosome complex component RRP45 |
| Q06787 | FMR1 | 17754170 | 5846199 | Fragile X messenger ribonucleoprotein 1 |
| Q06830 | PRDX1 | 117286280 | 340896416 | Peroxiredoxin-1 |
| Q07020 | RPL18 | 522276256 | 256268208 | Large ribosomal subunit protein eL18 |
| Q07065 | CKAP4 | 2458662 | 2836472 | Cytoskeleton-associated protein 4 |
| Q07157 | TJP1 | 141672560 | 156061792 | Tight junction protein ZO-1 |
| Q07666 | KHDRBS1 | 8240464400 | 6175449600 | KH domain-containing, RNA-binding, signal transduction-associated protein 1 |
| Q07864 | POLE | 0 | 216750 | DNA polymerase epsilon catalytic subunit A |
| Q07955 | SRSF1 | 28292600 | 117639272 | Serine/arginine-rich splicing factor 1 |
| Q07960 | ARHGAP1 | 6461739 | 0 | Rho GTPase-activating protein 1 |
| Q08188 | TGM3 | 30042650 | 42576844 | Protein-glutamine gamma-glutamyltransferase E |
| Q08211 | DHX9 | 10985966 | 129398952 | ATP-dependent RNA helicase A |
| Q08257 | CRYZ | 0 | 2741973 | Quinone oxidoreductase |

| | | | | |
|---|---|---|---|---|
| Q08378 | GOLGA3 | 0 | 48206832 | Golgin subfamily A member 3 |
| Q08379 | GOLGA2 | 0 | 17632124 | Golgin subfamily A member 2 |
| Q08554 | DSC1 | 58049212 | 94466104 | Desmocollin-1 |
| Q08945 | SSRP1 | 6802301 | 7448008 | FACT complex subunit SSRP1 |
| Q08AG7 | MZT1 | 30783732 | 0 | Mitotic-spindle organizing protein 1 |
| Q08J23 | NSUN2 | 22369780 | 146927344 | RNA cytosine C(5)-methyltransferase NSUN2 |
| Q09028 | RBBP4 | 0 | 11656000 | Histone-binding protein RBBP4 |
| Q09161 | NCBP1 | 1846925 | 1058322 | Nuclear cap-binding protein subunit 1 |
| Q09472 | EP300 | 0 | 7388785 | Histone acetyltransferase p300 |
| Q09666 | AHNAK | 18152752 | 26153456 | Neuroblast differentiation-associated protein AHNAK |
| Q10570 | CPSF1 | 427992768 | 502768992 | Cleavage and polyadenylation specificity factor subunit 1 |
| Q12769 | NUP160 | 3040263 | 0 | Nuclear pore complex protein Nup160 |
| Q12789 | GTF3C1 | 2470260 | 6099050 | General transcription factor 3C polypeptide 1 |
| Q12840 | KIF5A | 0 | 892051 | Kinesin heavy chain isoform 5A |
| Q12849 | GRSF1 | 0 | 663545 | G-rich sequence factor 1 |
| Q12857 | NFIA | 0 | 7475438 | Nuclear factor 1 A-type |
| Q12872 | SFSWAP | 1613006 | 620393 | Splicing factor, suppressor of white-apricot homolog |
| Q12873 | CHD3 | 4648268 | 7312617 | Chromodomain-helicase-DNA-binding protein 3 |
| Q12874 | SF3A3 | 1913500 | 2549439 | Splicing factor 3A subunit 3 |
| Q12904 | AIMP1 | 0 | 2917169 | Aminoacyl tRNA synthase complex-interacting multifunctional protein 1 |
| Q12905 | ILF2 | 21285622 | 53817944 | Interleukin enhancer-binding factor 2 |
| Q12906 | ILF3 | 46922980 | 143342112 | Interleukin enhancer-binding factor 3 |
| Q12931 | TRAP1 | 4794631 | 10334376 | Heat shock protein 75 kDa, mitochondrial |
| Q12933 | TRAF2 | 0 | 2068274 | TNF receptor-associated factor 2 |

| | | | | |
|---|---|---|---|---|
| Q12965 | MYO1E | 51956848 | 54034196 | Unconventional myosin-Ie |
| Q12968 | NFATC3 | 13401640 | 0 | Nuclear factor of activated T-cells, cytoplasmic 3 |
| Q12996 | CSTF3 | 9294790 | 3214091 | Cleavage stimulation factor subunit 3 |
| Q13045 | FLII | 833680 | 0 | Protein flightless-1 homolog |
| Q13084 | MRPL28 | 1547428 | 841915 | Large ribosomal subunit protein bL28m |
| Q13123 | IK | 0 | 3692714 | Protein Red |
| Q13136 | PPFIA1 | 627717380 | 161048544 | Liprin-alpha-1 |
| Q13148 | TARDBP | 0 | 20401492 | TAR DNA-binding protein 43 |
| Q13151 | HNRNPA0 | 3799173 | 69672376 | Heterogeneous nuclear ribonucleoprotein A0 |
| Q13155 | AIMP2 | 0 | 1412463 | Aminoacyl tRNA synthase complex-interacting multifunctional protein 2 |
| Q13162 | PRDX4 | 12081972 | 8452521 | Peroxiredoxin-4 |
| Q13185 | CBX3 | 1944948 | 7098907 | Chromobox protein homolog 3 |
| Q13188 | STK3 | 12464804 | 0 | Serine/threonine-protein kinase 3 |
| Q13200 | PSMD2 | 0 | 4246501 | 26S proteasome non-ATPase regulatory subunit 2 |
| Q13214 | SEMA3B | 0 | 1373248 | Semaphorin-3B |
| Q13242 | SRSF9 | 8119933 | 94563840 | Serine/arginine-rich splicing factor 9 |
| Q13243 | SRSF5 | 10288927 | 61185116 | Serine/arginine-rich splicing factor 5 |
| Q13247 | SRSF6 | 2504724 | 19432000 | Serine/arginine-rich splicing factor 6 |
| Q13263 | TRIM28 | 0 | 37089420 | Transcription intermediary factor 1-beta |
| Q13283 | G3BP1 | 86735496 | 46924976 | Ras GTPase-activating protein-binding protein 1 |
| Q13310 | PABPC4 | 161036208 | 9242048500 | Polyadenylate-binding protein 4 |
| Q13347 | EIF3I | 28748144 | 7644603 | Eukaryotic translation initiation factor 3 subunit I |
| Q13362 | PPP2R5C | 6027777 | 1459918 | Serine/threonine-protein phosphatase 2A 56 kDa regulatory subunit gamma isoform |
| Q13404 | UBE2V1 | 1923689860 | 1623120510 | Ubiquitin-conjugating enzyme E2 variant 1 |

| | | | | |
|---|---|---|---|---|
| Q13428 | TCOF1 | 2306324 | 23292532 | Treacle protein |
| Q13435 | SF3B2 | 19818238 | 123610392 | Splicing factor 3B subunit 2 |
| Q13472 | TOP3A | 6831890 | 3079308 | DNA topoisomerase 3-alpha |
| Q13501 | SQSTM1 | 5663166 | 16569032 | Sequestosome-1 |
| Q13503 | MED21 | 6144119 | 9411316 | Mediator of RNA polymerase II transcription subunit 21 |
| Q13509 | TUBB3 | 5505311 | 0 | Tubulin beta-3 chain |
| Q13510 | ASAH1 | 777039 | 0 | Acid ceramidase |
| Q13523 | PRPF4B | 13770424 | 10806262 | Serine/threonine-protein kinase PRP4 homolog |
| Q13547 | HDAC1 | 5166498 | 9716913 | Histone deacetylase 1 |
| Q13554 | CAMK2B | 2981208 | 5804264 | Calcium/calmodulin-dependent protein kinase type II subunit beta |
| Q13573 | SNW1 | 60708784 | 54796564 | SNW domain-containing protein 1 |
| Q13595 | TRA2A | 2860730 | 41512740 | Transformer-2 protein homolog alpha |
| Q13601 | KRR1 | 0 | 1902952 | KRR1 small subunit processome component homolog |
| Q13813 | SPTAN1 | 0 | 6043453 | Spectrin alpha chain, non-erythrocytic 1 |
| Q13835 | PKP1 | 17269992 | 83824816 | Plakophilin-1 |
| Q13867 | BLMH | 17632386 | 16306522 | Bleomycin hydrolase |
| Q13868 | EXOSC2 | 2433296 | 1441835 | Exosome complex component RRP4 |
| Q13884 | SNTB1 | 0 | 1441375 | Beta-1-syntrophin |
| Q14011 | CIRBP | 0 | 3068326 | Cold-inducible RNA-binding protein |
| Q14103 | HNRNPD | 8365623 | 21801536 | Heterogeneous nuclear ribonucleoprotein D0 |
| Q14137 | BOP1 | 0 | 3940668 | Ribosome biogenesis protein BOP1 |
| Q14145 | KEAP1 | 2041094 | 0 | Kelch-like ECH-associated protein 1 |
| Q14151 | SAFB2 | 90167376 | 365899904 | Scaffold attachment factor B2 |
| Q14152 | EIF3A | 83366264 | 34940680 | Eukaryotic translation initiation factor 3 subunit A |
| Q14155 | ARHGEF7 | 22707102 | 2997424 | Rho guanine nucleotide exchange factor 7 |
| Q14157 | UBAP2L | 127794352 | 129986432 | Ubiquitin-associated protein 2-like |
| Q14166 | TTLL12 | 0 | 6617322 | Tubulin--tyrosine ligase-like protein 12 |

| | | | | |
|---|---|---|---|---|
| Q14192 | FHL2 | 5801389 | 14349737 | Four and a half LIM domains protein 2 |
| Q14204 | DYNC1H1 | 5146803 | 10359990 | Cytoplasmic dynein 1 heavy chain 1 |
| Q14244 | MAP7 | 4987433 | 18762340 | Ensconsin |
| Q14247 | CTTN | 653873790 | 234018272 | Src substrate cortactin |
| Q14257 | RCN2 | 273895552 | 156996656 | Reticulocalbin-2 |
| Q14258 | TRIM25 | 2444994 | 21354652 | E3 ubiquitin/ISG15 ligase TRIM25 |
| Q14318 | FKBP8 | 0 | 1383410 | Peptidyl-prolyl cis-trans isomerase FKBP8 |
| Q14444 | CAPRIN1 | 84898920 | 38123328 | Caprin-1 |
| Q14493 | SLBP | 5450198 | 0 | Histone RNA hairpin-binding protein |
| Q14498 | RBM39 | 68233184 | 112769672 | RNA-binding protein 39 |
| Q14517 | FAT1 | 4612416 | 0 | Protocadherin Fat 1 |
| Q14525 | KRT33B | 3872623 | 1994770 | Keratin, type I cuticular Ha3-II |
| Q14566 | MCM6 | 2714203 | 5111158 | DNA replication licensing factor MCM6 |
| Q14571 | ITPR2 | 0 | 18286144 | Inositol 1,4,5-trisphosphate receptor type 2 |
| Q14574 | DSC3 | 5282134 | 5303572 | Desmocollin-3 |
| Q14677 | CLINT1 | 4747349 | 23076328 | Clathrin interactor 1 |
| Q14684 | RRP1B | 24224158 | 5311524 | Ribosomal RNA processing protein 1 homolog B |
| Q14687 | GSE1 | 2168400 | 1948536 | Genetic suppressor element 1 |
| Q14694 | USP10 | 20636968 | 1450273 | Ubiquitin carboxyl-terminal hydrolase 10 |
| Q14697 | GANAB | 0 | 8049890 | Neutral alpha-glucosidase AB |
| Q14739 | LBR | 1483068 | 22451854 | Delta(14)-sterol reductase LBR |
| Q14764 | MVP | 10303480 | 24541524 | Major vault protein |
| Q14839 | CHD4 | 4779430 | 28367122 | Chromodomain-helicase-DNA-binding protein 4 |
| Q14847 | LASP1 | 2515171 | 5665994 | LIM and SH3 domain protein 1 |
| Q14956 | GPNMB | 2218588 | 0 | Transmembrane glycoprotein NMB |
| Q14966 | ZNF638 | 39103376 | 23080604 | Zinc finger protein 638 |
| Q14974 | KPNB1 | 0 | 15485256 | Importin subunit beta-1 |
| Q14978 | NOLC1 | 4776588 | 37659428 | Nucleolar and coiled-body phosphoprotein 1 |

| | | | | |
|---|---|---|---|---|
| Q14980 | NUMA1 | 170789136 | 235019856 | Nuclear mitotic apparatus protein 1 |
| Q14C86 | GAPVD1 | 0 | 495654 | GTPase-activating protein and VPS9 domain-containing protein 1 |
| Q14CN4 | KRT72 | 23793970 | 260759136 | Keratin, type II cytoskeletal 72 |
| Q15008 | PSMD6 | 0 | 365255 | 26S proteasome non-ATPase regulatory subunit 6 |
| Q15020 | SART3 | 0 | 6977348 | Squamous cell carcinoma antigen recognized by T-cells 3 |
| Q15025 | TNIP1 | 1603025 | 0 | TNFAIP3-interacting protein 1 |
| Q15029 | EFTUD2 | 69149776 | 19362598 | 116 kDa U5 small nuclear ribonucleoprotein component |
| Q15046 | KARS1 | 0 | 1402820 | Lysine--tRNA ligase |
| Q15056 | EIF4H | 42509208 | 28533290 | Eukaryotic translation initiation factor 4H |
| Q15084 | PDIA6 | 0 | 5562948 | Protein disulfide-isomerase A6 |
| Q15149 | PLEC | 54989360 | 655511940 | Plectin |
| Q15181 | PPA1 | 0 | 4652898 | Inorganic pyrophosphatase |
| Q15185 | PTGES3 | 0 | 10223270 | Prostaglandin E synthase 3 |
| Q15233 | NONO | 89123912 | 423946720 | Non-POU domain-containing octamer-binding protein |
| Q15291 | RBBP5 | 0 | 1071836 | Retinoblastoma-binding protein 5 |
| Q15293 | RCN1 | 43967768 | 166576144 | Reticulocalbin-1 |
| Q15311 | RALBP1 | 0 | 2152684 | RalA-binding protein 1 |
| Q15365 | PCBP1 | 28235906 | 196493600 | Poly(rC)-binding protein 1 |
| Q15366 | PCBP2 | 12091747 | 108349792 | Poly(rC)-binding protein 2 |
| Q15370 | ELOB | 2424945 | 2354510 | Elongin-B |
| Q15388 | TOMM20 | 0 | 6412383 | Mitochondrial import receptor subunit TOM20 homolog |
| Q15393 | SF3B3 | 30098414 | 336624512 | Splicing factor 3B subunit 3 |
| Q15424 | SAFB | 32214176 | 188027216 | Scaffold attachment factor B1 |
| Q15427 | SF3B4 | 6768420 | 18853192 | Splicing factor 3B subunit 4 |
| Q15438 | CYTH1 | 1042349 | 0 | Cytohesin-1 |
| Q15459 | SF3A1 | 0 | 16512202 | Splicing factor 3A subunit 1 |
| Q15517 | CDSN | 19116648 | 24259848 | Corneodesmosin |
| Q15528 | MED22 | 1892912 | 535857 | Mediator of RNA polymerase II transcription subunit 22 |
| Q15637 | SF1 | 30793856 | 10498894 | Splicing factor 1 |

| | | | | |
|---|---|---|---|---|
| Q15645 | TRIP13 | 782692 | 0 | Pachytene checkpoint protein 2 homolog |
| Q15646 | OASL | 17555364 | 1964918 | 2'-5'-oligoadenylate synthase-like protein |
| Q15648 | MED1 | 23153874 | 21334770 | Mediator of RNA polymerase II transcription subunit 1 |
| Q15650 | TRIP4 | 1034310 | 0 | Activating signal cointegrator 1 |
| Q15654 | TRIP6 | 9702507 | 2896763 | Thyroid receptor-interacting protein 6 |
| Q15691 | MAPRE1 | 3847674 | 1706110 | Microtubule-associated protein RP/EB family member 1 |
| Q15717 | ELAVL1 | 14565528 | 27448032 | ELAV-like protein 1 |
| Q15828 | CST6 | 1100773 | 1322519 | Cystatin-M |
| Q16531 | DDB1 | 45179748 | 53556272 | DNA damage-binding protein 1 |
| Q16540 | MRPL23 | 2795156 | 1640559 | Large ribosomal subunit protein uL23m |
| Q16543 | CDC37 | 0 | 3884045 | Hsp90 co-chaperone Cdc37 |
| Q16563 | SYPL1 | 0 | 1434424 | Synaptophysin-like protein 1 |
| Q16576 | RBBP7 | 12799540 | 45871332 | Histone-binding protein RBBP7 |
| Q16610 | ECM1 | 3085284 | 0 | Extracellular matrix protein 1 |
| Q16629 | SRSF7 | 18101042 | 155554512 | Serine/arginine-rich splicing factor 7 |
| Q16630 | CPSF6 | 339146624 | 635258300 | Cleavage and polyadenylation specificity factor subunit 6 |
| Q16637 | SMN2 | 0 | 1002196 | Survival motor neuron protein |
| Q16658 | FSCN1 | 2726068 | 15833098 | Fascin |
| Q16795 | NDUFA9 | 0 | 1108802 | NADH dehydrogenase [ubiquinone] 1 alpha subcomplex subunit 9, mitochondrial |
| Q16822 | PCK2 | 0 | 1785976 | Phosphoenolpyruvate carboxykinase [GTP], mitochondrial |
| Q16851 | UGP2 | 1185577 | 1999726 | UTP--glucose-1-phosphate uridylyltransferase |
| Q16891 | IMMT | 1525831 | 22458802 | MICOS complex subunit MIC60 |
| Q17RY0 | CPEB4 | 9146580 | 4108579 | Cytoplasmic polyadenylation element-binding protein 4 |

| | | | | |
|---|---|---|---|---|
| Q1KMD3 | HNRNPUL2 | 64434732 | 112292704 | Heterogeneous nuclear ribonucleoprotein U-like protein 2 |
| Q1MSJ5 | CSPP1 | 3800465 | 0 | Centrosome and spindle pole-associated protein 1 |
| Q2NL82 | TSR1 | 1988785 | 3204063 | Pre-rRNA-processing protein TSR1 homolog |
| Q2TAY7 | SMU1 | 0 | 6509912 | WD40 repeat-containing protein SMU1 |
| Q3KQU3 | MAP7D1 | 8344699 | 0 | MAP7 domain-containing protein 1 |
| Q3MHD2 | LSM12 | 26857124 | 8968568 | Protein LSM12 |
| Q4G0J3 | LARP7 | 0 | 939411 | La-related protein 7 |
| Q52LJ0 | FAM98B | 0 | 68107240 | Protein FAM98B |
| Q53EL6 | PDCD4 | 0 | 3295573 | Programmed cell death protein 4 |
| Q53EP0 | FNDC3B | 0 | 474012 | Fibronectin type III domain-containing protein 3B |
| Q53ET0 | CRTC2 | 34624072 | 5411285 | CREB-regulated transcription coactivator 2 |
| Q53GA4 | PHLDA2 | 0 | 1749492 | Pleckstrin homology-like domain family A member 2 |
| Q562F6 | SGO2 | 0 | 2284085 | Shugoshin 2 |
| Q5BKY9 | FAM133B | 5228499 | 5781728 | Protein FAM133B |
| Q5BKZ1 | ZNF326 | 87603912 | 331191072 | DBIRD complex subunit ZNF326 |
| Q5D862 | FLG2 | 24094886 | 31253646 | Filaggrin-2 |
| Q5JRA6 | MIA3 | 0 | 4333718 | Transport and Golgi organization protein 1 homolog |
| Q5JSZ5 | PRRC2B | 600909250 | 3012032 | Protein PRRC2B |
| Q5JTH9 | RRP12 | 13243860 | 6794175 | RRP12-like protein |
| Q5JVF3 | PCID2 | 11461235 | 6127120 | PCI domain-containing protein 2 |
| Q5JWF2 | GNAS | 2207628 | 5144359 | Guanine nucleotide-binding protein G(s) subunit alpha isoforms XLas |
| Q5QJE6 | DNTTIP2 | 5091448 | 0 | Deoxynucleotidyltransferase terminal-interacting protein 2 |
| Q5SW79 | CEP170 | 16783886 | 2081527 | Centrosomal protein of 170 kDa |
| Q5T280 | SPOUT1 | 25002318 | 10514561 | Putative methyltransferase C9orf114 |
| Q5T3I0 | GPATCH4 | 5443921 | 3201888 | G patch domain-containing protein 4 |

| | | | | |
|---|---|---|---|---|
| Q5T6F2 | UBAP2 | 6314321 | 2262338 | Ubiquitin-associated protein 2 |
| Q5T749 | KPRP | 28764742 | 56517452 | Keratinocyte proline-rich protein |
| Q5T750 | KPLCE | 44527032 | 61365484 | Protein KPLCE |
| Q5T8P6 | RBM26 | 0 | 5919871 | RNA-binding protein 26 |
| Q5T9A4 | ATAD3B | 11685296 | 0 | ATPase family AAA domain-containing protein 3B |
| Q5TC82 | RC3H1 | 4548145 | 0 | Roquin-1 |
| Q5TGY3 | AHDC1 | 7171596 | 0 | Transcription factor Gibbin |
| Q5UIP0 | RIF1 | 0 | 3997153 | Telomere-associated protein RIF1 |
| Q5VT25 | CDC42BPA | 109899720 | 0 | Serine/threonine-protein kinase MRCK alpha |
| Q5VUA4 | ZNF318 | 43815672 | 0 | Zinc finger protein 318 |
| Q5VW36 | FOCAD | 6148919 | 8792147 | Focadhesin |
| Q5VWQ0 | RSBN1 | 4562062 | 3193172 | Lysine-specific demethylase 9 |
| Q5VYK3 | ECPAS | 0 | 2283864 | Proteasome adapter and scaffold protein ECM29 |
| Q5XKE5 | KRT79 | 1566605 | 0 | Keratin, type II cytoskeletal 79 |
| Q63HN8 | RNF213 | 0 | 5719652 | E3 ubiquitin-protein ligase RNF213 |
| Q63ZY3 | KANK2 | 10109797 | 0 | KN motif and ankyrin repeat domain-containing protein 2 |
| Q66PJ3 | ARL6IP4 | 9792117 | 2989945 | ADP-ribosylation factor-like protein 6-interacting protein 4 |
| Q69YL0 | NCBP2AS2 | 0 | 322829 | Protein NCBP2AS2 |
| Q6DN12 | MCTP2 | 0 | 2196969 | Multiple C2 and transmembrane domain-containing protein 2 |
| Q6DN90 | IQSEC1 | 1921250 | 45498048 | IQ motif and SEC7 domain-containing protein 1 |
| Q6KB66 | KRT80 | 32366840 | 40185240 | Keratin, type II cytoskeletal 80 |
| Q6KC79 | NIPBL | 2633289 | 2785716 | Nipped-B-like protein |
| Q6NZI2 | CAVIN1 | 62978744 | 167592192 | Caveolae-associated protein 1 |
| Q6NZY4 | ZCCHC8 | 681076 | 706979 | Zinc finger CCHC domain-containing protein 8 |
| Q6P0Q8 | MAST2 | 954029 | 0 | Microtubule-associated serine/threonine-protein kinase 2 |
| Q6P1L8 | MRPL14 | 0 | 663938 | Large ribosomal subunit protein uL14m |

| | | | | |
|---|---|---|---|---|
| Q6P1Q0 | LETMD1 | 0 | 1312115 | LETM1 domain-containing protein 1 |
| Q6P1X5 | TAF2 | 5940836 | 1666300 | Transcription initiation factor TFIID subunit 2 |
| Q6P2C8 | MED27 | 6830826 | 1848513 | Mediator of RNA polymerase II transcription subunit 27 |
| Q6P2E9 | EDC4 | 60508956 | 11383796 | Enhancer of mRNA-decapping protein 4 |
| Q6P2H3 | CEP85 | 4825259 | 0 | Centrosomal protein of 85 kDa |
| Q6P2Q9 | PRPF8 | 5108887 | 16773013 | Pre-mRNA-processing-splicing factor 8 |
| Q6P474 | PDXDC2P | 0 | 1458784 | Putative pyridoxal-dependent decarboxylase domain-containing protein 2 |
| Q6P5R6 | RPL22L1 | 25379634 | 9763268 | Ribosomal protein eL22-like |
| Q6P6C2 | ALKBH5 | 804149 | 0 | RNA demethylase ALKBH5 |
| Q6PJG2 | MIDEAS | 3457191 | 41941288 | Mitotic deacetylase-associated SANT domain protein |
| Q6PKG0 | LARP1 | 90835032 | 23382454 | La-related protein 1 |
| Q6UB99 | ANKRD11 | 1443285 | 0 | Ankyrin repeat domain-containing protein 11 |
| Q6UN15 | FIP1L1 | 204046624 | 235490448 | Pre-mRNA 3'-end-processing factor FIP1 |
| Q6UUV7 | CRTC3 | 45944744 | 7025717 | CREB-regulated transcription coactivator 3 |
| Q6UWP8 | SBSN | 7178252 | 5296635 | Suprabasin |
| Q6UXN9 | WDR82 | 258842512 | 290285472 | WD repeat-containing protein 82 |
| Q6W2J9 | BCOR | 11535392 | 0 | BCL-6 corepressor |
| Q6Y7W6 | GIGYF2 | 144328928 | 7283121 | GRB10-interacting GYF protein 2 |
| Q6ZRV2 | FAM83H | 20061322 | 2311821 | Protein FAM83H |
| Q6ZV73 | FGD6 | 2596950 | 1501011 | FYVE, RhoGEF and PH domain-containing protein 6 |
| Q6ZVX7 | NCCRP1 | 7504884 | 4934171 | F-box only protein 50 |
| Q70EL1 | USP54 | 17552694 | 0 | Inactive ubiquitin carboxyl-terminal hydrolase 54 |
| Q71RC2 | LARP4 | 13272594 | 528807 | La-related protein 4 |
| Q71U36 | TUBA1A | 578473600 | 454298048 | Tubulin alpha-1A chain |
| Q71UM5 | RPS27L | 5190563 | 0 | Ribosomal protein eS27-like |
| Q7KZF4 | SND1 | 0 | 7755668 | Staphylococcal nuclease domain-containing protein 1 |

| | | | | |
|---|---|---|---|---|
| Q7L014 | DDX46 | 89921704 | 245120320 | Probable ATP-dependent RNA helicase DDX46 |
| Q7L0Y3 | TRMT10C | 0 | 16174483 | tRNA methyltransferase 10 homolog C |
| Q7L2E3 | DHX30 | 95102144 | 173475056 | ATP-dependent RNA helicase DHX30 |
| Q7L2H7 | EIF3M | 2015654 | 1501637 | Eukaryotic translation initiation factor 3 subunit M |
| Q7L576 | CYFIP1 | 0 | 1531416 | Cytoplasmic FMR1-interacting protein 1 |
| Q7RTV0 | PHF5A | 0 | 4441160 | PHD finger-like domain-containing protein 5A |
| Q7Z2T5 | TRMT1L | 64695696 | 27163252 | TRMT1-like protein |
| Q7Z2W4 | ZC3HAV1 | 941016260 | 874549380 | Zinc finger CCCH-type antiviral protein 1 |
| Q7Z3B4 | NUP54 | 0 | 262030 | Nucleoporin p54 |
| Q7Z3K3 | POGZ | 0 | 1153605 | Pogo transposable element with ZNF domain |
| Q7Z406 | MYH14 | 0 | 703696 | Myosin-14 |
| Q7Z417 | NUFIP2 | 189748928 | 63083208 | FMR1-interacting protein NUFIP2 |
| Q7Z478 | DHX29 | 816473 | 0 | ATP-dependent RNA helicase DHX29 |
| Q7Z5J4 | RAI1 | 2821734 | 802893 | Retinoic acid-induced protein 1 |
| Q7Z5K2 | WAPL | 2759672 | 0 | Wings apart-like protein homolog |
| Q7Z5L9 | IRF2BP2 | 0 | 118294368 | Interferon regulatory factor 2-binding protein 2 |
| Q7Z5Y7 | KCTD20 | 13614305 | 0 | BTB/POZ domain-containing protein KCTD20 |
| Q7Z6E9 | RBBP6 | 7008263 | 12749030 | E3 ubiquitin-protein ligase RBBP6 |
| Q7Z794 | KRT77 | 210568432 | 150330960 | Keratin, type II cytoskeletal 1b |
| Q7Z7H8 | MRPL10 | 0 | 686299 | Large ribosomal subunit protein uL10m |
| Q86SQ0 | PHLDB2 | 97745928 | 2414022 | Pleckstrin homology-like domain family B member 2 |
| Q86TB9 | PATL1 | 40680024 | 0 | Protein PAT1 homolog 1 |
| Q86TC9 | MYPN | 3882539 | 993137 | Myopalladin |
| Q86TG7 | PEG10 | 0 | 482085 | Retrotransposon-derived protein PEG10 |
| Q86U42 | PABPN1 | 1974262 | 5497353 | Polyadenylate-binding protein 2 |

| | | | | |
|---|---|---|---|---|
| Q86UE4 | MTDH | 983097 | 0 | Protein LYRIC |
| Q86UK7 | ZNF598 | 2666708 | 0 | E3 ubiquitin-protein ligase ZNF598 |
| Q86UP2 | KTN1 | 3584870 | 0 | Kinectin |
| Q86V48 | LUZP1 | 1062586 | 0 | Leucine zipper protein 1 |
| Q86V81 | ALYREF | 36772928 | 92540496 | THO complex subunit 4 |
| Q86VM9 | ZC3H18 | 0 | 4179309 | Zinc finger CCCH domain-containing protein 18 |
| Q86W92 | PPFIBP1 | 12036450 | 2811151 | Liprin-beta-1 |
| Q86WA8 | LONP2 | 5409713 | 18619322 | Lon protease homolog 2, peroxisomal |
| Q86XI2 | NCAPG2 | 0 | 1328896 | Condensin-2 complex subunit G2 |
| Q86XP3 | DDX42 | 0 | 3626049 | ATP-dependent RNA helicase DDX42 |
| Q86XZ4 | SPATS2 | 1257637 | 0 | Spermatogenesis-associated serine-rich protein 2 |
| Q86Y56 | DNAAF5 | 0 | 4879536 | Dynein axonemal assembly factor 5 |
| Q86YJ7 | ANKRD13B | 3816408 | 2204022 | Ankyrin repeat domain-containing protein 13B |
| Q86YZ3 | HRNR | 44378812 | 57344168 | Hornerin |
| Q8IV04 | TBC1D10C | 11314280 | 17100724 | Carabin |
| Q8IVS2 | MCAT | 0 | 1270064 | Malonyl-CoA-acyl carrier protein transacylase, mitochondrial |
| Q8IVT2 | MISP | 2990748 | 4191264 | Mitotic interactor and substrate of PLK1 |
| Q8IW75 | SERPINA12 | 4358005 | 1297125 | Serpin A12 |
| Q8IWK6 | ADGRA3 | 126449424 | 84984216 | Adhesion G protein-coupled receptor A3 |
| Q8IWS0 | PHF6 | 0 | 10632921 | PHD finger protein 6 |
| Q8IWX8 | CHERP | 71774936 | 61298856 | Calcium homeostasis endoplasmic reticulum protein |
| Q8IWY9 | CDAN1 | 23539812 | 24394020 | Codanin-1 |
| Q8IX01 | SUGP2 | 1394711 | 8020124 | SURP and G-patch domain-containing protein 2 |
| Q8IX12 | CCAR1 | 0 | 8031161 | Cell division cycle and apoptosis regulator protein 1 |
| Q8IXT5 | RBM12B | 12241126 | 1876904 | RNA-binding protein 12B |
| Q8IY37 | DHX37 | 2864308 | 1013169 | Probable ATP-dependent RNA helicase DHX37 |
| Q8IY67 | RAVER1 | 0 | 4283354 | Ribonucleoprotein PTB-binding 1 |

| | | | | |
|---|---|---|---|---|
| Q8IY81 | FTSJ3 | 11003462 | 10691343 | pre-rRNA 2'-O-ribose RNA methyltransferase FTSJ3 |
| Q8IYB3 | SRRM1 | 2009486 | 9298477 | Serine/arginine repetitive matrix protein 1 |
| Q8IZH2 | XRN1 | 2538426 | 769384 | 5'-3' exoribonuclease 1 |
| Q8IZL8 | PELP1 | 0 | 2271182 | Proline-, glutamic acid- and leucine-rich protein 1 |
| Q8N108 | MIER1 | 5630928 | 9029042 | Mesoderm induction early response protein 1 |
| Q8N163 | CCAR2 | 322930752 | 155640720 | Cell cycle and apoptosis regulator protein 2 |
| Q8N1F8 | STK11IP | 0 | 27096248 | Serine/threonine-protein kinase 11-interacting protein |
| Q8N1G0 | ZNF687 | 0 | 2061946 | Zinc finger protein 687 |
| Q8N1N4 | KRT78 | 107801488 | 138301120 | Keratin, type II cytoskeletal 78 |
| Q8N2M4 | TMEM86A | 0 | 4332286 | Lysoplasmalogenase TMEM86A |
| Q8N344 | MIER2 | 7350614 | 2800798 | Mesoderm induction early response protein 2 |
| Q8N3C0 | ASCC3 | 0 | 896452 | Activating signal cointegrator 1 complex subunit 3 |
| Q8N3E9 | PLCD3 | 1686403 | 0 | 1-phosphatidylinositol 4,5-bisphosphate phosphodiesterase delta-3 |
| Q8N3J3 | HROB | 2820680 | 0 | Homologous recombination OB-fold protein |
| Q8N3U4 | STAG2 | 0 | 2626787 | Cohesin subunit SA-2 |
| Q8N3V7 | SYNPO | 3759261 | 585235 | Synaptopodin |
| Q8N5F7 | NKAP | 1850906 | 542661 | NF-kappa-B-activating protein |
| Q8N684 | CPSF7 | 821305980 | 1119915520 | Cleavage and polyadenylation specificity factor subunit 7 |
| Q8N6T3 | ARFGAP1 | 1104310 | 1821892 | ADP-ribosylation factor GTPase-activating protein 1 |
| Q8N7R7 | CCNYL1 | 0 | 758359 | Cyclin-Y-like protein 1 |
| Q8N8U2 | CDYL2 | 0 | 2207898 | Chromodomain Y-like protein 2 |
| Q8NAV1 | PRPF38A | 35253996 | 38098960 | Pre-mRNA-splicing factor 38A |
| Q8NBS9 | TXNDC5 | 0 | 3670977 | Thioredoxin domain-containing protein 5 |
| Q8NC51 | SERBP1 | 118154736 | 67301608 | SERPINE1 mRNA-binding protein 1 |

| | | | | |
|---|---|---|---|---|
| Q8NC56 | LEMD2 | 0 | 5418912 | LEM domain-containing protein 2 |
| Q8NCA5 | FAM98A | 213580864 | 81145680 | Protein FAM98A |
| Q8NCF5 | NFATC2IP | 9887413 | 10987689 | NFATC2-interacting protein |
| Q8ND56 | LSM14A | 23973734 | 4201079 | Protein LSM14 homolog A |
| Q8NEM2 | SHCBP1 | 7615023 | 3868315 | SHC SH2 domain-binding protein 1 |
| Q8NEW0 | SLC30A7 | 2868254 | 3921625 | Zinc transporter 7 |
| Q8NEX9 | SDR9C7 | 3532319 | 2872650 | Short-chain dehydrogenase/reductase family 9C member 7 |
| Q8NFW8 | CMAS | 1873919 | 372443 | N-acylneuraminate cytidylyltransferase |
| Q8NI35 | PATJ | 13202036 | 578170 | InaD-like protein |
| Q8NI36 | WDR36 | 0 | 1430387 | WD repeat-containing protein 36 |
| Q8TA86 | RP9 | 675744 | 1063729 | Retinitis pigmentosa 9 protein |
| Q8TAQ2 | SMARCC2 | 2749359 | 7450756 | SWI/SNF complex subunit SMARCC2 |
| Q8TCJ2 | STT3B | 403634 | 0 | Dolichyl-diphosphooligosaccharide--protein glycosyltransferase subunit STT3B |
| Q8TD26 | CHD6 | 15236404 | 0 | Chromodomain-helicase-DNA-binding protein 6 |
| Q8TDD1 | DDX54 | 3245013 | 11919508 | ATP-dependent RNA helicase DDX54 |
| Q8TDL5 | BPIFB1 | 0 | 1675796 | BPI fold-containing family B member 1 |
| Q8TDM6 | DLG5 | 299802816 | 38931108 | Disks large homolog 5 |
| Q8TDN6 | BRIX1 | 9083912 | 10869834 | Ribosome biogenesis protein BRX1 homolog |
| Q8TE02 | ELP5 | 376688 | 0 | Elongator complex protein 5 |
| Q8TED1 | GPX8 | 3448780 | 2088549 | Probable glutathione peroxidase 8 |
| Q8TEM1 | NUP210 | 3458676 | 4923674 | Nuclear pore membrane glycoprotein 210 |
| Q8TEQ6 | GEMIN5 | 2291159 | 6663218 | Gem-associated protein 5 |
| Q8TEX9 | IPO4 | 0 | 3449938 | Importin-4 |
| Q8TF72 | SHROOM3 | 0 | 7043462 | Protein Shroom3 |
| Q8WUA2 | PPIL4 | 0 | 2969703 | Peptidyl-prolyl cis-trans isomerase-like 4 |
| Q8WUA4 | GTF3C2 | 4266258 | 5100996 | General transcription factor 3C polypeptide 2 |

| | | | | |
|---|---|---|---|---|
| Q8WUM4 | PDCD6IP | 10334896 | 58670480 | Programmed cell death 6-interacting protein |
| Q8WV44 | TRIM41 | 3383025 | 0 | E3 ubiquitin-protein ligase TRIM41 |
| Q8WVK2 | SNRNP27 | 29602150 | 28380072 | U4/U6.U5 small nuclear ribonucleoprotein 27 kDa protein |
| Q8WVV4 | POF1B | 14057268 | 14901790 | Protein POF1B |
| Q8WWI1 | LMO7 | 3192853 | 7957637 | LIM domain only protein 7 |
| Q8WWM7 | ATXN2L | 412921248 | 76211080 | Ataxin-2-like protein |
| Q8WX93 | PALLD | 39972872 | 22393306 | Palladin |
| Q8WXF1 | PSPC1 | 480360 | 2332858 | Paraspeckle component 1 |
| Q8WXI9 | GATAD2B | 0 | 11880150 | Transcriptional repressor p66-beta |
| Q8WY36 | BBX | 12361842 | 1082788 | HMG box transcription factor BBX |
| Q8WYQ5 | DGCR8 | 22087802 | 0 | Microprocessor complex subunit DGCR8 |
| Q92499 | DDX1 | 1060690620 | 391165568 | ATP-dependent RNA helicase DDX1 |
| Q92522 | H1-10 | 14096330 | 9206808 | Histone H1.10 |
| Q92541 | RTF1 | 0 | 688607810 | RNA polymerase-associated protein RTF1 homolog |
| Q92552 | MRPS27 | 737994 | 3454487 | Small ribosomal subunit protein mS27 |
| Q92567 | FAM168A | 4433324 | 6591969 | Protein FAM168A |
| Q92570 | NR4A3 | 2560618 | 0 | Nuclear receptor subfamily 4 group A member 3 |
| Q92598 | HSPH1 | 1235187 | 1811692 | Heat shock protein 105 kDa |
| Q92599 | SEPTIN8 | 0 | 1641361 | Septin-8 |
| Q92615 | LARP4B | 576584320 | 769143 | La-related protein 4B |
| Q92616 | GCN1 | 0 | 5170897 | Stalled ribosome sensor GCN1 |
| Q92620 | DHX38 | 7054096 | 8809865 | Pre-mRNA-splicing factor ATP-dependent RNA helicase PRP16 |
| Q92754 | TFAP2C | 0 | 2287754 | Transcription factor AP-2 gamma |
| Q92769 | HDAC2 | 37249404 | 44490388 | Histone deacetylase 2 |
| Q92797 | SYMPK | 15071152 | 57087876 | Symplekin |
| Q92804 | TAF15 | 5929863 | 34995284 | TATA-binding protein-associated factor 2N |
| Q92817 | EVPL | 41552740 | 59882176 | Envoplakin |
| Q92820 | GGH | 1278498 | 426976 | Gamma-glutamyl hydrolase |

| | | | | |
|---|---|---|---|---|
| Q92841 | DDX17 | 25774440 | 49825848 | Probable ATP-dependent RNA helicase DDX17 |
| Q92878 | RAD50 | 0 | 3458788 | DNA repair protein RAD50 |
| Q92879 | CELF1 | 0 | 1653292 | CUGBP Elav-like family member 1 |
| Q92888 | ARHGEF1 | 0 | 4306540 | Rho guanine nucleotide exchange factor 1 |
| Q92900 | UPF1 | 101063736 | 0 | Regulator of nonsense transcripts 1 |
| Q92908 | GATA6 | 11190089 | 0 | Transcription factor GATA-6 |
| Q92917 | GPKOW | 0 | 10301048 | G-patch domain and KOW motifs-containing protein |
| Q92945 | KHSRP | 1892527 | 6534649 | Far upstream element-binding protein 2 |
| Q92974 | ARHGEF2 | 743115140 | 187624528 | Rho guanine nucleotide exchange factor 2 |
| Q92997 | DVL3 | 14077722 | 18650452 | Segment polarity protein dishevelled homolog DVL-3 |
| Q93009 | USP7 | 2846105 | 5627645 | Ubiquitin carboxyl-terminal hydrolase 7 |
| Q93074 | MED12 | 2858262 | 5596764 | Mediator of RNA polymerase II transcription subunit 12 |
| Q969G5 | CAVIN3 | 10586776 | 198522224 | Caveolae-associated protein 3 |
| Q969L2 | MAL2 | 0 | 703132 | Protein MAL2 |
| Q969Q0 | RPL36AL | 41375584 | 15274469 | Ribosomal protein eL42-like |
| Q969X5 | ERGIC1 | 7102679 | 0 | Endoplasmic reticulum-Golgi intermediate compartment protein 1 |
| Q96A72 | MAGOHB | 14887266 | 2611243 | Protein mago nashi homolog 2 |
| Q96AE4 | FUBP1 | 0 | 1156741 | Far upstream element-binding protein 1 |
| Q96AG4 | LRRC59 | 406006 | 4438887 | Leucine-rich repeat-containing protein 59 |
| Q96AQ6 | PBXIP1 | 2465579 | 3218799 | Pre-B-cell leukemia transcription factor-interacting protein 1 |
| Q96CP2 | FLYWCH2 | 0 | 10105634 | FLYWCH family member 2 |
| Q96CP6 | GRAMD1A | 2951271 | 973309 | Protein Aster-A |
| Q96CS3 | FAF2 | 0 | 743167 | FAS-associated factor 2 |
| Q96CW1 | AP2M1 | 7397209 | 7052767 | AP-2 complex subunit mu |
| Q96CW5 | TUBGCP3 | 2622187 | 0 | Gamma-tubulin complex component 3 |
| Q96D15 | RCN3 | 34286552 | 25130172 | Reticulocalbin-3 |

| | | | | |
|---|---|---|---|---|
| Q96D71 | REPS1 | 8238184 | 1432959 | RalBP1-associated Eps domain-containing protein 1 |
| Q96DH6 | MSI2 | 1092220 | 0 | RNA-binding protein Musashi homolog 2 |
| Q96DI7 | SNRNP40 | 0 | 790210 | U5 small nuclear ribonucleoprotein 40 kDa protein |
| Q96DR8 | MUCL1 | 2731612 | 1728262 | Mucin-like protein 1 |
| Q96DT7 | ZBTB10 | 26866890 | 1204761 | Zinc finger and BTB domain-containing protein 10 |
| Q96E39 | RBMXL1 | 0 | 14495250 | RNA binding motif protein, X-linked-like-1 |
| Q96EP5 | DAZAP1 | 0 | 1327175 | DAZ-associated protein 1 |
| Q96EU6 | RRP36 | 8410566 | 2573586 | Ribosomal RNA processing protein 36 homolog |
| Q96EV2 | RBM33 | 56358476 | 54300828 | RNA-binding protein 33 |
| Q96EY1 | DNAJA3 | 8616940 | 2032885 | DnaJ homolog subfamily A member 3, mitochondrial |
| Q96F86 | EDC3 | 30968844 | 0 | Enhancer of mRNA-decapping protein 3 |
| Q96FQ6 | S100A16 | 0 | 3916565 | Protein S100-A16 |
| Q96FZ2 | HMCES | 0 | 2911288 | Abasic site processing protein HMCES |
| Q96GQ7 | DDX27 | 4098039 | 1555399 | Probable ATP-dependent RNA helicase DDX27 |
| Q96HC4 | PDLIM5 | 1881675 | 16980924 | PDZ and LIM domain protein 5 |
| Q96HH9 | GRAMD2B | 0 | 2661962 | GRAM domain-containing protein 2B |
| Q96HR3 | MED30 | 10863232 | 2793714 | Mediator of RNA polymerase II transcription subunit 30 |
| Q96HS1 | PGAM5 | 199359200 | 172432416 | Serine/threonine-protein phosphatase PGAM5, mitochondrial |
| Q96I24 | FUBP3 | 0 | 2442623 | Far upstream element-binding protein 3 |
| Q96I25 | RBM17 | 75897104 | 69966528 | Splicing factor 45 |
| Q96IF1 | AJUBA | 1673102 | 677895 | LIM domain-containing protein ajuba |
| Q96IX5 | ATP5MK | 2866909 | 4174420 | ATP synthase membrane subunit K, mitochondrial |
| Q96JN8 | NEURL4 | 4008265 | 0 | Neuralized-like protein 4 |
| Q96KG9 | SCYL1 | 23462626 | 63237988 | N-terminal kinase-like protein |
| Q96KQ7 | EHMT2 | 22469612 | 12264915 | Histone-lysine N-methyltransferase EHMT2 |

| | | | | |
|---|---|---|---|---|
| Q96KR1 | ZFR | 0 | 1094957 | Zinc finger RNA-binding protein |
| Q96L46 | CAPNS2 | 3189558 | 2982819 | Calpain small subunit 2 |
| Q96LD4 | TRIM47 | 0 | 47436636 | E3 ubiquitin-protein ligase TRIM47 |
| Q96MU7 | YTHDC1 | 4933285 | 5056886 | YTH domain-containing protein 1 |
| Q96N67 | DOCK7 | 1960136 | 994843 | Dedicator of cytokinesis protein 7 |
| Q96P63 | SERPINB12 | 99271944 | 132161224 | Serpin B12 |
| Q96PC5 | MIA2 | 0 | 3206697 | Melanoma inhibitory activity protein 2 |
| Q96PE2 | ARHGEF17 | 2515553 | 0 | Rho guanine nucleotide exchange factor 17 |
| Q96PK6 | RBM14 | 106441328 | 376702464 | RNA-binding protein 14 |
| Q96PU8 | QKI | 0 | 3190147 | KH domain-containing RNA-binding protein QKI |
| Q96PV6 | LENG8 | 86888288 | 36521112 | Leukocyte receptor cluster member 8 |
| Q96PY5 | FMNL2 | 4443584 | 2936115 | Formin-like protein 2 |
| Q96QA5 | GSDMA | 22728752 | 28469052 | Gasdermin-A |
| Q96QC0 | PPP1R10 | 2082244480 | 1653716220 | Serine/threonine-protein phosphatase 1 regulatory subunit 10 |
| Q96RE7 | NACC1 | 1290328 | 0 | Nucleus accumbens-associated protein 1 |
| Q96RN5 | MED15 | 4817752 | 0 | Mediator of RNA polymerase II transcription subunit 15 |
| Q96RR1 | TWNK | 4165152 | 2892803 | Twinkle mtDNA helicase |
| Q96S55 | WRNIP1 | 0 | 9408712 | ATPase WRNIP1 |
| Q96SB4 | SRPK1 | 62850280 | 1319207 | SRSF protein kinase 1 |
| Q96SN8 | CDK5RAP2 | 2624497 | 0 | CDK5 regulatory subunit-associated protein 2 |
| Q96T37 | RBM15 | 6451397 | 9292248 | RNA-binding protein 15 |
| Q96T76 | MMS19 | 0 | 2220000 | MMS19 nucleotide excision repair protein homolog |
| Q99459 | CDC5L | 19241530 | 5265399 | Cell division cycle 5-like protein |
| Q99460 | PSMD1 | 0 | 1499778 | 26S proteasome non-ATPase regulatory subunit 1 |
| Q99496 | RNF2 | 3205475 | 2103290 | E3 ubiquitin-protein ligase RING2 |
| Q99497 | PARK7 | 2834246 | 3773477 | Parkinson disease protein 7 |
| Q99569 | PKP4 | 10454032 | 2246577 | Plakophilin-4 |

| | | | | |
|---|---|---|---|---|
| Q99575 | POP1 | 11010803 | 4752511 | Ribonucleases P/MRP protein subunit POP1 |
| Q99615 | DNAJC7 | 0 | 8606086 | DnaJ homolog subfamily C member 7 |
| Q99623 | PHB2 | 6270210 | 4594889 | Prohibitin-2 |
| Q99700 | ATXN2 | 5877505 | 0 | Ataxin-2 |
| Q99714 | HSD17B10 | 0 | 4122407 | 3-hydroxyacyl-CoA dehydrogenase type-2 |
| Q99729 | HNRNPAB | 0 | 5838839 | Heterogeneous nuclear ribonucleoprotein A/B |
| Q99735 | MGST2 | 0 | 824262 | Microsomal glutathione S-transferase 2 |
| Q99829 | CPNE1 | 0 | 784027 | Copine-1 |
| Q99832 | CCT7 | 9653504 | 8778153 | T-complex protein 1 subunit eta |
| Q99848 | EBNA1BP2 | 3440999 | 22611244 | Probable rRNA-processing protein EBP2 |
| Q99873 | PRMT1 | 2891360 | 6839838 | Protein arginine N-methyltransferase 1 |
| Q99958 | FOXC2 | 4283157 | 5762176 | Forkhead box protein C2 |
| Q99959 | PKP2 | 7239332 | 86413256 | Plakophilin-2 |
| Q9BPW8 | NIPSNAP1 | 0 | 17939826 | Protein NipSnap homolog 1 |
| Q9BQ39 | DDX50 | 18477722 | 21831512 | ATP-dependent RNA helicase DDX50 |
| Q9BQ48 | MRPL34 | 0 | 780836 | Large ribosomal subunit protein bL34m |
| Q9BQ67 | GRWD1 | 0 | 4867287 | Glutamate-rich WD repeat-containing protein 1 |
| Q9BQA1 | WDR77 | 3381603 | 5062641 | Methylosome protein WDR77 |
| Q9BQG0 | MYBBP1A | 44084384 | 20455194 | Myb-binding protein 1A |
| Q9BRA2 | TXNDC17 | 0 | 7227334 | Thioredoxin domain-containing protein 17 |
| Q9BRK5 | SDF4 | 1481279 | 7398841 | 45 kDa calcium-binding protein |
| Q9BRS2 | RIOK1 | 0 | 1913095 | Serine/threonine-protein kinase RIO1 |
| Q9BSF8 | BTBD10 | 12722156 | 0 | BTB/POZ domain-containing protein 10 |
| Q9BSJ2 | TUBGCP2 | 9312320 | 11420294 | Gamma-tubulin complex component 2 |
| Q9BT78 | COPS4 | 0 | 328950 | COP9 signalosome complex subunit 4 |
| Q9BTC0 | DIDO1 | 9336140 | 2519837 | Death-inducer obliterator 1 |
| Q9BTD8 | RBM42 | 0 | 1660689 | RNA-binding protein 42 |

| | | | | |
|---|---|---|---|---|
| Q9BTE3 | MCMBP | 0 | 1263832 | Mini-chromosome maintenance complex-binding protein |
| Q9BTT0 | ANP32E | 1067165 | 3864467 | Acidic leucine-rich nuclear phosphoprotein 32 family member E |
| Q9BTT4 | MED10 | 12450253 | 3669280 | Mediator of RNA polymerase II transcription subunit 10 |
| Q9BTV4 | TMEM43 | 2256713 | 1785565 | Transmembrane protein 43 |
| Q9BU76 | MMTAG2 | 10088557 | 8489917 | Multiple myeloma tumor-associated protein 2 |
| Q9BUF5 | TUBB6 | 40464304 | 7510965 | Tubulin beta-6 chain |
| Q9BUJ2 | HNRNPUL1 | 20435638 | 6500177 | Heterogeneous nuclear ribonucleoprotein U-like protein 1 |
| Q9BUQ8 | DDX23 | 3818694 | 13113436 | Probable ATP-dependent RNA helicase DDX23 |
| Q9BV38 | WDR18 | 0 | 2534920 | WD repeat-containing protein 18 |
| Q9BVG8 | KIFC3 | 11462138 | 3066367 | Kinesin-like protein KIFC3 |
| Q9BVI4 | NOC4L | 4284724 | 7347799 | Nucleolar complex protein 4 homolog |
| Q9BVJ6 | UTP14A | 2996757 | 1100866 | U3 small nucleolar RNA-associated protein 14 homolog A |
| Q9BVK6 | TMED9 | 0 | 2583514 | Transmembrane emp24 domain-containing protein 9 |
| Q9BVP2 | GNL3 | 56977112 | 40950852 | Guanine nucleotide-binding protein-like 3 |
| Q9BWF3 | RBM4 | 48386720 | 7414460 | RNA-binding protein 4 |
| Q9BWJ5 | SF3B5 | 0 | 5343781 | Splicing factor 3B subunit 5 |
| Q9BXF6 | RAB11FIP5 | 4802972 | 0 | Rab11 family-interacting protein 5 |
| Q9BXL6 | CARD14 | 4670224 | 0 | Caspase recruitment domain-containing protein 14 |
| Q9BXP5 | SRRT | 11181856 | 93895168 | Serrate RNA effector molecule homolog |
| Q9BXS6 | NUSAP1 | 0 | 735437 | Nucleolar and spindle-associated protein 1 |
| Q9BY44 | EIF2A | 5235655 | 11123147 | Eukaryotic translation initiation factor 2A |
| Q9BY77 | POLDIP3 | 0 | 14324911 | Polymerase delta-interacting protein 3 |
| Q9BYD1 | MRPL13 | 6441448 | 0 | Large ribosomal subunit protein uL13m |

| | | | | |
|---|---|---|---|---|
| Q9BYG3 | NIFK | 2055397 | 34988716 | MKI67 FHA domain-interacting nucleolar phosphoprotein |
| Q9BYJ1 | ALOXE3 | 1810092 | 2005001 | Hydroperoxide isomerase ALOXE3 |
| Q9BZE4 | GTPBP4 | 8382587 | 12596317 | GTP-binding protein 4 |
| Q9BZK7 | TBL1XR1 | 13161998 | 22947930 | F-box-like/WD repeat-containing protein TBL1XR1 |
| Q9C075 | KRT23 | 3380917 | 6033129 | Keratin, type I cytoskeletal 23 |
| Q9C0C2 | TNKS1BP1 | 17613490 | 2240231 | 182 kDa tankyrase-1-binding protein |
| Q9C0D7 | ZC3H12C | 1627548 | 0 | Probable ribonuclease ZC3H12C |
| Q9C0J8 | WDR33 | 268411632 | 225828096 | pre-mRNA 3' end processing protein WDR33 |
| Q9GZT3 | SLIRP | 0 | 2196243 | SRA stem-loop-interacting RNA-binding protein, mitochondrial |
| Q9H000 | MKRN2 | 2159069 | 2930923 | E3 ubiquitin-protein ligase makorin-2 |
| Q9H074 | PAIP1 | 10147335 | 2062673 | Polyadenylate-binding protein-interacting protein 1 |
| Q9H089 | LSG1 | 2211001 | 9100445 | Large subunit GTPase 1 homolog |
| Q9H0A0 | NAT10 | 1424719 | 8199485 | RNA cytidine acetyltransferase |
| Q9H0D6 | XRN2 | 5150469 | 2993381 | 5'-3' exoribonuclease 2 |
| Q9H0E2 | TOLLIP | 2782787 | 0 | Toll-interacting protein |
| Q9H0G5 | NSRP1 | 1342234 | 423364 | Nuclear speckle splicing regulatory protein 1 |
| Q9H0H5 | RACGAP1 | 3472190 | 8340437 | Rac GTPase-activating protein 1 |
| Q9H0U3 | MAGT1 | 178763920 | 84924448 | Magnesium transporter protein 1 |
| Q9H0W9 | C11orf54 | 0 | 580208 | Ester hydrolase C11orf54 |
| Q9H1K4 | SLC25A18 | 0 | 614265 | Mitochondrial glutamate carrier 2 |
| Q9H2H8 | PPIL3 | 874810 | 3148122 | Peptidyl-prolyl cis-trans isomerase-like 3 |
| Q9H2P0 | ADNP | 0 | 6327459 | Activity-dependent neuroprotector homeobox protein |
| Q9H329 | EPB41L4B | 4224091 | 767823 | Band 4.1-like protein 4B |
| Q9H3K6 | BOLA2B | 0 | 229761 | BolA-like protein 2 |
| Q9H3Q1 | CDC42EP4 | 1814165 | 4009359 | Cdc42 effector protein 4 |
| Q9H3U1 | UNC45A | 0 | 2975674 | Protein unc-45 homolog A |

| | | | | |
|---|---|---|---|---|
| Q9H444 | CHMP4B | 758128 | 4645642 | Charged multivesicular body protein 4b |
| Q9H4I3 | TRABD | 0 | 5100740 | TraB domain-containing protein |
| Q9H5V9 | STEEP1 | 2048199 | 0 | STING ER exit protein |
| Q9H694 | BICC1 | 1117423 | 0 | Protein bicaudal C homolog 1 |
| Q9H6R4 | NOL6 | 4390485 | 4767853 | Nucleolar protein 6 |
| Q9H6S1 | AZI2 | 1574375 | 0 | 5-azacytidine-induced protein 2 |
| Q9H6T0 | ESRP2 | 209115 | 0 | Epithelial splicing regulatory protein 2 |
| Q9H7B2 | RPF2 | 9101588 | 4582995 | Ribosome production factor 2 homolog |
| Q9H7E9 | C8orf33 | 0 | 3592358 | UPF0488 protein C8orf33 |
| Q9H944 | MED20 | 129430048 | 2546543 | Mediator of RNA polymerase II transcription subunit 20 |
| Q9H9A6 | LRRC40 | 0 | 2566062 | Leucine-rich repeat-containing protein 40 |
| Q9H9B1 | EHMT1 | 18721376 | 29555950 | Histone-lysine N-methyltransferase EHMT1 |
| Q9H9F9 | ACTR5 | 0 | 415199 | Actin-related protein 5 |
| Q9HAN9 | NMNAT1 | 16096794 | 6721797 | Nicotinamide/nicotinic acid mononucleotide adenylyltransferase 1 |
| Q9HAV4 | XPO5 | 0 | 2203632 | Exportin-5 |
| Q9HAW4 | CLSPN | 5484734 | 0 | Claspin |
| Q9HB71 | CACYBP | 1561516 | 1805762 | Calcyclin-binding protein |
| Q9HC36 | MRM3 | 0 | 493547 | rRNA methyltransferase 3, mitochondrial |
| Q9HC44 | GPBP1L1 | 16578401 | 0 | Vasculin-like protein 1 |
| Q9HC52 | CBX8 | 8783559 | 0 | Chromobox protein homolog 8 |
| Q9HCD6 | TANC2 | 7474114 | 0 | Protein TANC2 |
| Q9HCE1 | MOV10 | 235931728 | 20068376 | Helicase MOV-10 |
| Q9HCK8 | CHD8 | 5342724 | 8022377 | Chromodomain-helicase-DNA-binding protein 8 |
| Q9HCM4 | EPB41L5 | 6880172 | 3334732 | Band 4.1-like protein 5 |
| Q9HCY8 | S100A14 | 7173787 | 8317873 | Protein S100-A14 |
| Q9HD33 | MRPL47 | 3104519 | 1167579 | Large ribosomal subunit protein uL29m |
| Q9HDC5 | JPH1 | 131252440 | 5874340 | Junctophilin-1 |
| Q9NP79 | VTA1 | 944025 | 0 | Vacuolar protein sorting-associated protein VTA1 homolog |

| | | | | |
|---|---|---|---|---|
| Q9NPD3 | EXOSC4 | 1857928 | 4896294 | Exosome complex component RRP41 |
| Q9NPJ6 | MED4 | 5338088 | 2581294 | Mediator of RNA polymerase II transcription subunit 4 |
| Q9NQ29 | LUC7L | 18091916 | 17188454 | Putative RNA-binding protein Luc7-like 1 |
| Q9NQ55 | PPAN | 4039693 | 1290293 | Suppressor of SWI4 1 homolog |
| Q9NQT5 | EXOSC3 | 0 | 4042842 | Exosome complex component RRP40 |
| Q9NQZ2 | UTP3 | 662247 | 0 | Something about silencing protein 10 |
| Q9NR30 | DDX21 | 161614224 | 314002048 | Nucleolar RNA helicase 2 |
| Q9NR31 | SAR1A | 0 | 3494253 | GTP-binding protein SAR1a |
| Q9NRL2 | BAZ1A | 0 | 2082341 | Bromodomain adjacent to zinc finger domain protein 1A |
| Q9NRR4 | DROSHA | 32244684 | 5862674 | Ribonuclease 3 |
| Q9NSI2 | SLX9 | 882919 | 2854000 | Ribosome biogenesis protein SLX9 homolog |
| Q9NUI1 | DECR2 | 417908 | 1274224 | Peroxisomal 2,4-dienoyl-CoA reductase [(3E)-enoyl-CoA-producing] |
| Q9NUL3 | STAU2 | 16371511 | 4091048 | Double-stranded RNA-binding protein Staufen homolog 2 |
| Q9NUL7 | DDX28 | 15209197 | 27106678 | Probable ATP-dependent RNA helicase DDX28 |
| Q9NUU7 | DDX19A | 0 | 1864226 | ATP-dependent RNA helicase DDX19A |
| Q9NV31 | IMP3 | 4097105 | 4199024 | U3 small nucleolar ribonucleoprotein protein IMP3 |
| Q9NVC6 | MED17 | 17826076 | 5806844 | Mediator of RNA polymerase II transcription subunit 17 |
| Q9NVI7 | ATAD3A | 117089120 | 62922456 | ATPase family AAA domain-containing protein 3A |
| Q9NVN8 | GNL3L | 9301068 | 5378557 | Guanine nucleotide-binding protein-like 3-like protein |
| Q9NVP1 | DDX18 | 0 | 538139 | ATP-dependent RNA helicase DDX18 |
| Q9NVV4 | MTPAP | 1466106 | 3192097 | Poly(A) RNA polymerase, mitochondrial |

| | | | | |
|---|---|---|---|---|
| Q9NW13 | RBM28 | 2823514 | 817727 | RNA-binding protein 28 |
| Q9NW75 | GPATCH2 | 5627926 | 0 | G patch domain-containing protein 2 |
| Q9NW82 | WDR70 | 0 | 1132296 | WD repeat-containing protein 70 |
| Q9NWB6 | ARGLU1 | 0 | 2438091 | Arginine and glutamate-rich protein 1 |
| Q9NWF9 | RNF216 | 37595760 | 0 | E3 ubiquitin-protein ligase RNF216 |
| Q9NWH9 | SLTM | 1838245 | 4855265 | SAFB-like transcription modulator |
| Q9NWQ4 | GPATCH2L | 4332136 | 0 | G patch domain-containing protein 2-like |
| Q9NWU5 | MRPL22 | 1724122 | 15739826 | Large ribosomal subunit protein uL22m |
| Q9NX24 | NHP2 | 3898103 | 2192680 | H/ACA ribonucleoprotein complex subunit 2 |
| Q9NX63 | CHCHD3 | 2309213 | 8392216 | MICOS complex subunit MIC19 |
| Q9NX70 | MED29 | 5293689 | 2940978 | Mediator of RNA polymerase II transcription subunit 29 |
| Q9NX95 | SYBU | 2047590 | 1072451 | Syntabulin |
| Q9NXE8 | CWC25 | 2814999 | 0 | Pre-mRNA-splicing factor CWC25 homolog |
| Q9NXH9 | TRMT1 | 0 | 1246721 | tRNA (guanine(26)-N(2))-dimethyltransferase |
| Q9NXS2 | QPCTL | 6455932 | 0 | Glutaminyl-peptide cyclotransferase-like protein |
| Q9NY12 | GAR1 | 24562944 | 10729090 | H/ACA ribonucleoprotein complex subunit 1 |
| Q9NY33 | DPP3 | 3455148 | 2974311 | Dipeptidyl peptidase 3 |
| Q9NYD6 | HOXC10 | 5975393 | 0 | Homeobox protein Hox-C10 |
| Q9NYF8 | BCLAF1 | 15373712 | 44522544 | Bcl-2-associated transcription factor 1 |
| Q9NYK1 | TLR7 | 1675495170 | 1148394620 | Toll-like receptor 7 |
| Q9NYL2 | MAP3K20 | 1635691 | 0 | Mitogen-activated protein kinase kinase kinase 20 |
| Q9NYL9 | TMOD3 | 4080808 | 2735393 | Tropomodulin-3 |
| Q9NYV4 | CDK12 | 893290 | 5182872 | Cyclin-dependent kinase 12 |
| Q9NZ01 | TECR | 3663587 | 8307642 | Very-long-chain enoyl-CoA reductase |
| Q9NZB2 | FAM120A | 39160072 | 7368942 | Constitutive coactivator of PPAR-gamma-like protein 1 |

| | | | | |
|---|---|---|---|---|
| Q9NZI8 | IGF2BP1 | 5513360 | 502784 | Insulin-like growth factor 2 mRNA-binding protein 1 |
| Q9NZL4 | HSPBP1 | 610886 | 4590083 | Hsp70-binding protein 1 |
| Q9NZT1 | CALML5 | 13446874 | 5240700 | Calmodulin-like protein 5 |
| Q9P031 | CCDC59 | 32580926 | 4576602 | Thyroid transcription factor 1-associated protein 26 |
| Q9P086 | MED11 | 8169724 | 3155960 | Mediator of RNA polymerase II transcription subunit 11 |
| Q9P206 | KIAA1522 | 3470013 | 0 | Uncharacterized protein KIAA1522 |
| Q9P258 | RCC2 | 3901099 | 12789078 | Protein RCC2 |
| Q9P270 | SLAIN2 | 19746244 | 5541772 | SLAIN motif-containing protein 2 |
| Q9P2D1 | CHD7 | 75323480 | 31371040 | Chromodomain-helicase-DNA-binding protein 7 |
| Q9P2E9 | RRBP1 | 0 | 10087585 | Ribosome-binding protein 1 |
| Q9P2I0 | CPSF2 | 229352336 | 224431264 | Cleavage and polyadenylation specificity factor subunit 2 |
| Q9P2J5 | LARS1 | 8726289 | 10142574 | Leucine--tRNA ligase, cytoplasmic |
| Q9P2N5 | RBM27 | 24085468 | 20190856 | RNA-binding protein 27 |
| Q9P2R6 | RERE | 38471312 | 30071808 | Arginine-glutamic acid dipeptide repeats protein |
| Q9UBB4 | ATXN10 | 1315193 | 5248354 | Ataxin-10 |
| Q9UBQ5 | EIF3K | 5809168 | 3288948 | Eukaryotic translation initiation factor 3 subunit K |
| Q9UBV8 | PEF1 | 0 | 7051858 | Peflin |
| Q9UBX3 | SLC25A10 | 39317380 | 5429262 | Mitochondrial dicarboxylate carrier |
| Q9UDY2 | TJP2 | 360615744 | 229959376 | Tight junction protein ZO-2 |
| Q9UG63 | ABCF2 | 1573807 | 0 | ATP-binding cassette sub-family F member 2 |
| Q9UGI8 | TES | 0 | 34499544 | Testin |
| Q9UGM3 | DMBT1 | 0 | 1995346 | Deleted in malignant brain tumors 1 protein |
| Q9UGP4 | LIMD1 | 8326218 | 0 | LIM domain-containing protein 1 |
| Q9UGU0 | TCF20 | 7083501 | 6582778 | Transcription factor 20 |
| Q9UHB6 | LIMA1 | 0 | 6439694 | LIM domain and actin-binding protein 1 |
| Q9UHB7 | AFF4 | 4580301 | 0 | AF4/FMR2 family member 4 |
| Q9UHB9 | SRP68 | 4135782 | 2201553 | Signal recognition particle subunit SRP68 |

| | | | | |
|---|---|---|---|---|
| Q9UHD2 | TBK1 | 2700992 | 0 | Serine/threonine-protein kinase TBK1 |
| Q9UHD8 | SEPTIN9 | 0 | 1148105 | Septin-9 |
| Q9UHI6 | DDX20 | 3265422 | 8760639 | Probable ATP-dependent RNA helicase DDX20 |
| Q9UHL4 | DPP7 | 10506707 | 0 | Dipeptidyl peptidase 2 |
| Q9UHL9 | GTF2IRD1 | 3942141 | 0 | General transcription factor II-I repeat domain-containing protein 1 |
| Q9UHQ9 | CYB5R1 | 0 | 3553265 | NADH-cytochrome b5 reductase 1 |
| Q9UHV7 | MED13 | 2146117 | 2998379 | Mediator of RNA polymerase II transcription subunit 13 |
| Q9UHX1 | PUF60 | 8132982 | 50667452 | Poly(U)-binding-splicing factor PUF60 |
| Q9UI30 | TRMT112 | 0 | 1914651 | Multifunctional methyltransferase subunit TRM112-like protein |
| Q9UI42 | CPA4 | 222904608 | 132418304 | Carboxypeptidase A4 |
| Q9UIF9 | BAZ2A | 4632258 | 9291989 | Bromodomain adjacent to zinc finger domain protein 2A |
| Q9UIG0 | BAZ1B | 0 | 431546 | Tyrosine-protein kinase BAZ1B |
| Q9UII4 | HERC5 | 2894615 | 275534 | E3 ISG15--protein ligase HERC5 |
| Q9UJG1 | MOSPD1 | 0 | 17446032 | Motile sperm domain-containing protein 1 |
| Q9UJS0 | SLC25A13 | 10754058 | 5857428 | Electrogenic aspartate/glutamate antiporter SLC25A13, mitochondrial |
| Q9UJV9 | DDX41 | 9291527 | 4622422 | Probable ATP-dependent RNA helicase DDX41 |
| Q9UJX3 | ANAPC7 | 0 | 2162367 | Anaphase-promoting complex subunit 7 |
| Q9UK61 | TASOR | 1529950 | 13420250 | Protein TASOR |
| Q9UKF6 | CPSF3 | 294828288 | 339170624 | Cleavage and polyadenylation specificity factor subunit 3 |
| Q9UKK9 | NUDT5 | 1543989 | 1737931 | ADP-sugar pyrophosphatase |
| Q9UKL0 | RCOR1 | 3154730 | 11598294 | REST corepressor 1 |
| Q9UKM9 | RALY | 3518440 | 34705908 | RNA-binding protein Raly |
| Q9UKN8 | GTF3C4 | 13667864 | 1240006 | General transcription factor 3C polypeptide 4 |

| | | | | |
|---|---|---|---|---|
| Q9UKS6 | PACSIN3 | 23737394 | 7275268 | Protein kinase C and casein kinase substrate in neurons protein 3 |
| Q9UKV8 | AGO2 | 4225933 | 0 | Protein argonaute-2 |
| Q9ULC6 | PADI1 | 0 | 604625 | Protein-arginine deiminase type-1 |
| Q9ULD2 | MTUS1 | 2704607 | 802599 | Microtubule-associated tumor suppressor 1 |
| Q9ULK4 | MED23 | 8746954 | 2175077 | Mediator of RNA polymerase II transcription subunit 23 |
| Q9ULU4 | ZMYND8 | 0 | 2382776 | MYND-type zinc finger-containing chromatin reader ZMYND8 |
| Q9ULV0 | MYO5B | 4136721 | 0 | Unconventional myosin-Vb |
| Q9ULW0 | TPX2 | 4347797 | 655548 | Targeting protein for Xklp2 |
| Q9ULX3 | NOB1 | 18012678 | 4188251 | RNA-binding protein NOB1 |
| Q9ULX6 | AKAP8L | 83747312 | 32841748 | A-kinase anchor protein 8-like |
| Q9UM00 | TMCO1 | 0 | 1506564 | Calcium load-activated calcium channel |
| Q9UM54 | MYO6 | 3649312 | 0 | Unconventional myosin-VI |
| Q9UMS4 | PRPF19 | 3810494 | 8498290 | Pre-mRNA-processing factor 19 |
| Q9UN86 | G3BP2 | 20409268 | 5402420 | Ras GTPase-activating protein-binding protein 2 |
| Q9UNE7 | STUB1 | 0 | 19955398 | E3 ubiquitin-protein ligase CHIP |
| Q9UNF1 | MAGED2 | 0 | 9165040 | Melanoma-associated antigen D2 |
| Q9UNQ2 | DIMT1 | 0 | 564458 | Probable dimethyladenosine transferase |
| Q9UNX3 | RPL26L1 | 168856112 | 56450100 | Ribosomal protein uL24-like |
| Q9UPQ0 | LIMCH1 | 9063451 | 2943251 | LIM and calponin homology domains-containing protein 1 |
| Q9UPT8 | ZC3H4 | 209367536 | 164667296 | Zinc finger CCCH domain-containing protein 4 |
| Q9UQ35 | SRRM2 | 8025102 | 25329698 | Serine/arginine repetitive matrix protein 2 |
| Q9UQ80 | PA2G4 | 0 | 2180345 | Proliferation-associated protein 2G4 |
| Q9UQE7 | SMC3 | 0 | 452184 | Structural maintenance of chromosomes protein 3 |
| Q9Y224 | RTRAF | 404743168 | 138818368 | RNA transcription, translation and transport factor protein |

| | | | | |
|---|---|---|---|---|
| Q9Y230 | RUVBL2 | 67287640 | 52168020 | RuvB-like 2 |
| Q9Y232 | CDYL | 55250476 | 83141568 | Chromodomain Y-like protein |
| Q9Y262 | EIF3L | 41299472 | 10085985 | Eukaryotic translation initiation factor 3 subunit L |
| Q9Y263 | PLAA | 0 | 998334 | Phospholipase A-2-activating protein |
| Q9Y265 | RUVBL1 | 40740520 | 42065164 | RuvB-like 1 |
| Q9Y2H6 | FNDC3A | 0 | 340205 | Fibronectin type-III domain-containing protein 3A |
| Q9Y2R5 | MRPS17 | 2410196 | 2361837 | Small ribosomal subunit protein uS17m |
| Q9Y2R9 | MRPS7 | 902245 | 11584604 | Small ribosomal subunit protein uS7m |
| Q9Y2V2 | CARHSP1 | 0 | 20597144 | Calcium-regulated heat-stable protein 1 |
| Q9Y2W1 | THRAP3 | 10601952 | 68094264 | Thyroid hormone receptor-associated protein 3 |
| Q9Y2W2 | WBP11 | 0 | 1070484 | WW domain-binding protein 11 |
| Q9Y2X0 | MED16 | 14031188 | 6089259 | Mediator of RNA polymerase II transcription subunit 16 |
| Q9Y2X3 | NOP58 | 4801031 | 2934397 | Nucleolar protein 58 |
| Q9Y2X7 | GIT1 | 35699848 | 20435712 | ARF GTPase-activating protein GIT1 |
| Q9Y2Z0 | SUGT1 | 0 | 3092149 | Protein SGT1 homolog |
| Q9Y305 | ACOT9 | 0 | 1186541 | Acyl-coenzyme A thioesterase 9, mitochondrial |
| Q9Y314 | NOSIP | 0 | 3562208 | Nitric oxide synthase-interacting protein |
| Q9Y383 | LUC7L2 | 124997104 | 157855952 | Putative RNA-binding protein Luc7-like 2 |
| Q9Y3B4 | SF3B6 | 0 | 7044770 | Splicing factor 3B subunit 6 |
| Q9Y3C6 | PPIL1 | 2625946 | 0 | Peptidyl-prolyl cis-trans isomerase-like 1 |
| Q9Y3C7 | MED31 | 11746011 | 6789323 | Mediator of RNA polymerase II transcription subunit 31 |
| Q9Y3D9 | MRPS23 | 0 | 3760110 | Small ribosomal subunit protein mS23 |
| Q9Y3F4 | STRAP | 8329185 | 47770560 | Serine-threonine kinase receptor-associated protein |
| Q9Y3I0 | RTCB | 1059167550 | 363245408 | RNA-splicing ligase RtcB homolog |

| | | | | |
|---|---|---|---|---|
| Q9Y3U8 | RPL36 | 106225056 | 70882096 | Large ribosomal subunit protein eL36 |
| Q9Y3X0 | CCDC9 | 8381718 | 5539254 | Coiled-coil domain-containing protein 9 |
| Q9Y3Y2 | CHTOP | 1595168 | 7059697 | Chromatin target of PRMT1 protein |
| Q9Y3Z3 | SAMHD1 | 4986521 | 3219157 | Deoxynucleoside triphosphate triphosphohydrolase SAMHD1 |
| Q9Y446 | PKP3 | 604550340 | 215707792 | Plakophilin-3 |
| Q9Y448 | KNSTRN | 1442016 | 0 | Small kinetochore-associated protein |
| Q9Y450 | HBS1L | 4922829 | 2073273 | HBS1-like protein |
| Q9Y4B5 | MTCL1 | 14176131 | 0 | Microtubule cross-linking factor 1 |
| Q9Y4B6 | DCAF1 | 31171130 | 259525056 | DDB1- and CUL4-associated factor 1 |
| Q9Y4H2 | IRS2 | 1988778 | 0 | Insulin receptor substrate 2 |
| Q9Y4I1 | MYO5A | 75140392 | 19068940 | Unconventional myosin-Va |
| Q9Y4P3 | TBL2 | 3959708 | 0 | Transducin beta-like protein 2 |
| Q9Y512 | SAMM50 | 0 | 1676857 | Sorting and assembly machinery component 50 homolog |
| Q9Y520 | PRRC2C | 44026068 | 27583574 | Protein PRRC2C |
| Q9Y5A9 | YTHDF2 | 3769798 | 0 | YTH domain-containing family protein 2 |
| Q9Y5B9 | SUPT16H | 0 | 6207937 | FACT complex subunit SPT16 |
| Q9Y5Q8 | GTF3C5 | 13117659 | 12009674 | General transcription factor 3C polypeptide 5 |
| Q9Y5Q9 | GTF3C3 | 4586426 | 4269244 | General transcription factor 3C polypeptide 3 |
| Q9Y5S9 | RBM8A | 33500238 | 2066914 | RNA-binding protein 8A |
| Q9Y5U2 | TSSC4 | 0 | 214733 | U5 small nuclear ribonucleoprotein TSSC4 |
| Q9Y5X1 | SNX9 | 92167736 | 37912896 | Sorting nexin-9 |
| Q9Y5Y5 | PEX16 | 0 | 239233 | Peroxisomal membrane protein PEX16 |
| Q9Y618 | NCOR2 | 5622678 | 16071120 | Nuclear receptor corepressor 2 |
| Q9Y676 | MRPS18B | 0 | 2207022 | Small ribosomal subunit protein mS40 |
| Q9Y6A4 | CFAP20 | 2053315 | 2204715 | Cilia- and flagella-associated protein 20 |

| | | | | |
|---|---|---|---|---|
| Q9Y6C9 | MTCH2 | 0 | 1943430 | Mitochondrial carrier homolog 2 |
| Q9Y6K5 | OAS3 | 0 | 595695 | 2'-5'-oligoadenylate synthase 3 |
| Q9Y6N8 | CDH10 | 0 | 1083872 | Cadherin-10 |
