# Supplementary material for: Prion‐Like Protein LENG8‐Mediated Nucleation Drives Stress Granule Assembly
Source: Adv Sci (Weinh). 2026 Sep 8:e77622. Online ahead of print. doi: 10.1002/advs.77622 (PMC13554387; doi:10.1002/advs.77622)
Supplement: Supplementary file 4 [file ADVS-9999-e77622-s003.docx]

Table S4. Primers used in this study.

| Primer name | Sequence | Usage |
| --- | --- | --- |
| *Leng8* RT-LP homo | AGCACAGATTGGTCTTCTCAGT | Quantitative real-time PCR |
| *Leng8* RT-RP homo | TGGCAGGTTCGGTGTAGGT | Quantitative real-time PCR |
| *β-actin*-LP homo | TCGTGCGTGACATTAAGGAGAA | Quantitative real-time PCR |
| *β-actin* -RP homo | AGGAAGGAAGGCTGGAAGAGT | Quantitative real-time PCR |
| *Leng8* RT-LP mouse | GTGAGCAGATGAAGTCCATTCG | Quantitative real-time PCR |
| *Leng8* RT-RP mouse | GCCAGTTCCGTAGTGATGTCT | Quantitative real-time PCR |
| *Gapdh*-LP mouse | TGGTGAAGGTCGGTGTGAAC | Quantitative real-time PCR |
| *Gapdh*-RP mouse | TCTCGCTCCTGGAAGATGGT | Quantitative real-time PCR |
| GFP-S11-LENG8- LP | TCAGAGGAGGACCTGGGCGACGGAGGCTCT | PCR amplification |
| GFP-S11-LENG8- RP | CACGTTGGCCGCCATTGTAATCCCGGCAGC | PCR amplification |
| GFP-S1-10-TIA1- LP | ACCCGCGGGCCCACCGGGGACGGCGGCAGT | PCR amplification |
| GFP-S1-10-TIA1- RP | CATCTCGTCCTCCATCTTCTCGTTTGGGTC | PCR amplification |
| NES+EGFP-LENG8^full^- LP | GAGCGCCTGACCCTGATGGCGGCCAACGTG | PCR amplification |
| NES+EGFP-LENG8^full^- RP | CAGGGGAGGCAGCATCAGGTCCTCCTCTGA | PCR amplification |
| NES+EGFP- LENG8^△Q21^- LP | GAGCGCCTGACCCTGATGGCGGCCAACGTG | PCR amplification |
| NES+EGFP- LENG8^△Q21^- RP | CAGGGGAGGCAGCATCAGGTCCTCCTCTGA | PCR amplification |
| Myc-LENG8 | TCAGAGGAGGACCTGATGGCGGCCAACGTGGGT | PCR amplification |
| Myc-LENG8 | CTCCATGGCCATAAGTCAGAAGGCTGACAGCTGC | PCR amplification |
| Myc-mCherry-LENG8 | GACGAGCTGTACAAGATGGCGGCCAACGTG | PCR amplification |
| Myc-mCherry-LENG8 | CTCCATGGCCATAAGTCAGAAGGCTGACAG | PCR amplification |
| Myc- LENG8^△Q21^ | TACCCCTACAGCTACTACTATCCC | PCR amplification |
| Myc- LENG8^△Q21^ | CTGGGACACGTACTGTGCAC | PCR amplification |
| EGFP-LENG8 | TGGACGAGCTGTACAAGTCCATGGCGGCCAA | PCR amplification |
| EGFP-LENG8 | TGAGCTCGAGATCTGAGTCCTCAGAAGGCTG | PCR amplification |
| EGFP- LENG8^△Q21^ | TACCCCTACAGCTACTACTATCCC | PCR amplification |
| EGFP- LENG8^△Q21^ | CTGGGACACGTACTGTGCAC | PCR amplification |
| EGFP- LENG8^△73-147^ | ATGGATGAGAGCATGTCCTACC | PCR amplification |
| EGFP- LENG8^△73-147^ | GTACTGTGCACTGGCCACA | PCR amplification |
| EGFP-1-147 | GCCGGGGACTGGGGGCTG | PCR amplification |
| EGFP-1-267 | AGGGTTGGGCTGGGGGCC | PCR amplification |
| EGFP-1-400 | CTTGGTGAAGCTGTTGCCCC | PCR amplification |
| EGFP-401-801 | TTTGGCAACCGCAACGTCT | PCR amplification |
| EGFP-533-801 | CGAATCCTCTACTACATCTTCACCAAG | PCR amplification |
| EGFP-666-801 | CGAATCCTCTACTACATCTTCACCAAG | PCR amplification |
| *siLeng8-1* LP | GAAGCGACCCUUUGCUGUU | PCR amplification |
| *siLeng8-1* RP | AACAGCAAAGGGUCGCUUC | PCR amplification |
| *siLeng8-2* LP | GGACAAGGACCGCACGGAA | PCR amplification |
| *siLeng8-2* RP | UUCCGUGCGGUCCUUGUCC | PCR amplification |
| *siTia1-1* LP | GCUCUAAUUCUGCAACUCUUUTT | PCR amplification |
| *siTia1-1* RP | AAAGAGUUGCAGAAUUAGAGC | PCR amplification |
| *siTia1-2* LP | GCAAGUUCCUGCAUAUGGAAUGUAUTT | PCR amplification |
| *siTia1-2* RP | AUACAUUCCAUAUGCAGGAACUUGCTT | PCR amplification |
| GST-GFP-LENG8^1-400^ LP | CTGTTCCAGGGCCCGATGGTGAGCAAGGGC | PCR amplification |
| GST-GFP-LENG8^1-400^ RP | CTCCATATGTCCTCACTTGGTGAAGCTGTT | PCR amplification |
| GST-mCherry-TIA1 LP | CTGTTCCAGGGCCCGATGGCATCAATGCAG | PCR amplification |
| GST-mCherry-TIA1 RP | GAGCTCCATATGTCCTCACTGGGTTTCATA | PCR amplification |
| Cry2-1-147 LP | TCAGAGGAGGACCTGATGGCGGCCAACGTG | PCR amplification |
| Cry2-1-147 RP | GCCTTTAGACACCATTTGGGGTGCGGATGG | PCR amplification |
| Cry2-76-96 LP | TCAGAGGAGGACCTGGCAGAAGCCTCAGCT | PCR amplification |
| Cry2-76-96 RP | GCCTTTAGACACCATGGCATAGTTGTACTG | PCR amplification |
| Cry2-73-147 LP | TCAGAGGAGGACCTGGTGTCCCAGGCAGAA | PCR amplification |
