# Supplementary material for: Prion‐Like Protein LENG8‐Mediated Nucleation Drives Stress Granule Assembly
Source: Adv Sci (Weinh). 2026 Sep 8:e77622. Online ahead of print. doi: 10.1002/advs.77622 (PMC13554387; doi:10.1002/advs.77622)
Supplement: Supplementary file 5 [file ADVS-9999-e77622-s004.docx]

**Supporting Information**

**Prion-Like Protein LENG8-Mediated Nucleation Drives Stress Granule Assembly** Mingxing Zhang^1,4^, Xin Wang^1^, Yilan Teng^1^, Zhicheng Wu^1^, Jing Fan^2^, Hongwen Zhu^3*^, Peng Dai^1,5*^

^1^Shanghai Key Laboratory of Maternal and Fetal Medicine, Clinical and Translational Research Center of Shanghai First Maternity and Infant Hospital, Frontier Science Center for Stem Cell Research, School of Life Sciences and Technology, Tongji University, Shanghai, China

^2^The Key Laboratory of Developmental Genes and Human Disease, School of Life Science and Technology, Southeast University, Nanjing, China

^3^Precise Genome Engineering Center, School of Life Sciences, Guangzhou University, Guangzhou, China

^4^These authors contributed equally to this work

^5^Lead contact

^*^Correspondence: zhw@gzhu.edu.cn; [daipeng@tongji.edu.cn](mailto:daipeng@tongji.edu.cn)

**Supplementary Figure 1** LENG8 is recruited to SGs under stress conditions. (A) Representative fluorescence images depicting the localization of LENG8 relative to stress granules, P-bodies, mitochondria and lysosomes under As treatment in HeLa cells. (B) Representative fluorescence images showing the subcellular localization of LENG8 relative to other SG markers under As treatment in HeLa cells. (C) Localization of LENG8 relative to SGs at different As concentrations in HeLa cells. (D) Representative fluorescence images revealing LENG8 localization to SGs under diverse stress conditions in HeLa cells. (E) Effects of overexpression of G3BP1, eIF2α-S51D, TIA1 and eIF2α-S51A on SG formation and LENG8 localization in HeLa cells. (F, G) Representative immunofluorescence images showing the localization of LENG8 relative to SGs during treatment with 0.1 mM (F) or 1 mM (G) As and subsequent recovery in HeLa cells. (H) LENG8 is consistently localized to SGs in different cell types under As exposure.

**Supplementary Figure 2** LENG8 acts as a nucleation factor to regulate SG assembly. (A) Effect of RNase A treatment on the recruitment/ localization of LENG8 into SGs in HeLa cells. (B) Quantification showing the effect of RNase A treatment on SG formation, corresponding to images in (A). Data are presented as mean ± SD, *n*=8. *****p*<0.0001 by Student's *t*-test. (C) qPCR results show the knockdown efficiency of *LENG8* in HeLa cells. Data are presented as mean ± SD, *n*=3. *****p*<0.0001 by Student's *t*-test. (D) Western blot results show the knockdown efficiency of LENG8 protein and its effect on G3BP1, TIA1 and p-eIF2α levels in HeLa cells. (E) Effect of LENG8 knockdown on HUR-labeled SGs in HeLa cells. (F) Effect of LENG8 knockdown on G3BP2-labeled SGs in HeLa cells. (G) Representative fluorescence images illustrating nuclear accumulation of LENG8 induced by Myc-LENG8 overexpression in HeLa cells. (H) Western blot results show the expression level of Myc-LENG8 upon overexpression in HeLa cells. (I) Representative fluorescence images showing that cytoplasmic overexpression of NES-EGFP-LENG8 induces SG formation under non-stress conditions in HeLa cells.

**Supplementary Figure 3** PrLD mediates LENG8 translocation to SGs and drives phase separation in synergy with the C-terminus. (A) Representative immunofluorescence images showing the localization of different EGFP-LENG8 mutants to SGs under As treatment in HeLa cells. (B) Percentage of cells with target protein-positive SGs among all target protein-expressing cells. Data are presented as mean ± SD, *n*=3. ****p*<0.001, *****p*<0.0001 by one-way ANOVA. (C) Western blots showing protein expression of various EGFP-LENG8 mutants in HEK293T cells. (D) Neither mCherry nor Myc tag affects the localization of LENG8 to SGs in HeLa cells. (E) Western blot analysis of the expression levels of full-length and ΔPrLD LENG8. (F) Optogenetic system for validating the phase separation capacity of PrLD-containing peptides (1-147 aa and 73-147 aa). (G) Evolutionary relationship of the PrLD sequence across different species. (H) Conservation comparison of PrLD sequences across different species.

**Supplementary Figure 4** PrLD mediates the interaction between LENG8 and TIA1 to direct LENG8 targeting to SGs. (A) GO analysis revealed the interactome of LENG8 under stress conditions in HeLa cells. (B) Overlap of LENG8 interactomes in HEK293T and HeLa cells upon As stress. (C) Pearson correlation coefficient showing co-localization patterns between LENG8, TIA1 and G3BP1 as represented in (4D、S4D、S4E). mean ± SD, *n*=20, ** *p* < 0.01, *****p*<0.0001 by one-way ANOVA. (D, E) Representative fluorescence images illustrating the localization relationship between G3BP1 and TIA1 (D) or LENG8 (E) at the indicated time points following As treatment in HeLa cells. (F) Representative fluorescence images show that ΔPrLD LENG8 fails to localize to TIA1-positive SGs in HeLa cells. (G) Split-GFP complementation assays for detecting LENG8-TIA1 interaction in HeLa cells. (H) Competition assays reveal that tandem PrLDs block the LENG8-TIA1 interaction in HEK293T cells. (I) Knockdown of TIA1 prevents colocalization of LENG8 foci with G3BP1-positive SGs in HeLa cells.

**Supplementary Figure 5** PrLD mediates LENG8 phase separation *in vitro* and fusion with TIA1 droplets. (A) ΔPrLD LENG8 (1-400 aa) cannot be recruited to SGs under As treatment in HeLa cells. (B) Deletion of PrLD significantly attenuates the LENG8 (1-400 aa)-TIA1 interaction under As stress in HEK293T cells. (C) Coomassie staining was used to assess the purity of the recombinant EGFP-LENG8 (1-400 aa), ΔPrLD EGFP-LENG8 (1-400 aa), LENG8 (1-400 aa) and mCherry-TIA1 proteins. (D) Effects of varying NaCl concentrations on droplet formation by recombinant LENG8 (1-400 aa) proteins *in vitro*. (E) Quantification of droplet area of LENG8 (1-400 aa) proteins as represented in (D). The horizontal line indicates the median, *n*=30, *****p* < 0.0001 by one-way ANOVA. (F) Effects of PEG8000 on droplet formation by recombinant LENG8 (1-400 aa) proteins *in vitro*. (G) Quantification of droplet area of LENG8 (1-400 aa) proteins as represented in (F). The horizontal line indicates the median, *n*=30, ns, not significant, *****p* < 0.0001 by one-way ANOVA. (H) Effects of varying NaCl concentrations on droplet formation by recombinant mCherry-TIA1 proteins *in vitro*. (I) Quantification of droplet area of mCherry-TIA1 proteins as represented in (H). The horizontal line indicates the median, *n*=30, *****p* < 0.0001 by one-way ANOVA. (J) Effects of PEG8000 on droplet formation by recombinant mCherry-TIA1 proteins *in vitro*. (K) Quantification of droplets area for mCherry-TIA1 proteins as represented in (J). The horizontal line indicates the median, *n*=30, ns, not significant, ***p* < 0.01, *****p* < 0.0001 by one-way ANOVA. (L) PEG8000 enhances the fusion of LENG8 droplets with TIA1 droplets *in vitro*.
